# A Novel Aldo-Keto Reductase (AKR17A1) of *Anabaena* sp. PCC 7120 Degrades the Rice Field Herbicide Butachlor and Confers Tolerance to Abiotic Stresses in *E*. *coli*

**DOI:** 10.1371/journal.pone.0137744

**Published:** 2015-09-15

**Authors:** Chhavi Agrawal, Sonia Sen, Shivam Yadav, Shweta Rai, Lal Chand Rai

**Affiliations:** Molecular Biology Section, Centre of Advanced Study in Botany, Banaras Hindu University, Varanasi-221005, India; CEA-Saclay, FRANCE

## Abstract

Present study deals with the identification of a novel aldo/keto reductase, AKR17A1 from *Anabaena* sp. PCC7120 and adds on as 17^th^ family of AKR superfamily drawn from a wide variety of organisms. AKR17A1 shares many characteristics of a typical AKR such as— (i) conferring tolerance to multiple stresses like heat, UV-B, and cadmium, (ii) excellent activity towards known AKR substrates (isatin and 2-nitrobenzaldehyde), and (iii) obligate dependence on NADPH as a cofactor for enzyme activity. The most novel attribute of AKR17A1, first reported in this study, is its capability to metabolize butachlor, a persistent rice field herbicide that adversely affects agro-ecosystem and non-target organisms. The AKR17A1 catalyzed- degradation of butachlor resulted into formation of 1,2-benzene dicarboxylic acid and 2,6 bis (1,1, dimethylethyl) 4,-methyl phenol as the major products confirmed by GC-MS analysis.

## Introduction

Paddy fields are naturally endowed with plenty of biological nitrogen fixers like cyanobacteria which continue to present a self regenerating system important for soil health and productivity. Currently, the world's increasing population vis-à-vis accelerated food demand calls for augmented paddy cultivation which requires sustainable pest management through judicious use of agrochemicals. Rice paddies are often subjected to heavy herbicide application which negatively affects non-target organisms including cyanobacteria. Our earlier work on butachlor, a common rice field herbicide vividly demonstrated its negative impact on the physiology and metabolism of *Anabaena* spp. [1]. Though butachlor is a pre-emergent herbicide that controls annual grasses and broadleaf weeds, its ill-effects on environment and non-target organisms [1–7] cannot be overlooked.

Butachlor is a highly stable and persistent herbicide [8] with a typical field half-life of 18–19 days [9, 10]. Hence there is a need to underscore the factors which regulate its dissipation from soil. There are various reports cataloguing the influence of soil types [11], moisture condition [12], organic matter content [12, 13], and microbial activity [14, 15] on butachlor's persistence in soil. However, microbial transformation/degradation is considered the most promising route for the minimization of pesticides from soil [16]. The role of some bacterial isolates such as *Stenotrophomonas acidaminiphila* [17], *Rhodococcus* sp. strain B1 [18], *Catellibacterium caeni* sp. [19], *Mycobacterium* sp. J7A and *Sphingobium* sp. J7B [20] in butachlor degradation has been witnessed. However, nothing is known about cyanobacterial strains or their enzymes in degradation of butachlor. Since cyanobacteria are directly affected by repeated herbicide application, how they manage to survive under butachlor stress is a question worth asking. Organisms usually possess a battery of enzymes which metabolize toxic substances produced under stress. Our proteomic study [1] demonstrated enhanced accumulation of a number of stress responsive proteins including aldo-keto reductase (All2316) in *Anabaena* sp. PCC 7120 when exposed to butachlor stress.

Aldo-keto reductases (AKRs), in general, are a group of structurally related proteins with similar kinetics and wide distribution across all phyla ranging from prokaryotes, protozoans, yeast, plants, animals to humans. These proteins together form a growing aldo-keto reductase superfamily consisting of 15 families (<40% amino acid identity with other families, >60% amino acid identity among subfamilies) with diverse metabolic functions (http://www.med.upenn.edu/akr/). All members of AKR family known till date possess a conserved (β/α)_8_ or TIM (triosephosphate isomerase) barrel motif which provides a common scaffold for an NAD(P)(H)-dependent catalytic activity and a number of variable loops and helixes that determine substrate specificity [21]. The spectrum of AKR’s substrate is wide catalyzing redox transformation of a variety of carbonyl compounds including glucose, glucocorticoids, smaller carbonyl metabolites, glutathione conjugates, lipid peroxidation products, drugs, environmental pollutants, pesticides and xenobiotics [21–24]; however, their precise physiological function is still unclear. A number of plants AKRs are known to be stress regulated and have been exploited to produce stress resistant transgenic lines [25–28]. Positive role of AKR in detoxification of reactive carbonyl species (RCS) produced under oxidative stress, such as methylglyoxal, HNE and TBARS has also been witnessed [29, 30] and linked with its ability to confer tolerance against abiotic stresses [27, 31, 32]. A few recent proteome based studies in cyanobacteria also demonstrated enhanced accumulation of *Anabaena* AKR under a variety of stresses such as butachlor [1], salt [33], UV-B [34] and cadmium [35] hence indicating its physiological role in abiotic stress management. However, in diazotrophic cyanobacteria these important gene family candidates have never been investigated. *Anabaena* sp. PCC 7120 (also known as *Nostoc* sp. PCC 7120, from here onwords *Anabaena* 7120) represents a suitable model for plant and agriculture based research, because in addition to being photoautotrophic, it possesses the most desirable agricultural trait e.g., N_2_ fixation. In view of having a fully sequenced genome, it was selected for the present study. This study first time examines the potential of a butachlor responsive aldo-keto reductase (All2316) of *Anabaena* 7120 [1] in (i) metabolism of an agriculturally relevant chloroacetanilide herbicide butachlor, and (ii) development of stress resistant transgenic lines. Based on our experimental findings we demonstrate that ORF all2316 encoding aldo-keto reductase of *Anabaena* 7120 not only has potential to combat abiotic stresses but also plays important role in butachlor degradation, thus identifying it as a promising gene to produce transgenics capable of stress tolerance and butachlor degradation.

## Experimental Procedures

### 1. Homology search and evolutionary relationships

Protein sequences of all annotated aldo-keto reductases listed in AKR superfamily homepage (https://www.med.upenn.edu/akr/) were searched for protein homologous with AKR (All2316) of *Anabaena* 7120 by using BLASTP [36] algorithm. Evolutionary analyses were conducted in MEGA6 [37] using the Neighbor-Joining method [38]. The optimal tree with the sum of branch length = 27.84402135 is shown. The percentage of replicate trees in which the associated taxa clustered together in the bootstrap test (1000 replicates) is shown next to the branches [39]. The tree is drawn to scale, with branch lengths in the same units as those of the evolutionary distances used to infer the phylogenetic tree. The evolutionary distances were computed using the p-distance method [40] and are in the units of the number of amino acid differences per site. The analysis involved 156 amino acid sequences. All ambiguous positions were removed for each sequence pair. There were a total of 1125 positions in the final dataset.

### 2. Cyanobacterial and bacterial strains and plasmids

*Anabaena* 7120 was grown photoautotrophically in BG-11medium [41] buffered with 10 mM HEPES-NaOH, pH 7.5 at 24±2°C under day light fluorescent tubes emitting 72 μmol photon m^−2^ s^−1^PAR (photosynthetically active radiation) light intensity with a photoperiod of 14:10 h. *E*. *coli* strains DH5α and BL21 (DE3) (Novagen) were used as host for cloning and over-expression respectively. *E*. *coli* cultures were stored as 10% (v/v) glycerol stocks at −80°C and maintained on Luria–Bertani (LB) plates at 37°C containing 1.5% (w/v) agar. Cells harboring recombinant plasmids were grown and maintained on LB medium supplemented with 100 μg/ml ampicillin [42]. Plasmid pET21a (Novagen) was used as a vector for cloning.

### 3. AKR transcript analysis

Total RNA was extracted from 50 mL culture (OD_750nm_ 0.6) of *Anabaena* 7120 before and after 1 day of butachlor (32μM) [1], Cd (10 μM) [43], NaCl (100mM) [33], As (40mM) [44], heat (42°C for 1 h) [45], UV-B (12.9 m Wm^-2^ nm^-1^ for 30 min) [46], or desiccation (30°C for 10h) [47] treatment using the RNASure mini kit (Nucleo-pore). One microgram of total RNA was reverse transcribed in a 20μl reaction mixture using the iScript cDNA synthesis kit (BioRad). For the transcript analysis, following gene specific primer sets were designed using primer3 software: 5’-GGAATCCAGAACATCTGCGT-3’ and 5’-CCCACAAATCGAATCAAACC-3’ for *akr* and 5’-CACACTGGGACTGAGACAC-3’ and 5’-CTGCTGGCACGGAGTTAG-3’ for reference 16S rRNA gene. 15ng of cDNA extracted from each sample was used for quantitative real-time PCR (qRT-PCR). Reactions were performed in a total volume of 20μl including 10 pmol of forward and reverse primers and 1x Sso fast evagreen qPCR supermix (BioRad). CFX-96 (Bio-Rad) was used for PCR and for detection of fluorescence change. Transcript levels were normalized to 16S transcript and calculated relative to 0 h using the 2^−ΔΔCt^ method. The comparative ΔΔCt method was used to evaluate the relative quantities of each amplified product in the samples. The threshold cycle (Ct) was automatically determined for each reaction by the system set with default parameters. The specificity of the PCR was determined by melting curve analysis of the amplified products.

### 4. Cloning of aldo/keto reductase in *E*. *coli* BL21

For cloning and over-expression analysis, genomic DNA from *Anabaena* 7120 was extracted following the protocol of Srivastava et al. [48]. The open reading frame *all2316*, encoding aldo/keto reductase was amplified by polymerase chain reaction using genomic DNA as the template. The PCR primer pairs used were Pf: 5′ GGAGGAGCATATGGAAACTACACAGCTAG 3′ and Pr: 5′ CCAGTCTCGAGAATTTTCTGGACTTCTTCG 3′ (the Nde1 and Xho1 recognition sites are underlined). PCR was done with a temperature program starting at 98°C for 30 sec, followed by 30 cycles of 98°C for 10 sec, 52°C for 30 sec, 72°C for 30 sec and a final elongation at 72°C for 7 min. The reaction was performed with a final concentration of 100ng DNA, 5μl of 5× Phusion HF Buffer (NEB), 200μM dNTPs, 0.5μM forward and reverse primer and 0.5U Phusion DNA polymerase (NEB) in an iCycler (Bio-Rad, USA). The amplified product was gel purified using a QIAquick gel extraction kit (Qiagen). The purified PCR product was digested with Nde1 and Xho1 (NEB), and the resultant DNA fragment was cloned into the pET-21a expression vector (Novagen), digested with the same restriction enzymes. pET21a incorporates a C-terminal histidine (6x His) tag to aid purification. After ligation the construct pET-21a –AKR and empty vector pET-21a were introduced into *E*. *coli* strain BL21 (DE3) for expression studies. The recombinant plasmid was isolated and the DNA sequence of AKR was confirmed by sequencing. Prior to use in further experimental procedures, transformation was confirmed by colony PCR analysis for the targeted AKR gene and by Nde1 and Xho1 double digestion on plasmid isolated from the cell transformed with empty and recombinant vector.

### 5. Over-expression and purification of recombinant protein

BL21 (DE3) cells transformed with recombinant plasmid pET-21a–AKR were grown overnight in Luria-Bertani (LB) medium containing 100 μg/ml ampicillin. Overnight culture was diluted 1:100 in fresh LB broth (5x 100ml culture) containing 100μg/ml ampicillin and then incubated at 37°C for 3–4 h (or till OD_600_ reached ~0.7). Once the desired optical density reached the cultures were cooled at RT and induced with a final concentration of 0.05mM IPTG by incubating at 16°C for 16/17 h and shaking at 200 rpm. After incubation, cells were harvested by centrifugation, resuspended in lysis buffer containing 20mM sodium phosphate (pH 7.4), 0.5M NaCl and 40mM imidazole, and disrupted by grinding in the presence of 10 μM PMSF (Sigma), under liquid nitrogen. The cell debris was pelleted by centrifugation at 4°C and 20,000g for 30 min. The supernatant containing the recombinant His-tagged fusion protein was purified using PureProteome Nickel Magnetic Beads (Merck Millipore) by following the manufacturer’s instructions. To assess the protein purity, collected fraction was visualized on 12% SDS-PAGE stained with Coomassie Brilliant Blue R250. The purified protein was subjected to MALDI-TOF MS/MS analysis to confirm its identity. The relative protein concentration was measured by determination of its absorbance at 280 nm.

### 6. Western blotting

Western blotting was performed as described by Pandey et al [49]. Whole cell lysate of *E*. *coli* cells containing the pET-21a-AKR before and after IPTG induction was loaded on a 12% SDS–PAGE gel and blotted onto Immobilon-P PVDF membrane (Millipore, Billerica MA). The membrane was probed with penta-His antibody (mouse monoclonal, Qiagen) as primary and peroxidase-labelled anti-mouse antiserum as secondary antibody and visualized by ECL Western Blotting Analysis System (Amersham Bioscience).

### 7. Measurement of multiple stress tolerance by spotting and liquid assay

The effect of abiotic stresses on *E*. *coli* BL21(DE3) strains transformed with empty pET 21a and pET 21a-AKR was analyzed using spot assay in different treatments of cadmium (CdCl_2_), heat, UV-B, arsenic (Na_3_AsO_4_), mannitol (for drought) or NaCl. The transformed cells were grown in LB medium supplemented with 100 μg/ml ampicillin at 37°C. When OD600 reached 0.6, IPTG was added to a final concentration of 0.05 mM, and allowed to grow for additional 5–6 h at 16°C. Thereafter, the cultures were serially diluted to three levels (10^−1^, 10^−2^ and 10^−3^). 3μl from each dilutions was spotted on LB-ampicillin plates containing CdCl_2_ (0.1, 0.2 and 0.3 mM), Na_3_AsO_4_ (4, 6 and 8mM), mannitol (0.4, 0.6 and 0.8 M) or NaCl (0.4, 0.6 and 0.8 M). All these plates were incubated at 37°C overnight and photographed. For heat (50°C) and UV-B treatment (12.9 m Wm^-2^ nm^-1^), induced *E*. *coli* cells were first exposed for different time intervals (30m, 1h and 2h for heat and 10m, 15m and 20m for UV-B), then spotted on LB-ampicillin plates and incubated at 37°C overnight.

Growth of transformed *E*. *coli* cells was also examined by liquid culture assay as given in Shrivastava et al [50]. Briefly, single colony of *E*. *coli* cells transformed either with empty vector (pET 21a) or recombinant plasmid (pET 21a-AKR) was inoculated in tubes containing fresh LB medium spiked with 100 μg ml^−1^ ampicillin and grown overnight. 200 μl inoculum (OD_600 nm_ 0.5) was added into 50 ml LB medium having CdCl_2_ (0.3mM), NaCl (600 mM), mannitol (600mM) or Na_3_AsO_4_(6mM) and 0.2 mM IPTG, and incubated at 37°C with shaking (200 rpm). For UV-B stress, the inoculum already exposed to UV-B radiation (12.9 m Wm^-2^ nm^-1^) for 10 min was used. For heat stress, the 50 ml LB medium having 200 μl inoculum (OD_600 nm_ 0.5) and 0.2 mM IPTG was incubated at 50°C with shaking (200 rpm). The aliquots were removed from each treatment every 2 h and absorbance at 600 nm was recorded for 24 h.

### 8. Enzyme assay and measurement of kinetic parameters

Aldo-keto reductase activity was assayed spectrophotometrically as per the method of Smiley and Bolen [51]. Assay was performed at 37°C in a standard reaction mixture (1mL) containing 50mM potassium phosphate buffer (pH 7.4), 0.12mM NADPH, 10mM 2-mercaptoethanol, 4–20μg of purified protein and a range of substrates such as xylose (50 mM), arabinose (50 mM), benzaldehyde (10 mM), ethylpyruvate (10 mM), methylglyoxal (0.5 mM), butachlor (0.5 mM), isatin (0.5 mM), or o-nitrobenzaldehyde (0.5 mM). Activity was monitored by measuring the decrease in NADPH absorbance at 340 nm and the NADPH oxidation was calculated using a molar extinction coefficient of 6,200 M^-1^ cm^-1^ [49]. The kinetic parameters of the purified AKR (*K*_*m*_, k_cat_, k_cat_/*K*_*m*_ values) were calculated by non-linear regression of Michaelis–Menten data at a various concentrations of substrates (final concentrations: benzaldehyde, 1–50 mM; ethylpyruvate, 5–50 mM; methylglyoxal, 1–20 mM; isatin, 0.1–0.5 mM; o-nitrobenzaldehyde, 0.1–1.0 mM; butachlor, 0.1-.05 mM), using the GraphPad PRISM 5.04 version program (http://www.graphpad.com). All measurements were performed in three replicates, and the experiment without AKR under the same condition was used as control.

### 9. Measurement of butachlor degradation

In order to monitor the butachlor degrading potential of AKR17A1, the reaction mixtures were prepared in four variants which contained along with 50mM potassium phosphate buffer pH 7.4, 10 mM 2-mercaptoethanol and 0.1 mM butachlor, (i) neither NADPH nor AKR, (ii) NADPH (0.12mM), (iii) AKR (5 μg), or (iv) both NADPH (0.12mM) and AKR (5 μg). Each sample was scanned separately using UV-visible spectrophotometer (Hitachi U-2910) within the wave length range of 200–400nm against 50mM potassium phosphate buffer (pH 7.4) as baseline reference. The absorption spectra of each sample was measured in three replicates with a scanning speed of 3600 nm min^-1^ and a band width of 2.0 nm equipped with 1cm matched quartz cell.

### 10. Gas chromatography–mass spectrometry (GC–MS) analyses

To further attest the role of AKR17A1 in butachlor degradation and identify the degradation products if any, the reaction mixtures were prepared as described above (see section 9 in Experimental procedures). Samples were prepared in 10ml reaction volume. After incubation, the mixture was extracted with 20ml hexane. The hexane fraction was then filtered using 0.45 μm syringe filter and concentrated to 1 ml at 35~40°C under vacuum. The concentrate was injected into the GC–MS (Shimadzu QP-2010 Plus with thermal desorption system TD20) system equipped with an RTx-5Sil MS column (30 m × 0.25 mm i.d. × 0.25 μm). Helium was used as carrier gas with a flow rate of 1.21 ml min^-1^. Oven parameters were 120°C for 3 min, raised to 280°C at the rate of 10°C min^-1^ and held for 16 min. The injector was kept at 260°C. Pressure was 99.1 kPa, linear velocity 41.3 cm s^-1^, purge flow 3.0 ml min^-1^ and split ratio 10.0. The MS was operated in electron ionization (EI) mode at 70 eV in an m/z range of 40–600 amu and ion source temperature as 230°C. Identification of metabolites was done by comparing their mass spectra with those of the spectrophotometer database using the NIST08, and WILEY8 Libraries. The identification of compounds was confirmed by comparison of the fragmentation pattern and their retention indices (RI).

## Results

### 1. Identification of All2316 as an entirely new family protein AKR17A1

**Fig 1:**
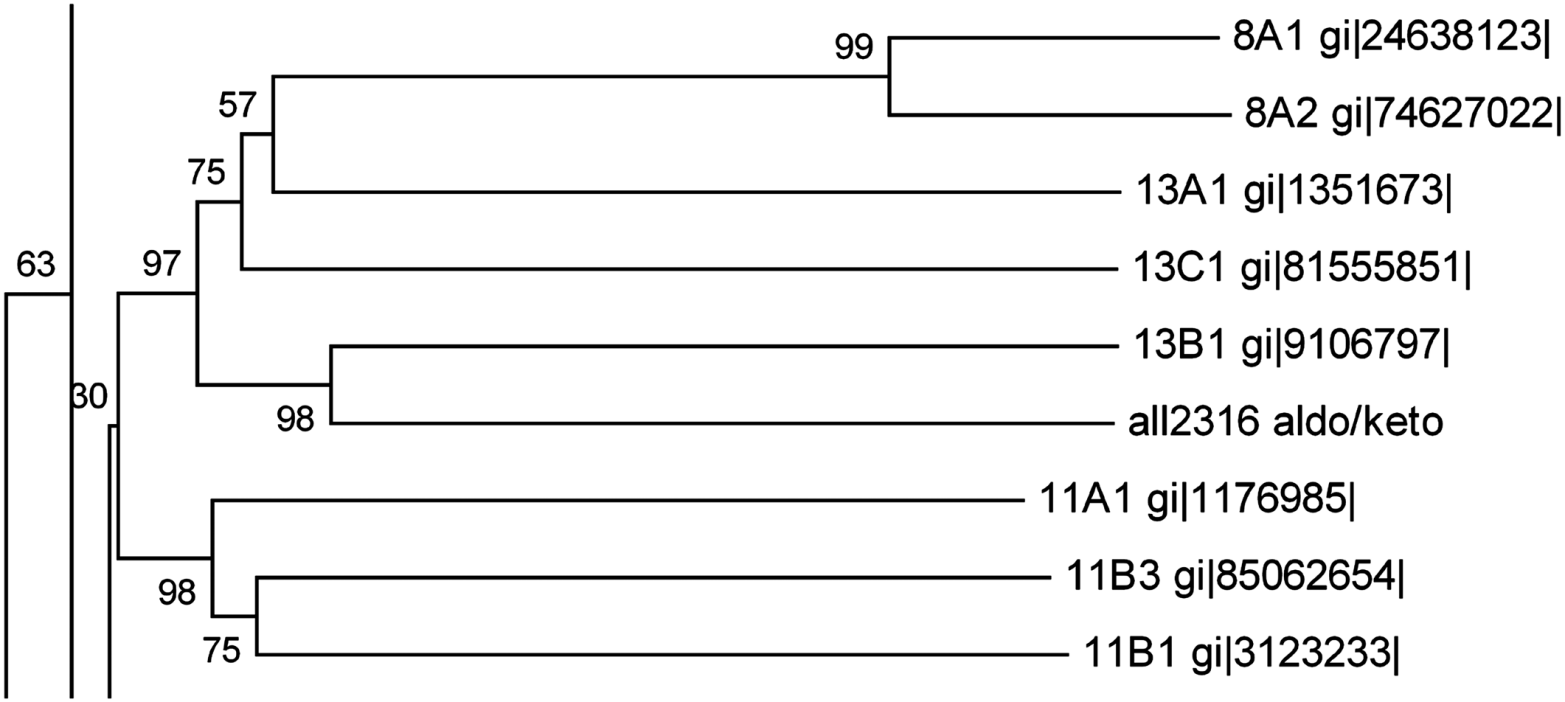
A segment of phylogenetic tree representing the relationship of ORF All2316 encoding *Anabaena* 7120 AKR to other closely related members of the AKR superfamily. Analysis involved all annotated AKRs (155 proteins) that fall into 15 families of AKR superfamily along with the new AKR from *Anabaena* 7120. The complete tree and the sequence alignment of the entire AKR protein sequences are given in supporting information (S1 and S2 Files). Full names for the sequences used in this figure are available on the AKR homepage (http://www.med.upenn.edu/akr).

**Fig 2:**
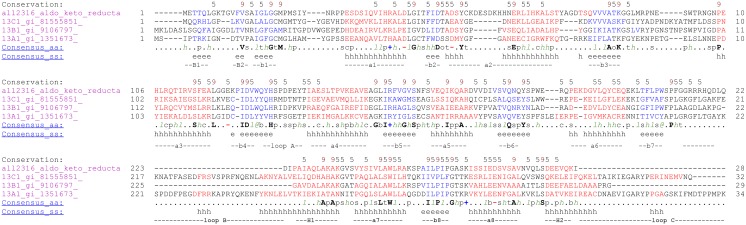
Sequence alignment of *Anabaena* 7120 aldo- keto reductase All2316 and members of AKR13 family using PROMALS3D multiple sequence and structure alignment server (prodata.swmed.edu/promals3d/). Representative sequences are colored according to predicted secondary structures (red: alpha-helix, blue: beta-strand). Consensus predicted secondary structure symbols: alpha-helix: h; beta-strand: e. Consensus amino acid symbols are: conserved amino acids are in bold and uppercase letters; aliphatic (I, V, L): l; aromatic (Y, H, W, F): @; hydrophobic (W, F, Y, M, L, I, V, A, C, T, H): h; alcohol (S, T): o; polar residues (D, E, H, K, N, Q, R, S, T): p; tiny (A, G, C, S): t; small (A, G, C, S, V, N, D, T, P): s; bulky residues (E, F, I, K, L, M, Q, R, W, Y): b; positively charged (K, R, H): +; negatively charged (D, E):-; charged (D, E, K, R, H): c.

Aldo-keto reductase encoded by ORF *all2316* was previously identified as butachlor responsive protein in the proteome of *Anabaena* 7120. Sequence analysis revealed that All2316 contained AKR signature motifs such as active side residues and catalytic tetrad (Asp50, Tyr55, Lys88, and His130) which are generally conserved among aldo-keto reductases [21]. For homology search, the All2316 protein sequence having 284 amino acids was compared with the members of all existing AKR families (AKR1-15). Unexpectedly, no proteins belonging to the AKR superfamily showed more than 40% homology. Although, All2316 showed close evolutionary relationships with AKR13B1 and assembled together by forming sister clade with AKR13A1 and AKR13C1 (Fig 1), the sequence similarity in terms of percent identity revealed that All2316 is only 40% identical to the AKR13B1. While with 13A1 and 13C1, it shows only 29% and 27% identity respectively. Furthermore, the comparison of amino acid sequences with the members of AKR13 family indicated that the sequences of the N-termini and loop regions of All2316 are also different from AKR13(s) (Fig 2). In view of the above, All2316 cannot be grouped with AKR13 family hence assigned a new family. The amino acid sequence of All2316 was thus submitted to the AKR superfamily web site (http://www.med.upenn.edu/akr/). Owing to low sequence identity with any of the existing AKR members the website recommended that protein be assigned an entirely new family AKR17 and referred to as AKR17A1.

### 2. Response of AKR17A1 to a variety of abiotic stresses

**Fig 3:**
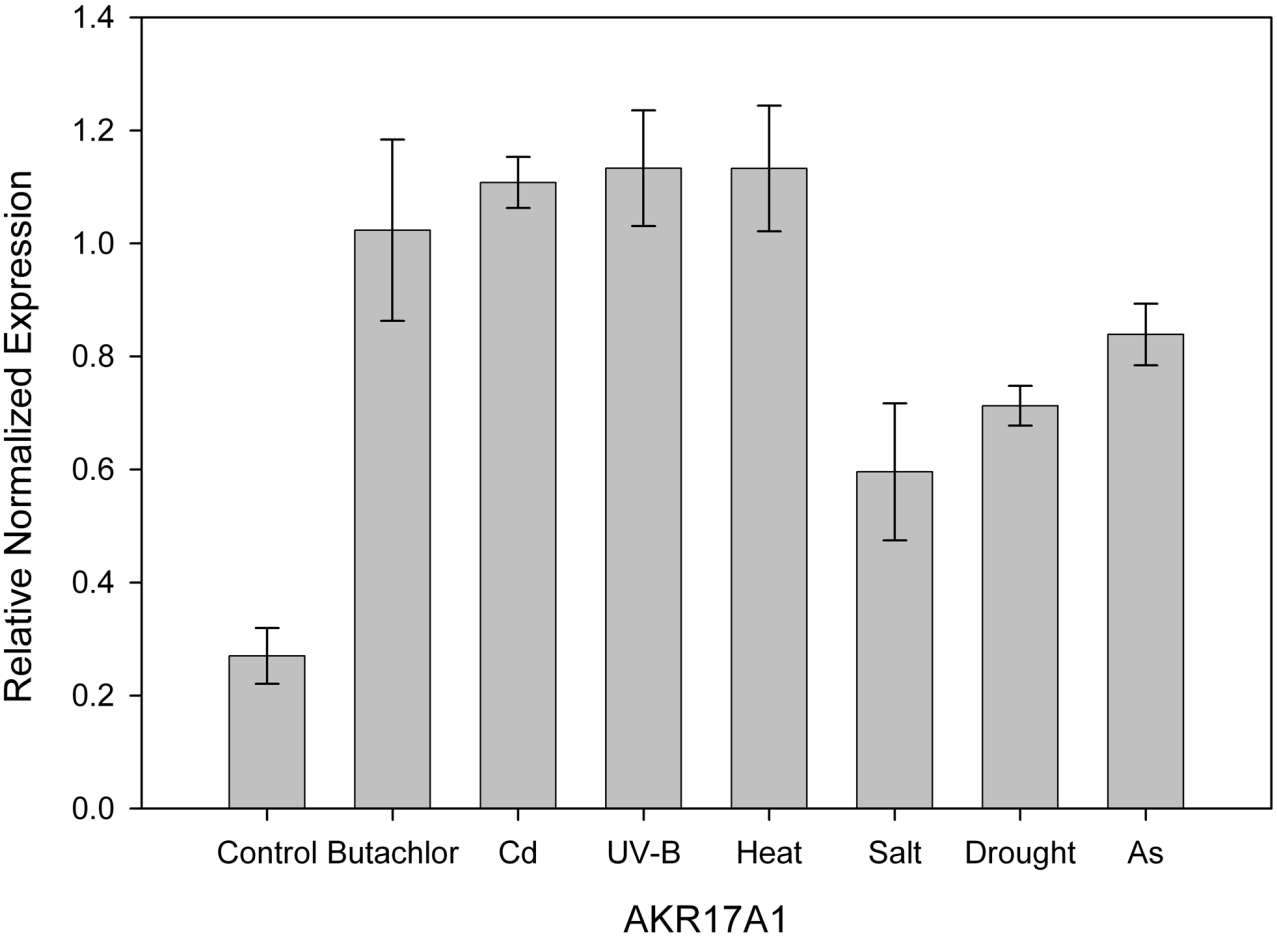
Relative normalized expression of AKR17A1 in *Anabaena* 7120 exposed to butachlor, cadmium, UV-B, heat, salt, drought and arsenic. Transcript levels were determined by qRT-PCR. The 16S rRNA gene was used as an internal control for normalizing the variations in cDNA amounts. Biological triplicates were averaged. Bars indicate SE.

To study the stress response of AKR17A1, *Anabaena* 7120 was subjected to different abiotic stresses such as butachlor, cadmium, UV-B, salt, heat, drought and arsenic. Our previous proteomic studies showed increased accumulation of AKR under butachlor [1], Cd [35], UV-B [34] and salt stress [33] which is also reflected in quantitative PCR analysis of AKR17A1 displaying 3.8, 4.1, 4.2 and 2.2 fold induction respectively. In line with the above results, the transcript level for *AKR17A1* was also elevated upto 4.2, 2.6 and 3.1 fold under heat, drought and arsenic stress respectively (Fig 3 and Table A in S3 File).

### 3. AKR17A1 confers tolerance against abiotic stresses

**Fig 4:**
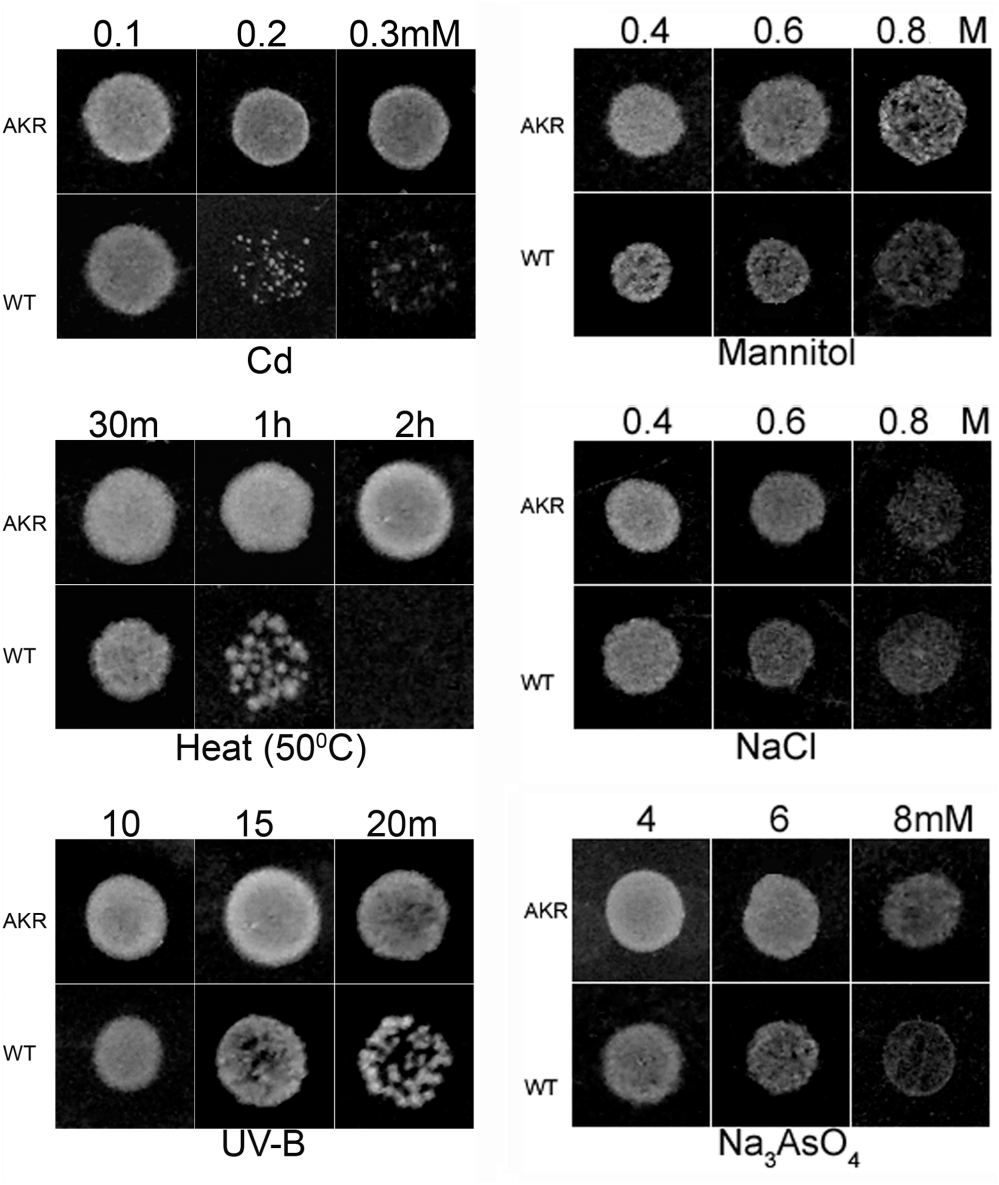
Spot assay of *E*. *coli* strain BL21 (DE3) transformed with vector alone (BL/pET21a) and recombinant plasmid (BL/ pET21a-AKR) under different abiotic stresses. 3 μL of sequential dilutions (only growth at dilution level 10^−2^ is shown here) were spotted on the Luria Bertani (LB) plates supplemented with varying concentration of (a) cadmium, (b) mannitol, (c) arsenic and (d) NaCl. For (e) UV-B and (f) heat (50°C),cells were pretreated for different time intervals and then spotted on the LB plates. All spot tests were performed in triplicate.

The stress tolerance potential of AKR17A1 was tested against a range of abiotic stressors such as cadmium (CdCl_2_), arsenic (Na_2_AsO_4_.7H2O), drought (mannitol), salinity (NaCl), heat (50°C) and UV-B by spot and liquid culture assay. For spot assay, growth of bacterial colonies at dilution level 10^−2^ producing the most significant differences has been displayed as Fig 4. The *E*. *coli* strain BL21 (DE3) transformed with recombinant plasmid (pET21a-AKR) and vector alone (control) following spotting on LB medium pretreated with cadmium, heat, and UV-B produced a significantly higher number of BL/pET21a-AKR colonies compared to BL/pET21a (Fig 4). However, the growth of BL/pET21a-AKR was almost similar to BL/pET21a in arsenic, salt and mannitol containing media (Fig 4).

**Fig 5:**
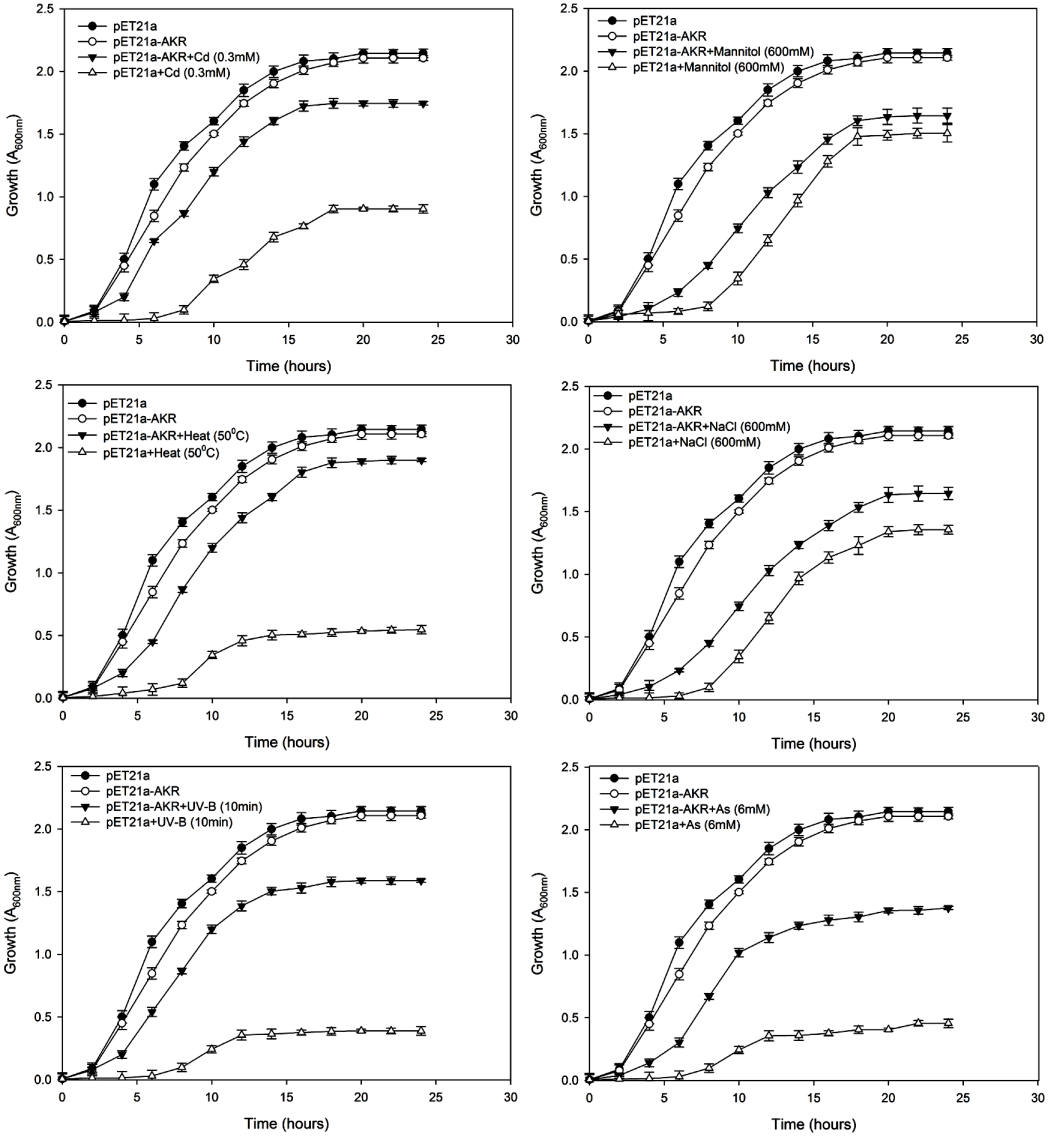
Growth behavior of *E*. *coli* BL21 (DE3) cells transformed with pET21a and pET21a-AKR in response to (a) cadmium (0.3 mM) (b) mannitol (600 mM), (c) heat (50°C), (d) NaCl (600 mM), (e) UV-B (10 min) and (f) arsenic (6 mM) stress using liquid culture assay. The mean of three independent replicates is plotted with error bars indicating standard deviations. (a-f) represents the growth curves of *E*. *coli* strains in LB medium supplemented with 100 μg ml^−1^ ampicillin and 0.2 mM IPTG.

It is worth mentioning that the growth pattern of the above cells in LB liquid medium showed similar pattern as observed with spot culture assays. pET21a-AKR depicted 81, 34, 31.2, 28.3, 19 and 10.2% better specific growth rate and 45, 25.5, 23.7, 22, 16.2 and 9% decrease in doubling time under CdCl_2_, UV-B, heat (50°C), arsenic, mannitol, and NaCl stress respectively when compared to control. These results indicate that the expression of AKR17A1 gene significantly increased the Cd, UV-B, and heat tolerance of *E*. *coli* cells (Fig 5).

### 4. Butachlor metabolism and substrate specificity of AKR17A1

**Fig 6:**
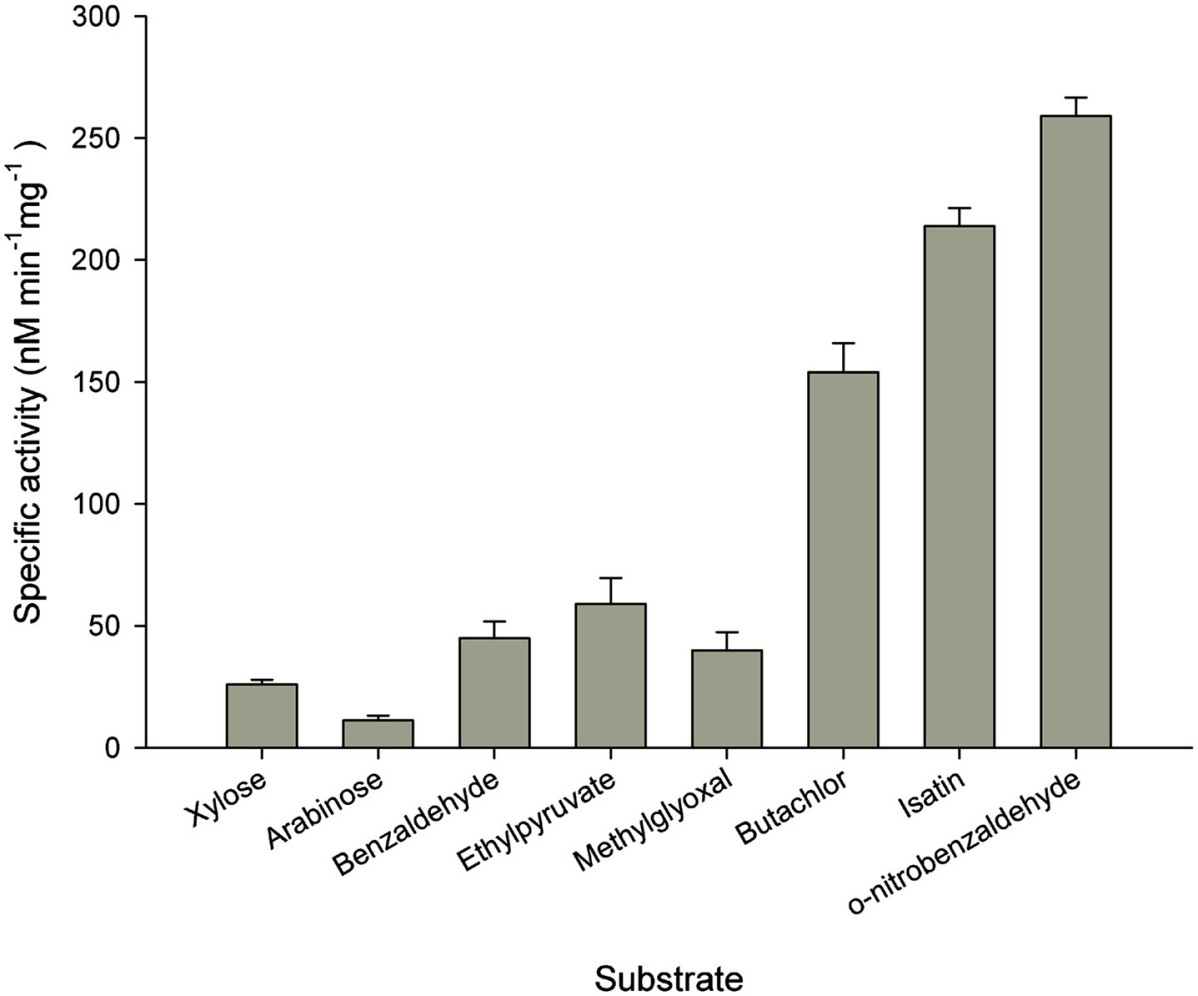
Specific activity of recombinant AKR17A1 on diverse substrates. Data represents the average of three measurements and bars indicate ±SD.

AKRs catalyze the reduction of aldehydes, ketones, dicarbonyls, steroids, and monosaccharides in an NAD(P)(H)-dependent manner [21]. To determine the substrate specificity and butachlor metabolizing potential of AKR17A1, the enzymatic activity of purified protein (Figures of coomassie stained 12% SDS-PAGE gel showing purified protein and immunoblot detection of recombinant protein AKR17A1 are given as Figs A and B in S3 File) against butachlor and other typical AKR substrates including aldehydes [52, 30], ketones [53], bicarbonyl [29, 54, 55], and sugars [56] was tested. Except for sugars, arabinose and xylose, AKR17A1 exhibited considerable reductase activity for all the substrates tested (Fig 6).

The best substrate studied in terms of highest specificity constant (K_cat_/K_m_) was isatin, an α-dicarbonyl tryptophan metabolite (Table 1). AKR17A1 also has ability to reduce o-nitro derivative of benzaldehyde with high efficiency (Table 1). Since, benzaldehyde as a substrate has high K_m_ and low K_cat_/K_m_ when compared to o-nitro benzaldehyde, 2-nitro group seems essential for high substrate activity. Methylglyoxal and ethyl pyruvate were poor substrates with high K_m_ and low K_cat_/K_m_. Extremely low enzyme activity with the sugars arabinose and xylose, suggests that these could be unlikely substrates of AKR17A1 in vivo. The most interesting observation was that AKR17A1 catalyzed the reduction of butachlor with the highest k_cat_ and considerably higher specificity constant (K_cat_/K_m_) thereby indicating its propensity to reduce chloroacetamide substrates as well (Table 1).

**Table 1:** Steady-state kinetic parameters of recombinant His-tagged AKR17A1. Kinetic constants are mean values of three independent experiments. Values in parentheses indicate standard error of the mean (SEM). The activity of AKR17A1 at various concentrations of substrates tested is given as Fig C in S3 File.

Substrate	k_cat_ (SEM) (min^-1^)	*K*_*m*_ (SEM) (mM)	k_cat_ /*K*_*m*_ (M^-1^min^-1^)
Benzaldehyde	90.68 (4.23)	3.740 (1.098)	2.424×10^4^
o-nitro benzaldehyde	95.50 (3.57)	0.014 (0.002)	68.12×10^5^
Methylglyoxal	96.01 (1.24)	4.164 (0.973)	2.3×10^4^
Isatin	94.64 (8.25)	0.011 (0.005)	81.79×10^5^
Ethyl pyruvate	85.51 (2.47)	6.213 (2.214)	1.37×10^4^
Butachlor	126.4 (7.79)	0.548 (0.190)	23.06×10^4^

### 5. Spectroscopic analysis affirms butachlor degrading potential of AKR17A1

**Fig 7:**
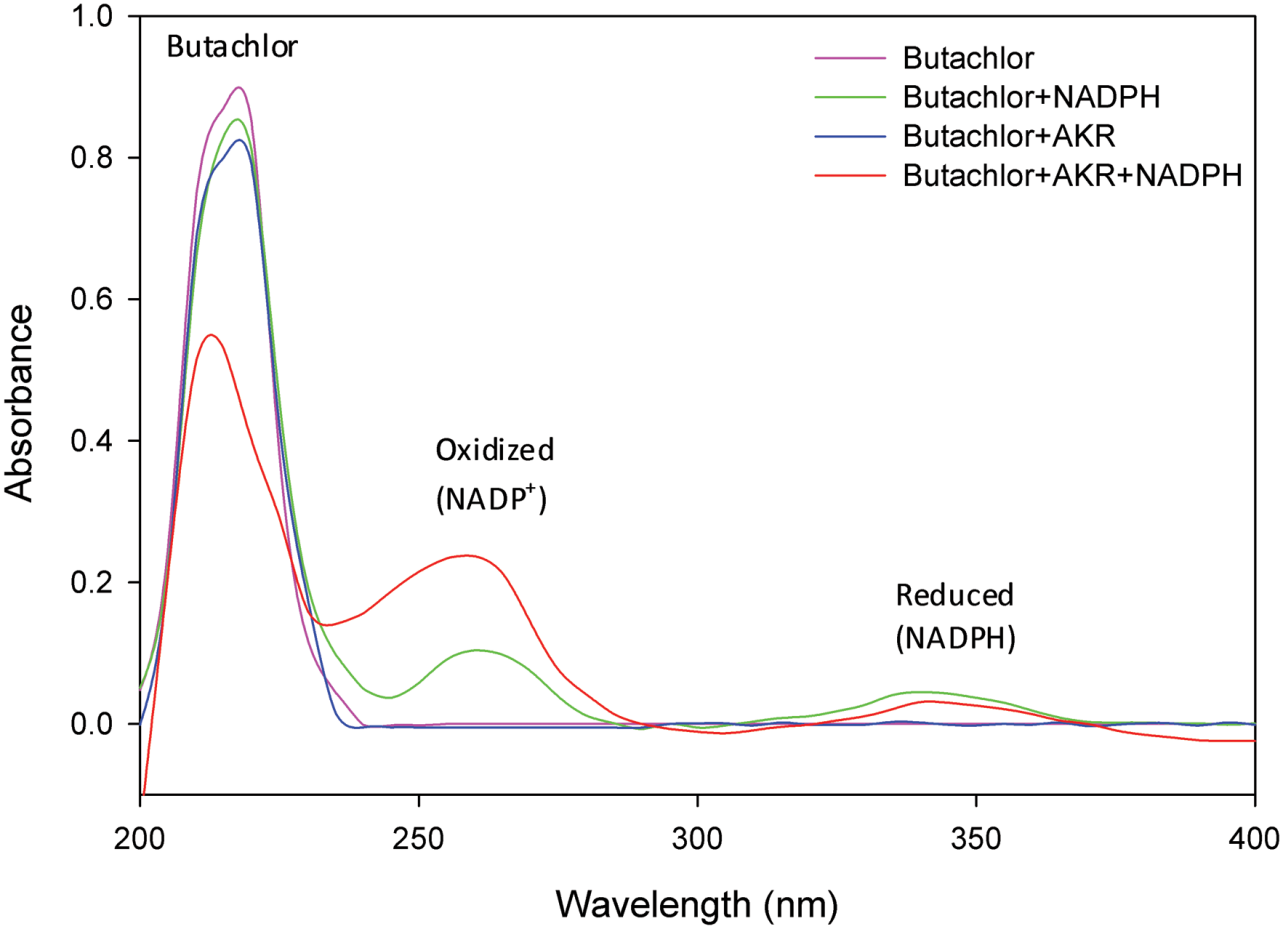
Absorption spectrograms showing degradation of butachlor by NADPH dependent AKR17A1. The analysis was performed in triplicates. The absorption spectra for each samples was recorded in 1 mL sample volume containing 50 mM potassium phosphate buffer (pH 7.4), 10 mM β-mercaptoethanol, 0.1 mM butachlor and either 0.12 mM NADPH, 5μg AKR or both.

Butachlor exhibited a characteristic absorption maximum in UV-C region at a wavelength near 215 nm. The presence of co-substrate NADPH or enzyme AKR17A1 alone did not have any significant effect on peak height of butachlor. Whereas in the presence of both NADPH and AKR17A1, the absorption peak of butachlor (at 215 nm) was decreased by 0.87 to 0.53 and of oxidized NADPH (near 261nm) was increased by 0.104 to 0.237 (Fig 7). This affirms the role of AKR17A1 in butachlor degradation and also indicates a strict requirement of cofactor NADPH for AKR17A1 because no significant activity was observed in its absence.

### 6. AKR17A1 metabolizes butachlor into benzenedicarboxylic acid and phenol

**Fig 8:**
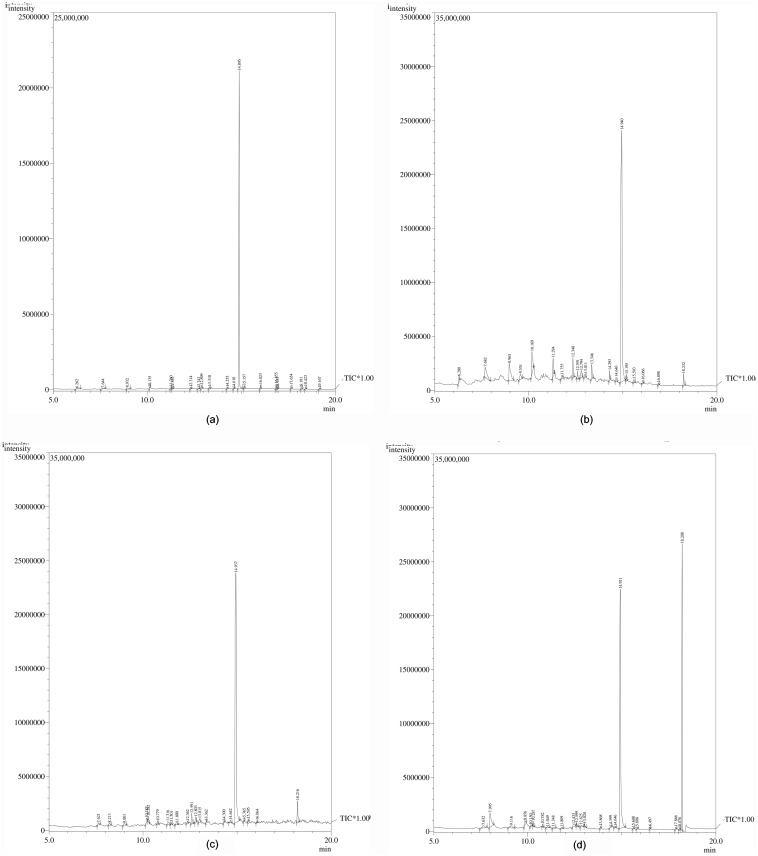
GC charts of the reaction products of AKR17A1 with butachlor used as substrate. (a) Control assay devoid of enzyme AKR17A1 and cofactor (NADPH); (b) reaction with NADPH alone; (c) enzymatic reaction with AKR17A1 alone; and (d) enzymatic reaction with both AKR17A1 and cofactor NADPH.

To identify the degradation products formed following the reaction of AKR17A1 on butachlor, GC-MS analysis was performed in 4 independent reactions. In the absence of both NADPH and AKR17A1 (reaction 1), the chromatogram showed a single major peak ‘A’ (Rt_min_ = 14.9) which corresponds to butachlor. NADPH alone (reaction 2) also showed a similar peak for butachlor (peak A). While, AKR17A1 alone (reaction 3) gave two major peaks of which peak A corresponds to butachlor and peak B to 1,2-benzene dicarboxylic acid; these constituted 80.56% and 4.49% of total peak area respectively. Interestingly, however, reaction 4 which contained both AKR and NADPH showed three major peaks (Fig 8) (i) a butachlor peak ‘A’, (ii) a 1,2-benzene dicarboxylic acid peak ‘B’, and (iii) peak ‘C’ corresponding to 2,6 bis (1,1, dimethylethyl) 4,-methyl phenol with respective peak areas of 50.89%, 33.91% and 5.07%. The percent composition of butachlor and products formed after each reaction is given in Table 2.

**Table 2:** Percent composition of butachlor and metabolites as determined by GC-MS analysis. The GC-MS measurements were carried out with the metabolites extracted from the reaction mix containing 50mM potassium phosphate buffer pH 7.4, 0.4 mM butachlor and 10mM 2-mercaptoethanol, (1) neither NADPH nor AKR, (2) NADPH (0.12mM), (3) AKR (5 μg), or (4) both NADPH (0.12mM) and AKR (5 μg).

Reaction Mix	Butachlor (%)	1,2-benzene dicarboxylic acid (%)	2,6 bis (1,1, dimethylethyl) 4,-methyl phenol (%)
1	98.7	1.294	-
2	97.26	2.73	-
3	95.71	4.28	-
4	56.62	37.73	5.64

These results indicate that reaction 4 containing both enzyme AKR17A1 and cofactor NADPH exhibited 41.08, 40.64 and 38.03% decrease in the amount of butachlor when compared to reaction 1 (containing neither NADPH nor AKR17A1), 2 (only NADPH) and 3 (only AKR17A1), respectively. Whereas, the amount of 1,2-benzene dicarboxylic acid was increased upto 36.44, 35.0 and 32.45% compared to that in reaction 1, 2 and 3, respectively (Table 2). This indicates that AKR17A1 significantly contributes to metabolism/degradation of butachlor.

## Discussion

To increase crop productivity, current agricultural practices are greatly dependent on accelerated pesticide usage. This not only causes environmental contamination through prolonged persistence but adversely affects various non-target organisms. Thus, there is a need to identify some eco-friendly systems to mitigate the ill-effects associated with excessive pesticide application. In this pursuit we identified a protein ‘AKR17A1’ from *Anabaena* 7120 an entirely new family member of AKR superfamily which degrades an extensively used rice field herbicide butachlor.

AKR17A1 primarily possess two novel attributes that validate its position as a new family member: (i) Although AKR17A1 shows close homology with AKR13B1 (Fig 1), its amino acid sequence displayed marked divergence particularly in the regions corresponding to loop B and C (Fig 2). Since, the key loops A, B and C contribute significantly to the substrate binding site [21]; AKR17A1 may not necessarily have similar function as AKR13B1 and therefore, cannot be placed in AKR13 family. (ii) AKR17A1 appears quite distinct from the only studied cyanobacterial AKR, methylglyoxal reductase (AKR11B3) of *Synechococcus* sp. PCC 7002 [27], in terms of sequence identity (sharing 26.5% amino acid identity with AKR11B3) and substrate specificity. For example, unlike AKR11B3 which shows high activity towards methylglyoxal, AKR17A1 depicted high activity towards isatin and o-nitrobenzaldehyde. In fact the k_cat_/K_m_ values for isatin and o-nitrobenzaldehyde were much higher when compared with those of known AKRs such as AKR14A1 [29], AKR11B3 [30],AKR8A1 [52], AKR2E4 [55] and AKR4C8 [56] (Table B in S3 File). This further supports our claim for aldo/keto reductase (AKR17A1) of *Anabaena* 7120 to be a novel AKR protein.

Owing to low sequence identities with functionally characterized AKRs, the specific physiological functions for potential new members have not been established till date. However, many AKRs are known to play important role in stress defence. Abiotic stresses such as heat, heavy metal, UV-B irradiation, salinity, drought etc. enhance the intracellular ROS production which in turn cause lipid peroxidation and production of reactive carbonyl species considered as indirect effect of oxidative stress. AKRs have been known to detoxify not only certain lipid peroxidation derived reactive carbonyls (such as malondialdehyde (MDA) and 4-hydroxynonenal (4-HNE) etc.) but glycolysis-derived toxic intermediates (e.g methylglyoxal) also [26, 27, 29–32]. Therefore, overexpression of many stress inducible AKRs have been found to be effective in conferring tolerance against oxidative stress induced by heavy metal [25], drought [26], heat [27], salt [28], and UV-B [32] in many plant species. Our results showed elevated level of AKR17A1 transcript after exposure of *Anabaena* 7120 cells to different stresses such as cadmium, butachlor, heat, UV-B, arsenic, salt and drought. These results are in tune with the previous studies where several members of AKR superfamily are reported to be appreciably induced under various adverse conditions, such as AKR11B in anaerobic stress [57], AKR2B6 under osmotic, hydrogen peroxide and ionic stress [58], AKR3A1 and AKR3A2 in osmotic stress [58–60], AKR4C5 and AKR4C6 at elevated salt concentrations, and drought and heat-shock treatment [61]. Furthermore, heterologous expression of AKR17A1 in *E*. *coli* showed better growth under abiotic stresses such as heat, UV-B and cadmium. These results, thus, indicate that AKR17A1 probably offers stress tolerance by detoxifying reactive carbonyl species generated through heat, UV-B and cadmium treatments.

Furthermore, an increased transcript and moderately high affinity (low K_m_) and catalytic efficiency (K_cat_/ K_m_) of AKR17A1 for butachlor lead us to hypothesize its role in metabolism of butachlor. This finds support from absorption spectrophotogram revealing AKR17A1 catalyzed NADPH dependent degradation of butachlor in vitro. The GC-MS results further attested AKR17A1 catalyzed butachlor degradation and production of 1,2-benzene dicarboxylic acid and 2,6 bis (1,1, dimethylethyl) 4,-methyl phenol as the major products. The GC-MS results are in agreement with the previous report where benzene dicarboxylic acid and phenol were the degradation products of butachlor during active growth of the cyanobacterium *Nostoc muscorum* [62].

Thus, aldo-keto reductase (AKR17A1) from *Anabaena* 7120 has emerged as a novel enzyme for degradation of an agriculturally relevant herbicide butachlor. The AKR17A1 seems to be a stress-responsive AKR and may offer multiple stress tolerance in vivo. Thus AKR17A1 emerges as a potential candidate for the development of transgenic cyanobacteria worth exploiting as effective biofertilizer in butachlor contaminated rice paddy fields.

## Supporting Information

S1 File The complete phylogenetic tree representing the relationship of ORF All2316 encoding *Anabaena* AKR to all existing members of the AKR superfamily. (EMF)

S2 File The sequence alignment of the entire AKR protein sequences. (PDF)

S3 File Real time quantitative RT PCR analysis results **(Table A).** SDS-PAGE (12%) analysis of recombinant protein in *E*. *coli* BL21 (DE3). **(Fig A).** Immunoblot detection of AKR17A1 protein in *E*. *coli* transformed with recombinant (R) and empty vectors (E) **(Fig B)**. Aldo/keto reductase activity at various concentrations of (a) benzaldehyde, (b) ethyl pyruvate, (c) methyl glyoxal, (d) isatin, (e) o-nitro benzaldehyde and (e) butachlor **(Fig C)**. GC-MS spectra of butachlor and metabolites produced after degradation **(Fig D)**. Absorption spectra of NADPH in reaction mix without butachlor **(Fig E)**. Comparison of AKR171A1 activity for isatin and o-nitrobenzaldehyde with other characterized AKRs **(Table B)**.
(DOCX)
