# Supplementary material for: A Novel Aldo-Keto Reductase (AKR17A1) of Anabaena sp. PCC 7120 Degrades the Rice Field Herbicide Butachlor and Confers Tolerance to Abiotic Stresses in E. coli
Source: PLoS One. 2015 Sep 15;10(9):e0137744. doi: 10.1371/journal.pone.0137744 (PMC4570671; doi:10.1371/journal.pone.0137744)
Supplement: S2 File — (PDF) [file pone.0137744.s002.pdf]

Alignment: C:\Documents and Settings\comp\Desktop\Swati\New Folder (2)\AKR SEQUENCES only.txt
Seaview [blocks=10 fontsize=10 A4] on Sat Apr 11 00:58:10 2015

```
                    1
1A1_gi|5174391|     ---------- ---------- ---------- ---------- ---------- ----------
1A2_gi|1703236|     ---------- ---------- ---------- ---------- ---------- ----------
1A3_gi|1703237|     ---------- ---------- ---------- ---------- ---------- ----------
1A4_gi|10946870|    ---------- ---------- ---------- ---------- ---------- ----------
1B1_gi|4502049|     ---------- ---------- ---------- ---------- ---------- ----------
1B2_gi|1703235|     ---------- ---------- ---------- ---------- ---------- ----------
1B3_gi|1351911|     ---------- ---------- ---------- ---------- ---------- ----------
1B4_gi|6978491|     ---------- ---------- ---------- ---------- ---------- ----------
1B5_gi|113594|      ---------- ---------- ---------- ---------- ---------- ----------
1B6_gi|584742|      ---------- ---------- ---------- ---------- ---------- ----------
1B7_gi|231525|      ---------- ---------- ---------- ---------- ---------- ----------
1B8_gi|6679791|     ---------- ---------- ---------- ---------- ---------- ----------
1B9_gi|2114406|     ---------- ---------- ---------- ---------- ---------- ----------
1B10_gi|223468663|  ---------- ---------- ---------- ---------- ---------- ----------
1B12_gi|14330324|   ---------- ---------- ---------- ---------- ---------- ----------
1B13_gi|15864567|   ---------- ---------- ---------- ---------- ---------- ----------
1B14_gi|148540194|  ---------- ---------- ---------- ---------- ---------- ----------
1B15_gi|51094822|   ---------- ---------- ---------- ---------- ---------- ----------
1C1_gi|5453543|     ---------- ---------- ---------- ---------- ---------- ----------
1C2_gi|4503285|     ---------- ---------- ---------- ---------- ---------- ----------
1C3_gi|24497583|    ---------- ---------- ---------- ---------- ---------- ----------
1C4_gi|308153631|   ---------- ---------- ---------- ---------- ---------- ----------
1C5_gi|1352733|     ---------- ---------- ---------- ---------- ---------- ----------
1C6_gi|13487925|    ---------- ---------- ---------- ---------- ---------- ----------
1C7_gi|129896|      ---------- ---------- ---------- ---------- ---------- ----------
1C8_gi|1709623|     ---------- ---------- ---------- ---------- ---------- ----------
1C9_gi|118634|      ---------- ---------- ---------- ---------- ---------- ----------
1C10a_gi|1345830|   ---------- ---------- ---------- ---------- ---------- ----------
1C10b_gi|1706132|   ---------- ---------- ---------- ---------- ---------- ----------
1C11_gi|1669605|    ---------- ---------- ---------- ---------- ---------- ----------
1C12_gi|85719330|   ---------- ---------- ---------- ---------- ---------- ----------
1C13_gi|171846276|  ---------- ---------- ---------- ---------- ---------- ----------
1C14_gi|19527294|   ---------- ---------- ---------- ---------- ---------- ----------
1C15_gi|741804|     ---------- ---------- ---------- ---------- ---------- ----------
1C16_gi|741803|     ---------- ---------- ---------- ---------- ---------- ----------
1C17_gi|741805|     ---------- ---------- ---------- ---------- ---------- ----------
1C18_gi|1654715|    ---------- ---------- ---------- ---------- ---------- ----------
1C20_gi|16905111|   ---------- ---------- ---------- ---------- ---------- ----------
1C21_gi|126090770|  ---------- ---------- ---------- ---------- ---------- ----------
1C22_gi|38603389|   ---------- ---------- ---------- ---------- ---------- ----------
1C23_gi|62856987|   ---------- ---------- ---------- ---------- ---------- ----------
1C24_gi|84993586|   ---------- ---------- ---------- ---------- ---------- ----------
1C25_gi|15216337|   ---------- ---------- ---------- ---------- ---------- ----------
1D1_gi|5174695|     ---------- ---------- ---------- ---------- ---------- ----------
1D2_gi|398962|      ---------- ---------- ---------- ---------- ---------- ----------
1D3_gi|5689216|     ---------- ---------- ---------- ---------- ---------- ----------
1E1_gi|1698718|     ---------- ---------- ---------- ---------- ---------- ----------
1E2_gi|269849539|   ---------- ---------- ---------- ---------- ---------- ----------
1G1_gi|17550248|    ---------- ---------- ---------- ---------- ---------- ----------
2A1_gi|134153|      ---------- ---------- ---------- ---------- ---------- ----------
2A2_gi|1835701|     ---------- ---------- ---------- ---------- ---------- ----------
2B1_gi|401428|      ---------- ---------- ---------- ---------- ---------- ----------
2B2_gi|1351442|     ---------- ---------- ---------- ---------- ---------- ----------
2B3_gi|2492803|     ---------- ---------- ---------- ---------- ---------- ----------
2B4_gi|1912051|     ---------- ---------- ---------- ---------- ---------- ----------
2B5_gi|3289019|     ---------- ---------- ---------- ---------- ---------- ----------
2B6_gi|6321895|     ---------- ---------- ---------- ---------- ---------- ----------
2B7_gi|1912049|     ---------- ---------- ---------- ---------- ---------- ----------
2B8_gi|4103055|     ---------- ---------- ---------- ---------- ---------- ----------
2C1_gi|1332539|     ---------- ---------- ---------- ---------- ---------- ----------
2D1_gi|7407095|     ---------- ---------- ---------- ---------- ---------- ----------
2E1_gi|4753912|     ---------- ---------- ---------- ---------- ---------- ----------
2E2_gi|18479021|    ---------- ---------- ---------- ---------- ---------- ----------
2E3_gi|5052610|     ---------- ---------- ---------- ---------- ---------- ----------
3A1_gi|121087|      ---------- ---------- ---------- ---------- ---------- ----------
3A2_gi|6320576|     ---------- ---------- ---------- ---------- ---------- ----------
3B1_gi|1142698|     ---------- ---------- ---------- ---------- ---------- ----------
3B2_gi|60458781|    ---------- ---------- ---------- ---------- ---------- ----------
3B3_gi|60458785|    ---------- ---------- ---------- ---------- ---------- ----------
3C1_gi|536474|      ---------- ---------- ---------- ---------- ---------- ----------
3C2_gi|74626610|    ---------- ---------- ---------- ---------- ---------- ----------
3C3_gi|38423524|    ---------- ---------- ---------- ---------- ---------- ----------
3D1_gi|31321885|    ---------- ---------- ---------- ---------- ---------- ----------
3E1_gi|22207641|    ---------- ---------- ---------- ---------- ---------- ----------
3F1_gi|81625481|    ---------- ---------- ---------- ---------- ---------- ----------
```

```
3F2_gi|13638516|      ---------- ---------- ---------- ---------- ---------- ----------
3F3_gi|81635765|      ---------- ---------- ---------- ---------- ---------- ----------
4A1_gi|112837|        ---------- ---------- ---------- ---------- ---------- ----------
4A2_gi|75220959|      ---------- ---------- ---------- ---------- ---------- ----------
4A3_gi|1215788|       ---------- ---------- ---------- ---------- ---------- ----------
4A4_gi|1514979|       ---------- ---------- ---------- ---------- ---------- ----------
4B1_gi|2792155|       ---------- ---------- ---------- ---------- ---------- ----------
4B2_gi|6478210|       ---------- ---------- ---------- ---------- ---------- ----------
4B3_gi|6478204|       ---------- ---------- ---------- ---------- ---------- ----------
4B4_gi|2792295|       ---------- ---------- ---------- ---------- ---------- ----------
4B5_gi|112807104|     ---------- ---------- ---------- ---------- ---------- ----------
4B6_gi|112807098|     ---------- ---------- ---------- ---------- ---------- ----------
4B7_gi|112807100|     ---------- ---------- ---------- ---------- ---------- ----------
4B8_gi|112807102|     ---------- ---------- ---------- ---------- ---------- ----------
4C1_gi|113595|        ---------- ---------- ---------- ---------- ---------- ----------
4C2_gi|167113|        ---------- ---------- ---------- ---------- ---------- ----------
4C3_gi|75221432|      ---------- ---------- ---------- ---------- ---------- ----------
4C4_gi|4539944|       ---------- ---------- ---------- ---------- ---------- ----------
4C5_gi|13160397|      ---------- ---------- ---------- ---------- ---------- ----------
4C6_gi|13160399|      ---------- ---------- ---------- ---------- ---------- ----------
4C8_gi|111182163|     ---------- ---------- ---------- ---------- ---------- ----------
4C9_gi|111182165|     ---------- ---------- ---------- ---------- ---------- ----------
4C10_gi|111182167|    ---------- ---------- ---------- ---------- ---------- ----------
4C11_gi|111182169|    ---------- ---------- ---------- ---------- ---------- ----------
5A1_gi|408360251|     ---------- ---------- ---------- ---------- ---------- ----------
5A2_gi|11127591|      ---------- ---------- ---------- ---------- ---------- ----------
5B1_gi|2506173|       ---------- ---------- ---------- ---------- ---------- ----------
5C1_gi|144969|        ---------- ---------- ---------- ---------- ---------- ----------
5C2_gi|3916039|       ---------- ---------- ---------- ---------- ---------- ----------
5D1_gi|112735|        ---------- ---------- ---------- ---------- ---------- ----------
5E1_gi|5354195|       ---------- ---------- ---------- ---------- ---------- ----------
5F1_gi|82504416|      ---------- ---------- ---------- ---------- ---------- ----------
5G1_gi|16080393|      ---------- ---------- ---------- ---------- ---------- ----------
5G2_gi|16079957|      ---------- ---------- ---------- ---------- ---------- ----------
6A1_gi|18202524|      ---------- ---------- ---------- ---------- ---------- ----------
6A2_gi|499328|        ---------- ---------- ---------- ---------- ---------- ----------
6A3_gi|2135947|       ---------- ---------- ---------- ---------- ---------- ----------
6A4_gi|975314|        ---------- ---------- ---------- ---------- ---------- ----------
6A5_gi|2827466|       ---------- ---------- ---------- ---------- ---------- ----------
6A6_gi|7914984|       ---------- ---------- ---------- ---------- ---------- ----------
6A7_gi|5019764|       ---------- ---------- ---------- ---------- ---------- ----------
6A8_gi|148747467|     ---------- ---------- ---------- ---------- ---------- ----------
6A9_gi|24648619|      ---------- ---------- ---------- ---------- ---------- ----------
6A10a_gi|726465|      ---------- ---------- ---------- ---------- ---------- ----------
6A10b_gi|5019766|     ---------- ---------- ---------- ---------- ---------- ----------
6A11_gi|5922729|      ---------- ---------- ---------- ---------- ---------- ----------
6A13_gi|52001073|     ---------- ---------- ---------- ---------- ---------- ----------
6A14_gi|226823214|    ---------- ---------- ---------- ---------- ---------- ----------
6B1_gi|902000|        MSMALCNLNG DGSAAQSTSQ SQSPAATAAA APLLPHSHSH TLQPESTPLL LGHEQSGSAA
6C1_gi|1063415|       ---------- ---------- ---------- ---------- ---------- ----------
6C2_gi|2832783|       ---------- ---------- ---------- ---------- ---------- ----------
7A1_gi|39932720|      ---------- ---------- ---------- ---------- ---------- ----------
7A2_gi|41327764|      ---------- ---------- ---------- ---------- ---------- ----------
7A3_gi|41152114|      ---------- ---------- ---------- ---------- ---------- ----------
7A4_gi|6815049|       ---------- ---------- ---------- ---------- ---------- ----------
8A1_gi|24638123|      ---------- ---------- ---------- ---------- ---------- ----------
8A2_gi|74627022|      ---------- ---------- ---------- ---------- ---------- ----------
9A1_gi|146345520|     ---------- ---------- ---------- ---------- ---------- ----------
9A2_gi|6093525|       ---------- ---------- ---------- ---------- ---------- ----------
9A3_gi|2492798|       ---------- ---------- ---------- ---------- ---------- ----------
9B1_gi|6323998|       ---------- ---------- ---------- ---------- ---------- ----------
9B2_gi|6319951|       ---------- ---------- ---------- ---------- ---------- ----------
9B3_gi|6319958|       ---------- ---------- ---------- ---------- ---------- ----------
9B4_gi|6322615|       ---------- ---------- ---------- ---------- ---------- ----------
9C1_gi|2459734|       ---------- ---------- ---------- ---------- ---------- ----------
10A1_gi|4731595|      ---------- ---------- ---------- ---------- ---------- ----------
10A2_gi|3256056|      ---------- ---------- ---------- ---------- ---------- ----------
11A1_gi|1176985|      ---------- ---------- ---------- ---------- ---------- ----------
11B1_gi|3123233|      ---------- ---------- ---------- ---------- ---------- ----------
11B2_gi|3123121|      ---------- ---------- ---------- ---------- ---------- ----------
11B3_gi|85062654|     ---------- ---------- ---------- ---------- ---------- ----------
11C1_gi|81787577|     ---------- ---------- ---------- ---------- ---------- ----------
12A1_gi|5305791|      ---------- ---------- ---------- ---------- ---------- ----------
12B1_gi|2599278|      ---------- ---------- ---------- ---------- ---------- ----------
12C1_gi|5921163|      ---------- ---------- ---------- ---------- ---------- ----------
13A1_gi|1351673|      ---------- ---------- ---------- ---------- ---------- ----------
13B1_gi|9106797|      ---------- ---------- ---------- ---------- ---------- ----------
13C1_gi|81555851|     ---------- ---------- ---------- ---------- ---------- ----------
14A1_gi|882530|       ---------- ---------- ---------- ---------- ---------- ----------
```

```
15A1_gi|37196700|   ---------- ---------- ---------- ---------- ---------- ----------
all2316_aldo/keto   ---------- ---------- ---------- ---------- ---------- ----------
```

```
                        61
1A1_gi|5174391|         ---------- ---------- ---------- ---------- ---------- ----------
1A2_gi|1703236|         ---------- ---------- ---------- ---------- ---------- ----------
1A3_gi|1703237|         ---------- ---------- ---------- ---------- ---------- ----------
1A4_gi|10946870|        ---------- ---------- ---------- ---------- ---------- ----------
1B1_gi|4502049|         ---------- ---------- ---------- ---------- ---------- ----------
1B2_gi|1703235|         ---------- ---------- ---------- ---------- ---------- ----------
1B3_gi|1351911|         ---------- ---------- ---------- ---------- ---------- ----------
1B4_gi|6978491|         ---------- ---------- ---------- ---------- ---------- ----------
1B5_gi|113594|          ---------- ---------- ---------- ---------- ---------- ----------
1B6_gi|584742|          ---------- ---------- ---------- ---------- ---------- ----------
1B7_gi|231525|          ---------- ---------- ---------- ---------- ---------- ----------
1B8_gi|6679791|         ---------- ---------- ---------- ---------- ---------- ----------
1B9_gi|2114406|         ---------- ---------- ---------- ---------- ---------- ----------
1B10_gi|223468663|      ---------- ---------- ---------- ---------- ---------- ----------
1B12_gi|14330324|       ---------- ---------- ---------- ---------- ---------- ----------
1B13_gi|15864567|       ---------- ---------- ---------- ---------- ---------- ----------
1B14_gi|148540194|      ---------- ---------- ---------- ---------- ---------- ----------
1B15_gi|51094822|       ---------- ---------- ---------- ---------- ---------- ----------
1C1_gi|5453543|         ---------- ---------- ---------- ---------- ---------- ----------
1C2_gi|4503285|         ---------- ---------- ---------- ---------- ---------- ----------
1C3_gi|24497583|        ---------- ---------- ---------- ---------- ---------- ----------
1C4_gi|308153631|       ---------- ---------- ---------- ---------- ---------- ----------
1C5_gi|1352733|         ---------- ---------- ---------- ---------- ---------- ----------
1C6_gi|13487925|        ---------- ---------- ---------- ---------- ---------- ----------
1C7_gi|129896|          ---------- ---------- ---------- ---------- ---------- ----------
1C8_gi|1709623|         ---------- ---------- ---------- ---------- ---------- ----------
1C9_gi|118634|          ---------- ---------- ---------- ---------- ---------- ----------
1C10a_gi|1345830|       ---------- ---------- ---------- ---------- ---------- ----------
1C10b_gi|1706132|       ---------- ---------- ---------- ---------- ---------- ----------
1C11_gi|1669605|        ---------- ---------- ---------- ---------- ---------- ----------
1C12_gi|85719330|       ---------- ---------- ---------- ---------- ---------- ----------
1C13_gi|171846276|      ---------- ---------- ---------- ---------- ---------- ----------
1C14_gi|19527294|       ---------- ---------- ---------- ---------- ---------- ----------
1C15_gi|741804|         ---------- ---------- ---------- ---------- ---------- ----------
1C16_gi|741803|         ---------- ---------- ---------- ---------- ---------- ----------
1C17_gi|741805|         ---------- ---------- ---------- ---------- ---------- ----------
1C18_gi|1654715|        ---------- ---------- ---------- ---------- ---------- ----------
1C20_gi|16905111|       ---------- ---------- ---------- ---------- ---------- ----------
1C21_gi|126090770|      ---------- ---------- ---------- ---------- ---------- ----------
1C22_gi|38603389|       ---------- ---------- ---------- ---------- ---------- ----------
1C23_gi|62856987|       ---------- ---------- ---------- ---------- ---------- ----------
1C24_gi|84993586|       ---------- ---------- ---------- ---------- ---------- ----------
1C25_gi|15216337|       ---------- ---------- ---------- ---------- ---------- ----------
1D1_gi|5174695|         ---------- ---------- ---------- ---------- ---------- ----------
1D2_gi|398962|          ---------- ---------- ---------- ---------- ---------- ----------
1D3_gi|5689216|         ---------- ---------- ---------- ---------- ---------- ----------
1E1_gi|1698718|         ---------- ---------- ---------- ---------- ---------- ----------
1E2_gi|269849539|       ---------- ---------- ---------- ---------- ---------- ----------
1G1_gi|17550248|        ---------- ---------- ---------- ---------- ---------- ----------
2A1_gi|134153|          ---------- ---------- ---------- ---------- ---------- ----------
2A2_gi|1835701|         ---------- ---------- ---------- ---------- ---------- ----------
2B1_gi|401428|          ---------- ---------- ---------- ---------- ---------- ----------
2B2_gi|1351442|         ---------- ---------- ---------- ---------- ---------- ----------
2B3_gi|2492803|         ---------- ---------- ---------- ---------- ---------- ----------
2B4_gi|1912051|         ---------- ---------- ---------- ---------- ---------- ----------
2B5_gi|3289019|         ---------- ---------- ---------- ---------- ---------- ----------
2B6_gi|6321895|         -----MTSIQ ERGTSAHLHS LKEGEASDRS SEMLPKQRSI IGSHVQRPPS QTTLGRSRAG
2B7_gi|1912049|         ---------- ---------- ---------- ---------- ---------- ----------
2B8_gi|4103055|         ---------- ---------- ---------- ---------- ---------- ----------
2C1_gi|1332539|         ---------- ---------- ---------- ---------- ---------- ----------
2D1_gi|7407095|         ---------- ---------- ---------- ---------- ---------- ----------
2E1_gi|4753912|         ---------- ---------- ---------- ---------- ---------- ----------
2E2_gi|18479021|        ---------- ---------- ---------- ---------- ---------- ----------
2E3_gi|5052610|         ---------- ---------- ---------- ---------- ---------- ----------
3A1_gi|121087|          ---------- ---------- ---------- ---------- ---------- ----------
3A2_gi|6320576|         ---------- ---------- ---------- ---------- ---------- ----------
3B1_gi|1142698|         ---------- ---------- ---------- ---------- ---------- ----------
3B2_gi|60458781|        ---------- ---------- ---------- ---------- ---------- ----------
3B3_gi|60458785|        ---------- ---------- ---------- ---------- ---------- ----------
3C1_gi|536474|          ---------- ---------- ---------- ---------- ---------- ----------
3C2_gi|74626610|        ---------- ---------- ---------- ---------- ---------- ----------
3C3_gi|38423524|        ---------- ---------- ---------- ---------- ---------- ----------
3D1_gi|31321885|        ---------- ---------- ---------- ---------- ---------- ----------
3E1_gi|22207641|        ---------- ---------- ---------- ---------- ---------- ----------
3F1_gi|81625481|        ---------- ---------- ---------- ---------- ---------- ----------
3F2_gi|13638516|        ---------- ---------- ---------- ---------- ---------- ----------
3F3_gi|81635765|        ---------- ---------- ---------- ---------- ---------- ----------
4A1_gi|112837|          ---------- ---------- ---------- ---------- ---------- ----------
```

```
4A2_gi|75220959|     ---------- ---------- ---------- ---------- ---------- ----------
4A3_gi|1215788|      ---------- ---------- ---------- ---------- ---------- ----------
4A4_gi|1514979|      ---------- ---------- ---------- ---------- ---------- ----------
4B1_gi|2792155|      ---------- ---------- ---------- ---------- ---------- ----------
4B2_gi|6478210|      ---------- ---------- ---------- ---------- ---------- ----------
4B3_gi|6478204|      ---------- ---------- ---------- ---------- ---------- ----------
4B4_gi|2792295|      ---------- ---------- ---------- ---------- ---------- ----------
4B5_gi|112807104|    ---------- ---------- ---------- ---------- ---------- ----------
4B6_gi|112807098|    ---------- ---------- ---------- ---------- ---------- ----------
4B7_gi|112807100|    ---------- ---------- ---------- ---------- ---------- ----------
4B8_gi|112807102|    ---------- ---------- ---------- ---------- ---------- ----------
4C1_gi|113595|       ---------- ---------- ---------- ---------- ---------- ----------
4C2_gi|167113|       ---------- ---------- ---------- ---------- ---------- ----------
4C3_gi|75221432|     ---------- ---------- ---------- ---------- ---------- ----------
4C4_gi|4539944|      ---------- ---------- ---------- ---------- ---------- ----------
4C5_gi|13160397|     ---------- ---------- ---------- ---------- ---------- ----------
4C6_gi|13160399|     ---------- ---------- ---------- ---------- ---------- ----------
4C8_gi|111182163|    ---------- ---------- ---------- ---------- ---------- ----------
4C9_gi|111182165|    ---------- ---------- ---------- ---------- ---------- ----------
4C10_gi|111182167|   ---------- ---------- ---------- ---------- ---------- ----------
4C11_gi|111182169|   ---------- ---------- ---------- ---------- ---------- ----------
5A1_gi|408360251|    ---------- ---------- ---------- ---------- ---------- ----------
5A2_gi|11127591|     ---------- ---------- ---------- ---------- ---------- ----------
5B1_gi|2506173|      ---------- ---------- ---------- ---------- ---------- ----------
5C1_gi|144969|       ---------- ---------- ---------- ---------- ---------- ----------
5C2_gi|3916039|      ---------- ---------- ---------- ---------- ---------- ----------
5D1_gi|112735|       ---------- ---------- ---------- ---------- ---------- ----------
5E1_gi|5354195|      ---------- ---------- ---------- ---------- ---------- ----------
5F1_gi|82504416|     ---------- ---------- ---------- ---------- ---------- ----------
5G1_gi|16080393|     ---------- ---------- ---------- ---------- ---------- ----------
5G2_gi|16079957|     ---------- ---------- ---------- ---------- ---------- ----------
6A1_gi|18202524|     ---------- ---------- ---------- ---------- ---------- ----------
6A2_gi|499328|       ---------- ---------- ---------- ---------- ---------- ----------
6A3_gi|2135947|      ---------- ---------- ---------- ---------- ---------- ----------
6A4_gi|975314|       ---------- ---------- ---------- ---------- ---------- ----------
6A5_gi|2827466|      ---------- ---------- ---------- ---------- ---------- ----------
6A6_gi|7914984|      ---------- ---------- ---------- ---------- ---------- ----------
6A7_gi|5019764|      ---------- ---------- ---------- ---------- ---------- ----------
6A8_gi|148747467|    ---------- ---------- ---------- ---------- ---------- ----------
6A9_gi|24648619|     ---------- ---------- -------MQK QEETLSRLRT IFDIFLRENF STTNNVYFEK
6A10a_gi|726465|     ---------- ---------- ---------- ---------- ---------- ----------
6A10b_gi|5019766|    ---------- ---------- ---------- ---------- ---------- ----------
6A11_gi|5922729|     ---------- ---------- ---------- ---------- ---------- ----------
6A13_gi|52001073|    ---------- ---------- ---------- ---------- ---------- ----------
6A14_gi|226823214|   ---------- ---------- ---------- ---------- ---------- ----------
6B1_gi|902000|       PEGSGGGDVA DGAVTSIEMP TVVADDAGVP PLPLPLPQST PQPLLMALPA NLNFITGPTT
6C1_gi|1063415|      ---------- ---------- ---------- ---------- ---------- ----------
6C2_gi|2832783|      ---------- ---------- ---------- ---------- ---------- ----------
7A1_gi|39932720|     ---------- ---------- ---------- ---------- ---------- ----------
7A2_gi|41327764|     ---------- ---------- ---------- ---------- ---------- ----------
7A3_gi|41152114|     ---------- ---------- ---------- ---------- ---------- ----------
7A4_gi|6815049|      ---------- ---------- ---------- ---------- ---------- ----------
8A1_gi|24638123|     ---------- ---------- ---------- ---------- ---------- ----------
8A2_gi|74627022|     ---------- ---------- ---------- ---------- ---------- ----------
9A1_gi|146345520|    ---------- ---------- ---------- ---------- ---------- ----------
9A2_gi|6093525|      ---------- ---------- ---------- ---------- ---------- ----------
9A3_gi|2492798|      ---------- ---------- ---------- ---------- ---------- ----------
9B1_gi|6323998|      ---------- ---------- ---------- ---------- ---------- ----------
9B2_gi|6319951|      ---------- ---------- ---------- ---------- ---------- ----------
9B3_gi|6319958|      ---------- ---------- ---------- ---------- ---------- ----------
9B4_gi|6322615|      ---------- ---------- ---------- ---------- ---------- ----------
9C1_gi|2459734|      ---------- ---------- ---------- ---------- ---------- ----------
10A1_gi|4731595|     ---------- ---------- ---------- ---------- ---------- ----------
10A2_gi|3256056|     ---------- ---------- ---------- ---------- ---------- ----------
11A1_gi|1176985|     ---------- ---------- ---------- ---------- ---------- ----------
11B1_gi|3123233|     ---------- ---------- ---------- ---------- ---------- ----------
11B2_gi|3123121|     ---------- ---------- ---------- ---------- ---------- ----------
11B3_gi|85062654|    ---------- ---------- ---------- ---------- ---------- ----------
11C1_gi|81787577|    ---------- ---------- ---------- ---------- ---------- ----------
12A1_gi|5305791|     ---------- ---------- ---------- ---------- ---------- ----------
12B1_gi|2599278|     ---------- ---------- ---------- ---------- ---------- ----------
12C1_gi|5921163|     ---------- ---------- ---------- ---------- ---------- ----------
13A1_gi|1351673|     ---------- ---------- ---------- ---------- ---------- ----------
13B1_gi|9106797|     ---------- ---------- ---------- ---------- ---------- ----------
13C1_gi|81555851|    ---------- ---------- ---------- ---------- ---------- ----------
14A1_gi|882530|      ---------- ---------- ---------- ---------- ---------- ----------
15A1_gi|37196700|    ---------- ---------- ---------- ---------- ---------- ----------
all2316_aldo/keto    ---------- ---------- ---------- ---------- ---------- ----------
```

```
                        121
1A1_gi|5174391|         ---------- ---------- ---------- ---------- ---------- ----------
1A2_gi|1703236|         ---------- ---------- ---------- ---------- ---------- ----------
1A3_gi|1703237|         ---------- ---------- ---------- ---------- ---------- ----------
1A4_gi|10946870|        ---------- ---------- ---------- ---------- ---------- ----------
1B1_gi|4502049|         ---------- ---------- ---------- ---------- ---------- ----------
1B2_gi|1703235|         ---------- ---------- ---------- ---------- ---------- ----------
1B3_gi|1351911|         ---------- ---------- ---------- ---------- ---------- ----------
1B4_gi|6978491|         ---------- ---------- ---------- ---------- ---------- ----------
1B5_gi|113594|          ---------- ---------- ---------- ---------- ---------- ----------
1B6_gi|584742|          ---------- ---------- ---------- ---------- ---------- ----------
1B7_gi|231525|          ---------- ---------- ---------- ---------- ---------- ----------
1B8_gi|6679791|         ---------- ---------- ---------- ---------- ---------- ----------
1B9_gi|2114406|         ---------- ---------- ---------- ---------- ---------- ----------
1B10_gi|223468663|      ---------- ---------- ---------- ---------- ---------- ----------
1B12_gi|14330324|       ---------- ---------- ---------- ---------- ---------- ----------
1B13_gi|15864567|       ---------- ---------- ---------- ---------- ---------- ----------
1B14_gi|148540194|      ---------- ---------- ---------- ---------- ---------- ----------
1B15_gi|51094822|       ---------- ---------- ---------- ---------- ---------- ----------
1C1_gi|5453543|         ---------- ---------- ---------- ---------- ---------- ----------
1C2_gi|4503285|         ---------- ---------- ---------- ---------- ---------- ----------
1C3_gi|24497583|        ---------- ---------- ---------- ---------- ---------- ----------
1C4_gi|308153631|       ---------- ---------- ---------- ---------- ---------- ----------
1C5_gi|1352733|         ---------- ---------- ---------- ---------- ---------- ----------
1C6_gi|13487925|        ---------- ---------- ---------- ---------- ---------- ----------
1C7_gi|129896|          ---------- ---------- ---------- ---------- ---------- ----------
1C8_gi|1709623|         ---------- ---------- ---------- ---------- ---------- ----------
1C9_gi|118634|          ---------- ---------- ---------- ---------- ---------- ----------
1C10a_gi|1345830|       ---------- ---------- ---------- ---------- ---------- ----------
1C10b_gi|1706132|       ---------- ---------- ---------- ---------- ---------- ----------
1C11_gi|1669605|        ---------- ---------- ---------- ---------- ---------- ----------
1C12_gi|85719330|       ---------- ---------- ---------- ---------- ---------- ----------
1C13_gi|171846276|      ---------- ---------- ---------- ---------- ---------- ----------
1C14_gi|19527294|       ---------- ---------- ---------- ---------- ---------- ----------
1C15_gi|741804|         ---------- ---------- ---------- ---------- ---------- ----------
1C16_gi|741803|         ---------- ---------- ---------- ---------- ---------- ----------
1C17_gi|741805|         ---------- ---------- ---------- ---------- ---------- ----------
1C18_gi|1654715|        ---------- ---------- ------GAGA ATGAATTCCA AAATTCAGAA GATAGAATTA
1C20_gi|16905111|       ---------- ---------- ---------- ---------- ---------- ----------
1C21_gi|126090770|      ---------- ---------- ---------- ---------- ---------- ----------
1C22_gi|38603389|       ---------- ---------- ---------- ---------- ---------- ----------
1C23_gi|62856987|       ---------- ---------- ---------- ---------- ---------- ----------
1C24_gi|84993586|       ---------- ---------- ---------- ---------- ---------- ----------
1C25_gi|15216337|       ---------- ---------- ---------- ---------- ---------- ----------
1D1_gi|5174695|         ---------- ---------- ---------- ---------- ---------- ----------
1D2_gi|398962|          ---------- ---------- ---------- ---------- ---------- ----------
1D3_gi|5689216|         ---------- ---------- ---------- ---------- ---------- ----------
1E1_gi|1698718|         ---------- ---------- ---------- ---------- ---------- ----------
1E2_gi|269849539|       ---------- ---------- ---------- ---------- ---------- ----------
1G1_gi|17550248|        ---------- ---------- ---------- ---------- ---------- ----------
2A1_gi|134153|          ---------- ---------- ---------- ---------- ---------- ----------
2A2_gi|1835701|         ---------- ---------- ---------- ---------- ---------- ----------
2B1_gi|401428|          ---------- ---------- ---------- ---------- ---------- ----------
2B2_gi|1351442|         ---------- ---------- ---------- ---------- ---------- ----------
2B3_gi|2492803|         ---------- ---------- ---------- ---------- ---------- ----------
2B4_gi|1912051|         ---------- ---------- ---------- ---------- ---------- ----------
2B5_gi|3289019|         ---------- ---------- ---------- ---------- ---------- ----------
2B6_gi|6321895|         SNTMNKVSGL DIARRPSENL LSNMNCSDNG NGGNMLNSFV NSALPPPKVN PAQTRRERPA
2B7_gi|1912049|         ---------- ---------- ---------- ---------- ---------- ----------
2B8_gi|4103055|         ---------- ---------- ---------- ---------- ---------- ----------
2C1_gi|1332539|         ---------- ---------- ---------- ---------- ---------- ----------
2D1_gi|7407095|         ---------- ---------- ---------- ---------- ---------- ----------
2E1_gi|4753912|         ---------- ---------- ---------- ---------- ---------- ----------
2E2_gi|18479021|        ---------- ---------- ---------- ---------- ---------- --------MK
2E3_gi|5052610|         ---------- ---------- ---------- ---------- ---------- ----------
3A1_gi|121087|          ---------- ---------- ---------- ---------- ---------- ----------
3A2_gi|6320576|         ---------- ---------- ---------- ---------- ---------- ----------
3B1_gi|1142698|         ---------- ---------- ---------- ---------- ---------- ----------
3B2_gi|60458781|        ---------- ---------- ---------- ---------- ---------- ----------
3B3_gi|60458785|        ---------- ---------- ---------- ---------- ---------- ----------
3C1_gi|536474|          ---------- ---------- ---------- ---------- ---------- ----------
3C2_gi|74626610|        ---------- ---------- ---------- ---------- ---------- ----------
3C3_gi|38423524|        ---------- ---------- ---------- ---------- ---------- ----------
3D1_gi|31321885|        ---------- ---------- ---------- ---------- ---------- ----------
3E1_gi|22207641|        ---------- ---------- ---------- ---------- ---------- ----------
3F1_gi|81625481|        ---------- ---------- ---------- ---------- ---------- ----------
3F2_gi|13638516|        ---------- ---------- ---------- ---------- ---------- ----------
3F3_gi|81635765|        ---------- ---------- ---------- ---------- ---------- ----------
4A1_gi|112837|          ---------- ---------- ---------- ---------- ---------- ----------
```

```
4A2_gi|75220959|     ---------- ---------- ---------- ---------- ---------- ----------
4A3_gi|1215788|      ---------- ---------- ---------- ---------- ---------- ----------
4A4_gi|1514979|      ---------- ---------- ---------- ---------- ---------- ----------
4B1_gi|2792155|      ---------- ---------- ---------- ---------- ---------- ----------
4B2_gi|6478210|      ---------- ---------- ---------- ---------- ---------- ----------
4B3_gi|6478204|      ---------- ---------- ---------- ---------- ---------- ----------
4B4_gi|2792295|      ---------- ---------- ---------- ---------- ---------- ----------
4B5_gi|112807104|    ---------- ---------- ---------- ---------- ---------- ----------
4B6_gi|112807098|    ---------- ---------- ---------- ---------- ---------- ----------
4B7_gi|112807100|    ---------- ---------- ---------- ---------- ---------- ----------
4B8_gi|112807102|    ---------- ---------- ---------- ---------- ---------- ----------
4C1_gi|113595|       ---------- ---------- ---------- ---------- ---------- ----------
4C2_gi|167113|       ---------- ---------- ---------- ---------- ---------- ----------
4C3_gi|75221432|     ---------- ---------- ---------- ---------- ---------- ----------
4C4_gi|4539944|      ---------- ---------- ---------- ---------- ---------- ----------
4C5_gi|13160397|     ---------- ---------- ---------- ---------- ---------- ----------
4C6_gi|13160399|     ---------- ---------- ---------- ---------- ---------- ----------
4C8_gi|111182163|    ---------- ---------- ---------- ---------- ---------- ----------
4C9_gi|111182165|    ---------- ---------- ---------- ---------- ---------- ----------
4C10_gi|111182167|   ---------- ---------- ---------- ---------- ---------- ----------
4C11_gi|111182169|   ---------- ---------- ---------- ---------- ---------- ----------
5A1_gi|408360251|    ---------- ---------- ---------- ---------- ---------- ----------
5A2_gi|11127591|     ---------- ---------- ---------- ---------- ---------- ----------
5B1_gi|2506173|      ---------- ---------- ---------- ---------- ---------- ----------
5C1_gi|144969|       ---------- ---------- ---------- ---------- ---------- ----------
5C2_gi|3916039|      ---------- ---------- ---------- ---------- ---------- ----------
5D1_gi|112735|       ---------- ---------- ---------- ---------- ---------- ----------
5E1_gi|5354195|      ---------- ---------- ---------- ---------- ---------- ----------
5F1_gi|82504416|     ---------- ---------- ---------- ---------- ---------- ----------
5G1_gi|16080393|     ---------- ---------- ---------- ---------- ---------- ----------
5G2_gi|16079957|     ---------- ---------- ---------- ---------- ---------- ----------
6A1_gi|18202524|     ---------- ---------- ---------- ---------- -----MYPES TTGSPARLSL
6A2_gi|499328|       ---------- ---------- ---------- ---------- -----MYPES TTGSPARLSL
6A3_gi|2135947|      ---------- -MQVSIACTE HNLKSRNGED RLLSKQSSTA PNVVNAARAK FRTVAIIARS
6A4_gi|975314|       ---------- ---------- ---------- ---------- -----MYPES TTGSPARLSL
6A5_gi|2827466|      ---------- ---------- ---------- ---------- ---------- ---------M
6A6_gi|7914984|      ---------- ---------- ---------- ---------- ---------- ---------M
6A7_gi|5019764|      ---------- -MQVSIACTE HNLKSRNGED RLLSKQSSNA PNVVNAARAK FRTVAIIARS
6A8_gi|148747467|    ---------- -MQVSIACTE HNLKSRNGED RLLSKQSSNA PNVVNAARAK FRTVAIIARS
6A9_gi|24648619|     LLTHLQAKEN AFVLQAPFVV EWVDRCITAV TEDFKKIHPK VISFMLNLTS FLANNEWMIV
6A10a_gi|726465|     ----MHLYKP ACADIPSPKL GLPKSSESAL KCRRHLAVTK PPPQAACWPA RPSGAAERKF
6A10b_gi|5019766|    ----MHLYKP ACADIPSPKL GLPKSSESAL KCRRHLMVTK TQPQAACWPV RPSGPTERKH
6A11_gi|5922729|     ---------- -MQVSFACTE QTLRSRTSED RLCPSRPSGG QNGVSMAQTK QRTPPMGAKN
6A13_gi|52001073|    ---------- -MQVSIACTE HNLKSRNGED RLLSKQSSTA PNVVNAARAK FRTVAIIARS
6A14_gi|226823214|   ----MQVSIA CTEQNLRSRS SEDRLCGPRP GPGGGNGGPV GGGHGNPPGG GGPSSKSRAA
6B1_gi|902000|       QQLMIGNGAM GVIPSTNDSN NNNNNNNNVD NTSDESNPVT IYRCRAPIAS LDCMEEFSGR
6C1_gi|1063415|      ---------- ---------- ---------- ---------- ---------- ----------
6C2_gi|2832783|      ---------- ---------- ---------- ---------- ---------- ----------
7A1_gi|39932720|     ---------- ---------- ---------- ---------- ---------- ----------
7A2_gi|41327764|     ---------- ---------- ---------- ---------- ---------- ---------M
7A3_gi|41152114|     ---------- ---------- ---------- ---------- ---------- ----------
7A4_gi|6815049|      ---------- ---------- ---------- ---------- ---------- ----------
8A1_gi|24638123|     ---------- ---------- ---------- ---------- ---------- ----------
8A2_gi|74627022|     ---------- ---------- ---------- ---------- ---------- ----------
9A1_gi|146345520|    ---------- ---------- ---------- ---------- ---------- ----------
9A2_gi|6093525|      ---------- ---------- ---------- ---------- ---------- ----------
9A3_gi|2492798|      ---------- ---------- ---------- ---------- ---------- ----------
9B1_gi|6323998|      ---------- ---------- ---------- ---------- ---------- ----------
9B2_gi|6319951|      ---------- ---------- ---------- ---------- ---------- ----------
9B3_gi|6319958|      ---------- ---------- ---------- ---------- ---------- ----------
9B4_gi|6322615|      ---------- ---------- ---------- ---------- ---------- ----------
9C1_gi|2459734|      ---------- ---------- ---------- ---------- ---------- ----------
10A1_gi|4731595|     ---------- ---------- ---------- ---------- ---------- ----------
10A2_gi|3256056|     ---------- ---------- ---------- ---------- ---------- ----------
11A1_gi|1176985|     ---------- ---------- ---------- ---------- ---------- ----------
11B1_gi|3123233|     ---------- ---------- ---------- ---------- ---------- ----------
11B2_gi|3123121|     ---------- ---------- ---------- ---------- ---------- ----------
11B3_gi|85062654|    ---------- ---------- ---------- ---------- ---------- ----------
11C1_gi|81787577|    ---------- ---------- ---------- ---------- ---------- ----------
12A1_gi|5305791|     ---------- ---------- ---------- ---------- ---------- ----------
12B1_gi|2599278|     ---------- ---------- ---------- ---------- ---------- ----------
12C1_gi|5921163|     ---------- ---------- ---------- ---------- ---------- ----------
13A1_gi|1351673|     ---------- ---------- ---------- ---------- ---------- ----------
13B1_gi|9106797|     ---------- ---------- ---------- ---------- ---------- ----------
13C1_gi|81555851|    ---------- ---------- ---------- ---------- ---------- ----------
14A1_gi|882530|      ---------- ---------- ---------- ---------- ---------- ----------
15A1_gi|37196700|    ---------- ---------- ---------- ---------- ---------- ----------
all2316_aldo/keto    ---------- ---------- ---------- ---------- ---------- ----------
```

```
                           181
1A1_gi|5174391|      ---------- ---------M AASCVLL--- H-TGQ----K -MPLI----- ---------G
1A2_gi|1703236|      ---------- ---------M AASCVLL--- H-TGQ----K -MPLI----- ---------G
1A3_gi|1703237|      ---------- ---------M TASSVLL--- H-TGQ----K -MPLI----- ---------G
1A4_gi|10946870|     ---------- ---------M TASSVLL--- H-TGQ----K -MPLI----- ---------G
1B1_gi|4502049|      ---------- ---------- MASRLLL--- N-NGA----K -MPIL----- ---------G
1B2_gi|1703235|      ---------- ---------- MATHLVL--- Y-NGA----K -MPIL----- ---------G
1B3_gi|1351911|      ---------- ---------- MASHLEL--- N-NGT----K -MPTL----- ---------G
1B4_gi|6978491|      ---------- ---------- MASHLEL--- N-NGT----K -MPTL----- ---------G
1B5_gi|113594|       ---------- ---------- -AHNIVL--- Y-TGA----K -MPIL----- ---------G
1B6_gi|584742|       ---------- ---------- MASHLVL--- Y-TGA----K -MPIL----- ---------G
1B7_gi|231525|       ---------- ---------- MATFVEL--- S-TKA----K -MPLV----- ---------G
1B8_gi|6679791|      ---------- ---------- MATFVEL--- S-TKA----K -MPIV----- ---------G
1B9_gi|2114406|      ---------- ---------- MSTFVEL--- S-TKA----K -MPIV----- ---------G
1B10_gi|223468663|   ---------- ---------- MATFVEL--- S-TKA----K -MPIV----- ---------G
1B12_gi|14330324|    ---------- ---------M AVPCMEL--- N-NKM----K -MPVL----- ---------G
1B13_gi|15864567|    ---------- ---------- MATFVEL--- S-TKA----K -MPIV----- ---------G
1B14_gi|148540194|   ---------- ---------- MTTFVKL--- R-TKA----K -MPLV----- ---------G
1B15_gi|51094822|    ---------- ---------- MATFVEL--- S-TKA----K -MPIV----- ---------G
1C1_gi|5453543|      ---------- -------MDS KYQCVKL--- N-DGH----F -MPVL----- ---------G
1C2_gi|4503285|      ---------- -------MDS KYQCVKL--- N-DGH----F -MPVL----- ---------G
1C3_gi|24497583|     ---------- -------MDS KHQCVKL--- N-DGH----F -MPVL----- ---------G
1C4_gi|308153631|    ---------- -------MDP KYQRVEL--- N-DGH----F -MPVL----- ---------G
1C5_gi|1352733|      ---------- -------MDP KFQRVAL--- S-DGH----F -IPVL----- ---------G
1C6_gi|13487925|     ---------- -------MDS KQQTVRL--- S-DGH----F -IPIL----- ---------G
1C7_gi|129896|       ---------- -------MDP KSQRVKL--- N-DGH----F -IPVL----- ---------G
1C8_gi|1709623|      ---------- -------MNS KIQKMEL--- N-DGH----S -IPVL----- ---------G
1C9_gi|118634|       ---------- -------MDS ISLRVAL--- N-DGN----F -IPVL----- ---------G
1C10a_gi|1345830|    ---------- ------MTLT KETRVTL--- N-DGN----M -MPIL----- ---------G
1C10b_gi|1706132|    ---------- ------MTLT KETRVTL--- N-DGN----M -MPIL----- ---------G
1C11_gi|1669605|     ---------- -------MDP KGQRVKL--- N-DGH----F -IPVL----- ---------G
1C12_gi|85719330|    ---------- -------MSS KQHYVKL--- N-DGH----L -IPAL----- ---------G
1C13_gi|171846276|   ---------- -------MSS KQHCVKL--- N-DGH----L -IPAL----- ---------G
1C14_gi|19527294|    ---------- -------MNS VSPRVVL--- N-DGH----F -IPAL----- ---------G
1C15_gi|741804|      ---------- -------MDL KHRSVKL--- N-DPH----L -MPVL----- ---------G
1C16_gi|741803|      ---------- ---------- --HCVKL--- N-HGH----F -IPAL----- ---------G
1C17_gi|741805|      ---------- --------MS KHHCVKL--- N-DGH----F -IPAL----- ---------G
1C18_gi|1654715|     AATGATGGTC ACTCCATTCC TGTCCTGGGC TTTGGCACCT ATGCA----- ---ACTGAAG
1C20_gi|16905111|    ---------- -------MNS KQQTVLL--- N-DGH----F -IPIL----- ---------G
1C21_gi|126090770|   ---------- -------MNS KCHCVIL--- N-DGN----F -IPVL----- ---------G
1C22_gi|38603389|    ---------- -------MSS KQHCVKL--- N-DGH----L -IPAL----- ---------G
1C23_gi|62856987|    ---------- -------MDP KGWRVEL--- N-DGH----F -IHAL----- ---------G
1C24_gi|84993586|    ---------- -------MSS KLHCVKL--- N-DGH----F -IPAL----- ---------G
1C25_gi|15216337|    ---------- -------MDP KYQRVAL--- N-DGH----F -MPVL----- ---------G
1D1_gi|5174695|      ---------- -----MDLSA ASHRIPL--- S-DGN----S -IPII----- ---------G
1D2_gi|398962|       ---------- -----MNLST ANHHIPL--- N-DGN----S -IPII----- ---------G
1D3_gi|5689216|      ---------- -----MDLSA TNHRIPL--- G-DGN----S -IPII----- ---------G
1E1_gi|1698718|      ---------- ---------- ---------- --MEN----- -IPTV----- ---------G
1E2_gi|269849539|    ---------- ---------- ---------- --MGD----- -IPAV----- ---------G
1G1_gi|17550248|     ---------- -------MSS ATASIKL--- S-NGV----E -MPVI----- ---------G
2A1_gi|134153|       ---------- ---------- -MSTVTL--- S-SGY----E -MPVI----- ---------G
2A2_gi|1835701|      ---------- ---------- --MAITL--- N-SGF----K -MPVL----- ---------G
2B1_gi|401428|       ---------- ---------- -MPSIKL--- N-SGY----D -MPAV----- ---------G
2B2_gi|1351442|      ---------- -------MTY LAETVTL--- N-NGE----K -MPLV----- ---------G
2B3_gi|2492803|      ---------- ---------M TLQYYTL--- N-NGR----K -IPAI----- ---------G
2B4_gi|1912051|      ---------- -----MSTTP TIPTIKL--- N-SGY----E -MPLV----- ---------G
2B5_gi|3289019|      ---------- -------MSA SIPDIKL--- S-SGH----L -MPSI----- ---------G
2B6_gi|6321895|      SNSSIGTKTT EVFSSTSASS SLGDTSDEGE GSDADKSKIN TFPSILMEKA TQGRGADGNG
2B7_gi|1912049|      ---------- -----MSTTP TIPTIKL--- N-SGY----E -MPLV----- ---------G
2B8_gi|4103055|      ---------- ---------- --MSIKL--- N-SGY----D -MPSV----- ---------G
2C1_gi|1332539|      ---------- ---------M STDYLTL--- NRTGD----K -MPIR----- ---------G
2D1_gi|7407095|      ---------- ---------M ASPTVKL--- N-SGY----D -MPLV----- ---------G
2E1_gi|4753912|      MFRASFILLL ACCGAMSATI DVPMLKM--- L-NDR----E -MPAI----- ---------A
2E2_gi|18479021|     LYLLPVALWA LVNAISGEPG KAPLKQL--- N-DGN----A -IPSL----- ---------A
2E3_gi|5052610|      ---------- -------MSS KIPYVKH--- N-NGT----Q -IQSI----- ---------G
3A1_gi|121087|       ---------- ---MPATLHD STKILSL--- N-TGA----Q -IPQI----- ---------G
3A2_gi|6320576|      ---------- ---MPATLKN SSATLKL--- N-TGA----S -IPVL----- ---------G
3B1_gi|1142698|      ---------- ---------- MVGTTTL--- N-TGA----S -LELV----- ---------G
3B2_gi|60458781|     ---------- --------MS YNKNIPL--- N-DGN----S -IPAL----- ---------G
3B3_gi|60458785|     ---------- --------MS YKQYIPL--- N-DGN----K -IPAL----- ---------G
3C1_gi|536474|       MSSSVASTEN IVENMLHPKT TEIYFSL--- N-NGV----R -IPAL----- ---------G
3C2_gi|74626610|     ---------- ---------- -MLIAAM--- G-PKI----P -VPAY----- ---------G
3C3_gi|38423524|     ---------- ----MTQSNL LPKTFRT--- K-SGK----E ISIAL----- ---------G
3D1_gi|31321885|     ---------- ---------M ADKDFTL--- N-TGA----K -IPAF----- ---------G
3E1_gi|22207641|     ---------- --------MS NGKTFTL--- S-NGV----K -IPGV----- ---------G
3F1_gi|81625481|     ---------- ---------- -MLYKEL--- GRTGE----E -IPAL----- ---------G
3F2_gi|13638516|     ---------- ---------- ---------- --MA------ -IPAF----- ---------G
3F3_gi|81635765|     ---------- --------MG GPMSVYF--- E-KSY----Q ----R----- ---------G
4A1_gi|112837|       ---------- -------MAA AIEIPTIVFP NSSAQ----Q RMPVV----- ---------G
```

```
4A2_gi|75220959|      ---------- ------MGSV EIPTKVLTNT S-SQL----K -MPVV----- ---------G
4A3_gi|1215788|       ---------- ------MAAA AAAAIEIPTK VLPNSTCELR -VPVI----- ---------G
4A4_gi|1514979|       ---------- ----MAAAAI EIPTKVLPNS T-CEL----R -VPVI----- ---------G
4B1_gi|2792155|       ---------- ------MAEK KIPEVLL--- N-SGH----K -MPVI----- ---------G
4B2_gi|6478210|       ---------- ------MESN GVPMITL--- S-SGI----R -MPAL----- ---------G
4B3_gi|6478204|       ---------- ------MESN GVPMITL--- S-SGI----R -MPAL----- ---------G
4B4_gi|2792295|       ---------- --------MA KVPSVTLSSC G-DDI----Q TMPVI----- ---------G
4B5_gi|112807104|     ---------- -----MSATG RAPC------ G--------- -LPRV----- ---------G
4B6_gi|112807098|     ---------- -------MSD GGAGAKG--- --AGF----G -MPRV----- ---------G
4B7_gi|112807100|     ---------- ---------M GAGDRTV--- --AG------ -MPRI----- ---------G
4B8_gi|112807102|     ---------- --------MG AGDRTAA--- G--------- -MPRI----- ---------G
4C1_gi|113595|        ---------M ASAKATMGQG EQDHFVL--- K-SGH----A -MPAV----- ---------G
4C2_gi|167113|        ---------M ASAKAMMGQE RQDHFVL--- K-SGH----A -IPAV----- ---------G
4C3_gi|75221432|      ---------- MASAKAMGQG EQDRFVL--- K-SGH----A -IPAV----- ---------G
4C4_gi|4539944|       ---------M AHAPCFADAK TQSFKLL--- --SGH----S -IPAV----- ---------G
4C5_gi|13160397|      ---------- -------MAE EIRFFKL--- N-TGA----K -IPSV----- ---------G
4C6_gi|13160399|      ---------- -------MAE EIRFFEL--- N-TGA----K -IPSV----- ---------G
4C8_gi|111182163|     ---------- -------MAA PIRFFEL--- N-TGA----K -LPCV----- ---------G
4C9_gi|111182165|     ---------- -------MAN AITFFKL--- N-TGA----K -FPSV----- ---------G
4C10_gi|111182167|    ---------- -------MAE EIRFFEL--- N-TGA----K -IPSV----- ---------G
4C11_gi|111182169|    ---------- -------MAD EIGFFQL--- N-TGA----K -IPSV----- ---------G
5A1_gi|408360251|     ---------- ------MAGV DKAMVTL--- S-NGV----K -MPQF----- ---------G
5A2_gi|11127591|      ---------- --------MA LTQSLKL--- S-NGV----M -MPVL----- ---------G
5B1_gi|2506173|       ---------- -------MAG KSPLINL--- N-NGV----K -MPAL----- ---------G
5C1_gi|144969|        ---------- ---------M TVPSIVL--- N-DGN----S -IPQL----- ---------G
5C2_gi|3916039|       ---------- --------MA NPTVIKL--- Q-DGN----V -MPQL----- ---------G
5D1_gi|112735|        ---------- --------MP NIPTISL--- N-DGR----P -FAEP----- ---------G
5E1_gi|5354195|       ---------- -----MMSPI SIPSIRL--- N-DGN----D -LPAV----- ---------G
5F1_gi|82504416|      ---------- ---------- -MQTVKL--- N-NGI----E -MPLL----- ---------G
5G1_gi|16080393|      ---------- ------MPTS LKDTVKL--- H-NGV----E -MPWF----- ---------G
5G2_gi|16079957|      ---------- ------MTTH LQAKATL--- H-NGV----E -MPWF----- ---------G
6A1_gi|18202524|      RQTGSPGMIY STRYGSPKRQ LQFYRNL--- GKSGL----R -VSCL----- ---------G
6A2_gi|499328|        RQTGSPGMIY STRYGSPKRQ LQFYRNL--- GKSGL----R -VSCL----- ---------G
6A3_gi|2135947|       LGTFTPQHHI SLKESTAKQT GMKYRNL--- GKSGL----R -VSCL----- ---------G
6A4_gi|975314|        RQTGSPGMIY STRYGSPKRQ LQFYRNL--- GKSGL----R -VSCL----- ---------G
6A5_gi|2827466|       YPESTTGSPA RLSLRQTGSP GMIYRNL--- GKSGL----R -VSCL----- ---------G
6A6_gi|7914984|       YPESTTGSPA RLSLRQTGSP GMIYRNL--- GKSGL----R -VSCL----- ---------G
6A7_gi|5019764|       LGTFTPQHHI SLKESTAKQT GMKYRNL--- GKSGL----R -VSCL----- ---------G
6A8_gi|148747467|     LGTFTPQHHI SLKESTAKQT GMKYRNL--- GKSGL----R -VSCL----- ---------G
6A9_gi|24648619|      RLRELDIVNR AVKLLQREHN LSPSIKL--- --GGI----R LMKAI----- ------TVYS
6A10a_gi|726465|      LEKFLRVHGI SLQETTRAET GMAYRNL--- GKSGL----R -VSCL----- ---------G
6A10b_gi|5019766|     LERFLCVHGV SLQETTKAET GMAYRNL--- GKSGL----R -VSCL----- ---------G
6A11_gi|5922729|      HNQVLPLLSH ILRESTVKST GMKYRNL--- GKSGL----R -VSCL----- ---------G
6A13_gi|52001073|     LGTFTPQHHI SLKESTAKQT GMKYRNL--- GKSGL----R -VSCL----- ---------G
6A14_gi|226823214|    VVPRPPAPAG ALRESTGRGT GMKYRNL--- GKSGL----R -VSCL----- ---------G
6B1_gi|902000|        SISLGSNPAL PLRHGSTPTP GLRYKNL--- GKSGL----R -ISNV----- ---------G
6C1_gi|1063415|       ---------- ---------- -MQYKNL--- GKSGL----K -VSTL----- ---------S
6C2_gi|2832783|       ---------- ---------- -MQYKNL--- GRSGL----R -VSQL----- ---------S
7A1_gi|39932720|      ---------- --------MS QARPATV--- ---------- -LGAM----- ---------E
7A2_gi|41327764|      LSAASRVVSR AAVHCALRSP PPEARALAMS RPPPP----R -VASV----- ----------
7A3_gi|41152114|      ---------- ----MSRQLS RARPATV--- ---------- -LGAM----- ---------E
7A4_gi|6815049|       ---------- -------MSR SPAPRAV--- --SGA----P LRPGT----- ---------V
8A1_gi|24638123|      ---------- ---------- ---MPIV--- --SGF----K -VGPI----- ---------G
8A2_gi|74627022|      ---------- ---------- ---MPIV--- --NGF----K -VGPI----- ---------G
9A1_gi|146345520|     -------MAA PPAPQPPSLL GYHRVLS--- PLAGI----R -VSPL----- ---------C
9A2_gi|6093525|       ---------M VLPTAPEPPT LLGYHRILSS S-AGV----R -VSPL----- ---------C
9A3_gi|2492798|       -------MNI WAPAPEPPTK LGRHRQL-AP G-CGL----H -VSPI----- ---------Q
9B1_gi|6323998|       -------MTD LFKPLPEPPT ELGRLRVLSK T-AGI----R VSPLI----- -----LGGAS
9B2_gi|6319951|       ---------- MIGSASDSSS KLGRLRFLSE T-AAI----K VSPLI----- ----------
9B3_gi|6319958|       ---------- ---------- ---------- --MGS----- -MNKE----- ----------
9B4_gi|6322615|       ---------- ---------- ---------- --MAS----R ---------- ----------
9C1_gi|2459734|       ---------- ---MTTLEDI DLDFVPF--- GQTGL----Q -TSEL----- ---------Q
10A1_gi|4731595|      ---------- ------MSTA SHPAAEAASP T-TGN----Q -PPVL----- ----------
10A2_gi|3256056|      ---------- ---MSSVPLP VSSDASS--- QPDGA----- -PPLL----- ----------
11A1_gi|1176985|      ---------- ---------- -MKKAKL--- GKSDL----Q -VFPI----- ---------G
11B1_gi|3123233|      ---------- ---------- -MEYTSI--- ADTGI----E -ASRI----- ---------G
11B2_gi|3123121|      ---------- ---------- -MKKIPL--- GTTDI----T -LSRM----- ---------G
11B3_gi|85062654|     ---------- ---MTRQKNE LMKTRQL--- GQSAV----Q ITPII----- ----------
11C1_gi|81787577|     ---------- ---------- -MKKRQL--- GTSDL----H -VSEL----- ---------G
12A1_gi|5305791|      ---------- ---------M SGMYVQL--- G-RGA----T LVSRL----- ---------W
12B1_gi|2599278|      ---------- ------MTTD AATHVRL--- GRSAL----L -TSRL----- ---------W
12C1_gi|5921163|      --MMPTTAEP APVQSDSNSS APLHTEL--- GRTRL----R -ISRL----- ---------A
13A1_gi|1351673|      ---------- ---------M SIPTRKI--- G-NDT----- -VPAI----- ---------G
13B1_gi|9106797|      ---------- ----MKLDAS LSGQFAI--- --GGD----L TVNRL----- ---------G
13C1_gi|81555851|     ---------- ---------- -MQQRHL--- --GPL----K -VGAL----- ---------A
14A1_gi|882530|       ---------- --MVWLANPE RYGQMQYRYC GKSGL----R -LPAL----- ---------S
15A1_gi|37196700|     ---------- ------MHLK ASEKRAL--- GRTGL----T -VTAL----- ---------G
all2316_aldo/keto     ---------- ---------- -METTQL--- GKTGV----F -VSAI----- ---------G
```

```
                          241
1A1_gi|5174391|           LGT------- -WKS----EP -----G---- QVKA--AV-- ---------- ----------
1A2_gi|1703236|           LGT------- -WKS----EP -----G---- QVKA--AI-- ---------- ----------
1A3_gi|1703237|           LGT------- -WKS----EP -----G---- QVKA--AI-- ---------- ----------
1A4_gi|10946870|          LGT------- -WKS----EP -----G---- QVKA--AI-- ---------- ----------
1B1_gi|4502049|           LGT------- -WKS----PP -----G---- QVTE--AV-- ---------- ----------
1B2_gi|1703235|           LGT------- -WKS----PP -----G---- QVTE--AV-- ---------- ----------
1B3_gi|1351911|           LGT------- -WKS----PP -----G---- QVTE--AV-- ---------- ----------
1B4_gi|6978491|           LGT------- -WKS----PP -----G---- QVTE--AV-- ---------- ----------
1B5_gi|113594|            LGT------- -WKS----PP -----G---- KVTE--AV-- ---------- ----------
1B6_gi|584742|            LGT------- -WKS----PP -----G---- KVTE--AV-- ---------- ----------
1B7_gi|231525|            LGT------- -WKS----SP -----G---- QVKE--AV-- ---------- ----------
1B8_gi|6679791|           LGT------- -WKS----PP -----N---- QVKE--AV-- ---------- ----------
1B9_gi|2114406|           LGT------- -WQS----PP -----G---- QVKE--AV-- ---------- ----------
1B10_gi|223468663|        LGT------- -W-K----SP ---L-G---- KVKE--AV-- ---------- ----------
1B12_gi|14330324|         LGT------- -WQA----PP -----G---- KVEE--VV-- ---------- ----------
1B13_gi|15864567|         LGT------- -WKS----MP -----N---- QVKE--AV-- ---------- ----------
1B14_gi|148540194|        LGT------- -WKS----PP -----G---- QVKE--AV-- ---------- ----------
1B15_gi|51094822|         LGT------- -W-R----SL ---L-G---- KVKE--AV-- ---------- ----------
1C1_gi|5453543|           FGT------- -Y-A-PAEVP ---K-S---- KALE--AT-- ---------- ----------
1C2_gi|4503285|           FGT------- -Y-A-PAEVP ---K-S---- KALE--AV-- ---------- ----------
1C3_gi|24497583|          FGT------- -Y-A-PPEVP ---R-S---- KALE--VT-- ---------- ----------
1C4_gi|308153631|         FGT------- -Y-A-PPEVP ---R-N---- RAVE--VT-- ---------- ----------
1C5_gi|1352733|           FGT------- -Y-A-PEEVP ---K-S---- KAME--AT-- ---------- ----------
1C6_gi|13487925|          FGT------- -Y-A-PQEVP ---K-S---- KATE--AT-- ---------- ----------
1C7_gi|129896|            FGT------- -Y-A-PEEVP ---K-S---- EALE--AT-- ---------- ----------
1C8_gi|1709623|           FGT------- -Y-A-TEENL ---R-K---- KSME--ST-- ---------- ----------
1C9_gi|118634|            FGT------- -T-V-PEKVA ---K-D---- EVIK--AT-- ---------- ----------
1C10a_gi|1345830|         LGT------- -Y-A-SPHVP ---K-S---- LAEE--AV-- ---------- ----------
1C10b_gi|1706132|         LGT------- -Y-A-APDVP ---K-S---- LAEE--AV-- ---------- ----------
1C11_gi|1669605|          FGT------- -F-A-PREVP ---K-S---- EALE--VT-- ---------- ----------
1C12_gi|85719330|         FGT------- -Y-K-PKEVP ---K-S---- KSLE--AA-- ---------- ----------
1C13_gi|171846276|        FGT------- -Y-K-PKEVP ---K-S---- KSLE--AA-- ---------- ----------
1C14_gi|19527294|         FGT------- ---TVPDKVP ---K-D---- ELIK--AT-- ---------- ----------
1C15_gi|741804|           FGT------- -F-A-SKEIP ---K-S---- KAAE--AT-- ---------- ----------
1C16_gi|741803|           FGT------- -Y-K-PEKVP ---K-S---- KSLE--AA-- ---------- ----------
1C17_gi|741805|           FGT------- -S-I-PNEVP ---K-S---- KSLE--AV-- ---------- ----------
1C18_gi|1654715|          AGCATCTCAA GAAAAAGTCT ATGGAG---- TCCA--CC-- ---------- ----------
1C20_gi|16905111|         FGT------- ---SAPQEVP ---R-S---- KATE--AT-- ---------- ----------
1C21_gi|126090770|        FGT------- -A-L-PLECP ---K-S---- KAKE--LT-- ---------- ----------
1C22_gi|38603389|         FGT------- -Y-K-PKEVP ---K-S---- KSLE--AA-- ---------- ----------
1C23_gi|62856987|         FGT------- -Y-A-PNEVP ---K-S---- KAVE--AT-- ---------- ----------
1C24_gi|84993586|         FGT------- -Y-K-PKEVP ---K-S---- KSLE--AA-- ---------- ----------
1C25_gi|15216337|         FGS------- -Y-A-PPEVP ---R-N---- RVVE--VT-- ---------- ----------
1D1_gi|5174695|           LGT------- -Y-SEPKSTP ---K-G---- ACAT--SV-- ---------- ----------
1D2_gi|398962|            LGT------- -Y-SDPRPVP -----G---- KTFI--AV-- ---------- ----------
1D3_gi|5689216|           LGT------- -Y-SEPKTTP ---K-G---- SCAT--SV-- ---------- ----------
1E1_gi|1698718|           LGT------- -WKA----SP -----G---- EVTD--AV-- ---------- ----------
1E2_gi|269849539|         LSS------- -WKA----SP -----G---- KVTE--AV-- ---------- ----------
1G1_gi|17550248|          LGT------- -WQS----SP -----A---- EVIT--AV-- ---------- ----------
2A1_gi|134153|            LGL------- -W-R----LE ---K-D---- ELKE--VI-- ---------- ----------
2A2_gi|1835701|           LGV------- -W-R----MD ---R-N---- EIKN--LL-- ---------- ----------
2B1_gi|401428|            FGC------- -W-K----VD ---V-D---- TCSE--QI-- ---------- ----------
2B2_gi|1351442|           LGC------- -W-K----MP ---N-D---- VCAD--QI-- ---------- ----------
2B3_gi|2492803|           MGC------- -WKL------ -----E---- NAAD--MV-- ---------- ----------
2B4_gi|1912051|           FGC------- -W-K----VN ---N-E---- TAAD--QI-- ---------- ----------
2B5_gi|3289019|           FGC------- -W-K----LA ---N-A---- TAGE--QV-- ---------- ----------
2B6_gi|6321895|           MRSASNNTIV EATTDGSKMA LQKSMSFDDT AAEK--TMNK SRHSYQEQFS SKKSQSSLLN
2B7_gi|1912049|           FGC------- -W-K----VT ---N-A---- TAAD--QI-- ---------- ----------
2B8_gi|4103055|           FGC------- -W-K----VD ---N-A---- TCAD--TI-- ---------- ----------
2C1_gi|1332539|           FGC------- -W-K----ID ---T-K---- DCEE--TV-- ---------- ----------
2D1_gi|7407095|           FGL------- -W-K----VN ---N-D---- TCAD--QI-- ---------- ----------
2E1_gi|4753912|           LGT---YL-- GFDKGGAVTS ---KDK---- QLRN--VV-- ---------- ----------
2E2_gi|18479021|          LGT------- ---FGFGDIP ---------- KVRQ--AV-- ---------- ----------
2E3_gi|5052610|           LGT------- -Y-T----SL ---G-G---- DCER--AT-- ---------- ----------
3A1_gi|121087|            LGT------- -WQS------ ---KEN---- DAYK--AV-- ---------- ----------
3A2_gi|6320576|           FGT------- -WRS----VD -----N---- NGYH--SV-- ---------- ----------
3B1_gi|1142698|           YGT------- -WQA----AP -----G---- EVGQ--GV-- ---------- ----------
3B2_gi|60458781|          YGT------- -WQA----EP -----G---- QVGE--GV-- ---------- ----------
3B3_gi|60458785|          FGT------- -WQA----EP -----G---- QVGA--SV-- ---------- ----------
3C1_gi|536474|            LGT------- ---ANPHEKL -----A---- ETKQ--AV-- ---------- ----------
3C2_gi|74626610|          VGT-AL---- -FKKEKGEIN -----R---- TIVD--SV-- ---------- ----------
3C3_gi|38423524|          TGT------- KWKQAQTIND ---VST---- ELVD--NI-- ---------- ----------
3D1_gi|31321885|          LGT------- -WQG------ ---DKG---- VIKE--AV-- ---------- ----------
3E1_gi|22207641|          FGT------- -F-A--SEGS ---K-G---- ETYT--AV-- ---------- ----------
3F1_gi|81625481|          LGT------- -WGIGGFETP DYSRDE---- EMVE--LL-- ---------- ----------
3F2_gi|13638516|          LGT------- -F-R----LK ---D-D---- VVIS--SV-- ---------- ----------
3F3_gi|81635765|          FGT------- -YPL------ ---KGE---- PLKA--AV-- ---------- ----------
4A1_gi|112837|            MGS------- --------AP DFTCKK---- DTKE--AI-- ---------- ----------
```

```
4A2_gi|75220959|      MGS------- --------AP DFTCKK---- DTKD--AI-- ---------- ----------
4A3_gi|1215788|       MGS------- --------AP DFTCKK---- DTKE--AI-- ---------- ----------
4A4_gi|1514979|       MGS------- --------AP DFTCKK---- DTKE--AI-- ---------- ----------
4B1_gi|2792155|       MGT------- -----SVESR ---PSN---- DVLA--SI-- ---------- ----------
4B2_gi|6478210|       MGT------- -AETMVKGTE ---------- REKL--AF-- ---------- ----------
4B3_gi|6478204|       MGT------- -AETMVKGTE ---------- REKL--AF-- ---------- ----------
4B4_gi|2792295|       MGT------- --SSYPRADP -----E---- TAKA--AI-- ---------- ----------
4B5_gi|112807104|     LGT------- ---AVQGPRP ---D------ PVRA--AV-- ---------- ----------
4B6_gi|112807098|     MGT------- ---AVQGPRP ---E------ PIRR--AV-- ---------- ----------
4B7_gi|112807100|     MGT------- ---AVQGPKP -----D---- PIRR--AV-- ---------- ----------
4B8_gi|112807102|     MGT------- ---AVQGPKP -----D---- PIRR--AV-- ---------- ----------
4C1_gi|113595|        LGT------- -WRA----GS ---------- DTAH--SV-- ---------- ----------
4C2_gi|167113|        LGT------- -WRA----GS ---------- DTAH--SV-- ---------- ----------
4C3_gi|75221432|      LGT------- -WRA----GS ---------- DTAH--SV-- ---------- ----------
4C4_gi|4539944|       LGT------- -WK------- ---------- ---SGDKA-- ---------- ----------
4C5_gi|13160397|      LGT------- -WQS----SP -----G---- DAAQ--AV-- ---------- ----------
4C6_gi|13160399|      LGT------- -WQS----SP -----G---- DAAQ--AV-- ---------- ----------
4C8_gi|111182163|     LGT------- -Y-------- -----A---- MVAT--AI-- ---------- ----------
4C9_gi|111182165|     LGT------- -WQA----SP -----G---- LVGD--AV-- ---------- ----------
4C10_gi|111182167|    LGT------- -WQA----DP -----G---- LVGN--AV-- ---------- ----------
4C11_gi|111182169|    LGT------- -WQA----AP -----G---- VVGD--AV-- ---------- ----------
5A1_gi|408360251|     LGV------- -W-Q----SP ---AGE---- VTEN--AV-- ---------- ----------
5A2_gi|11127591|      FGM------- -WKLQDGNEA ---------- ETAT------ ---------- ----------
5B1_gi|2506173|       LGV------- -FAA------ ---SAE---- ETAS--AI-- ---------- ----------
5C1_gi|144969|        YGV------- -FKV----PP -----A---- DTQR--AV-- ---------- ----------
5C2_gi|3916039|       LGV------- -WQA------ ---SNE---- EVIT--AI-- ---------- ----------
5D1_gi|112735|        LGT------- -Y-N----LR ---G-D---- EGVA--AM-- ---------- ----------
5E1_gi|5354195|       FGT------- -Y-K----LN ---G-S---- AGVS--DI-- ---------- ----------
5F1_gi|82504416|      FGV------- -FQM--SDAA ---------- ECER--AV-- ---------- ----------
5G1_gi|16080393|      LGV------- -F-K----VE ---NGN---- EATE--SV-- ---------- ----------
5G2_gi|16079957|      LGV------- -FQV--EEGS ---------- ELVN--AV-- ---------- ----------
6A1_gi|18202524|      LGT------- -WVTFGGQIT ---D-E---- MAEQ--LM-- ---------- ----------
6A2_gi|499328|        LGT------- -WVTFGGQIT ---D-E---- MAEH--LM-- ---------- ----------
6A3_gi|2135947|       LGT------- -WVTFGGQIS ---D-E---- VAER--LM-- ---------- ----------
6A4_gi|975314|        LGT------- -WVTFGGQIT ---------- DEMAEHLM-- ---------- ----------
6A5_gi|2827466|       LGT------- -WVTFGGQIT ---D-E---- MAEQ--LM-- ---------- ----------
6A6_gi|7914984|       LGT------- -WVTFGGQIT ---D-E---- MAEQ--LM-- ---------- ----------
6A7_gi|5019764|       LGT------- -WVTFGGQIS ---D-E---- VAER--LM-- ---------- ----------
6A8_gi|148747467|     LGT------- -WVTFGGQIS ---D-E---- VAER--LM-- ---------- ----------
6A9_gi|24648619|      MGLAFLRMHR IWTLLIQYSN NDHTLY---- VVRE--AR-- ---------- ----------
6A10a_gi|726465|      LGT------- -WVTFGGQIS ---D-E---- VAER--LM-- ---------- ----------
6A10b_gi|5019766|     LGT------- -WVTFGGQIS ---D-E---- VAER--LM-- ---------- ----------
6A11_gi|5922729|      LGT------- -WVTFGSQIS ---D-E---- VAEN--LM-- ---------- ----------
6A13_gi|52001073|     LGT------- -WVTFGGQIS ---D-E---- VAER--LM-- ---------- ----------
6A14_gi|226823214|    LGT------- -WVTFGSQIS ---D-E---- TAED--LL-- ---------- ----------
6B1_gi|902000|        LGT------- -WPVFSPGVS ---D-D---- QAEA--IL-- ---------- ----------
6C1_gi|1063415|       FGA------- -WVTFGNQLD ---V-K---- EAKS--IL-- ---------- ----------
6C2_gi|2832783|       YGS------- -WVSFGNQVD ---V-K---- EAKA--LL-- ---------- ----------
7A1_gi|39932720|      MGR------- -----RMDVT ---------- SSSA--SV-- ---------- ----------
7A2_gi|41327764|      LGT------- -M-EMGRRMD ---A-P---- ASAA--AV-- ---------- ----------
7A3_gi|41152114|      MGR------- -----RMDAP ---------- TSAA--VT-- ---------- ----------
7A4_gi|6815049|       LGT------- -M-EMGRRMD ---A-S---- ASAA--TV-- ---------- ----------
8A1_gi|24638123|      FGL----MGL TW------KP KQTPDE---- EAFE--VM-- ---------- ----------
8A2_gi|74627022|      LGL----MGL TW------RP KQTPIK---- QAFE--LM-- ---------- ----------
9A1_gi|146345520|     LGT--MHFGG QWTRAMGDVT ---K-E---- TAFA--LL-- ---------- ----------
9A2_gi|6093525|       LGT--MSFGN GWKGVMGECD ---Q-A---- TSFN--ML-- ---------- ----------
9A3_gi|2492798|       LGA--MSIGD KWHPYGMGTM ---DKE---- ASFK--LL-- ---------- ----------
9B1_gi|6323998|       IGD------- AWSGFMGSMN ---K-E---- QAFE--LL-- ---------- ----------
9B2_gi|6319951|       LGE------V SYDGARSDFL KSMNKN---- RAFE--LL-- ---------- ----------
9B3_gi|6319958|       ---------- ---------- ---------- QAFE--LL-- ---------- ----------
9B4_gi|6322615|       ---------- ---------- ---------- KLRD------ ---------- ----------
9C1_gi|2459734|       FGT------- -WRFGKETEQ GNVEID---- EERAHELL-- ---------- ----------
10A1_gi|4731595|      LGT------- ---SAFGQNE ---------- RAFP--VY-- ---------- ----------
10A2_gi|3256056|      LGT------- ---SAFGQDE ---------- RGFP--VY-- ---------- ----------
11A1_gi|1176985|      LGT------- -NAVGGHNLY PNLNEE---- TGKE--LV-- ---------- ----------
11B1_gi|3123233|      LGT------- -W-AIGGTMW GGTDEK---- TSIE--TI-- ---------- ----------
11B2_gi|3123121|      LGT------- -WAIGGGPAW ---NGDLDRQ ICID--TI-- ---------- ----------
11B3_gi|85062654|     LGT------- -W-QAGKRNW ADIDDQ---- EIVA--GI-- ---------- ----------
11C1_gi|81787577|     FGC------- --MSLGTDET ---------- KARR--IM-- ---------- ----------
12A1_gi|5305791|      LGT------- -V-NFSGRVP ---D-D---- QAIR--LM-- ---------- ----------
12B1_gi|2599278|      LGT------- -V-NFSGRVE ---D-D---- DALR--LM-- ---------- ----------
12C1_gi|5921163|      LGT------- VNIGGRVEEP ---------- EARR--LM-- ---------- ----------
13A1_gi|1351673|      FGC------- -MGLHAMYGP ---SSE---- EANQ--AV-- ---------- ----------
13B1_gi|9106797|      FGAMRITGPD VWGE-PEDHD ---------- EAIR--VL-- ---------- ----------
13C1_gi|81555851|     LGC------- -MGMTYGYGE VHDK-K---- QMVK--LI-- ---------- ----------
14A1_gi|882530|       LGL------- -W-HNFGHVN ---ALE---- SQRA--IL-- ---------- ----------
15A1_gi|37196700|     LGT------- -APLGGLYAP VSRA------ DADA--LL-- ---------- ----------
all2316_aldo/keto     LGG------- -MPMSIYNRP ---PES---- DSIQ--VI-- ---------- ----------
```

```
                         301
1A1_gi|5174391|          ---------K YAL-----SV GY-------- ---------- ---------- --RHIDCAAI
1A2_gi|1703236|          ---------K YAL-----TV GY-------- ---------- ---------- --RHIDCAAI
1A3_gi|1703237|          ---------K YAL-----SV GY-------- ---------- ---------- --RHIDCASV
1A4_gi|10946870|         ---------K HAL-----SA GY-------- ---------- ---------- --RHIDCASV
1B1_gi|4502049|          ---------K VAI-----DV GY-------- ---------- ---------- --RHIDCAHV
1B2_gi|1703235|          ---------K TAI-----DL GY-------- ---------- ---------- --RHIDCAHV
1B3_gi|1351911|          ---------K VAI-----DL GY-------- ---------- ---------- --RHIDCAQV
1B4_gi|6978491|          ---------K VAI-----DM GY-------- ---------- ---------- --RHIDCAQV
1B5_gi|113594|           ---------K VAI-----DL GY-------- ---------- ---------- --RHIDCAHV
1B6_gi|584742|           ---------K VAI-----DL GY-------- ---------- ---------- --RHIDCAHV
1B7_gi|231525|           ---------K AAI-----DA GY-------- ---------- ---------- --RHIDCAYV
1B8_gi|6679791|          ---------K AAI-----DA GY-------- ---------- ---------- --RHIDCAYA
1B9_gi|2114406|          ---------K VAI-----DA GY-------- ---------- ---------- --RHIDCAYA
1B10_gi|223468663|       ---------K VAI-----DA GY-------- ---------- ---------- --RHIDCAYV
1B12_gi|14330324|        ---------K HAI-----DA GY-------- ---------- ---------- --RHIDCAYF
1B13_gi|15864567|        ---------K AAI-----DA GY-------- ---------- ---------- --RHIDCAYA
1B14_gi|148540194|       ---------K AAI-----DA GY-------- ---------- ---------- --RHFDCAYV
1B15_gi|51094822|        ---------K VAI-----DA EY-------- ---------- ---------- --RHIDCAYF
1C1_gi|5453543|          ---------K LAI-----EA GF-------- ---------- ---------- --RHIDSAHL
1C2_gi|4503285|          ---------K LAI-----EA GF-------- ---------- ---------- --HHIDSAHV
1C3_gi|24497583|         ---------K LAI-----EA GF-------- ---------- ---------- --RHIDSAHL
1C4_gi|308153631|        ---------K LAI-----EA GF-------- ---------- ---------- --RHIDSAYL
1C5_gi|1352733|          ---------K IAI-----DA GF-------- ---------- ---------- --RHIDSAYF
1C6_gi|13487925|         ---------K IAI-----DA GF-------- ---------- ---------- --RHIDSASM
1C7_gi|129896|           ---------K FAI-----EV GF-------- ---------- ---------- --RHVDSAHL
1C8_gi|1709623|          ---------K IAI-----DV GF-------- ---------- ---------- --RHIDCSHL
1C9_gi|118634|           ---------K IAI-----DN GF-------- ---------- ---------- --RHFDSAYL
1C10a_gi|1345830|        ---------K IAI-----DV GY-------- ---------- ---------- --RHIDCAFI
1C10b_gi|1706132|        ---------K TAI-----DV GY-------- ---------- ---------- --RHIDCAFI
1C11_gi|1669605|         ---------K FAI-----EA GF-------- ---------- ---------- --RHIDSAHL
1C12_gi|85719330|        ---------C LAL-----DV GY-------- ---------- ---------- --RHVDTAYA
1C13_gi|171846276|       ---------C LAL-----DV GY-------- ---------- ---------- --RHVDTAYA
1C14_gi|19527294|        ---------K IAI-----DT GF-------- ---------- ---------- --RHFDSAYL
1C15_gi|741804|          ---------K VAI-----DV GF-------- ---------- ---------- --RHIDAAYF
1C16_gi|741803|          ---------H LAI-----DA GY-------- ---------- ---------- --RHIDTAYA
1C17_gi|741805|          ---------H LAI-----DA GY-------- ---------- ---------- --HHIDTASA
1C18_gi|1654715|         ---------A AAA-----TA GCTATAGATG TTGGGTTCTG CCATATTGAT TGTTCTCACT
1C20_gi|16905111|        ---------K IAI-----DA GF-------- ---------- ---------- --RHIDCAAV
1C21_gi|126090770|       ---------K IAI-----DA GF-------- ---------- ---------- --HHFDSASV
1C22_gi|38603389|        ---------C LAL-----DV GY-------- ---------- ---------- --RHVDTAYA
1C23_gi|62856987|        ---------K SAI-----DA GF-------- ---------- ---------- --RHIDCAHL
1C24_gi|84993586|        ---------H LAI-----DV GY-------- ---------- ---------- --RHIDTASA
1C25_gi|15216337|        ---------K LAI-----EA GF-------- ---------- ---------- --RHIDSAYL
1D1_gi|5174695|          ---------K VAI-----DT GY-------- ---------- ---------- --RHIDGAYI
1D2_gi|398962|           ---------K TAI-----DE GY-------- ---------- ---------- --RHIDGAYV
1D3_gi|5689216|          ---------K IAI-----DT GY-------- ---------- ---------- --RHIDGAYI
1E1_gi|1698718|          ---------K LAI-----NL GY-------- ---------- ---------- --RHFDCAYL
1E2_gi|269849539|        ---------K EAI-----DA GY-------- ---------- ---------- --RHFDCAYF
1G1_gi|17550248|         ---------K TAV-----KA GY-------- ---------- ---------- --RLIDTASV
2A1_gi|134153|           ---------L NAI-----KI GY-------- ---------- ---------- --RHFDCAAH
2A2_gi|1835701|          ---------L SAI-----NL GY-------- ---------- ---------- --RHFDCAAD
2B1_gi|401428|           ---------Y RAI-----KT GY-------- ---------- ---------- --RLFDGAED
2B2_gi|1351442|          ---------Y EAI-----KI GY-------- ---------- ---------- --RLFDGAQD
2B3_gi|2492803|          ---------Y AAI-----KE GY-------- ---------- ---------- --RLFDCACD
2B4_gi|1912051|          ---------Y NAI-----KT GY-------- ---------- ---------- --RLFDGAED
2B5_gi|3289019|          ---------Y QAI-----KA GY-------- ---------- ---------- --RLFDGAED
2B6_gi|6321895|          SKQRSRAKSQ TCS-----ST GY-------- ---------- ---------- --NNSSILKT
2B7_gi|1912049|          ---------Y NAI-----KT GY-------- ---------- ---------- --RLFDGAED
2B8_gi|4103055|          ---------Y NAI-----KV GY-------- ---------- ---------- --RLFDGAED
2C1_gi|1332539|          ---------Y QAI-----KT GY-------- ---------- ---------- --RLFDGACD
2D1_gi|7407095|          ---------Y HAI-----KE GY-------- ---------- ---------- --RLFDGACD
2E1_gi|4753912|          ---------M QAI-----DL GY-------- ---------- ---------- --RHFDTAAI
2E2_gi|18479021|         ---------L WAI-----QA GY-------- ---------- ---------- --RHIDTAAL
2E3_gi|5052610|          ---------L HAI-----DV GY-------- ---------- ---------- --RHIDTAYF
3A1_gi|121087|           ---------L TAL-----KD GY-------- ---------- ---------- --RHIDTAAI
3A2_gi|6320576|          ---------I AAL-----KA GY-------- ---------- ---------- --RHIDAAAI
3B1_gi|1142698|          ---------K VAI-----ET GY-------- ---------- ---------- --RHLDLAKV
3B2_gi|60458781|         ---------K LAV-----KA GY-------- ---------- ---------- --RHLDLAKV
3B3_gi|60458785|         ---------K NAV-----KA GY-------- ---------- ---------- --RHLDLAKV
3C1_gi|536474|           ---------K AAI-----KA GY-------- ---------- ---------- --RHIDTAWA
3C2_gi|74626610|         ---------K NAL-----AA GF-------- ---------- ---------- --IHIDCAEV
3C3_gi|38423524|         ---------L LGL-----KL GF-------- ---------- ---------- --RHIDTAEA
3D1_gi|31321885|         ---------L TAI-----KS GY-------- ---------- ---------- --RLIDGAYV
3E1_gi|22207641|         ---------T TAL-----KT GY-------- ---------- ---------- --RHLDCAWY
3F1_gi|81625481|         ---------K TAI-----KM GY-------- ---------- ---------- --THIDTAEY
3F2_gi|13638516|         ---------I TAL-----EL GY-------- ---------- ---------- --RAIDTAQI
3F3_gi|81635765|         ---------R EAI-----TV GY-------- ---------- ---------- --RAFDTAQM
4A1_gi|112837|           ---------I EAV-----KQ GY-------- ---------- ---------- --RHFDTAAA
```

```
4A2_gi|75220959|       ----------I EAI-----KQ GY--------  ----------  ----------  --RHFDTAAA
4A3_gi|1215788|        ----------I EAI-----KQ GY--------  ----------  ----------  --RHFDTAAA
4A4_gi|1514979|        ----------I EAI-----KQ GY--------  ----------  ----------  --RHFDTAAA
4B1_gi|2792155|        ---------FV DAI-----QV GY--------  ----------  ----------  --RHFDSASV
4B2_gi|6478210|        ----------L KAI-----EV GY--------  ----------  ----------  --RHFDTAAA
4B3_gi|6478204|        ----------L KAI-----EV GY--------  ----------  ----------  --RHFDTAAA
4B4_gi|2792295|        ----------L EAI-----RA GY--------  ----------  ----------  --RHFDTAAA
4B5_gi|112807104|      ----------L RAI-----QL GY--------  ----------  ----------  --RHFDTAAH
4B6_gi|112807098|      ----------L KAI-----EA GY--------  ----------  ----------  --RHFDTAAH
4B7_gi|112807100|      ----------L RAI-----EI GY--------  ----------  ----------  --RHFDTAAH
4B8_gi|112807102|      ----------L RAI-----EV GY--------  ----------  ----------  --RHFDTAAH
4C1_gi|113595|         ----------R TAI----TEA GY--------  ----------  ----------  --RHVDTAAE
4C2_gi|167113|         ----------Q TAI----TEA GY--------  ----------  ----------  --RHVDTAAE
4C3_gi|75221432|       ----------Q TAI    TEA GY--------  ----------  ----------  --RHVDTAAQ
4C4_gi|4539944|        ----------G NAVYTAITEG GY--------  ----------  ----------  --RHIDTAAQ
4C5_gi|13160397|       ----------E VAI     KC GY--------  ----------  ----------  --RHIDGARL
4C6_gi|13160399|       ----------E VAI     KC GY--------  ----------  ----------  --RHIDGARL
4C8_gi|111182163|      ----------E QAI-----KI GY--------  ----------  ----------  --RHIDCASI
4C9_gi|111182165|      ----------A AAV-----KI GY--------  ----------  ----------  --RHIDCAQI
4C10_gi|111182167|     ----------D AAV-----KI GY--------  ----------  ----------  --RHIDCAQI
4C11_gi|111182169|     ----------A AAV-----KI GY--------  ----------  ----------  --QHIDCASR
5A1_gi|408360251|      ----------K WAL-----CA GY--------  ----------  ----------  --RHIDTAAI
5A2_gi|11127591|       ----------M WAI-----KS GY--------  ----------  ----------  --RHIDTAAI
5B1_gi|2506173|        ----------A SAI-----SS GY--------  ----------  ----------  --RLIDTARS
5C1_gi|144969|         ----------E EAL-----EV GY--------  ----------  ----------  --RHIDTAAI
5C2_gi|3916039|        ----------Q KAL-----EV GY--------  ----------  ----------  --RSIDTAAA
5D1_gi|112735|         ----------V AAI-----DS GY--------  ----------  ----------  --RLLDTAVN
5E1_gi|5354195|        ----------V SAI-----KV GY--------  ----------  ----------  --RLLDSAFN
5F1_gi|82504416|       ----------I DAI-----DT GY--------  ----------  ----------  --RLIDTAAS
5G1_gi|16080393|       ----------K AAI-----KN GY--------  ----------  ----------  --RSIDTAAI
5G2_gi|16079957|       ----------K TAI-----VH GY--------  ----------  ----------  --RSIDTAAI
6A1_gi|18202524|       ----------T LAY-----DN GI--------  ----------  ----------  --NLFDTAEV
6A2_gi|499328|         ----------T LAY-----DN GI--------  ----------  ----------  --NLFDTAEV
6A3_gi|2135947|        ----------T IAY-----ES GV--------  ----------  ----------  --NLFDTAEV
6A4_gi|975314|         ----------T LAY-----DN GI--------  ----------  ----------  --NLFDTAEV
6A5_gi|2827466|        ----------T LAY-----DN GI--------  ----------  ----------  --NLFDTAEV
6A6_gi|7914984|        ----------T LAY-----DN GI--------  ----------  ----------  --NLFDTAEV
6A7_gi|5019764|        ----------T IAY-----ES GV--------  ----------  ----------  --NLFDTAEV
6A8_gi|148747467|      ----------T IAY-----ES GV--------  ----------  ----------  --NLFDTAEV
6A9_gi|24648619|       ----------Q VLY-----NM VY--------  ----------  ----------  --KSCDKLHD
6A10a_gi|726465|       ----------T IAY-----ES GV--------  ----------  ----------  --NLFDTAEV
6A10b_gi|5019766|      ----------T IAY-----ES GV--------  ----------  ----------  --NLFDTAEV
6A11_gi|5922729|       ----------T VAY-----EH GV--------  ----------  ----------  --NLFDTAEV
6A13_gi|52001073|      ----------T IAY-----ES GV--------  ----------  ----------  --NLFDTAEV
6A14_gi|226823214|     ----------T VAY-----EH GV--------  ----------  ----------  --NLFDTAEV
6B1_gi|902000|         ----------K LAI-----ES GI--------  ----------  ----------  --NLFDISEA
6C1_gi|1063415|        ----------Q CCR-----DH GV--------  ----------  ----------  --NFFDNAEV
6C2_gi|2832783|        ----------Q CCR-----DH GV--------  ----------  ----------  --NFFDNAEV
7A1_gi|39932720|       ----------R AFL-----QR GH--------  ----------  ----------  --TEIDTAFV
7A2_gi|41327764|       ----------R AFL-----ER GH--------  ----------  ----------  --TELDTAFM
7A3_gi|41152114|       ----------R AFL-----ER GH--------  ----------  ----------  --TEIDTAFV
7A4_gi|6815049|        ----------R AFL-----ER GL--------  ----------  ----------  --NELDTAFM
8A1_gi|24638123|       ----------N YAL-----SQ GS--------  ----------  ----------  --NYWDAGEF
8A2_gi|74627022|       ----------N YAL-----SQ GS--------  ----------  ----------  --NYWNAGEF
9A1_gi|146345520|      ----------D RFY-----EA GG--------  ----------  ----------  --NFIDTANF
9A2_gi|6093525|        ----------D TFY-----ES GG--------  ----------  ----------  --NFIDVANF
9A3_gi|2492798|        ----------D AFY-----NA GG--------  ----------  ----------  --NFIDTANV
9B1_gi|6323998|        ----------D AFY-----EA GG--------  ----------  ----------  --NCIDTANS
9B2_gi|6319951|        ----------D TFY-----EA GG--------  ----------  ----------  --NFIDAANN
9B3_gi|6319958|        ----------D AFY-----EA GG--------  ----------  ----------  --NCIDTANS
9B4_gi|6322615|        ----------Q IVI-----AT KF--------  ----------  ----------  -------TTD
9C1_gi|2459734|        ----------D AYE-----AA GG--------  ----------  ----------  --RFIDTADI
10A1_gi|4731595|       ----------D AYW-----EG GG--------  ----------  ----------  --RAFDTAWL
10A2_gi|3256056|       ----------D AYW-----EG GG--------  ----------  ----------  --RAFDTAWL
11A1_gi|1176985|       ----------R EAI-----RN GV--------  ----------  ----------  --TMLDTAYI
11B1_gi|3123233|       ----------R AAL-----DQ GI--------  ----------  ----------  --TLIDTAPA
11B2_gi|3123121|       ----------L EAH-----RC GI--------  ----------  ----------  --NLIDTAPG
11B3_gi|85062654|      ----------R AAV-----DA GI--------  ----------  ----------  --TTIDTAEI
11C1_gi|81787577|      ----------D EVL-----EL GI--------  ----------  ----------  --NYLDTADL
12A1_gi|5305791|       ----------D EAL-----DR GV--------  ----------  ----------  --NCVDTADI
12B1_gi|2599278|       ----------D HAR-----DR GI--------  ----------  ----------  --NCLDTADM
12C1_gi|5921163|       ----------D HAL-----AQ GI--------  ----------  ----------  --TLFDTANT
13A1_gi|1351673|       ---------LT HAA-----DL GC--------  ----------  ----------  --TFWDSSDM
13B1_gi|9106797|       ----------K RLP-----EI GV--------  ----------  ----------  --DLIDTADS
13C1_gi|81555851|      ----------H KAL-----EL GI--------  ----------  ----------  --NFFDTAEA
14A1_gi|882530|        ----------R KAF-----DL GI--------  ----------  ----------  --THFDLANN
15A1_gi|37196700|      ----------E AGW-----DS GI--------  ----------  ----------  --RYFDSAPM
all2316_aldo/keto      ----------H RAL-----DL GI--------  ----------  ----------  --TFIDTADS
```

```
                              361
1A1_gi|5174391|               Y-----G--- ---NEPEIGE A--------- ---------- ------LK-- --EDVGPGKA
1A2_gi|1703236|               Y-----G--- ---NELEIGE A--------- ---------- ------LT-- --ETVGPGKA
1A3_gi|1703237|               Y-----G--- ---NETEIGE A--------- ---------- ------LK-- --ESVGAGKA
1A4_gi|10946870|              Y-----G--- ---NETEIGE A--------- ---------- ------LK-- --ESVGSGKA
1B1_gi|4502049|               Y-----Q--- ---NENEVGV A--------- ---------- ------IQ-- --EKLREQ-V
1B2_gi|1703235|               Y-----Q--- ---NENEVGV A--------- ---------- ------LQ-- --EKLKEQ-V
1B3_gi|1351911|               Y-----Q--- ---NEKEVGV A--------- ---------- ------LQ-- --EKLKEQ-V
1B4_gi|6978491|               Y-----Q--- ---NEKEVGV A--------- ---------- ------LQ-- --EKLKEQ-V
1B5_gi|113594|                Y-----Q--- ---NENEVGL A--------- ---------- ------LQ-- --AKLQEQ-V
1B6_gi|584742|                Y-----Q--- ---NENEVGL G--------- ---------- ------LQ-- --EKLQGQ-V
1B7_gi|231525|                Y-----H--- ---NENEVGE A--------- ---------- ------IQ-- --EKIKEN-A
1B8_gi|6679791|               Y-----C--- ---NENEVGE A--------- ---------- ------IQ-- --EKIKEK-A
1B9_gi|2114406|               Y-----Y--- ---NEHEVGE A--------- ---------- ------IQ-- --EKIKEK-A
1B10_gi|223468663|            Y-----Q--- ---NEHEVGE A--------- ---------- ------IQ-- --EKIQEK-A
1B12_gi|14330324|             Y-----Q--- ---NEHEIGN A--------- ---------- ------IK-- --QKIKEG-A
1B13_gi|15864567|             Y-----C--- ---NENEVGE A--------- ---------- ------IQ-- --EKIKEK-A
1B14_gi|148540194|            Y-----Q--- ---NESEVGE A--------- ---------- ------IQ-- --EKIKEK-A
1B15_gi|51094822|             Y-----E--- ---NQHEVGE A--------- ---------- ------IQ-- --EKIQEK-A
1C1_gi|5453543|               Y-----N--- ---NEEQVGL A--------- ---------- ------IR-- --SKIADG-S
1C2_gi|4503285|               Y-----N--- ---NEEQVGL A--------- ---------- ------IR-- --SKIADG-S
1C3_gi|24497583|              Y-----N--- ---NEEQVGL A--------- ---------- ------IR-- --SKIADG-S
1C4_gi|308153631|             Y-----N--- ---NEEQVGL A--------- ---------- ------IR-- --SKIADG-S
1C5_gi|1352733|               Y-----K--- ---NEKEVGL A--------- ---------- ------IR-- --SKIADG-T
1C6_gi|13487925|              Y-----Q--- ---NEKEVGL A--------- ---------- ------IR-- --SKIADG-T
1C7_gi|129896|                Y-----Q--- ---NEEQVGQ A--------- ---------- ------IR-- --SKIADG-T
1C8_gi|1709623|               Y-----Q--- ---NEEEIGQ A--------- ---------- ------IV-- --SKIEDG-T
1C9_gi|118634|                Y-----E--- ---VEEEVGQ A--------- ---------- ------IR-- --SKIEDG-T
1C10a_gi|1345830|             T-----G--- ---NEMHIGN G--------- ---------- ------IR-- --SKISDG-T
1C10b_gi|1706132|             T-----G--- ---NEMHIGN G--------- ---------- ------IR-- --SKISDG-T
1C11_gi|1669605|              Y-----Q--- ---NEEQVGQ A--------- ---------- ------IR-- --SKIADG-T
1C12_gi|85719330|             Y-----Q--- ---VEEEIGQ A--------- ---------- ------IQ-- --SKIKAG-V
1C13_gi|171846276|            Y-----Q--- ---VEEEIGQ A--------- ---------- ------IQ-- --SKIKAG-V
1C14_gi|19527294|             Y-----Q--- ---IEEEVGQ A--------- ---------- ------IR-- --SKIEDG-T
1C15_gi|741804|               Y-----Q--- ---NEEEVGQ A--------- ---------- ------LR-- --DKMADG-T
1C16_gi|741803|               Y-----Q--- ---IEEEIGQ A--------- ---------- ------IQ-- --SKIKAG-V
1C17_gi|741805|               Y-----Q--- ---IEEEIGQ A--------- ---------- ------IQ-- --SKIKAG
1C18_gi|1654715|              T-----G--- ---TACCAGA A--------- ---------- ------TG-- --AAGAAGAG
1C20_gi|16905111|             Y-----Q--- ---NEKEVGL A--------- ---------- ------IR-- --SKIVDG-T
1C21_gi|126090770|            Y-----N--- ---TEDHVGE A--------- ---------- ------IR-- --SKIADG-T
1C22_gi|38603389|             Y-----Q--- ---VEEEIGQ A--------- ---------- ------IQ-- --SKIKAG-V
1C23_gi|62856987|             Y-----D--- ---NEKEVGL A--------- ---------- ------IR-- --SKIQDG-T
1C24_gi|84993586|             Y-----Q--- ---VEEEIGQ A--------- ---------- ------IQ-- --SKIKAG-V
1C25_gi|15216337|             Y-----N--- ---NEEQVGL A--------- ---------- ------IR-- --SKIADG-S
1D1_gi|5174695|               Y-----Q--- ---NEHEVGE A--------- ---------- ------IR-- --EKIAEG-K
1D2_gi|398962|                Y-----R--- ---NEHEVGE A--------- ---------- ------IR-- --EKVAEG-K
1D3_gi|5689216|               Y-----Q--- ---NEHEVGE T--------- ---------- ------FR-- --EKIAEG-K
1E1_gi|1698718|               Y-----H--- ---NESEVGM G--------- ---------- ------IS-- --EKIKEG-V
1E2_gi|269849539|             Y-----H--- ---NEREVGA G--------- ---------- ------IR-- --CKIKEG-A
1G1_gi|17550248|              Y-----Q--- ---NEEAIGT A--------- ---------- ------IK-- --ELLEEG-V
2A1_gi|134153|                Y-----K--- ---SEADVGE A--------- ---------- ------LA-- --EAFKTG-L
2A2_gi|1835701|               Y-----K--- ---NELEVGE A--------- ---------- ------FK-- --EAFDTD-L
2B1_gi|401428|                Y-----A--- ---NEKLVGA G--------- ---------- ------VK-- --KAIDEG-I
2B2_gi|1351442|               Y-----A--- ---NEKEVGQ G--------- ---------- ------VN-- --RAIKEG-L
2B3_gi|2492803|               Y-----G--- ---NEKEVGE G--------- ---------- ------IN-- --RAIKDG-L
2B4_gi|1912051|               Y-----G--- ---NEKEVGE G--------- ---------- ------IN-- --RAIKEG-L
2B5_gi|3289019|               Y-----G--- ---NEKEVGD G--------- ---------- ------VK-- --RAIDEG-L
2B6_gi|6321895|               F-----GISS KISNSSDRIE ASSLEFNVPS QKPLNCKPLT PSQKYRLR-K EQSEMNLRNT
2B7_gi|1912049|               Y-----G--- ---NEKEVGE G--------- ---------- ------IN-- --RAIKEG-L
2B8_gi|4103055|               Y-----G--- ---NEKEVGD G--------- ---------- ------IN-- --RALDEG-L
2C1_gi|1332539|               Y-----G--- ---NEVEVGR G--------- ---------- ------IN-- --KAINEG-L
2D1_gi|7407095|               Y-----G--- ---NEVEAGQ G--------- ---------- ------IA-- --RAIKDG-L
2E1_gi|4753912|               Y-----N--- ---TEAEVGE A--------- ---------- ------IR-- --MKIDEG-V
2E2_gi|18479021|              Y-----G--- ---NEEEVGK G--------- ---------- ------IA-- --DAIQQG-L
2E3_gi|5052610|               Y-----E--- ---NENEVGA A--------- ---------- ------VQ-- --RKIAEG-V
3A1_gi|121087|                Y-----R--- ---NEDQVGQ A--------- ---------- ------IK-- -----DSG--
3A2_gi|6320576|               Y-----L--- ---NEEEVGR A--------- ---------- ------IK-- -----DSG--
3B1_gi|1142698|               Y-----S--- ---NQPEVGA A--------- ---------- ------IK-- -----EAG--
3B2_gi|60458781|              Y-----Q--- ---NQTEIGQ A--------- ---------- ------LK-- --ELFDEG-V
3B3_gi|60458785|              Y-----Q--- ---NQSEIGV A--------- ---------- ------LQ-- --ELFDQG-I
3C1_gi|536474|                Y-----E--- ---TEPFVGE A--------- ---------- ------IK-- --ELLEDG-S
3C2_gi|74626610|              Y-----G--- ---NEEEVGV A--------- ---------- ------LK-- --EA     N
3C3_gi|38423524|              Y-----N--- ---TQKEVGE A--------- ---------- ------LK-- --RT     D
3D1_gi|31321885|              Y-----G--- ---NEEEVGQ G--------- ---------- ------IR-- --EAISSG-I
3E1_gi|22207641|              Y-----L--- ---NEGEVGE G--------- ---------- ------IR-- --DFLKENPS
3F1_gi|81625481|              Y-----G--- GGHTEELIGK A--------- ---------- ------IK--          D
3F2_gi|13638516|              Y-----D--- ---NEAAVGQ A--------- ---------- ------IA-- -----ESG--
3F3_gi|81635765|              Y-----G--- ---NEAETGE A--------- ---------- ------LA-- -----ESG--
4A1_gi|112837|                Y-----G--- ---SEQALGE A--------- ---------- ------LK-- --EAIHLG-L
```

```
4A2_gi|75220959|       Y-----G--- ---SEQALGE A--------- ---------- ------LK-- --EAIELG-L
4A3_gi|1215788|        Y-----G--- ---SETALGE A--------- ---------- ------LK-- --EARDLG-L
4A4_gi|1514979|        Y-----G--- ---SETALGE A--------- ---------- ------LK-- --EARDLG-L
4B1_gi|2792155|        Y-----G--- ---TEEAIGM A--------- ---------- ------VS-- --KAIEQGLI
4B2_gi|6478210|        Y-----Q--- ---SEECLGE A--------- ---------- ------IA-- --EALQLGLI
4B3_gi|6478204|        Y-----Q--- ---TEECLGE A--------- ---------- ------IA-- --EALQLGLI
4B4_gi|2792295|        Y-----G--- ---SEKDLGE A--------- ---------- ------IA-- --EALRLQLI
4B5_gi|112807104|      Y-----A--- ---TEAPIGE A--------- ---------- ------AA-- --EAVRTGLV
4B6_gi|112807098|      Y-----E--- ---TEAPIGE A--------- ---------- ------AA-- --EAVRSGAI
4B7_gi|112807100|      Y-----E--- ---TEAPIGE A--------- ---------- ------AA-- --EAVRSGAV
4B8_gi|112807102|      Y-----E--- ---TEAPIGE A--------- ---------- ------AA-- --EAVRSGAV
4C1_gi|113595|         Y-----G--- ---VEKEVGK G--------- ---------- ------LK-- --AAMEAG--
4C2_gi|167113|         Y-----G--- ---VEKEVGK G--------- ---------- ------LK-- --AAMEAG--
4C3_gi|75221432|       Y-----G--- ---IEKEVDK G--------- ---------- ------LK-- --AAMEAG--
4C4_gi|4539944|        Y-----G--- ---VHEEVGN A--------- ---------- ------LQ-- --SALKAG--
4C5_gi|13160397|       Y-----E--- ---NEKEIGV V--------- ---------- ------LK-- --KLFDDG-V
4C6_gi|13160399|       Y-----E--- ---NEKEIGV V--------- ---------- ------LK-- --KLFDDG-V
4C8_gi|111182163|      Y-----G--- ---NEKEIGG V--------- ---------- ------LK-- --KLIGDG-F
4C9_gi|111182165|      Y-----G--- ---NEKEIGA V--------- ---------- ------LK-- --KLFEDR-V
4C10_gi|111182167|     Y-----G--- ---NEKEIGL V--------- ---------- ------LK-- --KLFDGG-V
4C11_gi|111182169|     Y-----G--- ---NEIEIGK V--------- ---------- ------LK-- --KLFDDG-V
5A1_gi|408360251|      Y-----K--- ---NEESVGA G--------- ---------- ------LR-- --A---SG--
5A2_gi|11127591|       Y-----K--- ---NEESAGR A--------- ---------- ------IA-- -----SCG--
5B1_gi|2506173|        Y-----N--- ---NEAQVGE G--------- ---------- ------IR-- -----NSG--
5C1_gi|144969|         Y-----G--- ---NEEGVGA A--------- ---------- ------IA-- -----ASG--
5C2_gi|3916039|        Y-----K--- ---NEEGVGK A--------- ---------- ------LK-- -------NAS
5D1_gi|112735|         Y-----E--- ---NESEVGR A--------- ---------- ------VR-- -----ASS--
5E1_gi|5354195|        Y-----E--- ---NEGAVGE V--------- ---------- ------FR-- -----EAG--
5F1_gi|82504416|       Y-----Q--- ---NETQVGN A--------- ---------- ------LK-- -----QTG--
5G1_gi|16080393|       Y-----K--- ---NEEGVGI G--------- ---------- ------IK-- -----ESG--
5G2_gi|16079957|       Y-----G--- ---NEAGVGE G--------- ---------- ------IR-- --EGIEEA-G
6A1_gi|18202524|       Y-----A--- AGKAEVVLGN I--------- ---------- ------IK-- -----KKG--
6A2_gi|499328|         Y-----A--- AGKAEVVLGN I--------- ---------- ------IK-- -----KKG--
6A3_gi|2135947|        Y-----A--- AGKAEVILGS I--------- ---------- ------IK-- -----KKG--
6A4_gi|975314|         Y-----A--- AGKAEVVFRN I--------- ---------- ------IK-- -----KKG--
6A5_gi|2827466|        Y-----A--- AGKAEVVLGN I--------- ---------- ------IK-- -----KKG--
6A6_gi|7914984|        Y-----A--- AGKAEVVLGN I--------- ---------- ------IK-- -----KKG--
6A7_gi|5019764|        Y-----A--- AGKAEVILGS I--------- ---------- ------IK-- -----KKG--
6A8_gi|148747467|      Y-----A--- AGKAEVILGS I--------- ---------- ------IK-- -----KKG--
6A9_gi|24648619|       K-----A--- --VTLEILSE I--------- ---------- ------MQPI HDNLYKNTDG
6A10a_gi|726465|       Y-----A--- AGKAEVILGS I--------- ---------- ------IK-- -----KKG--
6A10b_gi|5019766|      Y-----A--- AGKAEVILGS I--------- ---------- ------IK-- -----KKG--
6A11_gi|5922729|       Y-----A--- AGRAERTLGK I--------- ---------- ------LK-- -------KKE
6A13_gi|52001073|      Y-----A--- AGKAEVILGS I--------- ---------- ------IK-- -----KKG--
6A14_gi|226823214|     Y-----A--- AGKAERTLGN I--------- ---------- ------LK-- -----SKG--
6B1_gi|902000|         H--------- ---SETEIGK I--------- ---------- ------LQ-- -----RAG--
6C1_gi|1063415|        Y-----A--- NGRAEEIMGQ A--------- ---------- ------IR-- -----ELG--
6C2_gi|2832783|        Y-----A--- NGRAEEIMGQ A--------- ---------- ------FR-- -----ELG--
7A1_gi|39932720|       Y-----A--- NGQSETILGD L--------- ---------- ---------- ---GLGLGRS
7A2_gi|41327764|       Y-----S--- DGQSETILGG L--------- ---------- ---------- ---GLGLGGG
7A3_gi|41152114|       Y-----S--- EGQSETILGG L--------- ---------- ---------- ---GLRLGGS
7A4_gi|6815049|        Y-----C--- DGQSESILGS ---------- ---------- ------LG-- --LGLGSGDC
8A1_gi|24638123|       Y-----GVDP PTSNLDLLAR Y--------- ---------- ------FE-- --KYPENA--
8A2_gi|74627022|       Y-----GINP PTANLDLLAD Y--------- ---------- ------FE-- --KYPKNA--
9A1_gi|146345520|      Y-----Q--- GEGSEKWLGE W--------- ---------- ------VA-- -----SRG--
9A2_gi|6093525|        Y-----Q--- GGDTERWVGE W--------- ---------- ------MA-- --------QR
9A3_gi|2492798|        Y-----Q--- DETSEEFIGE W--------- ---------- ------ME-- -----ARG--
9B1_gi|6323998|        Y-----Q--- NEESEIWIGE W--------- ---------- ------MA-- --------SR
9B2_gi|6319951|        C-----Q--- NEQSEEWIGE W--------- ---------- ------IQ-- -SRRLRDQIV
9B3_gi|6319958|        Y-----Q--- NEESEIWIGE W--------- ---------- ------MK-- -SRKLRDQIV
9B4_gi|6322615|        Y--------- ---KGYDVGK G--------- ---------- ------KS-- ----------
9C1_gi|2459734|        Y-----G--- GGASERWIGD W--------- ---------- ------LE-- ---------D
10A1_gi|4731595|       Y-----G--- ---YDYGPGC C--------- ---------- ---------- --ERAFGAWA
10A2_gi|3256056|       YG----HAYG PGCCERTFGA W--------- ---------- ------AR-- -----SRG--
11A1_gi|1176985|       Y-----G--- IGRSEELIGE V--------- ---------- ------LR-- ---------E
11B1_gi|3123233|       Y-----G--- FGQSEEIVGK A--------- ---------- ------IK-- -----EYG--
11B2_gi|3123121|       Y-----N--- FGNSEVIVGQ A--------- ---------- ------LK-- ---------K
11B3_gi|85062654|      Y-----G--- DGDSERRVAE A--------- ---------- ------IA-- ----------
11C1_gi|81787577|      Y-----N--- QGLNEQFVGK A--------- ---------- ------LK-- ----------
12A1_gi|5305791|       YGWRLYK--- -GHTEELVGR W--------- ---------- ------LR-- -----GSG--
12B1_gi|2599278|       YGWRLYK--- -GHTEELVGR W--------- ---------- ------LA-- -----QGG--
12C1_gi|5921163|       YGWRVHK--- -GYTEEVIGR W--------- ---------- ------LA-- -------DRP
13A1_gi|1351673|       Y-----G--- FGANEECIGR W--------- ---------- ------FK-- -----QTG--
13B1_gi|9106797|       Y-----G--- PFVSEQLIAD A--------- ---------- ------LHPY GGIKIATKGS
13C1_gi|81555851|      Y-----G--- -EDNEKLLGE A--------- ---------- ------IK-- --PFKDKVVV
14A1_gi|882530|        Y-----GPP- PGSAEENFGR L--------- ---------- ------LR-- ------EDFA
15A1_gi|37196700|      Y-----G--- YGRCEHLLGD M--------- ---------- ------LR-- --EKPERAVI
all2316_aldo/keto      YCKDESD--- KHHNERLIHK A--------- ---------- ------LS-- --TYAGDT--
```

```
                     421
1A1_gi|5174391|        VPR-----EE ---------- ---------- ---------- -LFVTS---- KL--------
1A2_gi|1703236|        VPR-----EE ---------- ---------- ---------- -LFVTS---- KL--------
1A3_gi|1703237|        VPR-----EE ---------- ---------- ---------- -LFVTS---- KL--------
1A4_gi|10946870|       VPR-----EE ---------- ---------- ---------- -LFVTS---- KL--------
1B1_gi|4502049|        VKR-----EE ---------- ---------- ---------- -LFIVS---- KL--------
1B2_gi|1703235|        VKR-----EE ---------- ---------- ---------- -LFIVS---- KL--------
1B3_gi|1351911|        VKR-----QD ---------- ---------- ---------- -LFIVS---- KL--------
1B4_gi|6978491|        VKR-----QD ---------- ---------- ---------- -LFIVS---- KL--------
1B5_gi|113594|         VKR-----ED ---------- ---------- ---------- -LFIVS---- KL--------
1B6_gi|584742|         VKR-----ED ---------- ---------- ---------- -LFIVS---- KL--------
1B7_gi|231525|         VKR-----ED ---------- ---------- ---------- -LFIVS---- KL--------
1B8_gi|6679791|        VQR-----ED ---------- ---------- ---------- -LFIVS---- KL--------
1B9_gi|2114406|        VRR-----ED ---------- ---------- ---------- -LFIVS---- KL--------
1B10_gi|223468663|     VKR-----ED ---------- ---------- ---------- -LFIVS---- KL--------
1B12_gi|14330324|      VKR-----ED ---------- ---------- ---------- -LFVVT---- KL--------
1B13_gi|15864567|      VRR-----ED ---------- ---------- ---------- -LFIVS---- KL--------
1B14_gi|148540194|     VRR-----ED ---------- ---------- ---------- -LFIVS---- KL--------
1B15_gi|51094822|      VMR-----ED ---------- ---------- ---------- -LFIVS---- KV--------
1C1_gi|5453543|        VKR-----ED ---------- ---------- ---------- -IFYTS---- KL--------
1C2_gi|4503285|        VKR-----ED ---------- ---------- ---------- -IFYTS---- KL--------
1C3_gi|24497583|       VKR-----ED ---------- ---------- ---------- -IFYTS---- KL--------
1C4_gi|308153631|      VKR-----ED ---------- ---------- ---------- -IFYTS---- KL--------
1C5_gi|1352733|        VKR-----ED ---------- ---------- ---------- -IFYTS---- KL--------
1C6_gi|13487925|       VKR-----ED ---------- ---------- ---------- -IFYTS---- KV--------
1C7_gi|129896|         VKR-----ED ---------- ---------- ---------- -IFYTS---- KL--------
1C8_gi|1709623|        VKR-----ED ---------- ---------- ---------- -IFYTS---- KL--------
1C9_gi|118634|         VKR-----ED ---------- ---------- ---------- -IFYTS---- KL--------
1C10a_gi|1345830|      VKR-----ED ---------- ---------- ---------- -IFYTG---- KL--------
1C10b_gi|1706132|      VKR-----ED ---------- ---------- ---------- -IFYTG---- KL--------
1C11_gi|1669605|       VKR-----ED ---------- ---------- ---------- -IFYTS---- KL--------
1C12_gi|85719330|      VKR-----ED ---------- ---------- ---------- -LFITT---- KL--------
1C13_gi|171846276|     VKR-----ED ---------- ---------- ---------- -LFITT---- KL--------
1C14_gi|19527294|      VKR-----ED ---------- ---------- ---------- -IFYTS---- KL--------
1C15_gi|741804|        VKR-----ED ---------- ---------- ---------- -LFYTT---- KI--------
1C16_gi|741803|        VKR-----ED ---------- ---------- ---------- -MFITT---- KL--------
1C17_gi|741805|        VKR-----ED ---------- ---------- ---------- -MFITT---- KL--------
1C18_gi|1654715|       ATAGGCCAGG ---------- ---------- ---------- -CCATTCTAA GCAAGATTGA
1C20_gi|16905111|      VKR-----ED ---------- ---------- ---------- -IFCTS---- KV--------
1C21_gi|126090770|     VRR-----ED ---------- ---------- ---------- -IFYTS---- KV--------
1C22_gi|38603389|      VKR-----ED ---------- ---------- ---------- -LFVTT---- KL--------
1C23_gi|62856987|      VKR-----ED ---------- ---------- ---------- -IFCTS---- KL--------
1C24_gi|84993586|      VKR-----ED ---------- ---------- ---------- -MFITT---- KL--------
1C25_gi|15216337|      VKR-----ED ---------- ---------- ---------- -IFYTS---- KL--------
1D1_gi|5174695|        VRR-----ED ---------- ---------- ---------- -IFYCG---- KL--------
1D2_gi|398962|         VKR-----EE ---------- ---------- ---------- -IFYCG---- KL--------
1D3_gi|5689216|        VRR-----ED ---------- ---------- ---------- -IFYCG---- KL--------
1E1_gi|1698718|        VKR-----ED ---------- ---------- ---------- -LFVVS---- KL--------
1E2_gi|269849539|      VRR-----ED ---------- ---------- ---------- -LFIAT---- KL--------
1G1_gi|17550248|       VKR-----EE ---------- ---------- ---------- -LFITT---- KA--------
2A1_gi|134153|         VKR-----EE ---------- ---------- ---------- -LFITT---- KI--------
2A2_gi|1835701|        VKR-----ED ---------- ---------- ---------- -LFITT---- KL--------
2B1_gi|401428|         VKR-----ED ---------- ---------- ---------- -LFLTS---- KL--------
2B2_gi|1351442|        VKR-----ED ---------- ---------- ---------- -LVVVS---- KL--------
2B3_gi|2492803|        VKR-----KD ---------- ---------- ---------- -LFITS---- KL--------
2B4_gi|1912051|        VKR-----EE ---------- ---------- ---------- -LFITS---- KL--------
2B5_gi|3289019|        VKR-----EE ---------- ---------- ---------- -IFLTS---- KL--------
2B6_gi|6321895|        IKR-----KE ---------- ---------- ---------- -KFYDSQE-- QI--------
2B7_gi|1912049|        VKR-----EE ---------- ---------- ---------- -LFITS---- KL--------
2B8_gi|4103055|        VAR-----DE ---------- ---------- ---------- -LFVVS---- KL--------
2C1_gi|1332539|        VKR-----ED ---------- ---------- ---------- -LFIVT---- KL--------
2D1_gi|7407095|        VKR-----EE ---------- ---------- ---------- -LFIVS---- KL--------
2E1_gi|4753912|        IKR-----ED ---------- ---------- ---------- -VFLTT---- KL--------
2E2_gi|18479021|       VKR-----EE ---------- ---------- ---------- -LFVTT---- KL--------
2E3_gi|5052610|        IKR-----ED ---------- ---------- ---------- -IHITT---- KL--------
3A1_gi|121087|         VPR-----EE ---------- ---------- ---------- -IFVTT---- KL--------
3A2_gi|6320576|        VPR-----EE ---------- ---------- ---------- -IFITT---- KL--------
3B1_gi|1142698|        VKR-----ED ---------- ---------- ---------- -LFITS---- KL--------
3B2_gi|60458781|       VKR-----ED ---------- ---------- ---------- -LFITS---- KL--------
3B3_gi|60458785|       VKR-----ED ---------- ---------- ---------- -LFITS---- KV--------
3C1_gi|536474|         IKR-----ED ---------- ---------- ---------- -LFITT---- KV--------
3C2_gi|74626610|       VPR-----SK ---------- ---------- ---------- -LFITS---- KV--------
3C3_gi|38423524|       VPR-----ED ---------- ---------- ---------- -IWVTT---- KY--------
3D1_gi|31321885|       VKR-----ED ---------- ---------- ---------- -LFVVS---- KC--------
3E1_gi|22207641|       VKR-----ED ---------- ---------- ---------- -IFVCT---- KV--------
3F1_gi|81625481|       FRR-----ED ---------- ---------- ---------- -LFIVS---- KV--------
3F2_gi|13638516|       VPR-----HE ---------- ---------- ---------- -LYITT---- KI--------
3F3_gi|81635765|       LAR-----DE ---------- ---------- ---------- -LCITT---- KV--------
4A1_gi|112837|         VSR-----QD ---------- ---------- ---------- -LFVTS---- KL--------
```

```
4A2_gi|75220959|       VTR-----DE ---------- ---------- ---------- -LFVTS---- KL--------
4A3_gi|1215788|        VTR-----EE ---------- ---------- ---------- -LFVTS---- KL--------
4A4_gi|1514979|        VTR-----ED ---------- ---------- ---------- -LFVTS---- KL--------
4B1_gi|2792155|        KSR-----DE ---------- ---------- ---------- -VFITS---- KP--------
4B2_gi|6478210|        KSR-----DE ---------- ---------- ---------- -LFITS---- KL--------
4B3_gi|6478204|        KSR-----DE ---------- ---------- ---------- -LFITS---- KL--------
4B4_gi|2792295|        KSR-----DE ---------- ---------- ---------- -LFITT---- KL--------
4B5_gi|112807104|      ASR-----ED ---------- ---------- ---------- -LFVTS---- KV--------
4B6_gi|112807098|      ASR-----AD ---------- ---------- ---------- -PFITS---- KL--------
4B7_gi|112807100|      ASR-----DD ---------- ---------- ---------- -LFITS---- KL--------
4B8_gi|112807102|      ASR-----DD ---------- ---------- ---------- -LFITS---- KL--------
4C1_gi|113595|         IDR-----KD ---------- ---------- ---------- -LFVTS---- KI--------
4C2_gi|167113|         IDR-----KD ---------- ---------- ---------- -LFVTS---- KL--------
4C3_gi|75221432|       IDR-----KD ---------- ---------- ---------- -LFVTS---- KI--------
4C4_gi|4539944|        INR-----KA ---------- ---------- ---------- -LFVTS---- KV--------
4C5_gi|13160397|       VKR-----ED ---------- ---------- ---------- -LFITS---- KL--------
4C6_gi|13160399|       VKR-----ED ---------- ---------- ---------- -LFITS---- KL--------
4C8_gi|111182163|      VKR-----EE ---------- ---------- ---------- -LFITS---- KL--------
4C9_gi|111182165|      VKR-----ED ---------- ---------- ---------- -LFITS---- KL--------
4C10_gi|111182167|     VKR-----EE ---------- ---------- ---------- -MFITS---- KL--------
4C11_gi|111182169|     VKR-----EK ---------- ---------- ---------- -LFITS---- KI--------
5A1_gi|408360251|      VPR-----ED ---------- ---------- ---------- -VFITT---- KL--------
5A2_gi|11127591|       VPR-----EE ---------- ---------- ---------- -LFVTT---- KL--------
5B1_gi|2506173|        VDR-----AE ---------- ---------- ---------- -MFVTT---- KL--------
5C1_gi|144969|         IAR-----DD ---------- ---------- ---------- -LFITT---- KL--------
5C2_gi|3916039|        VNR-----EE ---------- ---------- ---------- -LFITT---- KL--------
5D1_gi|112735|         VDR-----DE ---------- ---------- ---------- -LIVAS---- KI--------
5E1_gi|5354195|        IAR-----DK ---------- ---------- ---------- -LRIVS---- KL--------
5F1_gi|82504416|       IAR-----HE ---------- ---------- ---------- -LFVTT---- KL--------
5G1_gi|16080393|       VAR-----EE ---------- ---------- ---------- -LFITS---- KV--------
5G2_gi|16079957|       ISR-----ED ---------- ---------- ---------- -LFITS---- KV--------
6A1_gi|18202524|       WRR-----SS ---------- ---------- ---------- -LVITT---- KI--------
6A2_gi|499328|         WRR-----SS ---------- ---------- ---------- -LVITT---- KI--------
6A3_gi|2135947|        WRR-----SS ---------- ---------- ---------- -LVITT---- KL--------
6A4_gi|975314|         WRR-----SS ---------- ---------- ---------- -LVITT---- KI--------
6A5_gi|2827466|        WRR-----SS ---------- ---------- ---------- -LVITT---- KI--------
6A6_gi|7914984|        WRR-----SS ---------- ---------- ---------- -LVITT---- KI--------
6A7_gi|5019764|        WRR-----SS ---------- ---------- ---------- -LVITT---- KL--------
6A8_gi|148747467|      WRR-----SS ---------- ---------- ---------- -LVITT---- KL--------
6A9_gi|24648619|       VERIHVKVDD NEMLHKISST LDLLSYILQQ TLVLEERTTL IILLKEHHDF EI--------
6A10a_gi|726465|       WRR-----SS ---------- ---------- ---------- -LVITT---- KL--------
6A10b_gi|5019766|      WRR-----SS ---------- ---------- ---------- -LVITT---- KL--------
6A11_gi|5922729|       WRR-----SS ---------- ---------- ---------- -YVVTT---- KI--------
6A13_gi|52001073|      WRR-----SS ---------- ---------- ---------- -LVITT---- KL--------
6A14_gi|226823214|     WRR-----SS ---------- ---------- ---------- -YVITT---- KI--------
6B1_gi|902000|         WKR-----TA ---------- ---------- ---------- -YVITT---- KV--------
6C1_gi|1063415|        WRR-----SD ---------- ---------- ---------- -IVIST---- KI--------
6C2_gi|2832783|        WKR-----SD ---------- ---------- ---------- -LVVST---- KI--------
7A1_gi|39932720|       GCK------- ---------- ---------- ---------- -VKIAT---- KA--------
7A2_gi|41327764|       DCR-----VK ---------- ---------- ---------- -IATKA---- NP--------
7A3_gi|41152114|       DCR------- ---------- ---------- ---------- -VKIDT---- KA--------
7A4_gi|6815049|        TVK------- ---------- ---------- ---------- ---IAT---- KA--------
8A1_gi|24638123|       --------NK ---------- ---------- ---------- -VFLSVKGGL DF--------
8A2_gi|74627022|       --------DK ---------- ---------- ---------- -VFLSV---- KG--------
9A1_gi|146345520|      -NR-----DE ---------- ---------- ---------- -LVLAT---- K---------
9A2_gi|6093525|        QNR-----DE ---------- ---------- ---------- -IVLST---- K---------
9A3_gi|2492798|        -NR-----DQ ---------- ---------- ---------- -MVVAT---- K---------
9B1_gi|6323998|        KLR-----DQ ---------- ---------- ---------- -IVIAT---- KF--------
9B2_gi|6319951|        IAT------- ---------- ---------- ---------- -KFIKSDKKY KA--------
9B3_gi|6319958|        IAT------- ---------- ---------- ---------- -KFTGDYKKY EV--------
9B4_gi|6322615|        --------AN ---------- ---------- ---------- ---------- ----------
9C1_gi|2459734|        RDR-----ER ---------- ---------- ---------- -FTIAS---- KI--------
10A1_gi|4731595|       ASRGVE--KE ---------- ---------- ---------- -VWVLA---- KG--------
10A2_gi|3256056|       VEK-----E- ---------- ---------- ---------- -VWVLA---- KG--------
11A1_gi|1176985|       FNR-----ED ---------- ---------- ---------- -VVIAT---- KA--------
11B1_gi|3123233|       -KR-----DQ ---------- ---------- ---------- -VILAT---- KT--------
11B2_gi|3123121|       LPR-----EQ ---------- ---------- ---------- -VVVET---- KC--------
11B3_gi|85062654|      PQR-----DQ ---------- ---------- ---------- -VTLLT---- KV--------
11C1_gi|81787577|      GRR-----QD ---------- ---------- ---------- -IILAT---- KV--------
12A1_gi|5305791|       -RR-----DD ---------- ---------- ---------- -VVLAT---- KV--------
12B1_gi|2599278|       GRR-----ED ---------- ---------- ---------- -TVLAT---- KV--------
12C1_gi|5921163|       ARR-----EQ ---------- ---------- ---------- -VVLAT---- KV--------
13A1_gi|1351673|       -RR-----KE ---------- ---------- ---------- -IFLAT---- KF--------
13B1_gi|9106797|       LVR-----YP ---------- ---------- ---------- -GNSTN---- PS--------
13C1_gi|81555851|      ASK-----FG ---------- ---------- ---------- -IYYAD---- ----------
14A1_gi|882530|        AYR-----DE ---------- ---------- ---------- -LIIST---- KA--------
15A1_gi|37196700|      STK-----VG ---------- ---------- ---------- -RLMTNERAG RT--------
all2316_aldo/keto      --------SQ ---------- ---------- ---------- -VVVAT---- KG--------
```

```
                      481
1A1_gi|5174391|       ---------- ---------- WN-------- -------TKH HP-------- ----------
1A2_gi|1703236|       ---------- ---------- WN-------- -------TKH HP-------- ----------
1A3_gi|1703237|       ---------- ---------- WN-------- -------TKH HP-------- ----------
1A4_gi|10946870|      ---------- ---------- WN-------- -------TKH HP-------- ----------
1B1_gi|4502049|       ---------- ---------- WC-------- -------TYH EK-------- ----------
1B2_gi|1703235|       ---------- ---------- WC-------- -------TSH DK-------- ----------
1B3_gi|1351911|       ---------- ---------- WC-------- -------TFH DK-------- ----------
1B4_gi|6978491|       ---------- ---------- WC-------- -------TFH DQ-------- ----------
1B5_gi|113594|        ---------- ---------- WC-------- -------TYH DK-------- ----------
1B6_gi|584742|        ---------- ---------- WC-------- -------TDH EK-------- ----------
1B7_gi|231525|        ---------- ---------- WA-------- -------TFF EK-------- ----------
1B8_gi|6679791|       ---------- ---------- WP-------- -------TCF EK-------- ----------
1B9_gi|2114406|       ---------- ---------- WP-------- -------TCF ER-------- ----------
1B10_gi|223468663|    ---------- ---------- WP-------- -------TFF ER-------- ----------
1B12_gi|14330324|     ---------- ---------- WN-------- -------TFH EK-------- ----------
1B13_gi|15864567|     ---------- ---------- WP-------- -------TCF EK-------- ----------
1B14_gi|148540194|    ---------- ---------- WS-------- -------TFF EK-------- ----------
1B15_gi|51094822|     ---------- ----HMAHLV WP-------- -------TFF ER-------- ----------
1C1_gi|5453543|       ---------- ---------- WC-------- -------NSH RP-------- ----------
1C2_gi|4503285|       ---------- ---------- WS-------- -------NSH RP-------- ----------
1C3_gi|24497583|      ---------- ---------- WS-------- -------TFH RP-------- ----------
1C4_gi|308153631|     ---------- ---------- WC-------- -------TFF QP-------- ----------
1C5_gi|1352733|       ---------- ---------- WC-------- -------TFH RP-------- ----------
1C6_gi|13487925|      ---------- ---------- WC-------- -------TFH RP-------- ----------
1C7_gi|129896|        ---------- ---------- WC-------- -------NSL QP-------- ----------
1C8_gi|1709623|       ---------- ---------- WS-------- -------TSH RP-------- ----------
1C9_gi|118634|        ---------- ---------- WS-------- -------TFH RP-------- ----------
1C10a_gi|1345830|     ---------- ---------- WC-------- -------TYF SP-------- ----------
1C10b_gi|1706132|     ---------- ---------- WC-------- -------TYF SP-------- ----------
1C11_gi|1669605|      ---------- ---------- WS-------- -------TSL RP-------- ----------
1C12_gi|85719330|     ---------- ---------- WC-------- -------GCF RP-------- ----------
1C13_gi|171846276|    ---------- ---------- WC-------- -------TCF RP-------- ----------
1C14_gi|19527294|     ---------- ---------- WS-------- -------TFH RP-------- ----------
1C15_gi|741804|       ---------- ---------- WI-------- -------TFL RP-------- ----------
1C16_gi|741803|       ---------- ---------- WC-------- -------TCF RP-------- ----------
1C17_gi|741805|       ---------- ---------- WC-------- -------TCF RP-------- ----------
1C18_gi|1654715|      AGATGGCACT GTGAAAAGGG AAGATATATT CTATACTTCG AAGCTTTGGT CAACTTCCCA
1C20_gi|16905111|     ---------- ---------- WQ-------- -------TFH RP-------- ----------
1C21_gi|126090770|    ---------- ---------- WC-------- -------TSL HP-------- ----------
1C22_gi|38603389|     ---------- ---------- WC-------- -------TCF RP-------- ----------
1C23_gi|62856987|     ---------- ---------- WA-------- -------TSL RL-------- ----------
1C24_gi|84993586|     ---------- ---------- WC-------- -------SCF RT-------- ----------
1C25_gi|15216337|     ---------- ---------- WC-------- -------TFF RP-------- ----------
1D1_gi|5174695|       ---------- ---------- WA-------- -------TNH VP-------- ----------
1D2_gi|398962|        ---------- ---------- WS-------- -------TDH DP-------- ----------
1D3_gi|5689216|       ---------- ---------- WA-------- -------TNH DP-------- ----------
1E1_gi|1698718|       ---------- ---------- WC-------- -------TCH KK-------- ----------
1E2_gi|269849539|     ---------- ---------- WC-------- -------TCH KK-------- ----------
1G1_gi|17550248|      ---------- ---------- WT-------- -------HEL AP-------- ----------
2A1_gi|134153|        ---------- ---------- WN-------- -------SDH G--------- ----------
2A2_gi|1835701|       ---------- ---------- WN-------- -------SDH G--------- ----------
2B1_gi|401428|        ---------- ---------- WN-------- -------NYH HP-------- ----------
2B2_gi|1351442|       ---------- ---------- WN-------- -------SFH HP-------- ----------
2B3_gi|2492803|       ---------- ---------- WN-------- -------NFH AK-------- ----------
2B4_gi|1912051|       ---------- ---------- WN-------- -------NFH DP-------- ----------
2B5_gi|3289019|       ---------- ---------- WN-------- -------NYH DP-------- ----------
2B6_gi|6321895|       -------LEL QEGDVDDSLI WN---VPMAS LSTNSFLASA KP-------- ----------
2B7_gi|1912049|       ---------- ---------- WN-------- -------NFH DP-------- ----------
2B8_gi|4103055|       ---------- ---------- WN-------- -------SFH DP-------- ----------
2C1_gi|1332539|       ---------- ---------- WN-------- -------TFH SK-------- ----------
2D1_gi|7407095|       ---------- ---------- WN-------- -------SFH DG-------- ----------
2E1_gi|4753912|       ---------- ---------- WN-------- -------THH KR-------- ----------
2E2_gi|18479021|      ---------- ---------- WN-------- -------DKH GR-------- ----------
2E3_gi|5052610|       ---------- ---------- WC-------- -------HFH EP-------- ----------
3A1_gi|121087|        ---------- ---------- WC-------- -------TQH HE-------- ----------
3A2_gi|6320576|       ---------- ---------- WG-------- ------TEQR DP-------- ----------
3B1_gi|1142698|       ---------- ---------- WN-------- -------NSH RP-------- ----------
3B2_gi|60458781|      ---------- ---------- WN-------- -------NRH AP-------- ----------
3B3_gi|60458785|      ---------- ---------- WN-------- -------NRH AP-------- ----------
3C1_gi|536474|        ---------- ---------- WP-------- -------VLW ---------- ----------
3C2_gi|74626610|      ---------- ---------- ---------- --------MH NV-------- ----------
3C3_gi|38423524|      ---------- -----SP--G WG-------- -------SIK AY-------- ----------
3D1_gi|31321885|      ---------- ---------- WA-------- -------TYT TR-------- ----------
3E1_gi|22207641|      ---------- ---------- WN-------- -------HLH RY-------- ----------
3F1_gi|81625481|      ---------- ---------- WP-------- -------THL RR-------- ----------
3F2_gi|13638516|      ---------- ---------- WI-------- -------ENL SK-------- ----------
3F3_gi|81635765|      ---------- ---------- HP-------- -------DNY SE-------- ----------
4A1_gi|112837|        ---------- ---------- WV-------- -------TEN HP-------- ----------
```

```
4A2_gi|75220959|     ---------- ---------- WV-------- -------TEN HP-------- ----------
4A3_gi|1215788|      ---------- ---------- WV-------- -------TEN HP-------- ----------
4A4_gi|1514979|      ---------- ---------- WV-------- -------TEN HP-------- ----------
4B1_gi|2792155|      ---------- ---------- WN-------- -------TDA HH-------- ----------
4B2_gi|6478210|      ---------- ---------- WC-------- -------ADA HA-------- ----------
4B3_gi|6478204|      ---------- ---------- WC-------- -------ADA HA-------- ----------
4B4_gi|2792295|      ---------- ---------- WA-------- -------SFA EK-------- ----------
4B5_gi|112807104|    ---------- ---------- WC-------- -------ADA HR-------- ----------
4B6_gi|112807098|    ---------- ---------- WC-------- -------SDA HR-------- ----------
4B7_gi|112807100|    ---------- ---------- WC-------- -------SDA HG-------- ----------
4B8_gi|112807102|    ---------- ---------- WC-------- -------SDA HR-------- ----------
4C1_gi|113595|       ---------- ---------- WC-------- -------TNL AP-------- ----------
4C2_gi|167113|       ---------- ---------- WC-------- -------TDL VP-------- ----------
4C3_gi|75221432|     ---------- ---------- WR-------- -------TNL AP-------- ----------
4C4_gi|4539944|      ---------- ---------- WC-------- -------EDL SP-------- ----------
4C5_gi|13160397|     ---------- ---------- WS-------- -------TDH AP-------- ----------
4C6_gi|13160399|     ---------- ---------- WS-------- -------TDH AP-------- ----------
4C8_gi|111182163|    ---------- ---------- WS-------- -------NDH LP-------- ----------
4C9_gi|111182165|    ---------- ---------- WC-------- -------TDH DP-------- ----------
4C10_gi|111182167|   ---------- ---------- WC-------- -------TYH DP-------- ----------
4C11_gi|111182169|   ---------- ---------- WL-------- -------TDL DP-------- ----------
5A1_gi|408360251|    ---------- ---------- WN-------- -------TEQ GY-------- ----------
5A2_gi|11127591|     ---------- ---------- WN-------- -------SDQ GY-------- ----------
5B1_gi|2506173|      ---------- ---------- FN-------- -------CDY GY-------- ----------
5C1_gi|144969|       ---------- ---------- WN-------- -------DRH DG-------- ----------
5C2_gi|3916039|      ---------- ---------- WN-------- -------DDH KR-------- ----------
5D1_gi|112735|       ---------- ---------- PG-------- -------RQH GR-------- ----------
5E1_gi|5354195|      ---------- ---------- PG-------- -------RHH HF-------- ----------
5F1_gi|82504416|     ---------- ---------- WL-------- -------QDT HY-------- ----------
5G1_gi|16080393|     ---------- ---------- WN-------- -------EDQ GY-------- ----------
5G2_gi|16079957|     ---------- ---------- WN-------- -------ADL GY-------- ----------
6A1_gi|18202524|     ---------- ---------F WG-------- -GKAETERGL SR-------- ----------
6A2_gi|499328|       ---------- ---------F WG-------- -GKAETERGL SR-------- ----------
6A3_gi|2135947|      ---------- ---------Y WG-------- -GKAETERGL SR-------- ----------
6A4_gi|975314|       ---------- ---------F WG-------- -GKAETERGL SR-------- ----------
6A5_gi|2827466|      ---------- ---------F WG-------- -GKAETERGL SR-------- ----------
6A6_gi|7914984|      ---------- ---------F WG-------- -GKAETERGL SR-------- ----------
6A7_gi|5019764|      ---------- ---------Y WG-------- -GKAETERGL SR-------- ----------
6A8_gi|148747467|    ---------- ---------Y WG-------- -GKAETERGL SR-------- ----------
6A9_gi|24648619|     ---------- --------TV WKLIDMTHNP YFTEKIFTTL SSYNFALLLH EKLLNPNATE
6A10a_gi|726465|     ---------- ---------Y WG-------- -GKAETERGL SR-------- ----------
6A10b_gi|5019766|    ---------- ---------Y WG-------- -GKAETERGL SR-------- ----------
6A11_gi|5922729|     ---------- ---------Y WG-------- -GQAETERGL SR-------- ----------
6A13_gi|52001073|    ---------- ---------Y WG-------- -GKAETERGL SR-------- ----------
6A14_gi|226823214|   ---------- ---------F WG-------- -GQAETERGL SR-------- ----------
6B1_gi|902000|       ---------- ---------Y WS-------- --TKSEERGL SR-------- ----------
6C1_gi|1063415|      ---------- ---------F WG-------- -GPGPNDKGL SR-------- ----------
6C2_gi|2832783|      ---------- ---------F WG-------- -GPGPNDKGL SR-------- ----------
7A1_gi|39932720|     ---------- -------APM FG-------- -------KTL KP-------- ----------
7A2_gi|41327764|     ---------- ---------- WD-------- ------GKSL KP-------- ----------
7A3_gi|41152114|     ---------- -------IPL FG-------- -------NSL KP-------- ----------
7A4_gi|6815049|      ---------- --------NP WD-------- ------GKSL KP-------- ----------
8A1_gi|24638123|     ---------- --------KT LV-------- -------PDG NP-------- ----------
8A2_gi|74627022|     ---------- ----GTDFKT LA-------- -------PHG DP-------- ----------
9A1_gi|146345520|    ---------- ---------- YTMSYRLTGP EKIKSNFQGS HS-------- ----------
9A2_gi|6093525|      ---------- ---------- YTMGYTMFGP QKIKSNYQGN HA-------- ----------
9A3_gi|2492798|      ---------- ---------- YSLVYKRGAS FEEIPQKTQY VG-------- ----------
9B1_gi|6323998|      ----TGDYKK YEVGGGKSAN YC-------- -------GNH KR-------- ----------
9B2_gi|6319951|      ---------- ---GESNTAN YC-------- -------GNH KR-------- ----------
9B3_gi|6319958|      ---------- ---GGGKSAN YC-------- -------GNH KH-------- ----------
9B4_gi|6322615|      ---------- ---------- FC-------- -------GNH KR-------- ----------
9C1_gi|2459734|      ---------- ---------Y WQ-------I RDGDPNSRGT NR-------- ----------
10A1_gi|4731595|     ---------- ---------A HT-------- -------PEC LP-------- ----------
10A2_gi|3256056|     ---------- ---------A HT-------- -------PEC LP-------- ----------
11A1_gi|1176985|     ---------- AHRKQGN--D FV-------- -------FDN SP-------- ----------
11B1_gi|3123233|     ---------- -------ALD WK-------- --NNQLFRHA NR-------- ----------
11B2_gi|3123121|     ---------- -------GIV WERKGSLFNK VGDRQLYKNL SP-------- ----------
11B3_gi|85062654|    ---------- ---------- FA-------- -------NHL HH-------- ----------
11C1_gi|81787577|    ---------- GNRFEQGKEG WW-------- -------WDP SK-------- ----------
12A1_gi|5305791|     ---------- --GEPMSDRV ND-------- -------RGL SA-------- ----------
12B1_gi|2599278|     ---------- --GGEMSERV ND-------- -------SGL SA-------- ----------
12C1_gi|5921163|     ---------- ---------- -------GDP MGSGPNDHGL SV-------- ----------
13A1_gi|1351673|     ---------- GYEKNPETGE LS-------- -------LNN EP-------- ----------
13B1_gi|9106797|     ---------- ---------- WP-------- -------VIG DP-------- ----------
13C1_gi|81555851|    ---------- ------PNDK YA-------- ----TMFLDS SP-------- ----------
14A1_gi|882530|      ---------- ------GYDM WP-------- ---GPYGSGG SR-------- ----------
15A1_gi|37196700|    -------LPP APPKNPLDSG WH-------N GLNFREVFDY SY-------- ----------
all2316_aldo/keto    ---------- --GLMRPNES WT-------- -------RNG NP-------- ----------
```

```
                          541
1A1_gi|5174391|      ---------- ---EDVE--- ---------- ---------- ---------- ----------
1A2_gi|1703236|      ---------- ---EDVE--- ---------- ---------- ---------- ----------
1A3_gi|1703237|      ---------- ---EDVE--- ---------- ---------- ---------- ----------
1A4_gi|10946870|     ---------- ---EDVE--- ---------- ---------- ---------- ----------
1B1_gi|4502049|      ---------- ---GLVK--- ---------- ---------- ---------- ----------
1B2_gi|1703235|      ---------- ---SLVK--- ---------- ---------- ---------- ----------
1B3_gi|1351911|      ---------- ---SMVK--- ---------- ---------- ---------- ----------
1B4_gi|6978491|      ---------- ---SMVK--- ---------- ---------- ---------- ----------
1B5_gi|113594|       ---------- ---DLVK--- ---------- ---------- ---------- ----------
1B6_gi|584742|       ---------- ---NLVK--- ---------- ---------- ---------- ----------
1B7_gi|231525|       ---------- ---SLVK--- ---------- ---------- ---------- ----------
1B8_gi|6679791|      ---------- ---KLLK--- ---------- ---------- ---------- ----------
1B9_gi|2114406|      ---------- ---KLLK--- ---------- ---------- ---------- ----------
1B10_gi|223468663|   ---------- ---PLVR--- ---------- ---------- ---------- ----------
1B12_gi|14330324|    ---------- ---SLVK--- ---------- ---------- ---------- ----------
1B13_gi|15864567|    ---------- ---KLLK--- ---------- ---------- ---------- ----------
1B14_gi|148540194|   ---------- ---SLMK--- ---------- ---------- ---------- ----------
1B15_gi|51094822|    ---------- ---PLVR--- ---------- ---------- ---------- ----------
1C1_gi|5453543|      ---------- ---ELVR--- ---------- ---------- ---------- ----------
1C2_gi|4503285|      ---------- ---ELVR--- ---------- ---------- ---------- ----------
1C3_gi|24497583|     ---------- ---ELVR--- ---------- ---------- ---------- ----------
1C4_gi|308153631|    ---------- ---QMVQ--- ---------- ---------- ---------- ----------
1C5_gi|1352733|      ---------- ---ELVR--- ---------- ---------- ---------- ----------
1C6_gi|13487925|     ---------- ---ELVR--- ---------- ---------- ---------- ----------
1C7_gi|129896|       ---------- ---ELVR--- ---------- ---------- ---------- ----------
1C8_gi|1709623|      ---------- ---ELVR--- ---------- ---------- ---------- ----------
1C9_gi|118634|       ---------- ---ELVR--- ---------- ---------- ---------- ----------
1C10a_gi|1345830|    ---------- ---EMVR--- ---------- ---------- ---------- ----------
1C10b_gi|1706132|    ---------- ---DMVR--- ---------- ---------- ---------- ----------
1C11_gi|1669605|     ---------- ---ELVR--- ---------- ---------- ---------- ----------
1C12_gi|85719330|    ---------- ---ELVK--- ---------- ---------- ---------- ----------
1C13_gi|171846276|   ---------- ---ELVK--- ---------- ---------- ---------- ----------
1C14_gi|19527294|    ---------- ---ELVR--- ---------- ---------- ---------- ----------
1C15_gi|741804|      ---------- ---ELVR--- ---------- ---------- ---------- ----------
1C16_gi|741803|      ---------- ---ELVK--- ---------- ---------- ---------- ----------
1C17_gi|741805|      ---------- ---ELVK--- ---------- ---------- ---------- ----------
1C18_gi|1654715|     TCGTCCAGAG TTGGTCA--- ---------- ---------- ---------- ----------
1C20_gi|16905111|    ---------- ---ELVQ--- ---------- ---------- ---------- ----------
1C21_gi|126090770|   ---------- ---ELVR--- ---------- ---------- ---------- ----------
1C22_gi|38603389|    ---------- ---ELVK--- ---------- ---------- ---------- ----------
1C23_gi|62856987|    ---------- ---QLVR--- ---------- ---------- ---------- ----------
1C24_gi|84993586|    ---------- ---EMVR--- ---------- ---------- ---------- ----------
1C25_gi|15216337|    ---------- ---QLVQ--- ---------- ---------- ---------- ----------
1D1_gi|5174695|      ---------- ---EMVR--- ---------- ---------- ---------- ----------
1D2_gi|398962|       ---------- ---EMVR--- ---------- ---------- ---------- ----------
1D3_gi|5689216|      ---------- ---VMVR--- ---------- ---------- ---------- ----------
1E1_gi|1698718|      ---------- ---SLVK--- ---------- ---------- ---------- ----------
1E2_gi|269849539|    ---------- ---SLVE--- ---------- ---------- ---------- ----------
1G1_gi|17550248|     ---------- ---GKLE--- ---------- ---------- ---------- ----------
2A1_gi|134153|       ---------- ----HVV--- ---------- ---------- ---------- ----------
2A2_gi|1835701|      ---------- ----HVI--- ---------- ---------- ---------- ----------
2B1_gi|401428|       ---------- ---DNVE--- ---------- ---------- ---------- ----------
2B2_gi|1351442|      ---------- ---DNVP--- ---------- ---------- ---------- ----------
2B3_gi|2492803|      ---------- ---ENVK--- ---------- ---------- ---------- ----------
2B4_gi|1912051|      ---------- ---KNVE--- ---------- ---------- ---------- ----------
2B5_gi|3289019|      ---------- ---KNVE--- ---------- ---------- ---------- ----------
2B6_gi|6321895|      ---------- ---DDMN--- ---------- ---------- ---------- ----------
2B7_gi|1912049|      ---------- ---KNVE--- ---------- ---------- ---------- ----------
2B8_gi|4103055|      ---------- ---KNVE--- ---------- ---------- ---------- ----------
2C1_gi|1332539|      ---------- ---KHVR--- ---------- ---------- ---------- ----------
2D1_gi|7407095|      ---------- ---DRVE--- ---------- ---------- ---------- ----------
2E1_gi|4753912|      ---------- ---EQVA--- ---------- ---------- ---------- ----------
2E2_gi|18479021|     ---------- ---HQVV--- ---------- ---------- ---------- ----------
2E3_gi|5052610|      ---------- ---KRVE--- ---------- ---------- ---------- ----------
3A1_gi|121087|       ---------- ---PEV---- ---------- ---------- ---------- ----------
3A2_gi|6320576|      ---------- ---------- ---------- ---------- ---------- ----------
3B1_gi|1142698|      ---------- ---EQVE--- ---------- ---------- ---------- ----------
3B2_gi|60458781|     ---------- ---EHVE--- ---------- ---------- ---------- ----------
3B3_gi|60458785|     ---------- ---EHVE--- ---------- ---------- ---------- ----------
3C1_gi|536474|       ---------- ---DEVD--- ---------- ---------- ---------- ----------
3C2_gi|74626610|     ---------- ---DNIP--- ---------- ---------- ---------- ----------
3C3_gi|38423524|     ---------- ---SKSP--- ---------- ---------- ---------- ----------
3D1_gi|31321885|     ---------- -----CE--- ---------- ---------- ---------- ----------
3E1_gi|22207641|     ---------- ---EDVL--- ---------- ---------- ---------- ----------
3F1_gi|81625481|     ---------- ---DDLL--- ---------- ---------- ---------- ----------
3F2_gi|13638516|     ---------- ---DKLI--- ---------- ---------- ---------- ----------
3F3_gi|81635765|     ---------- ---EAFL--- ---------- ---------- ---------- ----------
4A1_gi|112837|       ---------- ---HLVL--- ---------- ---------- ---------- ----------
```

```
4A2_gi|75220959|      ---------- ---HLVI--- ---------- ---------- ---------- ----------
4A3_gi|1215788|       ---------- ---HLVI--- ---------- ---------- ---------- ----------
4A4_gi|1514979|       ---------- ---HLVV--- ---------- ---------- ---------- ----------
4B1_gi|2792155|       ---------- ---DLIV--- ---------- ---------- ---------- ----------
4B2_gi|6478210|       ---------- ---DLVL--- ---------- ---------- ---------- ----------
4B3_gi|6478204|       ---------- ---DLVL--- ---------- ---------- ---------- ----------
4B4_gi|2792295|       ---------- ---DLVL--- ---------- ---------- ---------- ----------
4B5_gi|112807104|     ---------- ---DRVL--- ---------- ---------- ---------- ----------
4B6_gi|112807098|     ---------- ---DRVL--- ---------- ---------- ---------- ----------
4B7_gi|112807100|     ---------- ---DRVV--- ---------- ---------- ---------- ----------
4B8_gi|112807102|     ---------- ---DRVV--- ---------- ---------- ---------- ----------
4C1_gi|113595|        ---------- ---ERVR--- ---------- ---------- ---------- ----------
4C2_gi|167113|        ---------- ---DRVR--- ---------- ---------- ---------- ----------
4C3_gi|75221432|      ---------- ---ERAR--- ---------- ---------- ---------- ----------
4C4_gi|4539944|       ---------- ---ERVR--- ---------- ---------- ---------- ----------
4C5_gi|13160397|      ---------- ---EDVP--- ---------- ---------- ---------- ----------
4C6_gi|13160399|      ---------- ---EDVP--- ---------- ---------- ---------- ----------
4C8_gi|111182163|     ---------- ---EDVP--- ---------- ---------- ---------- ----------
4C9_gi|111182165|     ---------- ---QDVP--- ---------- ---------- ---------- ----------
4C10_gi|111182167|    ---------- ---QEVP--- ---------- ---------- ---------- ----------
4C11_gi|111182169|    ---------- ---PDVQ--- ---------- ---------- ---------- ----------
5A1_gi|408360251|     ---------- ---ESTL--- ---------- ---------- ---------- ----------
5A2_gi|11127591|      ---------- ---ESTL--- ---------- ---------- ---------- ----------
5B1_gi|2506173|       ---------- ---ERAL--- ---------- ---------- ---------- ----------
5C1_gi|144969|        ---------- ---DEPA--- ---------- ---------- ---------- ----------
5C2_gi|3916039|       ---------- -----PR--- ---------- ---------- ---------- ----------
5D1_gi|112735|        ---------- ---AEAV--- ---------- ---------- ---------- ----------
5E1_gi|5354195|       ---------- ---EEAI--- ---------- ---------- ---------- ----------
5F1_gi|82504416|      ---------- ---EGAK--- ---------- ---------- ---------- ----------
5G1_gi|16080393|      ---------- ---ETTL--- ---------- ---------- ---------- ----------
5G2_gi|16079957|      ---------- ---EETL--- ---------- ---------- ---------- ----------
6A1_gi|18202524|      ---------- ---KHII--- ---------- ---------- ---------- ----------
6A2_gi|499328|        ---------- ---KHII--- ---------- ---------- ---------- ----------
6A3_gi|2135947|       ---------- ---KHII--- ---------- ---------- ---------- ----------
6A4_gi|975314|        ---------- ---KHII--- ---------- ---------- ---------- ----------
6A5_gi|2827466|       ---------- ---KHII--- ---------- ---------- ---------- ----------
6A6_gi|7914984|       ---------- ---KHII--- ---------- ---------- ---------- ----------
6A7_gi|5019764|       ---------- ---KHII--- ---------- ---------- ---------- ----------
6A8_gi|148747467|     ---------- ---KHII--- ---------- ---------- ---------- ----------
6A9_gi|24648619|      PSEEFTEFGL AFFNLMKFLI TRKDGLSFVK LAELNHVLWK KLGPRAPKEI VIQQERVTFE
6A10a_gi|726465|      ---------- ---KHII--- ---------- ---------- ---------- ----------
6A10b_gi|5019766|     ---------- ---KHII--- ---------- ---------- ---------- ----------
6A11_gi|5922729|      ---------- ---KHII--- ---------- ---------- ---------- ----------
6A13_gi|52001073|     ---------- ---KHII--- ---------- ---------- ---------- ----------
6A14_gi|226823214|    ---------- ---KHII--- ---------- ---------- ---------- ----------
6B1_gi|902000|        ---------- ---KHII--- ---------- ---------- ---------- ----------
6C1_gi|1063415|       ---------- ---KHIV--- ---------- ---------- ---------- ----------
6C2_gi|2832783|       ---------- ---KHVV--- ---------- ---------- ---------- ----------
7A1_gi|39932720|      ---------- ---ADVR--- ---------- ---------- ---------- ----------
7A2_gi|41327764|      ---------- ---DSVR--- ---------- ---------- ---------- ----------
7A3_gi|41152114|      ---------- ---DSLR--- ---------- ---------- ---------- ----------
7A4_gi|6815049|       ---------- ---DSVR--- ---------- ---------- ---------- ----------
8A1_gi|24638123|      ---------- ---DFVS--- ---------- ---------- ---------- ----------
8A2_gi|74627022|      ---------- ---ESVT--- ---------- ---------- ---------- ----------
9A1_gi|146345520|     ---------- ---KSLR--- ---------- ---------- ---------- ----------
9A2_gi|6093525|       ---------- ---KSLR--- ---------- ---------- ---------- ----------
9A3_gi|2492798|       ---------- NSLKSMH--- ---------- ---------- ---------- ----------
9B1_gi|6323998|       ---------- ----SLH--- ---------- ---------- ---------- ----------
9B2_gi|6319951|       ---------- ---SLHV--- ---------- ---------- ---------- ----------
9B3_gi|6319958|       ---------- ---SLHV--- ---------- ---------- ---------- ----------
9B4_gi|6322615|       ---------- ---SLHV--- ---------- ---------- ---------- ----------
9C1_gi|2459734|       ---------- ---KNVR--- ---------- ---------- ---------- ----------
10A1_gi|4731595|      ---------- ---DRIE--- ---------- ---------- ---------- ----------
10A2_gi|3256056|      ---------- ---DRVE--- ---------- ---------- ---------- ----------
11A1_gi|1176985|      ---------- ---DFLK--- ---------- ---------- ---------- ----------
11B1_gi|3123233|      ---------- ---ARIV--- ---------- ---------- ---------- ----------
11B2_gi|3123121|      ---------- ---ESIR--- ---------- ---------- ---------- ----------
11B3_gi|85062654|     ---------- ---DQVI--- ---------- ---------- ---------- ----------
11C1_gi|81787577|     ---------- ---AYIK--- ---------- ---------- ---------- ----------
12A1_gi|5305791|      ---------- ---RHVI--- ---------- ---------- ---------- ----------
12B1_gi|2599278|      ---------- ---RHII--- ---------- ---------- ---------- ----------
12C1_gi|5921163|      ---------- ---RNIV--- ---------- ---------- ---------- ----------
13A1_gi|1351673|      ---------- ---DYIE--- ---------- ---------- ---------- ----------
13B1_gi|9106797|      ---------- ---AYLR--- ---------- ---------- ---------- ----------
13C1_gi|81555851|     ---------- ---NRIK--- ---------- ---------- ---------- ----------
14A1_gi|882530|       ---------- ---KYLL--- ---------- ---------- ---------- ----------
15A1_gi|37196700|     ---------- ---DGVM--- ---------- ---------- ---------- ----------
all2316_aldo/keto     ---------- ---EHLR--- ---------- ---------- ---------- ----------
```

```
                   601
1A1_gi|5174391|      ---------- ---------- ---------- ---------- ---------- ----------
1A2_gi|1703236|      ---------- ---------- ---------- ---------- ---------- ----------
1A3_gi|1703237|      ---------- ---------- ---------- ---------- ---------- ----------
1A4_gi|10946870|     ---------- ---------- ---------- ---------- ---------- ----------
1B1_gi|4502049|      ---------- ---------- ---------- ---------- ---------- ----------
1B2_gi|1703235|      ---------- ---------- ---------- ---------- ---------- ----------
1B3_gi|1351911|      ---------- ---------- ---------- ---------- ---------- ----------
1B4_gi|6978491|      ---------- ---------- ---------- ---------- ---------- ----------
1B5_gi|113594|       ---------- ---------- ---------- ---------- ---------- ----------
1B6_gi|584742|       ---------- ---------- ---------- ---------- ---------- ----------
1B7_gi|231525|       ---------- ---------- ---------- ---------- ---------- ----------
1B8_gi|6679791|      ---------- ---------- ---------- ---------- ---------- ----------
1B9_gi|2114406|      ---------- ---------- ---------- ---------- ---------- ----------
1B10_gi|223468663|   ---------- ---------- ---------- ---------- ---------- ----------
1B12_gi|14330324|    ---------- ---------- ---------- ---------- ---------- ----------
1B13_gi|15864567|    ---------- ---------- ---------- ---------- ---------- ----------
1B14_gi|148540194|   ---------- ---------- ---------- ---------- ---------- ----------
1B15_gi|51094822|    ---------- ---------- ---------- ---------- ---------- ----------
1C1_gi|5453543|      ---------- ---------- ---------- ---------- ---------- ----------
1C2_gi|4503285|      ---------- ---------- ---------- ---------- ---------- ----------
1C3_gi|24497583|     ---------- ---------- ---------- ---------- ---------- ----------
1C4_gi|308153631|    ---------- ---------- ---------- ---------- ---------- ----------
1C5_gi|1352733|      ---------- ---------- ---------- ---------- ---------- ----------
1C6_gi|13487925|     ---------- ---------- ---------- ---------- ---------- ----------
1C7_gi|129896|       ---------- ---------- ---------- ---------- ---------- ----------
1C8_gi|1709623|      ---------- ---------- ---------- ---------- ---------- ----------
1C9_gi|118634|       ---------- ---------- ---------- ---------- ---------- ----------
1C10a_gi|1345830|    ---------- ---------- ---------- ---------- ---------- ----------
1C10b_gi|1706132|    ---------- ---------- ---------- ---------- ---------- ----------
1C11_gi|1669605|     ---------- ---------- ---------- ---------- ---------- ----------
1C12_gi|85719330|    ---------- ---------- ---------- ---------- ---------- ----------
1C13_gi|171846276|   ---------- ---------- ---------- ---------- ---------- ----------
1C14_gi|19527294|    ---------- ---------- ---------- ---------- ---------- ----------
1C15_gi|741804|      ---------- ---------- ---------- ---------- ---------- ----------
1C16_gi|741803|      ---------- ---------- ---------- ---------- ---------- ----------
1C17_gi|741805|      ---------- ---------- ---------- ---------- ---------- ----------
1C18_gi|1654715|     ---------- ---------- ---------- ---------- ---------- ----------
1C20_gi|16905111|    ---------- ---------- ---------- ---------- ---------- ----------
1C21_gi|126090770|   ---------- ---------- ---------- ---------- ---------- ----------
1C22_gi|38603389|    ---------- ---------- ---------- ---------- ---------- ----------
1C23_gi|62856987|    ---------- ---------- ---------- ---------- ---------- ----------
1C24_gi|84993586|    ---------- ---------- ---------- ---------- ---------- ----------
1C25_gi|15216337|    ---------- ---------- ---------- ---------- ---------- ----------
1D1_gi|5174695|      ---------- ---------- ---------- ---------- ---------- ----------
1D2_gi|398962|       ---------- ---------- ---------- ---------- ---------- ----------
1D3_gi|5689216|      ---------- ---------- ---------- ---------- ---------- ----------
1E1_gi|1698718|      ---------- ---------- ---------- ---------- ---------- ----------
1E2_gi|269849539|    ---------- ---------- ---------- ---------- ---------- ----------
1G1_gi|17550248|     ---------- ---------- ---------- ---------- ---------- ----------
2A1_gi|134153|       ---------- ---------- ---------- ---------- ---------- ----------
2A2_gi|1835701|      ---------- ---------- ---------- ---------- ---------- ----------
2B1_gi|401428|       ---------- ---------- ---------- ---------- ---------- ----------
2B2_gi|1351442|      ---------- ---------- ---------- ---------- ---------- ----------
2B3_gi|2492803|      ---------- ---------- ---------- ---------- ---------- ----------
2B4_gi|1912051|      ---------- ---------- ---------- ---------- ---------- ----------
2B5_gi|3289019|      ---------- ---------- ---------- ---------- ---------- ----------
2B6_gi|6321895|      ---------- ---------- ---------- ---------- ---------- ----------
2B7_gi|1912049|      ---------- ---------- ---------- ---------- ---------- ----------
2B8_gi|4103055|      ---------- ---------- ---------- ---------- ---------- ----------
2C1_gi|1332539|      ---------- ---------- ---------- ---------- ---------- ----------
2D1_gi|7407095|      ---------- ---------- ---------- ---------- ---------- ----------
2E1_gi|4753912|      ---------- ---------- ---------- ---------- ---------- ----------
2E2_gi|18479021|     ---------- ---------- ---------- ---------- ---------- ----------
2E3_gi|5052610|      ---------- ---------- ---------- ---------- ---------- ----------
3A1_gi|121087|       ---------- ---------- ---------- ---------- ---------- ----------
3A2_gi|6320576|      ---------- ---------- ---------- ---------- ---------- ----------
3B1_gi|1142698|      ---------- ---------- ---------- ---------- ---------- ----------
3B2_gi|60458781|     ---------- ---------- ---------- ---------- ---------- ----------
3B3_gi|60458785|     ---------- ---------- ---------- ---------- ---------- ----------
3C1_gi|536474|       ---------- ---------- ---------- ---------- ---------- ----------
3C2_gi|74626610|     ---------- ---------- ---------- ---------- ---------- ----------
3C3_gi|38423524|     ---------- ---------- ---------- ---------- ---------- ----------
3D1_gi|31321885|     ---------- ---------- ---------- ---------- ---------- ----------
3E1_gi|22207641|     ---------- ---------- ---------- ---------- ---------- ----------
3F1_gi|81625481|     ---------- ---------- ---------- ---------- ---------- ----------
3F2_gi|13638516|     ---------- ---------- ---------- ---------- ---------- ----------
3F3_gi|81635765|     ---------- ---------- ---------- ---------- ---------- ----------
4A1_gi|112837|       ---------- ---------- ---------- ---------- ---------- ----------
```

```
4A2_gi|75220959|      ---------- ---------- ---------- ---------- ---------- ----------
4A3_gi|1215788|       ---------- ---------- ---------- ---------- ---------- ----------
4A4_gi|1514979|       ---------- ---------- ---------- ---------- ---------- ----------
4B1_gi|2792155|       ---------- ---------- ---------- ---------- ---------- ----------
4B2_gi|6478210|       ---------- ---------- ---------- ---------- ---------- ----------
4B3_gi|6478204|       ---------- ---------- ---------- ---------- ---------- ----------
4B4_gi|2792295|       ---------- ---------- ---------- ---------- ---------- ----------
4B5_gi|112807104|     ---------- ---------- ---------- ---------- ---------- ----------
4B6_gi|112807098|     ---------- ---------- ---------- ---------- ---------- ----------
4B7_gi|112807100|     ---------- ---------- ---------- ---------- ---------- ----------
4B8_gi|112807102|     ---------- ---------- ---------- ---------- ---------- ----------
4C1_gi|113595|        ---------- ---------- ---------- ---------- ---------- ----------
4C2_gi|167113|        ---------- ---------- ---------- ---------- ---------- ----------
4C3_gi|75221432|      ---------- ---------- ---------- ---------- ---------- ----------
4C4_gi|4539944|       ---------- ---------- ---------- ---------- ---------- ----------
4C5_gi|13160397|      ---------- ---------- ---------- ---------- ---------- ----------
4C6_gi|13160399|      ---------- ---------- ---------- ---------- ---------- ----------
4C8_gi|111182163|     ---------- ---------- ---------- ---------- ---------- ----------
4C9_gi|111182165|     ---------- ---------- ---------- ---------- ---------- ----------
4C10_gi|111182167|    ---------- ---------- ---------- ---------- ---------- ----------
4C11_gi|111182169|    ---------- ---------- ---------- ---------- ---------- ----------
5A1_gi|408360251|     ---------- ---------- ---------- ---------- ---------- ----------
5A2_gi|11127591|      ---------- ---------- ---------- ---------- ---------- ----------
5B1_gi|2506173|       ---------- ---------- ---------- ---------- ---------- ----------
5C1_gi|144969|        ---------- ---------- ---------- ---------- ---------- ----------
5C2_gi|3916039|       ---------- ---------- ---------- ---------- ---------- ----------
5D1_gi|112735|        ---------- ---------- ---------- ---------- ---------- ----------
5E1_gi|5354195|       ---------- ---------- ---------- ---------- ---------- ----------
5F1_gi|82504416|      ---------- ---------- ---------- ---------- ---------- ----------
5G1_gi|16080393|      ---------- ---------- ---------- ---------- ---------- ----------
5G2_gi|16079957|      ---------- ---------- ---------- ---------- ---------- ----------
6A1_gi|18202524|      ---------- ---------- ---------- ---------- ---------- ----------
6A2_gi|499328|        ---------- ---------- ---------- ---------- ---------- ----------
6A3_gi|2135947|       ---------- ---------- ---------- ---------- ---------- ----------
6A4_gi|975314|        ---------- ---------- ---------- ---------- ---------- ----------
6A5_gi|2827466|       ---------- ---------- ---------- ---------- ---------- ----------
6A6_gi|7914984|       ---------- ---------- ---------- ---------- ---------- ----------
6A7_gi|5019764|       ---------- ---------- ---------- ---------- ---------- ----------
6A8_gi|148747467|     ---------- ---------- ---------- ---------- ---------- ----------
6A9_gi|24648619|      NQLICFHMLP LLFSMKYAQK IADEESKIEL FDAYVVKLLE ISCEQTLRLC YTMRDNFFSA
6A10a_gi|726465|      ---------- ---------- ---------- ---------- ---------- ----------
6A10b_gi|5019766|     ---------- ---------- ---------- ---------- ---------- ----------
6A11_gi|5922729|      ---------- ---------- ---------- ---------- ---------- ----------
6A13_gi|52001073|     ---------- ---------- ---------- ---------- ---------- ----------
6A14_gi|226823214|    ---------- ---------- ---------- ---------- ---------- ----------
6B1_gi|902000|        ---------- ---------- ---------- ---------- ---------- ----------
6C1_gi|1063415|       ---------- ---------- ---------- ---------- ---------- ----------
6C2_gi|2832783|       ---------- ---------- ---------- ---------- ---------- ----------
7A1_gi|39932720|      ---------- ---------- ---------- ---------- ---------- ----------
7A2_gi|41327764|      ---------- ---------- ---------- ---------- ---------- ----------
7A3_gi|41152114|      ---------- ---------- ---------- ---------- ---------- ----------
7A4_gi|6815049|       ---------- ---------- ---------- ---------- ---------- ----------
8A1_gi|24638123|      ---------- ---------- ---------- ---------- ---------- ----------
8A2_gi|74627022|      ---------- ---------- ---------- ---------- ---------- ----------
9A1_gi|146345520|     ---------- ---------- ---------- ---------- ---------- ----------
9A2_gi|6093525|       ---------- ---------- ---------- ---------- ---------- ----------
9A3_gi|2492798|       ---------- ---------- ---------- ---------- ---------- ----------
9B1_gi|6323998|       ---------- ---------- ---------- ---------- ---------- ----------
9B2_gi|6319951|       ---------- ---------- ---------- ---------- ---------- ----------
9B3_gi|6319958|       ---------- ---------- ---------- ---------- ---------- ----------
9B4_gi|6322615|       ---------- ---------- ---------- ---------- ---------- ----------
9C1_gi|2459734|       ---------- ---------- ---------- ---------- ---------- ----------
10A1_gi|4731595|      ---------- ---------- ---------- ---------- ---------- ----------
10A2_gi|3256056|      ---------- ---------- ---------- ---------- ---------- ----------
11A1_gi|1176985|      ---------- ---------- ---------- ---------- ---------- ----------
11B1_gi|3123233|      ---------- ---------- ---------- ---------- ---------- ----------
11B2_gi|3123121|      ---------- ---------- ---------- ---------- ---------- ----------
11B3_gi|85062654|     ---------- ---------- ---------- ---------- ---------- ----------
11C1_gi|81787577|     ---------- ---------- ---------- ---------- ---------- ----------
12A1_gi|5305791|      ---------- ---------- ---------- ---------- ---------- ----------
12B1_gi|2599278|      ---------- ---------- ---------- ---------- ---------- ----------
12C1_gi|5921163|      ---------- ---------- ---------- ---------- ---------- ----------
13A1_gi|1351673|      ---------- ---------- ---------- ---------- ---------- ----------
13B1_gi|9106797|      ---------- ---------- ---------- ---------- ---------- ----------
13C1_gi|81555851|     ---------- ---------- ---------- ---------- ---------- ----------
14A1_gi|882530|       ---------- ---------- ---------- ---------- ---------- ----------
15A1_gi|37196700|     ---------- ---------- ---------- ---------- ---------- ----------
all2316_aldo/keto     ---------- ---------- ---------- ---------- ---------- ----------
```

```
                     661
1A1_gi|5174391|      ---------- ---------- ---------- ---------- ---------- ----------
1A2_gi|1703236|      ---------- ---------- ---------- ---------- ---------- ----------
1A3_gi|1703237|      ---------- ---------- ---------- ---------- ---------- ----------
1A4_gi|10946870|     ---------- ---------- ---------- ---------- ---------- ----------
1B1_gi|4502049|      ---------- ---------- ---------- ---------- ---------- ----------
1B2_gi|1703235|      ---------- ---------- ---------- ---------- ---------- ----------
1B3_gi|1351911|      ---------- ---------- ---------- ---------- ---------- ----------
1B4_gi|6978491|      ---------- ---------- ---------- ---------- ---------- ----------
1B5_gi|113594|       ---------- ---------- ---------- ---------- ---------- ----------
1B6_gi|584742|       ---------- ---------- ---------- ---------- ---------- ----------
1B7_gi|231525|       ---------- ---------- ---------- ---------- ---------- ----------
1B8_gi|6679791|      ---------- ---------- ---------- ---------- ---------- ----------
1B9_gi|2114406|      ---------- ---------- ---------- ---------- ---------- ----------
1B10_gi|223468663|   ---------- ---------- ---------- ---------- ---------- ----------
1B12_gi|14330324|    ---------- ---------- ---------- ---------- ---------- ----------
1B13_gi|15864567|    ---------- ---------- ---------- ---------- ---------- ----------
1B14_gi|148540194|   ---------- ---------- ---------- ---------- ---------- ----------
1B15_gi|51094822|    ---------- ---------- ---------- ---------- ---------- ----------
1C1_gi|5453543|      ---------- ---------- ---------- ---------- ---------- ----------
1C2_gi|4503285|      ---------- ---------- ---------- ---------- ---------- ----------
1C3_gi|24497583|     ---------- ---------- ---------- ---------- ---------- ----------
1C4_gi|308153631|    ---------- ---------- ---------- ---------- ---------- ----------
1C5_gi|1352733|      ---------- ---------- ---------- ---------- ---------- ----------
1C6_gi|13487925|     ---------- ---------- ---------- ---------- ---------- ----------
1C7_gi|129896|       ---------- ---------- ---------- ---------- ---------- ----------
1C8_gi|1709623|      ---------- ---------- ---------- ---------- ---------- ----------
1C9_gi|118634|       ---------- ---------- ---------- ---------- ---------- ----------
1C10a_gi|1345830|    ---------- ---------- ---------- ---------- ---------- ----------
1C10b_gi|1706132|    ---------- ---------- ---------- ---------- ---------- ----------
1C11_gi|1669605|     ---------- ---------- ---------- ---------- ---------- ----------
1C12_gi|85719330|    ---------- ---------- ---------- ---------- ---------- ----------
1C13_gi|171846276|   ---------- ---------- ---------- ---------- ---------- ----------
1C14_gi|19527294|    ---------- ---------- ---------- ---------- ---------- ----------
1C15_gi|741804|      ---------- ---------- ---------- ---------- ---------- ----------
1C16_gi|741803|      ---------- ---------- ---------- ---------- ---------- ----------
1C17_gi|741805|      ---------- ---------- ---------- ---------- ---------- ----------
1C18_gi|1654715|     ---------- ---------- ---------- ---------- ---------- ----------
1C20_gi|16905111|    ---------- ---------- ---------- ---------- ---------- ----------
1C21_gi|126090770|   ---------- ---------- ---------- ---------- ---------- ----------
1C22_gi|38603389|    ---------- ---------- ---------- ---------- ---------- ----------
1C23_gi|62856987|    ---------- ---------- ---------- ---------- ---------- ----------
1C24_gi|84993586|    ---------- ---------- ---------- ---------- ---------- ----------
1C25_gi|15216337|    ---------- ---------- ---------- ---------- ---------- ----------
1D1_gi|5174695|      ---------- ---------- ---------- ---------- ---------- ----------
1D2_gi|398962|       ---------- ---------- ---------- ---------- ---------- ----------
1D3_gi|5689216|      ---------- ---------- ---------- ---------- ---------- ----------
1E1_gi|1698718|      ---------- ---------- ---------- ---------- ---------- ----------
1E2_gi|269849539|    ---------- ---------- ---------- ---------- ---------- ----------
1G1_gi|17550248|     ---------- ---------- ---------- ---------- ---------- ----------
2A1_gi|134153|       ---------- ---------- ---------- ---------- ---------- ----------
2A2_gi|1835701|      ---------- ---------- ---------- ---------- ---------- ----------
2B1_gi|401428|       ---------- ---------- ---------- ---------- ---------- ----------
2B2_gi|1351442|      ---------- ---------- ---------- ---------- ---------- ----------
2B3_gi|2492803|      ---------- ---------- ---------- ---------- ---------- ----------
2B4_gi|1912051|      ---------- ---------- ---------- ---------- ---------- ----------
2B5_gi|3289019|      ---------- ---------- ---------- ---------- ---------- ----------
2B6_gi|6321895|      ---------- ---------- ---------- ---------- ---------- ----------
2B7_gi|1912049|      ---------- ---------- ---------- ---------- ---------- ----------
2B8_gi|4103055|      ---------- ---------- ---------- ---------- ---------- ----------
2C1_gi|1332539|      ---------- ---------- ---------- ---------- ---------- ----------
2D1_gi|7407095|      ---------- ---------- ---------- ---------- ---------- ----------
2E1_gi|4753912|      ---------- ---------- ---------- ---------- ---------- ----------
2E2_gi|18479021|     ---------- ---------- ---------- ---------- ---------- ----------
2E3_gi|5052610|      ---------- ---------- ---------- ---------- ---------- ----------
3A1_gi|121087|       ---------- ---------- ---------- ---------- ---------- ----------
3A2_gi|6320576|      ---------- ---------- ---------- ---------- ---------- ----------
3B1_gi|1142698|      ---------- ---------- ---------- ---------- ---------- ----------
3B2_gi|60458781|     ---------- ---------- ---------- ---------- ---------- ----------
3B3_gi|60458785|     ---------- ---------- ---------- ---------- ---------- ----------
3C1_gi|536474|       ---------- ---------- ---------- ---------- ---------- ----------
3C2_gi|74626610|     ---------- ---------- ---------- ---------- ---------- ----------
3C3_gi|38423524|     ---------- ---------- ---------- ---------- ---------- ----------
3D1_gi|31321885|     ---------- ---------- ---------- ---------- ---------- ----------
3E1_gi|22207641|     ---------- ---------- ---------- ---------- ---------- ----------
3F1_gi|81625481|     ---------- ---------- ---------- ---------- ---------- ----------
3F2_gi|13638516|     ---------- ---------- ---------- ---------- ---------- ----------
3F3_gi|81635765|     ---------- ---------- ---------- ---------- ---------- ----------
4A1_gi|112837|       ---------- ---------- ---------- ---------- ---------- ----------
```

```
4A2_gi|75220959|     ---------- ---------- ---------- ---------- ---------- ----------
4A3_gi|1215788|      ---------- ---------- ---------- ---------- ---------- ----------
4A4_gi|1514979|      ---------- ---------- ---------- ---------- ---------- ----------
4B1_gi|2792155|      ---------- ---------- ---------- ---------- ---------- ----------
4B2_gi|6478210|      ---------- ---------- ---------- ---------- ---------- ----------
4B3_gi|6478204|      ---------- ---------- ---------- ---------- ---------- ----------
4B4_gi|2792295|      ---------- ---------- ---------- ---------- ---------- ----------
4B5_gi|112807104|    ---------- ---------- ---------- ---------- ---------- ----------
4B6_gi|112807098|    ---------- ---------- ---------- ---------- ---------- ----------
4B7_gi|112807100|    ---------- ---------- ---------- ---------- ---------- ----------
4B8_gi|112807102|    ---------- ---------- ---------- ---------- ---------- ----------
4C1_gi|113595|       ---------- ---------- ---------- ---------- ---------- ----------
4C2_gi|167113|       ---------- ---------- ---------- ---------- ---------- ----------
4C3_gi|75221432|     ---------- ---------- ---------- ---------- ---------- ----------
4C4_gi|4539944|      ---------- ---------- ---------- ---------- ---------- ----------
4C5_gi|13160397|     ---------- ---------- ---------- ---------- ---------- ----------
4C6_gi|13160399|     ---------- ---------- ---------- ---------- ---------- ----------
4C8_gi|111182163|    ---------- ---------- ---------- ---------- ---------- ----------
4C9_gi|111182165|    ---------- ---------- ---------- ---------- ---------- ----------
4C10_gi|111182167|   ---------- ---------- ---------- ---------- ---------- ----------
4C11_gi|111182169|   ---------- ---------- ---------- ---------- ---------- ----------
5A1_gi|408360251|    ---------- ---------- ---------- ---------- ---------- ----------
5A2_gi|11127591|     ---------- ---------- ---------- ---------- ---------- ----------
5B1_gi|2506173|      ---------- ---------- ---------- ---------- ---------- ----------
5C1_gi|144969|       ---------- ---------- ---------- ---------- ---------- ----------
5C2_gi|3916039|      ---------- ---------- ---------- ---------- ---------- ----------
5D1_gi|112735|       ---------- ---------- ---------- ---------- ---------- ----------
5E1_gi|5354195|      ---------- ---------- ---------- ---------- ---------- ----------
5F1_gi|82504416|     ---------- ---------- ---------- ---------- ---------- ----------
5G1_gi|16080393|     ---------- ---------- ---------- ---------- ---------- ----------
5G2_gi|16079957|     ---------- ---------- ---------- ---------- ---------- ----------
6A1_gi|18202524|     ---------- ---------- ---------- ---------- ---------- ----------
6A2_gi|499328|       ---------- ---------- ---------- ---------- ---------- ----------
6A3_gi|2135947|      ---------- ---------- ---------- ---------- ---------- ----------
6A4_gi|975314|       ---------- ---------- ---------- ---------- ---------- ----------
6A5_gi|2827466|      ---------- ---------- ---------- ---------- ---------- ----------
6A6_gi|7914984|      ---------- ---------- ---------- ---------- ---------- ----------
6A7_gi|5019764|      ---------- ---------- ---------- ---------- ---------- ----------
6A8_gi|148747467|    ---------- ---------- ---------- ---------- ---------- ----------
6A9_gi|24648619|     DGMAVGLVTA GLANKCIHSL LALENVLDRE QAVTVCQALL YVLREAVAMS VITNNGEDDC
6A10a_gi|726465|     ---------- ---------- ---------- ---------- ---------- ----------
6A10b_gi|5019766|    ---------- ---------- ---------- ---------- ---------- ----------
6A11_gi|5922729|     ---------- ---------- ---------- ---------- ---------- ----------
6A13_gi|52001073|    ---------- ---------- ---------- ---------- ---------- ----------
6A14_gi|226823214|   ---------- ---------- ---------- ---------- ---------- ----------
6B1_gi|902000|       ---------- ---------- ---------- ---------- ---------- ----------
6C1_gi|1063415|      ---------- ---------- ---------- ---------- ---------- ----------
6C2_gi|2832783|      ---------- ---------- ---------- ---------- ---------- ----------
7A1_gi|39932720|     ---------- ---------- ---------- ---------- ---------- ----------
7A2_gi|41327764|     ---------- ---------- ---------- ---------- ---------- ----------
7A3_gi|41152114|     ---------- ---------- ---------- ---------- ---------- ----------
7A4_gi|6815049|      ---------- ---------- ---------- ---------- ---------- ----------
8A1_gi|24638123|     ---------- ---------- ---------- ---------- ---------- ----------
8A2_gi|74627022|     ---------- ---------- ---------- ---------- ---------- ----------
9A1_gi|146345520|    ---------- ---------- ---------- ---------- ---------- ----------
9A2_gi|6093525|      ---------- ---------- ---------- ---------- ---------- ----------
9A3_gi|2492798|      ---------- ---------- ---------- ---------- ---------- ----------
9B1_gi|6323998|      ---------- ---------- ---------- ---------- ---------- ----------
9B2_gi|6319951|      ---------- ---------- ---------- ---------- ---------- ----------
9B3_gi|6319958|      ---------- ---------- ---------- ---------- ---------- ----------
9B4_gi|6322615|      ---------- ---------- ---------- ---------- ---------- ----------
9C1_gi|2459734|      ---------- ---------- ---------- ---------- ---------- ----------
10A1_gi|4731595|     ---------- ---------- ---------- ---------- ---------- ----------
10A2_gi|3256056|     ---------- ---------- ---------- ---------- ---------- ----------
11A1_gi|1176985|     ---------- ---------- ---------- ---------- ---------- ----------
11B1_gi|3123233|     ---------- ---------- ---------- ---------- ---------- ----------
11B2_gi|3123121|     ---------- ---------- ---------- ---------- ---------- ----------
11B3_gi|85062654|    ---------- ---------- ---------- ---------- ---------- ----------
11C1_gi|81787577|    ---------- ---------- ---------- ---------- ---------- ----------
12A1_gi|5305791|     ---------- ---------- ---------- ---------- ---------- ----------
12B1_gi|2599278|     ---------- ---------- ---------- ---------- ---------- ----------
12C1_gi|5921163|     ---------- ---------- ---------- ---------- ---------- ----------
13A1_gi|1351673|     ---------- ---------- ---------- ---------- ---------- ----------
13B1_gi|9106797|     ---------- ---------- ---------- ---------- ---------- ----------
13C1_gi|81555851|    ---------- ---------- ---------- ---------- ---------- ----------
14A1_gi|882530|      ---------- ---------- ---------- ---------- ---------- ----------
15A1_gi|37196700|    ---------- ---------- ---------- ---------- ---------- ----------
all2316_aldo/keto    ---------- ---------- ---------- ---------- ---------- ----------
```

```
                         721
1A1_gi|5174391|      ---------- ----P-ALRK TLADL-Q--- -----LEY-L DLYLMHW--- PYAFER----
1A2_gi|1703236|      ---------- ----P-ALRK TLADL-Q--- -----LEY-L DLYLMHW--- PYAFER----
1A3_gi|1703237|      ---------- ----P-AVRK TLADL-Q--- -----LEY-L DLYLMHW--- PYAFER----
1A4_gi|10946870|     ---------- ----P-ALRK TLADL-Q--- -----LEY-L DLYLMHW--- PYAFER----
1B1_gi|4502049|      ---------- ----G-ACQK TLSDL-K--- -----LDY-L DLYLIHW--- PTGFKP----
1B2_gi|1703235|      ---------- ----G-ACQK TLNDL-K--- -----LDY-L DLYLIHW--- PTGFKH----
1B3_gi|1351911|      ---------- ----G-AFQK TLSDL-Q--- -----LDY-L DLYLIHW--- PTGFKP----
1B4_gi|6978491|      ---------- ----G-ACQK TLSDL-Q--- -----LDY-L DLYLIHW--- PTGFKP----
1B5_gi|113594|       ---------- ----G-ACQK TLSDL-K--- -----LDY-L DLYLIHW--- PTGFKP----
1B6_gi|584742|       ---------- ----G-ACQT TLRDL-K--- -----LDY-L DLYLIHW--- PTGFKP----
1B7_gi|231525|       ---------- ----K-AFQN TLSDL-K--- -----LDY-L DLYLVHW--- PQGFQA----
1B8_gi|6679791|      ---------- ----E-AFQK TLTDL-K--- -----LDY-L DLYLIHW--- PQGLQP----
1B9_gi|2114406|      ---------- ----E-AFQK TLTDL-K--- -----LDY-L DLYLIHW--- PQGLQP----
1B10_gi|223468663|   ---------- ----K-AFEK TLKDL-K--- -----LSY-L DVYLIHW--- PQGFKS----
1B12_gi|14330324|    ---------- ----E-GCKR SLTAL-Q--- -----LDY-V DLYLMHY--- PMGFKA----
1B13_gi|15864567|    ---------- ----E-AFQK TLTDL-K--- -----LDY-L DLYLIHW--- PQGFQA----
1B14_gi|148540194|   ---------- ----E-AFQK TLSDL-K--- -----LDY-L DLYLIHW--- PQGLQA----
1B15_gi|51094822|    ---------- ----K-AFEK TLKDL-K--- -----LSY-L DVYLIHW--- PQGFKSLKKW
1C1_gi|5453543|      ---------- ----P-ALER SLKNL-Q--- -----LDY-V DLYLIHF--- PVSVKP----
1C2_gi|4503285|      ---------- ----P-ALER SLKNL-Q--- -----LDY-V DLYLIHF--- PVSVKP----
1C3_gi|24497583|     ---------- ----P-ALEN SLKKA-Q--- -----LDY-V DLYLIHS--- PMSLKP----
1C4_gi|308153631|    ---------- ----P-ALES SLKKL-Q--- -----LDY-V DLYLLHF--- PMALKP----
1C5_gi|1352733|      ---------- ----P-SLED SLKNL-Q--- -----LDY-V DLYIIHF--- PTALKP----
1C6_gi|13487925|     ---------- ----V-CLEQ SLKQL-Q--- -----LDY-V DLYLIHF--- PMAMKP----
1C7_gi|129896|       ---------- ----P-ALEK SLQNL-Q--- -----LDY-V DLYIIHS--- PVSLKP----
1C8_gi|1709623|      ---------- ----P-SLEN SLRKL-N--- -----LDY-V DLYLIHF--- PVSLKP----
1C9_gi|118634|       ---------- ----T-CLEK TLKST-Q--- -----LDY-V DLYIIHF--- PMALQP----
1C10a_gi|1345830|    ---------- ----K-GLER SLRDV-G--- -----MDY-L DLFLMHW--- PVSLKP----
1C10b_gi|1706132|    ---------- ----K-GLER SLRDV-G--- -----MDY-L DLFLMHW--- PVSLKP----
1C11_gi|1669605|     ---------- ----P-ALEK SLNNL-Q--- -----LDY-V DLYIIHF--- PVALKP----
1C12_gi|85719330|    ---------- ----P-ALEK SLKSL-Q--- -----LDY-V DLYLIHY--- PVPMKP----
1C13_gi|171846276|   ---------- ----P-ALEK SLKKL-Q--- -----LDY-V DLYIMHY--- PVPMKS----
1C14_gi|19527294|    ---------- ----S-CLEK TLKNA-Q--- -----LDY-V DLYIIHF--- PMALQP----
1C15_gi|741804|      ---------- ----Q-CLER SLKKL-G--- -----LDY-V DLCIIHI--- PIAMKP----
1C16_gi|741803|      ---------- ----P-ALEK SLKNL-Q--- -----LDY-A DLYIMHY--- PVPMKS----
1C17_gi|741805|      ---------- ----P-ALEK SLKNL-Q--- -----LDY-A DLYIMHY--- PVPMKS----
1C18_gi|1654715|     ---------- ----GACCCA GCTTG-G--- -----AAA-A TTCCCTG--- AGGAAA----
1C20_gi|16905111|    ---------- ----P           G--- -----ENY
1C21_gi|126090770|   ---------- ----A-SLER SLQKL-Q--- -----FDY-V DLYLIHY--- PMALKP----
1C22_gi|38603389|    ---------- ----P-ALEK SLKKL-Q--- -----LDY-V DLYIMHY--- PVPMKS----
1C23_gi|62856987|    ---------- ----P-ALEK SLKNL-Q--- -----LDY-V DLYIIHF--- PVAVKP----
1C24_gi|84993586|    ---------- ----P-ALEK SLKNL-Q--- -----LDY-V DLFLIHY--- PVPIKS----
1C25_gi|15216337|    ---------- ----P-ALES SLKKL-Q--- -----LDY-V DLYLIHF--- PMALKP----
1D1_gi|5174695|      ---------- ----P-TLER TLRVL-Q--- -----LDY-V DLYIIEV--- PMAFKP----
1D2_gi|398962|       ---------- ----P-ALER TLQTL-K--- -----LDY-I DLYIIEM--- PMAFKP----
1D3_gi|5689216|      ---------- ----P-TLER TLKVL-K--- -----LDY-I DLYIIEI--- PMAFKP----
1E1_gi|1698718|      ---------- ----T-ACTN TLEAL-N--- -----LDY-L DLYLIHW--- PIGFKP----
1E2_gi|269849539|    ---------- ----T-ACRK SLKAL-K--- -----LNY-L DLYLIHW--- PMGFKP----
1G1_gi|17550248|     ---------- ----G-GLRE SLKKL-Q--- -----LEY-V DLYLAHM--- PAAFND----
2A1_gi|134153|       ---------- ----E-ACKN SLEKL-Q--- -----IDY-L DLYLVHY--- PMPTKH----
2A2_gi|1835701|      ---------- ----E-ACKN SLKKL-Q--- -----LEY-L DLYLIHF--- PMASKH----
2B1_gi|401428|       ---------- ----K-ALNR TLSDL-Q--- -----VDY-V DLFLIHF--- PVTFKF----
2B2_gi|1351442|      ---------- ----R-ALER TLSDL-Q--- -----LDY-V DIFYIHF--- PLAFKP----
2B3_gi|2492803|      ---------- ----K-ALMK SLSDF-N--- -----LDY-F DLYLMHF--- PISFKF----
2B4_gi|1912051|      ---------- ----T-ALNK TLSDL-N--- -----LDY-V DLFLIHF--- PIAFKF----
2B5_gi|3289019|      ---------- ----T-ALNK TLADL-K--- -----VDY-V DLFLIHF--- PIAFKF----
2B6_gi|6321895|      ---------- ----NLAGKN DLSEYTGGLV NDNSEISY-T KQNHRYS--- NISFAS----
2B7_gi|1912049|      ---------- ----T-ALNK TLSDL-N--- -----LDY-V DLFLIHF--- PIAFKF----
2B8_gi|4103055|      ---------- ----K-ALDK TLSDL-K--- -----VDY-L DLFLIHF--- PIAFKF----
2C1_gi|1332539|      ---------- ----A-LFDR QLKDT-G--- -----LEY-F DLYLIHF--- PVPLQY----
2D1_gi|7407095|      ---------- ----P-ICRK QLADW-G--- -----IDY-F DLYIVHF--- PISLKY----
2E1_gi|4753912|      ---------- ----V-AMKE TLNKT-G--- -----LDY-V DLFLMHW--- PIALNE----
2E2_gi|18479021|     ---------- ----P-ALRE SLTKL-G--- -----LSY-V DLYLIHS--- PEATNE----
2E3_gi|5052610|      ---------- ----Y-ACRK TLQNF-G--- -----LQY-V DLYLMHW--- PYSYVY----
3A1_gi|121087|       ---------- ------ALDQ SLKRL-G--- -----LDY-V DLYLMHW--- PARLDP----
3A2_gi|6320576|      ---------- ----EAALNK SLKRL-G--- -----LDY-V DLYLMHW--- PVPLKT----
3B1_gi|1142698|      ---------- ----P-ALDD TLKEL-G--- -----LEY-L DLYLIHW--- PVAFPP----
3B2_gi|60458781|     ---------- ----P-ALDE TLKEL-G--- -----LSY-L DLYLIHW--- PVAFKF----
3B3_gi|60458785|     ---------- ----P-ALDE TLKEL-G--- -----LSY-L DLYLIHW--- PVAFKF----
3C1_gi|536474|       ---------- ----R-SLNE SLKAL-G--- -----LEY-V DLLLQHW--- PLCFEK----
3C2_gi|74626610|     ---------- ----E-ALNE SLRKL-G--- -----TDY-L DLYLLHS--- PIPFYE----
3C3_gi|38423524|     ---------- ----SDSIDK ALAQL-G--- -----VDY-V DLFLIHS--- PFFTTE----
3D1_gi|31321885|     ---------- ----L-GLDQ SLKLL-G--- -----LDY-V DLYLVHW--- PILMNP----
3E1_gi|22207641|     ---------- ----W-SIDD SLKRL-G--- -----LDY-V DMFLVHW--- PIAAEK----
3F1_gi|81625481|     ---------- ----R-SLEN TLKRL-D--- -----TDY-V DLYLIHW--- PNPEIP----
3F2_gi|13638516|     ---------- ----P-SLKE SLQKL-R--- -----TDY-V DLTLIHW--- PSPNDE----
3F3_gi|81635765|     ---------- ----P-SVEA SLKAL-R--- -----VDQ-A DVLMLHW--- P
4A1_gi|112837|       ---------- ----P-ALRK SLKTL-Q--- -----LEY-L DLYLIHW--- PLSSQP----
```

```
4A2_gi|75220959|     ---------- ----P-ALQK SLKTL-Q--- -----LDY-L DLYLIHW--- PLSSQP----
4A3_gi|1215788|      ---------- ----P-ALRK SLETL-Q--- -----LEY-L DLYLIHW--- PLSSQP----
4A4_gi|1514979|      ---------- ----P-ALRK SLETL-Q--- -----LEY-L DLYLIHW--- PLSSQP----
4B1_gi|2792155|      ---------- ----P-ALKT TLKKL-G--- -----MEY-V DLYLIHW--- PVRLRH----
4B2_gi|6478210|      ---------- ----P-ALQN SLRNL-K--- -----LEY-L DLYLIHH--- PVSLKP----
4B3_gi|6478204|      ---------- ----P-ALQN SLRNL-K--- -----LDY-L DLYLIHH--- PVSLKP----
4B4_gi|2792295|      ---------- ----P-SIKA SLSNL-Q--- -----VEY-I DMYIIHW--- PFKLGK----
4B5_gi|112807104|    ---------- ----P-ALRR TLSNL-Q--- -----MEY-V DLYMVHW--- PVTMKA----
4B6_gi|112807098|    ---------- ----P-ALRQ TLWNL-Q--- -----MEY-V DLYLVHW--- PVSMKP----
4B7_gi|112807100|    ---------- ----P-ALRH TLRNL-Q--- -----MEY-V DLYLVHW--- PVSMKP----
4B8_gi|112807102|    ---------- ----P-ALRQ TLRNL-Q--- -----MEY-V DLYLVHW--- PVSMKP----
4C1_gi|113595|       ---------- ----P-ALEN TLKDL-Q--- -----LDY-I DLYHIHW--- PFRLKD----
4C2_gi|167113|       ---------- ----P-ALEK TLKDL-Q--- -----LDY-L DLYLIHW--- PFRLKD----
4C3_gi|75221432|     ---------- ----P-ALEN TLKDL-Q--- -----LDY-I DLYLIHW--- PFRLKD----
4C4_gi|4539944|      ---------- ----P-ALKN TLEEL-Q--- -----LDY-L DLYLIHW--- PIHLKK----
4C5_gi|13160397|     ---------- ----V-ALDK TLEDL-Q--- -----LDY-I DLYLIHW--- PVRLKK----
4C6_gi|13160399|     ---------- ----V-ALDK TLEDL-Q--- -----LDY-I DLYLIHW--- PVRLKK----
4C8_gi|111182163|    ---------- ----K-ALEK TLQDL-Q--- -----IDY-V DLYLIHW--- PASLKK----
4C9_gi|111182165|    ---------- ----E-ALNR TLKDL-Q--- -----LEY-V DLYLIHW--- PARIKK----
4C10_gi|111182167|   ---------- ----E-ALNR TLQDL-Q--- -----LDY-V DLYLIHW--- PVSLKK----
4C11_gi|111182169|   ---------- ----D-ALNR TLQDL-Q--- -----LDY-V DLYLMHW--- PVRLKK----
5A1_gi|408360251|    ---------- ----A-AFEE SRQKL-G--- -----VDY-I DLYLIHW--- PR
5A2_gi|11127591|     ---------- ----S-AFEK SIKKL-G--- -----LEY-V DLYLIHW--- PGKDKF----
5B1_gi|2506173|      ---------- ----R-AFDE SLGRL-G--- -----LDY-V DLYLLHW--- PTKDWN----
5C1_gi|144969|       ---------- ----A-AIAE SLAKL-A--- -----LDQ-V DLYLVHW--- PTPAAD----
5C2_gi|3916039|      ---------- ----E-ALLD SLKKL-Q--- -----LDY-I DLYLMHW--- PVPAID----
5D1_gi|112735|       ---------- ----D-SIRG SLDRL-G--- -----LDV-I DLQLIHW--- PNPSVG----
5E1_gi|5354195|      ---------- ----A-TVEE SLYRA-Q--- -----LDY-Y DLYLIHW--- PNPSKD----
5F1_gi|82504416|     ---------- ----A-QFER SLNRL-Q--- -----LDY-V DLYLIHQ--- PYGDVH----
5G1_gi|16080393|     ---------- ----A-AFEK SLERL-Q--- -----LDY-L DLYLIHW--- PGKDKY----
5G2_gi|16079957|     ---------- ----A-AFET SLSKL-G--- -----LDY-L DLYLIHW--- PVEGKY----
6A1_gi|18202524|     ---------- ----E-GLKA SLERL-Q--- -----LEY-V DVVFANR--- PDPNTP----
6A2_gi|499328|       ---------- ----E-GLKA SLERL-Q--- -----LEY-V DVVFANR--- PDPNTP----
6A3_gi|2135947|      ---------- ----E-GLKG SLQRL-Q--- -----LEY-V DVVFANR--- PDSNTP----
6A4_gi|975314|       ---------- ----E-GLKA SLERL-Q--- -----LEY-V DVVFANR--- PDPNTP----
6A5_gi|2827466|      ---------- ----E-GLKA SLERL-Q--- -----LEY-V DVVFANR--- PDPNTP----
6A6_gi|7914984|      ---------- ----E-GLKA SLERL-Q--- -----LEY-V DVVFANR--- PDPNTP----
6A7_gi|5019764|      ---------- ----E-GLKG SLQRL-Q--- -----LEY-V DVVFANR--- PDSNTP----
6A8_gi|148747467|    ---------- ----E-GLKG SLQRL-Q--- -----LEY-V DVVFANR--- PDSNTP----
6A9_gi|24648619|     HSVSSAGSSY LKLYPRGTEQ VVNDP-QVLH SVMVGLRTLI ERFKITW--- KESVET----
6A10a_gi|726465|     ---------- ----E-GLKG SLQRL-Q--- -----LEY-V DVVFANR--- PDSNTP----
6A10b_gi|5019766|    ---------- ----E-GLKG SLQRL-Q--- -----LEY-V DVVFANR--- PDSNTP----
6A11_gi|5922729|     ---------- ----E-GLRG SLERL-Q--- -----LDY-V DIVFANR--- MDPNSP----
6A13_gi|52001073|    ---------- ----E-GLKG SLQRL-Q--- -----LEY-V DVVFANR--- PDSNTP----
6A14_gi|226823214|   ---------- ----E-GLQG SLDRL-Q--- -----LEY-V DIVFANR--- SDPNSP----
6B1_gi|902000|       ---------- ----E-CVRA SLQRL-Q--- -----LQY-I DIVIIHK--- ADPMCP----
6C1_gi|1063415|      ---------- ----E-GTKA SLKRL-D--- -----MDY-V DVLYCHR--- PDASTP----
6C2_gi|2832783|      ---------- ----E-GLRA SLKRL-D--- -----MDY-V DIVYCHR--- PDASTP----
7A1_gi|39932720|     ---------- ----F-QLET SLKRL-Q--- -----CPR-V DLFYLHF--- PDHGTP----
7A2_gi|41327764|     ---------- ----S-QLET SLKRL-Q--- -----CPQ-V DLFYLHA--- PDHGTP----
7A3_gi|41152114|     ---------- ----F-QLET SLKRL-Q--- -----CPR-V DLFYLHM--- PDHSTP----
7A4_gi|6815049|      ---------- ----S-QLET SLKRL-Q--- -----CPR-V DLFYLHA--- PDHGTP----
8A1_gi|24638123|     ---------- ----K-SVEN VIAHLRG--- -----TKK-L DLFQCAR--- VDPNVP----
8A2_gi|74627022|     ---------- ----K-SVKN ALTRLRG--- -----KKK-L DLFQCAR--- VDHKVP----
9A1_gi|146345520|    ---------- ----L-SVEA SLAKL-R--- -----TDY-I DLLYVHM--- WDFSTS----
9A2_gi|6093525|      ---------- ----L-SVKA SLQKL-Q--- -----TDY-I DLLYVHM--- WDFTTS----
9A3_gi|2492798|      ---------- ----I-SVHD SLRKL-R--- -----TSY-I DIFYVHF--- WDYTCT----
9B1_gi|6323998|      ---------- ----V-SVRD SLRKL-Q--- -----TDW-I DILYIHW--- WDYMSS----
9B2_gi|6319951|      ---------- ------SVRD SLRKL-Q--- -----TDW-I DILYVHW--- WDYMSS----
9B3_gi|6319958|      ---------- ------SVRD SLRKL-Q--- -----TDW-I DILYVHW--- WDYMSS----
9B4_gi|6322615|      ---------- ------SVRD SLRKL-Q--- -----TDW-I DILYVHW--- WDYMSS----
9C1_gi|2459734|      ---------- ----H-RIDA LLDRL-D--- -----TDY-V DVLYIHR--- WDDETP----
10A1_gi|4731595|     ---------- ----P-QLRE SFERM-G--- -----REN-A ALYMLHR--- DNPSVP----
10A2_gi|3256056|     ---------- ----A-QLAE SFDRM-G--- -----RDD-A AMYMLHR--- DNPDVP----
11A1_gi|1176985|     ---------- ----K-SVDE SLKRL-N--- -----TDY-I DLFYIHF--- PDEHTP----
11B1_gi|3123233|     ---------- ----E-EVEN SLKRL-Q--- -----TDY-I DLYQVHW--- PDPLVP----
11B2_gi|3123121|     ---------- ----E-EVAA SLQRL-G--- -----IDY-I DIYMTHWQSV PPFFTP----
11B3_gi|85062654|    ---------- ----T-ACEN SLNRL-Q--- -----TDY-I DLYQIHW    PAGTWN----
11C1_gi|81787577|    ---------- ----E-AVKD SLRRL-Q--- -----TDY-I DLYQLHG--- GTIDDP----
12A1_gi|5305791|     ---------- ----R-SCEA SLRRL-G--- -----VDH-I DLYQMHR--- MDRTVR----
12B1_gi|2599278|     ---------- ----A-SCEG SLRRL-G--- -----VDH-I DVYQMHH--- IDRSAP----
12C1_gi|5921163|     ---------- ----A-ACDA SLRRL-R--- -----TDW-I DLYQLHH--- IDRRAG----
13A1_gi|1351673|     ---------- ----K-ALDL SLKRL-G--- -----IDC-I DLYYVHR--- FSGETP----
13B1_gi|9106797|     ---------- ----Q-CVYM SLRRL-K--- -----LEQ-I DLWQLHR--- IDPKVP----
13C1_gi|81555851|    ---------- ----S-AIEG SLKRL-K--- -----VEC-I DLYYQHR--- MDTNTP----
14A1_gi|882530|      ---------- ----A-SLDQ SLKRM-G--- -----LEY-V DIFYSHR--- VDENTP----
15A1_gi|37196700|    ---------- ----R-SFDD SQQRL-G--- -----FPE-I DLLYVHD--- IGRVTH----
all2316_aldo/keto    ---------- ----Q-TIRV SFEAL-G--- -----GEKPI DVWQYHS--- PDPEYT----
```

```
                      781
1A1_gi|5174391|       ---------- ---------- ---------- ------GD-- ---------- -------NPF
1A2_gi|1703236|       ---------- ---------- ---------- ------GD-- ---------- -------NPF
1A3_gi|1703237|       ---------- ---------- ---------- ------GD-- ---------- -------NPF
1A4_gi|10946870|      ---------- ---------- ---------- ------GD-- ---------- -------NPF
1B1_gi|4502049|       ---------- ---------- ---------- ------GK-- ---------- -------EFF
1B2_gi|1703235|       ---------- ---------- ---------- ------GS-- ---------- -------EYF
1B3_gi|1351911|       ---------- ---------- ---------- ------GP-- ---------- -------DYF
1B4_gi|6978491|       ---------- ---------- ---------- ------GP-- ---------- -------DYF
1B5_gi|113594|        ---------- ---------- ---------- ------GK-- ---------- -------DFF
1B6_gi|584742|        ---------- ---------- ---------- ------GK-- ---------- -------DPF
1B7_gi|231525|        ---------- ---------- ---------- ------GN-- ---------- -------ALL
1B8_gi|6679791|       ---------- ---------- ---------- ------GK-- ---------- -------ELF
1B9_gi|2114406|       ---------- ---------- ---------- ------GK-- ---------- -------ELF
1B10_gi|223468663|    ---------- ---------- ---------- ------GD-- ---------- -------DLF
1B12_gi|14330324|     ---------- ---------- ---------- ------GE-- ---------- -------ELL
1B13_gi|15864567|     ---------- ---------- ---------- ------GK-- ---------- -------ELF
1B14_gi|148540194|    ---------- ---------- ---------- ------GK-- ---------- -------EFL
1B15_gi|51094822|     YADVVFSKHQ DPETLLNRTK CPDADSSKLF ILYLQTGD-- ---------- -------DFF
1C1_gi|5453543|       ---------- ---------- ---------- ------GE-- ---------- -------EVI
1C2_gi|4503285|       ---------- ---------- ---------- ------GE-- ---------- -------EVI
1C3_gi|24497583|      ---------- ---------- ---------- ------GE-- ---------- -------ELS
1C4_gi|308153631|     ---------- ---------- ---------- ------GE-- ---------- -------TPL
1C5_gi|1352733|       ---------- ---------- ---------- ------GV-- ---------- -------EII
1C6_gi|13487925|      ---------- ---------- ---------- ------GE-- ---------- -------NYL
1C7_gi|129896|        ---------- ---------- ---------- ------GN-- ---------- -------KFV
1C8_gi|1709623|       ---------- ---------- ---------- ------GD-- ---------- -------ELL
1C9_gi|118634|        ---------- ---------- ---------- ------GD-- ---------- -------IFF
1C10a_gi|1345830|     ---------- ---------- ---------- ------SG-- ---------- -------ASD
1C10b_gi|1706132|     ---------- ---------- ---------- ------SG-- ---------- -------ASD
1C11_gi|1669605|      ---------- ---------- ---------- ------GE-- ---------- -------TLF
1C12_gi|85719330|     ---------- ---------- ---------- ------GD-- ---------- -------NES
1C13_gi|171846276|    ---------- ---------- ---------- ------GD-- ---------- -------NDF
1C14_gi|19527294|     ---------- ---------- ---------- ------GD-- ---------- -------KLF
1C15_gi|741804|       ---------- ---------- ---------- ------GE-- ---------- -------ELL
1C16_gi|741803|       ---------- ---------- ---------- ------GD-- ---------- -------NDF
1C17_gi|741805|       ---------- ---------- ---------- ------GD-- ---------- -------KYL
1C18_gi|1654715|      ---------- ---------- --------CT TAATTTGG-- ---------- -------ACT
1C20_gi|16905111|     ---------- ---------- ---------- ---------- ---------- ---------F
1C21_gi|126090770|    ---------- ---------- ---------- ------GE-- ---------- -------ENF
1C22_gi|38603389|     ---------- ---------- ---------- ------GD-- ---------- -------NDF
1C23_gi|62856987|     ---------- ---------- ---------- ------GE-- ---------- -------EHL
1C24_gi|84993586|     ---------- ---------- ---------- ------SV-- ---------- -------DES
1C25_gi|15216337|     ---------- ---------- ---------- ------GE-- ---------- -------TPL
1D1_gi|5174695|       ---------- ---------- ---------- ------GD-- ---------- -------EIY
1D2_gi|398962|        ---------- ---------- ---------- ------GE-- ---------- -------EFY
1D3_gi|5689216|       ---------- ---------- ---------- ------GD-- ---------- -------VVY
1E1_gi|1698718|       ---------- ---------- ---------- ------GE-- ---------- -------KDI
1E2_gi|269849539|     ---------- ---------- ---------- ------PHPE WIMSCSELSF CLSHPRVQDL
1G1_gi|17550248|      ---------- ---------- ---------- ---------- ---------- ----------
2A1_gi|134153|        ---------- ---------- ---------- ---NAIGK-- ---------- -------TAS
2A2_gi|1835701|       ---------- ---------- ---------- ---SGIGT-- ---------- -------TRS
2B1_gi|401428|        ---------- ---------- ---------- ---VPLEE-- ---------- -------KYP
2B2_gi|1351442|       ---------- ---------- ---------- ---VPFDE-- ---------- -------KYP
2B3_gi|2492803|       ---------- ---------- ---------- ---VPFEE-- ---------- -------KYP
2B4_gi|1912051|       ---------- ---------- ---------- ---VPIEE-- ---------- -------KYP
2B5_gi|3289019|       ---------- ---------- ---------- ---VPIEE-- ---------- -------KYP
2B6_gi|6321895|       ---------- ---------- ---------- ---------- ---------- --TTSNASLL
2B7_gi|1912049|       ---------- ---------- ---------- ---VPIEE-- ---------- -------KYP
2B8_gi|4103055|       ---------- ---------- ---------- ---VPFEE-- ---------- -------KYP
2C1_gi|1332539|       ---------- ---------- ---------- ---VDPAT-- ---------- -------VYP
2D1_gi|7407095|       ---------- ---------- ---------- ---VDPAV-- ---------- -------RYP
2E1_gi|4753912|       ---------- ---------- ---------- ---------- ---------- ----------
2E2_gi|18479021|      ---------- ---------- ---------- ---------- ---------- ----------
2E3_gi|5052610|       ---------- ---------- ---------- -----RGDN- ---------- -------EMM
3A1_gi|121087|        ---------- ---------- ---------- ---------- ---------A YIKNEDILSV
3A2_gi|6320576|       ---------- ---------- ---------- ---------- ---------D RVTDGNVLCI
3B1_gi|1142698|       ---------- ---------- ---------- -----EGDIT Q--------- -------NLF
3B2_gi|60458781|      ---------- ---------- ---------- ---------- ---------- ---TTPDELL
3B3_gi|60458785|      ---------- ---------- ---------- ---------- ---------- ---TTPQELF
3C1_gi|536474|        ---------- ---------- ---------- ---------- -------IKD PKGISGLVKT
3C2_gi|74626610|      ---------- ---------- ---------- ---------- ---------- ----------
3C3_gi|38423524|      ---------- ---------- ---------- ---------- ---------- ----------
3D1_gi|31321885|      ---------- ---------- ---------- ----EGND-- ---------- -------EKF
3E1_gi|22207641|      ---------- ---------- ---------- -----NGQ-- ---------- -------GEP
3F1_gi|81625481|      ---------- ---------- ---------- ---------- ---------- ----------
3F2_gi|13638516|      ---------- ---------- ---------- ---------- ---------- ----------
3F3_gi|81635765|      ---------- ---------- ---------- ---------- ---------- ----------
4A1_gi|112837|        ---------- ---------- ---------- ---------- ---------- -------GKF
```

```
4A2_gi|75220959|      ---------- ---------- ---------- ------GK-- ---------- -------FTF
4A3_gi|1215788|       ---------- ---------- ---------- ---------- ---------- -------GKF
4A4_gi|1514979|       ---------- ---------- ---------- ---------- ---------- -------GKF
4B1_gi|2792155|       ---------- ---------- ---------- ---------- ---------- -----DLENP
4B2_gi|6478210|       ---------- ---------- ---------- ---------- ---------- -------GKF
4B3_gi|6478204|       ---------- ---------- ---------- ---------- ---------- -------GKF
4B4_gi|2792295|       ---------- ---------- ---------- ---------- ---------- -------EVR
4B5_gi|112807104|     ---------- ---------- ---------- ---------- ---------- -------GRF
4B6_gi|112807098|     ---------- ---------- ---------- ---------- ---------- -------GRY
4B7_gi|112807100|     ---------- ---------- ---------- ---------- ---------- -------GRF
4B8_gi|112807102|     ---------- ---------- ---------- ---------- ---------- -------GRF
4C1_gi|113595|        ---------- ---------- ---------- ---------- ---------- -------GAH
4C2_gi|167113|        ---------- ---------- ---------- ---------- ---------- -------GAH
4C3_gi|75221432|      ---------- ---------- ---------- ---------- ---------- -------GAH
4C4_gi|4539944|       ---------- ---------- ---------- ---------- ---------- -------GAH
4C5_gi|13160397|      ---------- ---------- ---------- ---------- ---------- -------GSV
4C6_gi|13160399|      ---------- ---------- ---------- ------GS-- ---------- ----------
4C8_gi|111182163|     ---------- ---------- ---------- ---------- ---------- ------ESLM
4C9_gi|111182165|     ---------- ---------- ---------- ------GS-- ---------- ----------
4C10_gi|111182167|    ---------- ---------- ---------- ------GS-- ---------- ----------
4C11_gi|111182169|    ---------- ---------- ---------- ------GT-- ---------- ----------
5A1_gi|408360251|     ---------- ---------- ---------- ---------- ---------- ----------
5A2_gi|11127591|      ---------- ---------- ---------- ---------- ---------- ----------
5B1_gi|2506173|       ---------- ---------- ---------- ---------- ---------- ----------
5C1_gi|144969|        ---------- ---------- ---------- ---------- ---------- ----------
5C2_gi|3916039|       ---------- ---------- ---------- ---------- ---------- ----------
5D1_gi|112735|        ---------- ---------- ---------- ---------- ---------- ----------
5E1_gi|5354195|       ---------- ---------- ---------- ---------- ---------- ----------
5F1_gi|82504416|      ---------- ---------- ---------- ------GA-- ---------- ----------
5G1_gi|16080393|      ---------- ---------- ---------- ---------- ---------- ----------
5G2_gi|16079957|      ---------- ---------- ---------- ---------- ---------- ----------
6A1_gi|18202524|      ---------- ---------- ---------- ---------- ---------- ----------
6A2_gi|499328|        ---------- ---------- ---------- ---------- ---------- ----------
6A3_gi|2135947|       ---------- ---------- ---------- ---------- ---------- ----------
6A4_gi|975314|        ---------- ---------- ---------- ---------- ---------- ----------
6A5_gi|2827466|       ---------- ---------- ---------- ---------- ---------- ----------
6A6_gi|7914984|       ---------- ---------- ---------- ---------- ---------- ----------
6A7_gi|5019764|       ---------- ---------- ---------- ---------- ---------- ----------
6A8_gi|148747467|     ---------- ---------- ---------- ---------- ---------- ----------
6A9_gi|24648619|      ---------- -------IGL VNCLAFVLEN TSMDARCTVQ ALKLVQLAVE HFLSPNMALL
6A10a_gi|726465|      ---------- ---------- ---------- ---------- ---------- ----------
6A10b_gi|5019766|     ---------- ---------- ---------- ---------- ---------- ----------
6A11_gi|5922729|      ---------- ---------- ---------- ---------- ---------- ----------
6A13_gi|52001073|     ---------- ---------- ---------- ---------- ---------- ----------
6A14_gi|226823214|    ---------- ---------- ---------- ---------- ---------- ----------
6B1_gi|902000|        ---------- ---------- ---------- ---------- ---------- ----------
6C1_gi|1063415|       ---------- ---------- ---------- ---------- ---------- ----------
6C2_gi|2832783|       ---------- ---------- ---------- ---------- ---------- ----------
7A1_gi|39932720|      ---------- ---------- ---------- ---------- ---------- ----------
7A2_gi|41327764|      ---------- ---------- ---------- ---------- ---------- ----------
7A3_gi|41152114|      ---------- ---------- ---------- ---------- ---------- ----------
7A4_gi|6815049|       ---------- ---------- ---------- ---------- ---------- ----------
8A1_gi|24638123|      ---------- ---------- ---------- ---------- ---------- ----------
8A2_gi|74627022|      ---------- ---------- ---------- ---------- ---------- ----------
9A1_gi|146345520|     ---------- ---------- ---------- ---------- ---------- ----------
9A2_gi|6093525|       ---------- ---------- ---------- ---------- ---------- ----------
9A3_gi|2492798|       ---------- ---------- ---------- ---------- ---------- ----------
9B1_gi|6323998|       ---------- ---------- ---------- ---------- ---------- ----------
9B2_gi|6319951|       ---------- ---------- ---------- ---------- ---------- ----------
9B3_gi|6319958|       ---------- ---------- ---------- ---------- ---------- ----------
9B4_gi|6322615|       ---------- ---------- ---------- ---------- ---------- ----------
9C1_gi|2459734|       ---------- ---------- ---------- ---------- ---------- ----------
10A1_gi|4731595|      ---------- ---------- ---------- ---------- ---------- ----------
10A2_gi|3256056|      ---------- ---------- ---------- ---------- ---------- ----------
11A1_gi|1176985|      ---------- ---------- ---------- ---------- ---------- ----------
11B1_gi|3123233|      ---------- ---------- ---------- ---------- ---------- ----------
11B2_gi|3123121|      ---------- ---------- ---------- ---------- ---------- ----------
11B3_gi|85062654|     ---------- ---------- ---------- ---------- ---------- ----------
11C1_gi|81787577|     ---------- ---------- ---------- ---------- ---------- ----------
12A1_gi|5305791|      ---------- ---------- ---------- ---------- ---------- ----------
12B1_gi|2599278|      ---------- ---------- ---------- ---------- ---------- ----------
12C1_gi|5921163|      ---------- ---------- ---------- ---------- ---------- ----------
13A1_gi|1351673|      ---------- ---------- ---------- ---------- ---------- ----------
13B1_gi|9106797|      ---------- ---------- ---------- ---------- ---------- ----------
13C1_gi|81555851|     ---------- ---------- ---------- ---------- ---------- ----------
14A1_gi|882530|       ---------- ---------- ---------- ---------- ---------- ----------
15A1_gi|37196700|     ---------- ---------- ---------- ---------- ---------- ----------
all2316_aldo/keto     ---------- ---------- ---------- ---------- ---------- ----------
```

```
                    841
1A1_gi|5174391|     PKNADGTIC- ------YDST HYKET----- ------WKAL EALV---AKG LVQA------
1A2_gi|1703236|     PKNADGTIR- ------YDAT HYKDT----- ------WKAL EALV---AKG LVRA------
1A3_gi|1703237|     PKNADGTVK- ------YDST HYKET----- ------WKAL EALV---AKG LVKA------
1A4_gi|10946870|    PKNADGTVR- ------YDST HYKET----- ------WKAL EVLV---AKG LVKA------
1B1_gi|4502049|     PLDESGNVV- ------PSDT NILDT----- ------WAAM EELV---DEG LVKA------
1B2_gi|1703235|     PLDAAGNVI- ------PSDT DFLDT----- ------WEAM EGLV---DEG LVKS------
1B3_gi|1351911|     PLDASGNVI- ------PSDT DFVDT----- ------WTAM EQLV---DEG LVKT------
1B4_gi|6978491|     PLDASGNVI- ------PSDT DFVDT----- ------WTAM EQLV---DEG LVKA------
1B5_gi|113594|      PLDEDGNVI- ------PSEK DFVDT----- ------WTAM EELV---DEG LVKA------
1B6_gi|584742|      PLDGDGNVV- ------PDES DFVET----- ------WEAM EELV---DEG LVKA------
1B7_gi|231525|      PKDNKGKVL- ------LSKS TFLDA----- ------WEAM EELV---DQG LVKA------
1B8_gi|6679791|     PKDDQGRIL- ------TSKT TFLEA----- ------WEGM EELV---DQG LVKA------
1B9_gi|2114406|     PKDDQGNVL- ------TSKI TFLDA----- ------WEVM EELV---DEG LVKA------
1B10_gi|223468663|  PKDDKGNAI- ------GGKA TFLDA----- ------WEAM EELV---DEG LVKA------
1B12_gi|14330324|   PEDDKGMII- ------PSDT DFLDT----- ------WEAM EELV---DCG KVKA------
1B13_gi|15864567|   PKDEQGNVL- ------PSKT TFLEA----- ------WEGM EELV---DQG LVKA------
1B14_gi|148540194|  PKDSQGKVL- ------MSKS TFLDA----- ------WEGM EELV---DQG LVKA------
1B15_gi|51094822|   PKDDKGNMI- ------SGKG TFLDA----- ------WEAM EELV---DEG LVKA------
1C1_gi|5453543|     PKDENGKIL- ------FDTV DLCAT----- ------WEAV EKCK---DAG LAKS------
1C2_gi|4503285|     PKDENGKIL- ------FDTV DLCAT----- ------WEAM EKCK---DAG LAKS------
1C3_gi|24497583|    PTDENGKVI- ------FDIV DLCTT----- ------WEAM EKCK---DAG LAKS------
1C4_gi|308153631|   PKDENGKVI- ------FDTV DLSAT----- ------WEVM EKCK---DAG LAKS------
1C5_gi|1352733|     PTDEHGKAI- ------FDTV DICAT----- ------WEAM EKCK---DAG LAKS------
1C6_gi|13487925|    PKDENGKLI- ------YDAV DICDT----- ------WEAM EKCK---DAG LAKS------
1C7_gi|129896|      PKDESGKLI- ------FDSV DLCHT----- ------WEAL EKCK---DAG LTKS------
1C8_gi|1709623|     PQDEHGNLI- ------LDTV DLCDT----- ------WEAM EKCK---DAG LAKS------
1C9_gi|118634|      PRDEHGKLL- ------FETV DICDT----- ------WEAM EKCK---DAG LAKS------
1C10a_gi|1345830|   PSDKDKPFI- ------YDNV DLCAT----- ------WEAL EARK---DAG LVRS------
1C10b_gi|1706132|   PSDKDKPFI- ------YDNV DLCAT----- ------WEAL EARK---DAG LVRS------
1C11_gi|1669605|    PTDENGKPI- ------FDSV DLCRT----- ------WEAL EKCK---DAG LTKS------
1C12_gi|85719330|   PLDENGKFL- ------LDTV DFCDT----- ------WERL EECK---DAG LVKS------
1C13_gi|171846276|  PVNEQGKSL- ------LDTV DFCDT----- ------WERL EECK---DAG LVKS------
1C14_gi|19527294|   PRDEHGKLL- ------AEAV DLCDT----- ------WEAM EKCK---DAG LAKS------
1C15_gi|741804|     PKPANGKFI- ------FDTV DIRDT----- ------WEAL EKCK---DAG LSKS------
1C16_gi|741803|     PVPEKGKSL- ------LDTV DFCDT----- ------WEML EKCK---DAG LVKS------
1C17_gi|741805|     PVPDNGKWL- ------LDTV DFCDT----- ------WEML EKCK---DAG LVKS------
1C18_gi|1654715|    ATGTAGACCT CTATCTCATT CATTTCCCAG TGTCTCTGAA GCCA---GGG AATGAGCTTT
1C20_gi|16905111|   PKDENGKFI- ------YDAV DICDT----- ------WEAM EKCK---DAG LAKS------
1C21_gi|126090770|  PVDEHGKLI- ------FDRV DLCAT----- ------WEAM EKCK---DAG LTKS------
1C22_gi|38603389|   PVNEQGKSL- ------LDTV DFCDT----- ------WERL EECK---DAG LVKS------
1C23_gi|62856987|   PQDEQGRMI- ------FDTV DLCAT----- ------WEAM EKCK---DAG LTKS------
1C24_gi|84993586|   PLDEKGKFL- ------LDTV DFCDT----- ------WEML EKCK---DAG LVKS------
1C25_gi|15216337|   PKDENGKVM- ------FDTV DLCAI----- ------WEAM EKCK---DAG LAKS------
1D1_gi|5174695|     PRDENGKWL- ------YHKS NLCAT----- ------WEAM EACK---DAG LVKS------
1D2_gi|398962|      PKDENGRVI- ------YHKS NLCAT----- ------WEAL EACK---DAG LVKS------
1D3_gi|5689216|     PRDENGKWL- ------YHKT NLCAT----- ------WEAL EACK---DAG LVKS------
1E1_gi|1698718|     PLDRNGKVI- ------PSHT SFLDT----- ------WEAM EDLV---FEG LVKN------
1E2_gi|269849539|   PLDESNMVI- ------PSDT DFLDT----- ------WEAM EDLV---ITG LVKN------
1G1_gi|17550248|    ---DMSEHI- --------AS PVEDV----- ------WRQF DAVY---KAG LAKA------
2A1_gi|134153|      LLGEDKVLD- ---I--DVTI SLQQT----- ------WEGM EKTV---SLG LVRS------
2A2_gi|1835701|     ILDDEGVWE- ---V--DATI SLEAT----- ------WHEM EKLV---EMG LVRS------
2B1_gi|401428|      PGFYCGKGD- ---NFDYEDV PILET----- ------WKAL EKLV---KAG KIRS------
2B2_gi|1351442|     PGFYTGKEDE AKGHIEEEQV PLLDT----- ------WRAL EKLV---DQG KIKS------
2B3_gi|2492803|     PGFYCGDGD- ---KFIYEDV PIIET----- ------WRAM ENLV---DEG LVKS------
2B4_gi|1912051|     PGFYCGDGD- ---NFHYEDV PLLDT----- ------WKAL EKLV---EAG KIKS------
2B5_gi|3289019|     PGFYCGDGN- ---NFVYEDV PILET----- ------WKAL EKLV---AAG KIKS------
2B6_gi|6321895|     DFNEMPTSPI PGLNKVTDFQ FIQDT----- ------TKSL ASVYLHSSNR LSRSKLSERT
2B7_gi|1912049|     PGFYCGDGD- ---NFHYEDV PLLDT----- ------WKAL EKLV---EAG KIKS------
2B8_gi|4103055|     PGFYCGDGD- ---KFHYEDV PLIDT----- ------WRAL EKLV---EKG KIRS------
2C1_gi|1332539|     PGWYVGDAK- ---SLQFEQS PIHEC----- ------WAEL EKIV---DAG LARN------
2D1_gi|7407095|     PGWKSEKDE- ----LEFGNA TIQET----- ------WTAM ESLV---DKK LARS------
2E1_gi|4753912|     ---------- ---DYSHSNT DYLET----- ------WRAT EEMV---KLG YTKS------
2E2_gi|18479021|    ----NGDPV- --------DI DVLNT----- ------WNGM EEAK---KLG LAKS------
2E3_gi|5052610|     PTDAKGEVE- ---L---NDI DYLDT----- ------WREM EKLV---ELG LTKS------
3A1_gi|121087|      PTKKDGSRA- ----VDITNW NFIKT----- ------WELM QELP---KTG KTKA------
3A2_gi|6320576|     PTLEDGTVD- ----IDTKEW NFIKT----- ------WELM QELP---KTG KTKA------
3B1_gi|1142698|     PKANDKEVK- -----LDLEV SLVDT----- ------WKAM VKLL---DTG KVKA------
3B2_gi|60458781|    PADPTNKDL- ---AYVDDSV KLSDT----- ------WKAV VALK---KTG KTKS------
3B3_gi|60458785|    PTEPDNKEL- ---AAIDDSI KLVDT----- ------WKAV VALK---KTG KTKS------
3C1_gi|536474|      PVDDSGKTM- -----YAADG DYLET----- ------YKQL EKIYLDPNDH RVRA------
3C2_gi|74626610|    ---------- -------KKI PISEG----- ------WKAM ETAL---GTG LVHS------
3C3_gi|38423524|    ---------- -----QTHGY TLEQA----- ------WEAL VEAK---KAG KVRE------
3D1_gi|31321885|    PKHADGSRD- -----IIHTH NHVDT----- ------WKLM EKLP---ATG KTKA------
3E1_gi|22207641|    KIGPDGKYV- ---ILKDLTE NPEPT----- ------WRAM EKIY---EDR KARS------
3F1_gi|81625481|    ---------- ---------- -LEET----- ------LSAM AEGV---RQG LIRY------
3F2_gi|13638516|    ---------- ---------V SVEEF----- ------MQAL LEAK---KQG LTRE------
3F3_gi|81635765|    --EINGENA- ---------- ---RS----- ------LRLL QKAF---DIG LARN------
4A1_gi|112837|      SFPIEVEDL- -------LPF DVKGV----- ------WESM EECQ---KLG LTKA------
```

```
4A2_gi|75220959|     PIDVAD---- ------LLPF DVKGV----- ------WESM EESL---KLG LTKA------
4A3_gi|1215788|      SFPIQVEDL- -------LPF DVKGV----- ------WESM EECL---KLG LTKA------
4A4_gi|1514979|      SFPIQAEDL- -------LPF DVKGV----- ------WESM EESL---KLG LTKA------
4B1_gi|2792155|      VIFSKEDLL- --------PF DIEGT----- ------WKAM EECY---RLG LAKS------
4B2_gi|6478210|      VNEIPKDHI- -------LPM DYKSV----- ------WAAM EECQ---TLG FTRA------
4B3_gi|6478204|      VNEIPKDHI- -------LPM DYKSV----- ------WAAM EECQ---TLG FTRA------
4B4_gi|2792295|      TMPVERDLV- -------QPL DIKSV----- ------WEAM EECK---KLG LARG------
4B5_gi|112807104|    TAPFTPEDF- -------EPF DMRAV----- ------WEAM EECH---RLG LAKA------
4B6_gi|112807098|    KAPFTADDF- -------VPF DMRAV----- ------WEAM EECH---RLG LAKA------
4B7_gi|112807100|    KAPFTAEDF- -------VPF DMRAV----- ------WEAM EECH---RLG LAKA------
4B8_gi|112807102|    KAPFTADDF- -------VPF DMRAV----- ------WEAM EECH---RLG LAKA------
4C1_gi|113595|       MPPEAGEVL- --------EF DMEGV----- ------WKEM ENLV---KDG LVKD------
4C2_gi|167113|       KPPEAGEVL- --------EF DMEGV----- ------WKEM ENLV---KDG LVKD------
4C3_gi|75221432|     QPPEAGEVL- --------EF DMEGV----- ------WKEM EKLV---KDG LVKD------
4C4_gi|4539944|      MPPEAGEVL- --------EF DIGGV----- ------WREM EKLV---KVG LVRD------
4C5_gi|13160397|     GLDPEN---- ------FIPT DIPGT----- ------WKAM EALY---DSG KARA------
4C6_gi|13160399|     -VGLDPENF- -------VPT DIPGT----- ------WKAM EALY---DSG KARA------
4C8_gi|111182163|    PTPEM----- ------LTKP DITST----- ------WKAM EALY---DSG KARA------
4C9_gi|111182165|    -VGIKPENL- -------LPV DIPST----- ------WKAM EALY---DSG KARA------
4C10_gi|111182167|   -TGFKPENI- -------LPT DIPST----- ------WKAM ESLF---DSG KARA------
4C11_gi|111182169|   -VDFKPENI- -------MPI DIPST----- ------WKAM EALV---DSG KARA------
5A1_gi|408360251|    -----GKDI- ---L-SKEGK KYLDS----- ------WRAF EQLY---KEK KVRA------
5A2_gi|11127591|     ---------- ---------- --IDT----- ------WKAF EKLY---ADK KVRA------
5B1_gi|2506173|      ---------- ---------- ATIQS----- ------WKAA EKIL---GDG RARA------
5C1_gi|144969|       ---------- ---------- NYVHA----- ------WEKM IELR---AAG LTRS------
5C2_gi|3916039|      ---------- ---------- HYVEA----- ------WKGM IELQ---KEG LIKS------
5D1_gi|112735|       ---------- ---------- RWLDT----- ------WRGM IDAR---EAG LVRS------
5E1_gi|5354195|      ---------- ---------- LYVEA----- ------WQAL IEAR---KKG LIRS------
5F1_gi|82504416|     ---------- ---------- ---------- ------WRAM EELQ---QAG KIRA------
5G1_gi|16080393|     ---------- ---------- --KDT----- ------WRAL EKLY---KDG KIRA------
5G2_gi|16079957|     ---------- ---------- --KEA----- ------WRAL ETLY---KEG RIKA------
6A1_gi|18202524|     ---------- ---------- -MEET----- ------VRAM THVI---NQG MAMY------
6A2_gi|499328|       ---------- ---------- -MEET----- ------VRAM THVI---NQG MAMY------
6A3_gi|2135947|      ---------- ---------- -MEEI----- ------VRAM THVI---NQG MAMY------
6A4_gi|975314|       ---------- ---------- -MEET----- ------VRAM THVI---NQG MAMY------
6A5_gi|2827466|      ---------- ---------- -MEET----- ------VRAM THVI---NQG MAMY------
6A6_gi|7914984|      ---------- ---------- -MEET----- ------VRAM THVI---NQG MAMY------
6A7_gi|5019764|      ---------- ---------- -MEEI----- ------VRAM THVI---NQG MAMY------
6A8_gi|148747467|    ---------- ---------- -MEEI----- ------VRAM THVI---NQG MAMY------
6A9_gi|24648619|     VDNLQGSAL- ----VCLGPI IVKRM----- ------HDTM WEVR---DTT LELT------
6A10a_gi|726465|     ---------- ---------- -MEEI----- ------VRAM THVI---NQG MAMY------
6A10b_gi|5019766|    ---------- ---------- -MEEI----- ------VRAM THVI---NQG MAMY------
6A11_gi|5922729|     ---------- ---------- -MEEI----- ------VRAM TFVI---NQG MAMY------
6A13_gi|52001073|    ---------- ---------- -MEEI----- ------VRAM THVI---NQG MAMY------
6A14_gi|226823214|   ---------- ---------- -MEEI----- ------VRAM TYVI---NQG LALY------
6B1_gi|902000|       ---------- ---------- --MEV----- ------VRAM SYVI---QQG WAMY------
6C1_gi|1063415|      ---------- ---------- -IEEA----- ------VRAM NYVI---DKG WAFY------
6C2_gi|2832783|      ---------- ---------- -IEET----- ------VRAM NHVI---DRG WAFY------
7A1_gi|39932720|     ---------- ---------- -IEET----- ------LQAC HQLH---QEG KFVE------
7A2_gi|41327764|     ---------- ---------- -VEET----- ------LHAC QRLH---QEG KFVE------
7A3_gi|41152114|     ---------- ---------- -VEET----- ------LRAC HQLH---QEG KFVE------
7A4_gi|6815049|      ---------- ---------- -IVET----- ------LQAC QQLH---QEG KFVE------
8A1_gi|24638123|     ---------- ---------- -IETT----- ------MKTL KGFV---DSG KISC------
8A2_gi|74627022|     ---------- ---------- -IETT----- ------MKAL KAFV---DSG EISC------
9A1_gi|146345520|    ---------- ---------- -VEEV----- ------MQSL HHLV---AAG KVLN------
9A2_gi|6093525|      ---------- ---------- -VEEV----- ------MRSL NHLV---ANG KVLY------
9A3_gi|2492798|      ---------- ---------- -IEEV----- ------MNGL HNLV---AQG KVLY------
9B1_gi|6323998|      ---------- ---------- -IEEV----- ------MDSL HILV---QQG KVLY------
9B2_gi|6319951|      ---------- ---------- -IEEF----- ------MDSL HILV---QQG KVLY------
9B3_gi|6319958|      ---------- ---------- -IEEV----- ------MDSL HILV---QQG KVLY------
9B4_gi|6322615|      ---------- ---------- -IEEV----- ------MDSL HILV---QQG KVLY------
9C1_gi|2459734|      ---------- ---------- -TREM----- ------MKTL NGLV---EDG KVHY------
10A1_gi|4731595|     ---------- ---------- -VGEF----- ------VTVL AELV---ERG VIGG------
10A2_gi|3256056|     ---------- ---------- -VGEF----- ------VSVL ADLV---ARG LIGA------
11A1_gi|1176985|     ---------- ---------- -KDEA----- ------VNAL NEMK---KAG KIRS------
11B1_gi|3123233|     ---------- ---------- -IEET----- ------AEVM KELY---DAG KIRA------
11B2_gi|3123121|     ---------- ---------- -IAET----- ------VAVL NELK---SEG KIRA------
11B3_gi|85062654|    ---------- ------SDLV PIAET----- ------MAAL NQLK---EQG KIRA------
11C1_gi|81787577|    ---------- ---------- -IDET----- ------IEAF EELK---QEG VIRY------
12A1_gi|5305791|     ---------- ---------- -WDEL----- ------WQAM DQLV---ASG KVRY------
12B1_gi|2599278|     ---------- ---------- -WDEV----- ------WQAM DSLV---ASG KVSY------
12C1_gi|5921163|     ---------- ---------- -WDEV----- ------WQAM DLLI---TQG KVRY------
13A1_gi|1351673|     ---------- ---------- -IEKI----- ------MGAL KKCV---EAG KIRY------
13B1_gi|9106797|     ---------- ---------- -RAEQ----- ------FGAI REFI---DEG LIRH------
13C1_gi|81555851|    ---------- ---------- -IGEV----- ------AEVM QLLI---KEG KIKA------
14A1_gi|882530|      ---------- ---------- -MEET----- ------ASAL AHAV---QSG KALY------
15A1_gi|37196700|    --ADRHEFH- ------WNAL TRGGG----- ------FRAL TELR---AAG NIKG------
all2316_aldo/keto    ---------- ---------- -IAES----- ------LTPV KEAV---EAG LIRF------
```

```
                        901
1A1_gi|5174391|          -------LGL SN--FNSRQI DDIL---SVA SVRPAV---- ---------L -Q-----VEC
1A2_gi|1703236|          -------LGL SN--FSSRQI DDVL---SVA SVRPAV---- ---------L -Q-----VEC
1A3_gi|1703237|          -------LGL SN--FSSRQI DDVL---SVA SVRPAV---- ---------L -Q-----VEC
1A4_gi|10946870|         -------LGL SN--FNSRQI DDVL---SVA SVRPAV---- ---------L -Q-----VEC
1B1_gi|4502049|          -------IGI SN--FNHLQV EMIL-NKPGL KYKPAV---- ---------N -Q-----IEC
1B2_gi|1703235|          -------IGV SN--FNHLQI ERIL-NKPGL KYKPAV---- ---------N -Q-----IEC
1B3_gi|1351911|          -------IGV SN--FNPLQI ERIL-NKPGL KYKPAV---- ---------N -Q-----IEC
1B4_gi|6978491|          -------IGV SN--FNPLQI ERIL-NKPGL KYKPAV---- ---------N -Q-----IEC
1B5_gi|113594|           -------IGV SN--FNHLQV EKIL-NKPGL KYKPAV---- ---------N -Q-----IEC
1B6_gi|584742|           -------IGV SN--FNHLQV EKIL-NKPGL KYKPAV---- ---------N -Q-----IEV
1B7_gi|231525|           -------LGI SN--FNHFQI ERLL-NKPGL KHKPVT---- ---------N -Q-----IES
1B8_gi|6679791|          -------LGV SN--FNHFQI ERLL-NKPGL KHKPVT---- ---------N -Q-----VEC
1B9_gi|2114406|          -------LGV SN--FNHFQI ERIL-NKPGL KHKPVT---- ---------N -Q-----VEC
1B10_gi|223468663|       -------LGV SN--FSHFQI EKLL-NKPGL KYKPVT---- ---------N -Q-----VEC
1B12_gi|14330324|        -------IGI SN--FNHEQI ERLL-NKPGL KYKPVV---- ---------N -Q-----IEC
1B13_gi|15864567|        -------LGV SN--FNHFQI ERLL-NKPGL KHKPVT---- ---------N -Q-----VEC
1B14_gi|148540194|       -------LGV SN--FNHFQI ERLL-NKPGL KHKPVT---- ---------N -Q-----VEC
1B15_gi|51094822|        -------LGV SN--FNHFQI ERLL-NKPGL KYKPVT---- ---------N -Q-----VEC
1C1_gi|5453543|          -------IGV SN--FNRRQL EMIL-NKPGL KYKPVC---- ---------N -Q-----VEC
1C2_gi|4503285|          -------IGV SN--FNHRLL EMIL-NKPGL KYKPVC---- ---------N -Q-----VEC
1C3_gi|24497583|         -------IGV SN--FNRRQL EMIL-NKPGL KYKPVC---- ---------N -Q-----VEC
1C4_gi|308153631|        -------IGV SN--FNCRQL EMIL-NKPGL KYKPVC---- ---------N -Q-----VEC
1C5_gi|1352733|          -------IGV SN--FNRRQL EMIL-NKPGL KYKPVC---- ---------N -Q-----VEC
1C6_gi|13487925|         -------IGV SN--FNRRQL EKIL-KKPGL KYKPVC---- ---------N -Q-----VEC
1C7_gi|129896|           -------IGV SN--FNHKQL EKIL-NKPGL KYKPVC---- ---------N -Q-----VEC
1C8_gi|1709623|          -------IGV SN--FNRRQL EKIL-NKPGL KHRPVC---- ---------N -Q-----VEC
1C9_gi|118634|           -------IGV SN--FNCRQL ERIL-NKPGL KYKPVC---- ---------N -Q-----VEC
1C10a_gi|1345830|        -------LGV SN--FNRRQL ERIL-NKPGL KYKPVC---- ---------N -Q-----VEC
1C10b_gi|1706132|        -------LGV SN--FNRRQL ERIL-NKPGL KYKPVC---- ---------N -Q-----VEC
1C11_gi|1669605|         -------IGV SN--FNHKQL EKIL-NKPGL KYKPVC---- ---------N -Q-----VEC
1C12_gi|85719330|        -------IGV SN--FNHRQL ERIL-NKPGL KYKPVC---- ---------N -Q-----VEC
1C13_gi|171846276|       -------IGV SN--FNHRQL ERIL-NKPGL KYKPVC---- ---------N -Q-----VEC
1C14_gi|19527294|        -------IGV SN--FNFRQL ETIL-NKPGL KYKPVC---- ---------N -Q-----VEC
1C15_gi|741804|          -------IGV SN--FNLKQL ELIL-NKPGL KYKPTC---- ---------N -Q-----VEC
1C16_gi|741803|          -------IGV SN--FNHKGL ERLL-NKPGL KYKPVC---- ---------N -Q-----VEC
1C17_gi|741805|          -------IGV SN--FNHKQL ERLI-NKPGL KYKPVC---- ---------N -Q-----VEC
1C18_gi|1654715|         -------TGC CTAAAGATGA GCATGGAAAC TTAATA---- ---------T TT-----GAC
1C20_gi|16905111|        -------IGV CN--FNRRQL EKIL-SKPGL KYKPVC---- ---------N -Q-----VEC
1C21_gi|126090770|       -------IGV SN--FNYRQL EMIL-NKPGL KYKPVC---- ---------N -Q-----VEC
1C22_gi|38603389|        -------IGV SN--FNHRQL ERIL-NKPGL KYKPVC---- ---------N -Q-----VEC
1C23_gi|62856987|        -------IGV SN--FNRRQL EMIL-NKPGL KHKPVC---- ---------N -Q-----VEC
1C24_gi|84993586|        -------IGV SN--FNHKQL ERLL-NKPGL KYKPVC---- ---------N -Q-----VEC
1C25_gi|15216337|        -------IGV SN--FNRRQL EMIL-NNPGL KYKPVC---- ---------N -Q-----VEC
1D1_gi|5174695|          -------LGV SN--FNRRQL ELIL-NKPGL KHKPVS---- ---------N -Q-----VEC
1D2_gi|398962|           -------LGV SN--FNRRQL EVIL-NKPGL KYKPVT---- ---------N -Q-----VEC
1D3_gi|5689216|          -------LGV SN--FNRQQL ELLL-NKPGL KHKPVC---- ---------N -Q-----VEC
1E1_gi|1698718|          -------LGV SN--FNHEQL ERLL-DKPGL RVRPIT---- ---------N -Q-----IEC
1E2_gi|269849539|        -------IGV SN--FNHEQL ERLL-NKPGL RFKPLT---- ---------N -Q-----IEC
1G1_gi|17550248|         -------VGV SN--WNNDQI SRAL----AL GLTPVH---- ---------N SQ-----VEL
2A1_gi|134153|           -------IGL SN--YELFLT RDCL---AYS KIKPAV---- ---------S -Q-----FET
2A2_gi|1835701|          -------IGI SN--YDVYLT RDIL---SYS KIKPAV---- ---------N -Q-----IET
2B1_gi|401428|           -------IGV SN--FPGALL LDLL---RGA TIKPSV---- ---------L -Q-----VEH
2B2_gi|1351442|          -------LGI SN--FSGALI QDLL---RGA RIKPVA---- ---------L -Q-----IEH
2B3_gi|2492803|          -------IGV SN--VSGGLL EDLI---KAA RIKPAS---- ---------L -Q-----IEH
2B4_gi|1912051|          -------IGI SN--FTGALI YDLI---RGA TIKPAV---- ---------L -Q-----IEH
2B5_gi|3289019|          -------IGV SN--FPGALL LDLL---RGA TIKPAV---- ---------L -Q-----VEH
2B6_gi|6321895|          KSSDFLPIEL KE--AQNQGM EDLI-LVSEN KLDVVS---- ---------H SR-----PSW
2B7_gi|1912049|          -------IGI SN--FTGALI YDLI---RGA TIKPAV---- ---------L -Q-----IEH
2B8_gi|4103055|          -------IGI SN--FSGALI QDLL---RSA KIKPAV---- ---------L -Q-----IEH
2C1_gi|1332539|          -------IGV AN--FNCQAI LDLL---TYA RIKPAV---- ---------L -Q-----IEL
2D1_gi|7407095|          -------IGI SN--FSAQLV MDLL---RYA RIRPAT---- ---------L -Q-----IEH
2E1_gi|4753912|          -------IGL SN--FNKLQV ATVL---QEC TIKPVA---- ---------L -Q-----IEV
2E2_gi|18479021|         -------IGV SN--FDTALL DRLI---AGS NTVPAV---- ---------N -Q-----IEV
2E3_gi|5052610|          -------IGV SN--FNSEQL TRLL---ANC KIKPIH---- ---------N -Q-----IEC
3A1_gi|121087|           -------VGV SN--FSINNL KDLL-ASQGN KLTPAA---- ---------N -Q-----VEI
3A2_gi|6320576|          -------VGV SN--FSINNI KELL-ESPNN KVVPAT---- ---------N -Q-----IEI
3B1_gi|1142698|          -------IGV SN--FDAKMV DAII---EAT GVTPSV---- ---------N -Q-----IER
3B2_gi|60458781|         -------VGV SN--FSTRLV DLVE---EAS GERPAV---- ---------N -Q-----IEA
3B3_gi|60458785|         -------VGV SN--FTTDLV DLVE---KAS GERPAV---- ---------N -Q-----IEA
3C1_gi|536474|           -------IGV SN--FSIEYL ERLI---KEC RVKPTV---- ---------N -Q-----VET
3C2_gi|74626610|         -------VGV SN--FRIPDL EELL---KTS TITPRV---- ---------N -Q-----IEF
3C3_gi|38423524|         -------IGI SN--AAIPHL EKLFAASPSP EYYPVV---- ---------N -Q-----IEF
3D1_gi|31321885|         -------VGV SN--YSKAWL EQLL---PHA TTVPAV---- ---------N -Q-----VEN
3E1_gi|22207641|         -------IGV SN--WTIADL EKMS---KFA KVMPHA---- ---------N -Q-----IEI
3F1_gi|81625481|         -------IGV SN--FDRRLL EEAI---SKS QEPIVC---- ---------D -Q-----VKY
3F2_gi|13638516|         -------IGI SN--FTIPLM EKAI---AAV GAENIA---- ---------T NQ-----IEL
3F3_gi|81635765|         -------IGV SN--YTAPMM REAQ---SIV EAPLVT---- ---------N -Q-----VEF
4A1_gi|112837|           -------IGV SN--FSVKKL QNLL---SVA TIRPVV---- ---------D -Q-----VEM
```

```
4A2_gi|75220959|      -------IGV SN--FSVKKL ENLL---SVA TVLPAV---- ---------N -Q----VEM
4A3_gi|1215788|       -------IGV SN--FSVKKL QNLL---SVA TIRPAV---- ---------N -Q----VEM
4A4_gi|1514979|       -------IGV SN--FSVKKL QNLL---SVA TIRPAV---- ---------N -Q----VEM
4B1_gi|2792155|       -------IGI CN--YGTKKL TKLL---EIA TIPPAV---- ---------N -Q----VEM
4B2_gi|6478210|       -------IGV SN--FSCKKL QELM---AAA KIPPVV---- ---------N -Q----VEM
4B3_gi|6478204|       -------IGV CN--FSCKRL QELM---ETA NSPPVV---- ---------N -Q----VEM
4B4_gi|2792295|       -------IGV SN--FTSSML EELL---SFA EIPPAV---- ---------N -Q----LEM
4B5_gi|112807104|     -------IGV CN--FSCKKL ETLL---SFA TIPPVV---- ---------N -Q----VEI
4B6_gi|112807098|     -------IGV CN--FSCKKL DTLL---SFA TIPPAV---- ---------N -Q----VEV
4B7_gi|112807100|     -------IGV AN--FSCKKL DTLL---SFA TIPPTV---- ---------N -Q----VEV
4B8_gi|112807102|     -------IGV AN--FSCKKL DTLL---SFA TIPPTV---- ---------N -Q----VEV
4C1_gi|113595|        -------IGV CN--YTVTKL NRLL---RSA KIPPAV---- ---------C -Q----MEM
4C2_gi|167113|        -------IGV CN--YTVTKL NRLL---QSA KIAPAV---- ---------C -Q----MEM
4C3_gi|75221432|      -------IDV CN--FTVTKL NRLL---RSA NIPPAV---- ---------C -Q----MEM
4C4_gi|4539944|       -------IGI SN--FTVKKL EKLL---NFA EIKPSV---- ---------C -Q----MEM
4C5_gi|13160397|      -------IGV SN--FTLKKL SDLL---DVA RIPPAV---- ---------N -Q----VGC
4C6_gi|13160399|      -------IGV SN--FTLKKL SDLL---DVA RIPPAV---- ---------N -Q----VGC
4C8_gi|111182163|     -------IGV SN--FSSKKL TDLL---NVA RVTPAV---- ---------N -Q----VEC
4C9_gi|111182165|     -------IGV SN--FSTKKL ADLL---ELA RVPPAV---- ---------N -Q----VEC
4C10_gi|111182167|    -------IGV SN--FSSKKL ADLL---VVA RVPPAV---- ---------N -Q----VEC
4C11_gi|111182169|    -------IGV SN--FSTKKL SDLV---EAA RVPPAV---- ---------N -Q----VEC
5A1_gi|408360251|     -------IGV SN--FHIHHL EDVL---AMC TVTPMV---- ---------N -Q----VEL
5A2_gi|11127591|      -------IGV SN--FHEHHI EELL---KHC KVAPMV---- ---------N -Q----IEL
5B1_gi|2506173|       -------IGV CN--FLEDQL DELI---AAS DVVPAV---- ---------N -Q----IEL
5C1_gi|144969|        -------IGV SN--HLVPHL ERIV---AAT GVVPAV---- ---------N -Q----IEL
5C2_gi|3916039|       -------IGV CN--FQIHHL QRLI---DET GVTPVI---- ---------N -Q----IEL
5D1_gi|112735|        -------IGV SN--FTEPML KTLI---DET GVTPAV---- ---------N -Q----VEL
5E1_gi|5354195|       -------IGV CN--FLPEHL ERLI---KET GVTPVV---- ---------N -Q----VEL
5F1_gi|82504416|      -------IGV SN--FHPDRL ADLI---AFN HVVPAV---- ---------N -Q----IEV
5G1_gi|16080393|      -------IGV SN--FQVHHL EELL---KDA EIKPMV---- ---------N -Q----VEF
5G2_gi|16079957|      -------IGV SN--FQIHHL EDLM---TAA EIKPMI---- ---------N -Q----VEF
6A1_gi|18202524|      -------WGT SR--WSSMEI MEAYSVARQF NLIPPI---- ---------C EQ----AEY
6A2_gi|499328|        -------WGT SR--WSSMEI MEAYSVARQF NLIPPI---- ---------C EQ----AEY
6A3_gi|2135947|       -------WGT SR--WSAMEI MEAYSVARQF NMIPPV---- ---------C EQ----AEY
6A4_gi|975314|        -------WGT SR--WSSMEI MEAYSVARQF NLIPPI---- ---------C EQ----AEY
6A5_gi|2827466|       -------WGT SR--WSSMEI MEAYSVARQF NLTPPI---- ---------C EQ----AEY
6A6_gi|7914984|       -------WGT SR--WSSMEI MEAYSVARQF NLIPPI---- ---------C EQ----AEY
6A7_gi|5019764|       -------WGT SR--WSAMEI MEAYSVARQF NMIPPV---- ---------C EQ----AEY
6A8_gi|148747467|     -------WGT SR--WSAMEI MEAYSVARQF NMIPPV---- ---------C EQ----AEY
6A9_gi|24648619|      -------TSI AS--ISRIKF PAFQRFLIDS KIPPIVYEMA KNDSEVYVRA SA----FQC
6A10a_gi|726465|      -------WGT SR--WSAMEI MEAYSVARQF NMIPPV---- ---------C EQ----AEY
6A10b_gi|5019766|     -------WGT SR--WSAMEI MEAYSVARQF NMIPPV---- ---------C EQ----AEY
6A11_gi|5922729|      -------WGT SR--WSAMEI MEAYSVARQF NLIPPV---- ---------C EQ----AEY
6A13_gi|52001073|     -------WGT SR--WSAMEI MEAYSVARQF NMIPPV---- ---------C EQ----AEY
6A14_gi|226823214|    -------WGT SR--WSAAEI MEAYSMARQF NLIPPV---- ---------C EQ----AEN
6B1_gi|902000|        -------WGT AR--WSQVEI MEAYTNCRQF NCITPI---- ---------V EQ----SEY
6C1_gi|1063415|       -------WGI SE--WSAQQI TEAWGAADRL DLVGPI---- ---------V EQ----PEY
6C2_gi|2832783|       -------WGT SE--WSSQQI TEPWAAAENL DLVGPI---- ---------V EQ----PEY
7A1_gi|39932720|      -------LGL SN--YVSWEV AEICTLCKKN GWIMPT---- ---------V YQ----GMY
7A2_gi|41327764|      -------LGL SN--YASWEV AEICTLCKSN GWILPT---- ---------V YQ----GMY
7A3_gi|41152114|      -------LGL SN--YAAWEV AEICTLCKSN GWILPT---- ---------V YQ----GMY
7A4_gi|6815049|       -------LGL SN--YASWEV AEIYTLCKSN GWILPT---- ---------V YQ----GMY
8A1_gi|24638123|      -------VGL SE--VSAETI KRAH------ AVVPIA---- ---------A VE----VEY
8A2_gi|74627022|      -------VGL SE--ASAESI KRAL       AIVPIA---- ---------A VE----TEY
9A1_gi|146345520|     -------IGI SD--APAWVV AKCNEYARFH GLTRFC---- ---------V YQ----GRW
9A2_gi|6093525|       -------LGV SD--TPAWLV VKCNAFARAN GLTPFS---- ---------V YQ----GHW
9A3_gi|2492798|       -------LGV SD--TPAWVV SKANNYARMA GKTPFV---- ---------I YE----GEW
9B1_gi|6323998|       -------LGV SD--TPAWVV SAANYYATSH GKTPFS---- ---------V YQ----GKW
9B2_gi|6319951|       -------LGV SD--TPAWVV SAANYYATSY GKTPFS---- ---------I YQ----GKW
9B3_gi|6319958|       -------LGV SD--TPAWVV SAANYYATSH GKTPFS---- ---------I YQ----GKW
9B4_gi|6322615|       -------LGV SD  TPAWVV SAANYYATSH GKTPFS---- ---------I YQ----GKW
9C1_gi|2459734|       -------LGA STLRPNAWKV ARANEIARAE GWEPFT---- ---------V AQ----PRY
10A1_gi|4731595|      -------YGM SN--WPLVRV QDAVSYAHVH DLVPPT---- ---------G VSNQFSLIDM
10A2_gi|3256056|      -------YGM SN--WSLDRV REAVGYAREQ GLPAPA---- ---------G VSNQLSLIDM
11A1_gi|1176985|      -------IGV SN--FSLEQL KEAN----KD GLVDVL---- ---------- -Q----GEY
11B1_gi|3123233|      -------IGV SN--FSIEQM DTFRAVAPLH TIQPPY---- ---------N -L----FER
11B2_gi|3123121|      -------IGA AN--VDADHI REYL---QYG ELDIIQ---- ---------A -K----YSI
11B3_gi|85062654|     -------IGV SN--FSLAQL QEAMEHGQID SIQPPY---- ---------S -L-------
11C1_gi|81787577|     -------YGI SS--IRPNVI KEYL---KRS NIVSIM---- ---------M -Q----YSI
12A1_gi|5305791|      -------IGS SN--FAGWHL AAGQESAARR GSLGLV---- ---------S EQ----CLY
12B1_gi|2599278|      -------VGS SN--FAGWHI AAAQENAARR HSLGMV---- ---------S HQ----CLY
12C1_gi|5921163|      -------VGS SN--FAGWDI ASAQEAARRR NALGLA---- ---------S EQ----CVY
13A1_gi|1351673|      -------IGL SE--CSANTI RRAA------ AVYPVS---- ---------A VQ----VEY
13B1_gi|9106797|      -------AGL SQ--VSVEAI EEAR------ KVFPVA---- ---------T VQ----NRY
13C1_gi|81555851|     -------WGM SE--AGLSSI QKAH------ QICPLS---- ---------A LQ----SEY
14A1_gi|882530|       -------VGI SS--YSPERT QKMVELLREW KIPLLI---- ---------H -Q----PSY
15A1_gi|37196700|     -------FGL G---VNEWQI IRDALEEADL DCSLLA---- ---------G -R----YSL
all2316_aldo/keto     -------VGV SN--FSVEQI KQAR------ DVVDIV---- ---------S VQ----NQY
```

```
                          961
1A1_gi|5174391|           HP--Y----- ----LAQN-- ELIAH----- -------CQA R-GLEVTA-- ----------
1A2_gi|1703236|           HP--Y----- ----LAQN-- ELIAH----- -------CQA R-GLEVTA-- ----------
1A3_gi|1703237|           HP--Y----- ----LAQN-- ELIAH----- -------CQA R-GLEVTA-- ----------
1A4_gi|10946870|          HP--Y----- ----LAQN-- ELIAH----- -------CHA R-GLEVTA-- ----------
1B1_gi|4502049|           HP--Y----- ----LTQE-- KLIQY----- -------CQS K-GIVVTA-- ----------
1B2_gi|1703235|           HP--Y----- ----LTQE-- KLIQY----- -------CHS K-GIVVTA-- ----------
1B3_gi|1351911|           HP--Y----- ----LTQE-- KLIEY----- -------CHS K-GIVVTA-- ----------
1B4_gi|6978491|           HP--Y----- ----LTQE-- KLIEY----- -------CHC K-GIVVTA-- ----------
1B5_gi|113594|            HP--Y----- ----LTQE-- KLIQY----- -------CNS K-GIVVTA-- ----------
1B6_gi|584742|            HP--Y----- ----LTQE-- KLIEY----- -------CKS K-GIVVTA-- ----------
1B7_gi|231525|            HP--Y----- ----LTQE-- KLIQY----- -------CQS K-GIAVTA-- ----------
1B8_gi|6679791|           HP--Y----- ----LTQE-- KLIQY----- -------CHS K-GISVTA-- ----------
1B9_gi|2114406|           HP--Y----- ----LTQE-- KLIEY----- -------CHS K-GITVTA-- ----------
1B10_gi|223468663|        HP--Y----- ----LTQE-- KLIQY----- -------CHS K-GITVTA-- ----------
1B12_gi|14330324|         HP--Y----- ----LTQE-- KLIKY----- -------CHS K-GIAVTA-- ----------
1B13_gi|15864567|         HP--Y----- ----LTQE-- KLIQY----- -------CHS K-GIVVTA-- ----------
1B14_gi|148540194|        HP--Y----- ----LTQE-- KLIQY----- -------CHS K-GIAVIA-- ----------
1B15_gi|51094822|         HP--Y----- ----LTQE-- KLIQY----- -------CHS K-GITVTA-- ----------
1C1_gi|5453543|           HP--Y----- ----FNQR-- KLLDF----- -------CKS K-DIVLVA-- ----------
1C2_gi|4503285|           HP--Y----- ----FNQR-- KLLDF----- -------CKS K-DIVLVA-- ----------
1C3_gi|24497583|          HP--Y----- ----FNRS-- KLLDF----- -------CKS K-DIVLVA-- ----------
1C4_gi|308153631|         HP--Y----- ----LNQS-- KLLDF----- -------CKS K-DIVLVA-- ----------
1C5_gi|1352733|           HP--Y----- ----LNQG-- KLLEF----- -------CKS K-GIVLVA-- ----------
1C6_gi|13487925|          HP--Y----- ----LNQG-- KLLDF----- -------CRS K-DIVLVA-- ----------
1C7_gi|129896|            HP--Y----- ----LNQS-- KLLEF----- -------CKS H-DIVLVA-- ----------
1C8_gi|1709623|           HL--Y----- ----LNQS-- KLLAY----- -------CKM N-DIVLVA-- ----------
1C9_gi|118634|            HL--Y----- ----LNQS-- KMLDY----- -------CKS K-DIILVS-- ----------
1C10a_gi|1345830|         HV--Y----- ----LNQN-- KLHSY----- -------CKS K-DIVLVT-- ----------
1C10b_gi|1706132|         HV--Y----- ----LNQN-- KLHSY----- -------CKS K-DIVLVT-- ----------
1C11_gi|1669605|          HP--Y----- ----FNQS-- KLLDF----- -------CKS H-DIVLVA-- ----------
1C12_gi|85719330|         HL--Y----- ----LNQS-- KLLDY----- -------CKS K-DIVLVA-- ----------
1C13_gi|171846276|        HL--Y----- ----LNQR-- KLLDY----- -------CES K-DIVLVA-- ----------
1C14_gi|19527294|         HL--Y----- ----LNQS-- QMLDY----- -------CKS K-DIILVS-- ----------
1C15_gi|741804|           HP--Y----- ----LNQS-- KLLEF----- -------CKS K-DIVLVA-- ----------
1C16_gi|741803|           HL--Y----- ----LNQS-- KLLDY----- -------CKS K-DIVLVA-- ----------
1C17_gi|741805|           HL--Y----- ----MNQS-- KLLDY----- -------CKS K-DIVLVA-- ----------
1C18_gi|1654715|          AC--CGTGGA ATCCCTGT-- GACAC----- -------CTT GGGAAGCC-- ----------
1C20_gi|16905111|         HP--Y----- ----LNQR-- KLLDF----- -------CRS K-DIVLVA-- ----------
1C21_gi|126090770|        HP--Y----- ----LNQM-- KLLDF----- -------CKS K-DIVLVA-- ----------
1C22_gi|38603389|         HL--Y----- ----LNQR-- KLLDY----- -------CES K-DIVLVA-- ----------
1C23_gi|62856987|         HP--Y----- ----LNQS-- KLLDF----- -------CKS K-DIVLVA-- ----------
1C24_gi|84993586|         HL--Y----- ----LNQS-- KLLDY----- -------CKS K-DIVLVA-- ----------
1C25_gi|15216337|         HP--Y----- ----LNQS-- KLLDF----- -------CKS K-DIVLVA-- ----------
1D1_gi|5174695|           HP--Y----- ----FTQP-- KLLKF----- -------CQQ H-DIVITA-- ----------
1D2_gi|398962|            HP--Y----- ----FTQT-- KLLEV----- -------SAS SMTSFIVA-- ----------
1D3_gi|5689216|           HP--Y----- ----FTQP-- KLLKF----- -------CQQ H-DIIIVA-- ----------
1E1_gi|1698718|           HP--Y----- ----LNQK-- KLIDF----- -------CHK R-NVSVTA-- ----------
1E2_gi|269849539|         HP--Y----- ----LTQK-- NLISF----- -------CQS R-DVSVTA-- ----------
1G1_gi|17550248|          HL--Y----- ----FPQH-- DHVDF----- -------CKK H-NISVTS-- ----------
2A1_gi|134153|            HP--Y----- ----FQRD-- SLVKF----- -------CMK H-GVLPTA-- ----------
2A2_gi|1835701|           HP--Y----- ----FQRD-- SLIKF----- -------CQK Y-GIAITA-- ----------
2B1_gi|401428|            HP--Y----- ----LQQP-- RLIEF----- -------AQS R-GIAVTA-- ----------
2B2_gi|1351442|           HP--Y----- ----LTQE-- RLIKY----- -------VKN A-GIQVVA-- ----------
2B3_gi|2492803|           HP--Y----- ----LQQN-- KLVEY----- -------AQL K-GIVVTG-- ----------
2B4_gi|1912051|           HP--Y----- ----LQQP-- KLIEY----- -------VQK A-GIAITG-- ----------
2B5_gi|3289019|           HP--Y----- ----LQQP-- KLIEF----- -------AQK A-GVTITA-- ----------
2B6_gi|6321895|           LP--P----- ----KDRQEK KLHER----- -------QIN K-SMSVAS-- ----------
2B7_gi|1912049|           HP--Y----- ----LQQP-- KLIEY----- -------VQK A-GIAITG-- ----------
2B8_gi|4103055|           HP--Y----- ----LQQP-- RLVEY----- -------VQS Q-GIAITA-- ----------
2C1_gi|1332539|           HP--Y----- ----LPQE-- RLVKW----- -------VKE Q-GIQITA-- ----------
2D1_gi|7407095|           HP--Y----- ----LTQT-- RLVEY----- -------AQK E-GLTVTA-- ----------
2E1_gi|4753912|           HP--Q----- ----IIQE-- DLITY----- -------AKD E-GIIVMG-- ----------
2E2_gi|18479021|          HP--S----- ----KTQE-- KLVAD----- -------SHE R-GIEVMA-- ----------
2E3_gi|5052610|           HP--A----- ----LNQK-- KLIAL----- -------CKK N-DIVVTA-- ----------
3A1_gi|121087|            HP--L----- ----LPQD-- ELINF----- -------CKS K-GIVVEA-- ----------
3A2_gi|6320576|           HP--L----- ----LPQD-- ELIAF----- -------CKE K-GIVVEA-- ----------
3B1_gi|1142698|           HP--L----- ----LLQP-- ELIAH----- -------HKA K-NIHITA-- ----------
3B2_gi|60458781|          HP--L----- ----LQQD-- ELVAH----- -------HKS K-NIVITA-- ----------
3B3_gi|60458785|          HP--L----- ----LQQD-- ELVAH----- -------HKS K-NIVITA-- ----------
3C1_gi|536474|            HP--H----- ----LPQM-- ELRKF----- -------CFM H-DILLTA-- ----------
3C2_gi|74626610|          HPQVY----- ----KAAK-- PLVEF----- -------CQS K-GIIVEG-- ----------
3C3_gi|38423524|          HP--F----- ----LQNQSK NIVRF----- -------CQE H-GILVEA-- ----------
3D1_gi|31321885|          HP--Q----- ----LPQQ-- ELVDF----- -------CKE K-GIHIMA-- ----------
3E1_gi|22207641|          HP--F----- ----LPNE-- ELVQY----- -------CFS K-NIMPVA-- ----------
3F1_gi|81625481|          NI--EDRD-- ----PERD-- GLLEF----- -------CQK N-GVTLVA-- ----------
3F2_gi|13638516|          SP--Y----- ----LQNR-- KVVAW----- -------AKQ H-GIHITS-- ----------
3F3_gi|81635765|          HP--L----- ----IDQS-- RLLDA----- -------AEE T-KIALSS-- ----------
4A1_gi|112837|            NL--A----- ----WQQK-- KLREF----- -------CKE N-GIIVTA-- ----------
```

```
4A2_gi|75220959|      NL--A----- ----WQQK-- KLREF----- -------CNA H-GIVLTA-- ----------
4A3_gi|1215788|       NL--A----- ----WQQK-- KLREF----- -------CTA N-GIVLTA-- ----------
4A4_gi|1514979|       NL--A----- ----WQQK-- KLREF----- -------CNA N-GIVLTA-- ----------
4B1_gi|2792155|       NP--S----- ----WQQG-- NLREF----- -------CKQ K-GIHVSA-- ----------
4B2_gi|6478210|       SP--T----- ----LHQK-- NLREY----- -------CKA N-NIMITA-- ----------
4B3_gi|6478204|       SP--T----- ----LHQK-- NLREY----- -------CKA N-NIMITA-- ----------
4B4_gi|2792295|       NP--A----- ----WQLK-- KLRDF----- -------CKA K-GIHVTA-- ----------
4B5_gi|112807104|     NP--V----- ----WQQR-- KLREF----- -------CRA K-GIQLCA-- ----------
4B6_gi|112807098|     NP--V----- ----WQQR-- KLREL----- -------CRE K-GVQICA-- ----------
4B7_gi|112807100|     NP--V----- ----WQQR-- KLREF----- -------CRG K-GIQLCA-- ----------
4B8_gi|112807102|     NP--V----- ----WQQR-- KLREF----- -------CRG K-GIQLCA-- ----------
4C1_gi|113595|        HP--G----- ----WKND-- KIFEA----- -------CKK H-GIHVTA-- ----------
4C2_gi|167113|        HP--G----- ----WKND-- KILEA----- -------CKK H-GIHATA-- ----------
4C3_gi|75221432|      HP--G----- ----WKND-- KIFEA----- -------CKK H-GIHVTA-- ----------
4C4_gi|4539944|       HP--G----- ----WRKH-- KMFEI----- -------CRK Y-GIHTTA-- ----------
4C5_gi|13160397|      HP--S----- ----CAQT-- KLRAF----- -------CKS K-GVHLSG-- ----------
4C6_gi|13160399|      HP--S----- ----CAQT-- KLRAF----- -------CKS K-GIHLSG-- ----------
4C8_gi|111182163|     HP--V----- ----WQQQ-- GLHEL----- -------CKS K-GVHLSG-- ----------
4C9_gi|111182165|     HP--S----- ----WRQT-- KLQEF----- -------CKS K-GVHLSA-- ----------
4C10_gi|111182167|    HP--S----- ----WQQN-- VLRDF----- -------CKS K-GVHLSG-- ----------
4C11_gi|111182169|    HP--S----- ----WQQH-- KLHEF----- -------CKS K-GIHLSG-- ----------
5A1_gi|408360251|     HP--L----- ----NNQA-- DLRAF----- -------CDA K-QIKVEA-- ----------
5A2_gi|11127591|      HP--L----- ----LNQK-- ALCEY----- -------CKS K-NIAVTA-- ----------
5B1_gi|2506173|       HP--Y----- ----FAQK-- PLLAK----- -------NRA L-GIVTEA-- ----------
5C1_gi|144969|        HP--A----- ----YQQR-- EITDW----- -------AAA H-DVKIES-- ----------
5C2_gi|3916039|       HP--L----- ----MQQR-- QLHAW----- -------NAT H-KIQTES-- ----------
5D1_gi|112735|        HP--Y----- ----FPQA-- ALRAF----- -------HDE H-GIRTES-- ----------
5E1_gi|5354195|       HP--Y----- ----FPQE-- EQRAW----- -------DKA H-GIVTES-- ----------
5F1_gi|82504416|      NP--F----- ----NQQL-- QAVPW----- -------NQS R-GIQPEA-- ----------
5G1_gi|16080393|      HP--R----- ----LTQK-- ELRDY----- -------CKG Q-GIQLEA-- ----------
5G2_gi|16079957|      HP--R----- ----LTQK-- ELIRY----- -------CQN Q-GIQMEA-- ----------
6A1_gi|18202524|      HM--FQRE-- ----KVEV-- QLPEL----- -------FHK I-GVGAMT-- ----------
6A2_gi|499328|        HM--FQRE-- ----KVEV-- QLPEL----- -------FHK I-GVGAMT-- ----------
6A3_gi|2135947|       HL--FQRE-- ----KVEV-- QLPEL----- -------YHK I-GVGAMT-- ----------
6A4_gi|975314|        HM--FQRE-- ----KVEV-- QLPEL----- -------FHK I-GVGAMT-- ----------
6A5_gi|2827466|       HM--FQRE-- ----KVEV-- QLPEL----- -------FHK I-GVGAMT-- ----------
6A6_gi|7914984|       HM--FQRE-- ----KVEV-- QLPEL----- -------FHK I-GVGAMT-- ----------
6A7_gi|5019764|       HL--FQRE-- ----KVEV-- QLPEL----- -------YHK I-GVGAMT-- ----------
6A8_gi|148747467|     HL--FQRE-- ----KVEV-- QLPEL----- -------YHK I-GVGAMT-- ----------
6A9_gi|24648619|      LSQMVSINLL WENGLSQL-- DLVDHLLFVM YRETNDIVRS EAVITLMKIY EHRKIHEKYK
6A10a_gi|726465|      HL--FQRE-- ----KVEV-- QLPEL----- -------YHK I-GVGAMT-- ----------
6A10b_gi|5019766|     HL--FQRE-- ----KVEV-- QLPEL----- -------YHK I-GVGAMT-- ----------
6A11_gi|5922729|      HL--FQRE-- ----KVET-- QLPEL----- -------YHK I-GVGSMT-- ----------
6A13_gi|52001073|     HL--FQRE-- ----KVEV-- QLPEL----- -------YHK I-GVGAMT-- ----------
6A14_gi|226823214|    HF--FQRE-- ----KVEM-- QLPEL----- -------YHK I-GVGSVT-- ----------
6B1_gi|902000|        HM--FCRE-- ----KCEL-- YLPEM----- -------YNK I-GVGLMA-- ----------
6C1_gi|1063415|       NM--FARH-- ----KVET-- EFLPL----- -------YTN H-GIGLTT-- ----------
6C2_gi|2832783|       NL--LSRH-- ----KVEA-- EYLPL----- -------YEK Y-GIGLTT-- ----------
7A1_gi|39932720|      NA--I----- ----TRQVET ELFP------ -------CLR HFGLRFYA-- ----------
7A2_gi|41327764|      NA--T----- ----TRQVET ELFP------ -------CLR HFGLRFYA-- ----------
7A3_gi|41152114|      NA--I----- ----TRQVET ELFP------ -------CLR HFGLRFYA-- ----------
7A4_gi|6815049|       NA--T----- ----TRQVET ELLP------ -------CLR YFGLRFYA-- ----------
8A1_gi|24638123|      SL--FSRD-- ----IETN   GIMDI----- -------CRK L-SIPIIA-- ----------
8A2_gi|74627022|      SL--FSRD-- ----IEKN   GILDT----- -------CTQ L-SIPIIA-- ----------
9A1_gi|146345520|     AC--S----- ----YRDFER EILPM----- -------CQS E-GLALAP-- ----------
9A2_gi|6093525|       SC--A----- ----FRDFER DILPM----- -------CES E-GMGLAP-- ----------
9A3_gi|2492798|       NI--T----- ----MRDMER DIIPM----- -------CIH E-GMAIAP-- ----------
9B1_gi|6323998|       NV--L----- ----NRDFER DIIPM----- -------ARH F-GMALAP-- ----------
9B2_gi|6319951|       NV--L----- ----NRDFER DIIPM----- -------ARH F-GMALAP-- ----------
9B3_gi|6319958|       NV--L----- ----NRDFER DIIPM----- -------ARH F-GMALAP-- ----------
9B4_gi|6322615|       NV--L----- ----NRDFER DIIPM----- -------ARH F-GMALAP-- ----------
9C1_gi|2459734|       NL--V----- ----DREIEG DYLEM----- -------TRS Y-GIAVCP-- ----------
10A1_gi|4731595|      VRPIYPGT-- ----LSAKDP RWRSW----- -------LAE N-RMCLYP-- ----------
10A2_gi|3256056|      VQPIYPGT-- ----MGSHDP RWRAW----- -------LTE N-PVTLYP-- ----------
11A1_gi|1176985|      NL--L----- ----NREAEK TFFPY----- -------TKE H-NISFIP-- ----------
11B1_gi|3123233|      EM-------- ------EE-- SVLPY----- -------AKD N-KITTLL-- ----------
11B2_gi|3123121|      LD--R----- ----AMEN-- ELLPL----- -------CRD N-GIVVQV-- ----------
11B3_gi|85062654|     ----F----- ----WRAIER EIQPF----- -------CAA Q-QISILA-- ----------
11C1_gi|81787577|     LD-------- ----RRPE-- EWFPL----- -------IQE H-GVSVVV-- ----------
12A1_gi|5305791|      NL--A----- ----VRHAEL EVLPA----- -------ARA Y-GIGVFA-- ----------
12B1_gi|2599278|      NL--A----- ----VRHAEL EVLPA----- -------AQA Y-GLGVFA-- ----------
12C1_gi|5921163|      NL--V----- ----TRHAEL EVIPA----- -------ASA Y-GVGVLV-- ----------
13A1_gi|1351673|      SP--FSLE-- ----IERPEI GVMKA----- -------CRE N-NITIVC-- ----------
13B1_gi|9106797|      NL-------- ----ADRADE DVLDY----- -------CEA N-GIGFIP-- ----------
13C1_gi|81555851|     SL--W----- ----WREPEK EILGF----- -------LEK E-KIGFVA-- ----------
14A1_gi|882530|       NL--LNRW-- ----VDKS-- GLLDT----- -------LQN N-GVGCIA-- ----------
15A1_gi|37196700|     LD--Q----- ----VSEK-- EFLPL----- -------AQK R-GMALVI-- ----------
all2316_aldo/keto     SP--WERQ-- ----PEKD-- GVLQY----- -------CEQ E-KLTFLP-- ----------
```

```
                       1021
1A1_gi|5174391|        --------YS PL------GS S-DRA----- ---------- ---------- ---------W
1A2_gi|1703236|        --------YS PL------GS S-DRA----- ---------- ---------- ---------W
1A3_gi|1703237|        --------YS PL------GS S-DRA----- ---------- ---------- ---------W
1A4_gi|10946870|       --------YS PL------GS S-DRA----- ---------- ---------- ---------W
1B1_gi|4502049|        --------YS PL------GS P-DRP----- ---------- ---------- ---------W
1B2_gi|1703235|        --------YS PL------GS P-DRP----- ---------- ---------- ---------W
1B3_gi|1351911|        --------YS PL------GS P-DRP----- ---------- ---------- ---------W
1B4_gi|6978491|        --------YS PL------GS P-DRP----- ---------- ---------- ---------W
1B5_gi|113594|         --------YS PL------GS P-DRP----- ---------- ---------- ---------W
1B6_gi|584742|         --------YS PL------GS P-DRP----- ---------- ---------- ---------W
1B7_gi|231525|         --------YS PL------GS P-DRP----- ---------- ---------- ---------Y
1B8_gi|6679791|        --------YS PL------GS P-DRP----- ---------- ---------- ---------S
1B9_gi|2114406|        --------YS PL------GS P-NRP----- ---------- ---------- ---------W
1B10_gi|223468663|     --------YS PL------GS P-DRP----- ---------- ---------- ---------W
1B12_gi|14330324|      --------YS PL------GS P-NRP----- ---------- ---------- ---------W
1B13_gi|15864567|      --------YS PL------GS P-DRP----- ---------- ---------- ---------R
1B14_gi|148540194|     --------YS PL------GS P-DRP----- ---------- ---------- ---------Y
1B15_gi|51094822|      --------YS PL------GS P-DRP----- ---------- ---------- ---------W
1C1_gi|5453543|        --------YS AL------GS HREEP----- ---------- ---------- ---------W
1C2_gi|4503285|        --------YS AL------GS HREEP----- ---------- ---------- ---------W
1C3_gi|24497583|       --------YS AL------GS QRDKR----- ---------- ---------- ---------W
1C4_gi|308153631|      --------HS AL------GT QRHKL----- ---------- ---------- ---------W
1C5_gi|1352733|        --------YS AL------GS HREPE----- ---------- ---------- ---------W
1C6_gi|13487925|       --------YS AL------GS HREKQ----- ---------- ---------- ---------W
1C7_gi|129896|         --------YA AL------GA QLLSE----- ---------- ---------- ---------W
1C8_gi|1709623|        --------YG AL------GT QRYKY----- ---------- ---------- ---------C
1C9_gi|118634|         --------YC TL------GS SRDKT----- ---------- ---------- ---------W
1C10a_gi|1345830|      --------YS VL------GS HRDRN----- ---------- ---------- ---------W
1C10b_gi|1706132|      --------YS VL------GS HRDRN----- ---------- ---------- ---------W
1C11_gi|1669605|       --------YG AL------GS QRLKE----- ---------- ---------- ---------W
1C12_gi|85719330|      --------YG AL------GT QRYKE----- ---------- ---------- ---------W
1C13_gi|171846276|     --------YG AL------GT QRYKE----- ---------- ---------- ---------W
1C14_gi|19527294|      --------YC TL------GS SRDKI----- ---------- ---------- ---------W
1C15_gi|741804|        --------YS AL------GS HRDSS----- ---------- ---------- ---------W
1C16_gi|741803|        --------YG AL------GT QRYKE----- ---------- ---------- ---------W
1C17_gi|741805|        --------YG AL------GT QRYKE----- ---------- ---------- ---------W
1C18_gi|1654715|       --------AT GAAGAAGTGT AAGGA----- ---------- ---------- -TGCAGGGCT
1C20_gi|16905111|      --------HS AL------GS NRDKE----- ---------- ---------- ---------W
1C21_gi|126090770|     --------YG VL------GT QRYGG----- ---------- ---------- ---------W
1C22_gi|38603389|      --------YG AL------GT QRYKE----- ---------- ---------- ---------W
1C23_gi|62856987|      --------YS AL------GS QREH------ ---------- ---------- ---------W
1C24_gi|84993586|      --------YG AL------GT QRYKE----- ---------- ---------- ---------W
1C25_gi|15216337|      --------HS AL------GT QRHKL----- ---------- ---------- ---------W
1D1_gi|5174695|        --------YS PL------GT SRNPI----- ---------- ---------- ---------W
1D2_gi|398962|         --------YS PL------GT CRNPL----- ---------- ---------- ---------W
1D3_gi|5689216|        --------YS PL------GT CRNPM----- ---------- ---------- ---------W
1E1_gi|1698718|        --------YR PL------GG SGGGF----- ---------- ---------- ----------
1E2_gi|269849539|      --------YR PL------GG SCEGV----- ---------- ---------- ----------
1G1_gi|17550248|       --------YA TL------GS PGRVN----- ---------- ---------F TLPTGQKLDW
2A1_gi|134153|         --------HT PLG-----GA AANKD----- ---------- ---------- ----------
2A2_gi|1835701|        --------HT PLG-----GA LANTE----- ---------- ---------- ----------
2B1_gi|401428|         --------YS SF------GP QSFVE----- ---------- ---------- ------LNQG
2B2_gi|1351442|        --------YS SF------GP VSFLE----- ---------- ---------- ------LENK
2B3_gi|2492803|        --------YS NF------GP LSFLE----- ---------- ---------- ---------L
2B4_gi|1912051|        --------YS SF------GP QSFLE----- ---------- ---------- ------LESK
2B5_gi|3289019|        --------YS SF------GP QSFVE----- ---------- ---------- ------MNQG
2B6_gi|6321895|        --------LD QL------GK NKDREEKLIR DETNRQKYVL LLDRDITRNS SLQSLSKMVW
2B7_gi|1912049|        --------YS SF------GP QSFLE----- ---------- ---------- ------LESK
2B8_gi|4103055|        --------YS SF------GP QSFVE----- ---------- ---------- ---------L
2C1_gi|1332539|        --------YS SF------GP TSYVD----- ---------- ---------- ---------L
2D1_gi|7407095|        --------YS SF------GP LSFLE----- ---------- ---------- ---------L
2E1_gi|4753912|        --------YS PF------GS LVKRF----- ---------- ---------- ---------G
2E2_gi|18479021|       --------YS PF------GF YVSRG----- ---------- ---------- ----------
2E3_gi|5052610|        --------YC PL------GR PNPAE----- ---------- ---------- ----------
3A1_gi|121087|         --------YS PL------GS T--------- ---------- ---------- ----------
3A2_gi|6320576|        --------YS PF------GS A--------- ---------- ---------- ----------
3B1_gi|1142698|        --------YS PL------GN NTVGA----- ---------- ---------- ----------
3B2_gi|60458781|       --------YS PL------GN NVAGK----- ---------- ---------- ----------
3B3_gi|60458785|       --------YS PL------GN NVSGK----- ---------- ---------- ----------
3C1_gi|536474|         --------YS PL------GS H--------- ---------- ---------- ----------
3C2_gi|74626610|       --------YG PL------SP LVRDA----- ---------- ---------- ----------
3C3_gi|38423524|       --------FS PL------AP LARVE----- ---------- ---------- ----------
3D1_gi|31321885|       --------YS PL------GS TG-------- ---------- ---------- ----------
3E1_gi|22207641|       --------YS PL------GS QNQVP----- ---------- ---------- ----------
3F1_gi|81625481|       --------YS PL-------- ---------- ---------- ---------- ----------
3F2_gi|13638516|       --------YM TL------A- ---------- ---------- ---------- ----------
3F3_gi|81635765|       --------YC SVA------- ---------- ---------- ---------- ----------
4A1_gi|112837|         --------FS PLRK----GA SRGPN----- ---------- ---------- ----------
```

```
4A2_gi|75220959|     --------FS PLRK----GA SRGPN----- ---------- ---------- ----------
4A3_gi|1215788|      --------FS PLRK----GA SRGPN----- ---------- ---------- ----------
4A4_gi|1514979|      --------FS PLRK----GA SRGPN----- ---------- ---------- ----------
4B1_gi|2792155|      --------WS PL------GA YKIF------ ---------- ---------- ---------W
4B2_gi|6478210|      --------HS VL------GA IGAP------ ---------- ---------- ---------W
4B3_gi|6478204|      --------HS VL------GA VGAA------ ---------- ---------- ---------W
4B4_gi|2792295|      --------YS PL------GA ARTK------ ---------- ---------- ---------W
4B5_gi|112807104|    --------YS PL------GA KGTH------ ---------- ---------- ---------W
4B6_gi|112807098|    --------YS PL------GA SGTH------ ---------- ---------- ---------W
4B7_gi|112807100|    --------YS PL------GA KGTH------ ---------- ---------- ---------W
4B8_gi|112807102|    --------YS PL------GA KGTHR----- ---------- ---------- ----------
4C1_gi|113595|       --------YS PL------GS S--------- ---------- ---------- ----------
4C2_gi|167113|       --------YS PL------CS S--------- ---------- ---------- ----------
4C3_gi|75221432|     --------YS PL------GS S-EKN----- ---------- ---------- ----------
4C4_gi|4539944|      --------YS PL------GS S--------- ---------- ---------- ----------
4C5_gi|13160397|     --------YS PL------GS PGTP------ ---------- ---------- ---------W
4C6_gi|13160399|     --------YS PL------GS PGTP------ ---------- ---------- ---------W
4C8_gi|111182163|    --------YS PL------GS QSKGE----- ---------- ---------- ----------
4C9_gi|111182165|    --------YS PL------GS PGTT------ ---------- ---------- ---------W
4C10_gi|111182167|   --------YS PL------GS PGTT------ ---------- ---------- ---------W
4C11_gi|111182169|   --------YS PL------GS PGTT------ ---------- ---------- ---------W
5A1_gi|408360251|    --------WS PL------G- ---------- ---------- ---------- ----------
5A2_gi|11127591|     --------WS PL------G- ---------- ---------- ---------- ----------
5B1_gi|2506173|      --------WS PIG-----GA INDGD----- ---------- ---------- ---------G
5C1_gi|144969|       --------WG PL------GQ GKYDL----- ---------- ---------- ---------F
5C2_gi|3916039|      --------WS PL-------A QGGKG----- ---------- ---------- ----------
5D1_gi|112735|       --------WS PL-------- ARRSE----- ---------- ---------- ----------
5E1_gi|5354195|      --------WS PL------GR ---------- ---------- ---------- ----------
5F1_gi|82504416|     --------WA PF------AE G--------- ---------- ---------- ----------
5G1_gi|16080393|     --------WS PL-------- ---------- ---------- ---------- ----------
5G2_gi|16079957|     --------WS PL-M------ ---------- ---------- ---------- ----------
6A1_gi|18202524|     --------WS PLAC----GI VSGKY----- ---------- ---------- -------DSG
6A2_gi|499328|       --------WS PLAC----GI VSGKY----- ---------- ---------- -------DSG
6A3_gi|2135947|      --------WS PLAC----GI ISGKY----- ---------- ---------- -------GNG
6A4_gi|975314|       --------WS PLAC----GI VSGKY----- ---------- ---------- -------DSG
6A5_gi|2827466|      --------WS PLAC----GI VSGKY----- ---------- ---------- ---DSGIPPY
6A6_gi|7914984|      --------WS PLAC----GI VSGKY----- ---------- ---------- ---DSGIPPY
6A7_gi|5019764|      --------WS PLAC----GI ISGKY----- ---------- ---------- -------GNG
6A8_gi|148747467|    --------WS PLACGIISGK YGNGV----- ---------- -------PES SRASLKCYQW
6A9_gi|24648619|     NTLFSTLNYC VI------GD HHTEV----- ---------- ---------- --KLNALNFW
6A10a_gi|726465|     --------WS PLAC----GI ISGKY----- ---------- ---------- -------GNG
6A10b_gi|5019766|    --------WS PLACGIISGK YGNGV----- ---------- -------PES SRASLKCYQW
6A11_gi|5922729|     --------WS PLACGLITGK YTDTV----- ---------- ---------- ----------
6A13_gi|52001073|    --------WS PLACGIISGK YGNGV----- ---------- -------PES SRASLKCYQW
6A14_gi|226823214|   --------WS PLACGLITSK YDGRV----- ---------- ---------- ----------
6B1_gi|902000|       --------WG PLSMALSDTQ NGDKL----- ---------- ------FLPK GSFKTKSFSW
6C1_gi|1063415|      --------WS PLASGVLTGK YNKGA----- ---------- --------IP SDSRFALENY
6C2_gi|2832783|      --------WS PLASGVLTGK YTKGN----- ---------- --------IP ADSRFALENY
7A1_gi|39932720|     --------FN PLAGGLLTGR YKYQD----- ---------- ---------K DGKNPESRFF
7A2_gi|41327764|     --------YN PLAGGLLTGK YKYED----- ---------- -----KDGKQ PVGRFFGNSW
7A3_gi|41152114|     --------FN PLAGGLLTGK YKYED----- ---------- -----KNGKQ PVGRFFGNTW
7A4_gi|6815049|      --------YN PLAGGLLTGK YRYED----- ---------- -----KDGKQ PEGRFFGNSW
8A1_gi|24638123|     --------YS PFCRGLLTGR IKTVE----- ---------- ------DLKE FAKSFPFLEY
8A2_gi|74627022|     --------YA PFCH----GL LTGRV----- ---------- ---------- ---------K
9A1_gi|146345520|    --------WG AL------GR GQYKS----- ---------- ---------- ---------A
9A2_gi|6093525|      --------WG VL------GR GQFRS----- ---------- ---------- ----------
9A3_gi|2492798|      --------WN VLCA----GK IRTDA----- ---------- ---------- ---------E
9B1_gi|6323998|      --------WD VM------GG GRFQS----- ---------- ---------- ----KKAMEE
9B2_gi|6319951|      --------WD VM------GG GRFQS----- ---------- ---------- ---------K
9B3_gi|6319958|      --------WD VM------GG GRFQS----- ---------- ---------- ---------K
9B4_gi|6322615|      --------WD VM------GG GRFQS----- ---------- ---------- ---------K
9C1_gi|2459734|      --------WS PLGQGFLTGK YTRED----- ---------- ---------- ---------G
10A1_gi|4731595|     --------WA SQ------GR GAHA------ ---------- ---------- ---------L
10A2_gi|3256056|     --------WA SQGR----GA HALAD----- ---------- ---------- ---------P
11A1_gi|1176985|     --------YF PLVS----GL LAGKY----- ---------- ---------- ---------T
11B1_gi|3123233|     --------YG SLCR----GL LTGKM----- ---------- ---------- ---------T
11B2_gi|3123121|     --------YS PLEQGLLTGT ITRDY----- ---------- ---------V PGGARANKVW
11B3_gi|85062654|    --------YS SLAQ----GL LTGKF----- ---------- ---------- ---------G
11C1_gi|81787577|    --------RG PVAR----GL LSRRP----- ---------- ---------- ---------L
12A1_gi|5305791|     --------WS PLHGGLLSGA LRKLA----- ---------- ---------- EGTAVKSGQG
12B1_gi|2599278|     --------WS PLHGGLLSGA LEKLA----- ---------- ---------- ----------
12C1_gi|5921163|     --------WS PLHGGLLGGV LRKTR----- ---------- ---------- ---ENTAVKS
13A1_gi|1351673|     --------YA PLGRGFLTGA YKSPD----- ---------- ---------- --------DF
13B1_gi|9106797|     --------WF PL-------- ---------- ---------- ---------- ----------
13C1_gi|81555851|    --------FS PLGKGFL-GA KFEKN----- ---------- ---------- -------ATF
14A1_gi|882530|      --------FT PLAQ----GL LTGKY----- ---------- ---------- -------LNG
15A1_gi|37196700|    --------AG VFNSGILAAP RGGEQ----- ---------- ---------- ------KFDY
all2316_aldo/keto    --------WS PF------GG RRRHQ----- ---------- ---------- ----------
```

```
                        1081
1A1_gi|5174391|         RDPD------ -------EPV LLE------- ----EP---- -------VVL ALAEKY-GRS
1A2_gi|1703236|         RDPN------ -------EPV LLE------- ----EP---- -------VVQ ALAEKY-NRS
1A3_gi|1703237|         RHPD------ -------EPV LLE------- ----EP---- -------VVL ALAEKH-GRS
1A4_gi|10946870|        RHPD------ -------EPV LLE------- ----EP---- -------VVL ALAEKH-GRS
1B1_gi|4502049|         AKPE------ -------DPS LLE------- ----DP---- -------RIK AIAAKH-NKT
1B2_gi|1703235|         AKPE------ -------DPS LLE------- ----DP---- -------RIK AIADKH-KKT
1B3_gi|1351911|         AKPE------ -------DPS LLE------- ----DP---- -------RIK AIAAKY-NKT
1B4_gi|6978491|         AKPE------ -------DPS LLE------- ----DP---- -------RIK EIAAKY-NKT
1B5_gi|113594|          AKPE------ -------DPS ILE------- ----DP---- -------RIK AIADKY-NKT
1B6_gi|584742|          AKPE------ -------DPS LLE------- ----DP---- -------RIK AIAAKY-NKT
1B7_gi|231525|          AKPE------ -------DPV VME------- ----IP---- -------KIK EIAAKH-KKT
1B8_gi|6679791|         AKPE------ -------DPS LLE------- ----DP---- -------KIK EIAAKH-EKT
1B9_gi|2114406|         AKPE------ -------DPS LLE------- ----DP---- -------KIK EIAAKH-KKT
1B10_gi|223468663|      AKPE------ -------DPS LLE------- ----DP---- -------KIK EIAAKH-KKT
1B12_gi|14330324|       AKPG------ -------EPM LLE------- ----DP---- -------KIK EIAARY-HKT
1B13_gi|15864567|       AKPD------ -------DPS LLQ------- ----DP---- -------KIK EIAAKH-KKT
1B14_gi|148540194|      AKPE------ -------DPV VLE------- ----IP---- -------KIK EIAAKH-KKT
1B15_gi|51094822|       AKPE------ -------DPS LLE------- ----DP---- -------KIK EIAAKH-KKT
1C1_gi|5453543|         VDPN------ -------SPV LLE------- ----DP---- -------VLC ALAKKH-KRT
1C2_gi|4503285|         VDPN------ -------SPV LLE------- ----DP---- -------VLC ALAKKH-KRT
1C3_gi|24497583|        VDPN------ -------SPV LLE------- ----DP---- -------VLC ALAKKH-KRT
1C4_gi|308153631|       VDPN------ -------SPV LLE------- ----DP---- -------VLC ALAKKH-KQT
1C5_gi|1352733|         VDQS------ -------APV LLE------- ----DP---- -------LIG ALAKKH-QQT
1C6_gi|13487925|        VDQS------ -------SPV LLD------- ----NP---- -------VLG SMAKKY-NRT
1C7_gi|129896|          VNSN------ -------NPV LLE------- ----DP---- -------VLC AIAKKH-KQT
1C8_gi|1709623|         INED------ -------TPV LLD------- ----DP---- -------ILC TMAKKY-KRT
1C9_gi|118634|          VDQK------ -------SPV LLD------- ----DP---- -------VLC AIAKKY-KQT
1C10a_gi|1345830|       VDLS------ -------LPV LLD------- ----DP---- -------ILN KVAAKY-NRT
1C10b_gi|1706132|       VDLS------ -------LPV LLD------- ----DP---- -------ILN KIAAKY-NRT
1C11_gi|1669605|        VNPN------ -------LPF LLE------- ----DP---- -------VLS AIAKKH-RQT
1C12_gi|85719330|       VDQN------ -------SPV LLN------- ----DP---- -------VLC DVAKRN-KRS
1C13_gi|171846276|      VDQN------ -------SPV LLN------- ----DP---- -------VLC DVAKKN-KRS
1C14_gi|19527294|       VDQK------ -------SPV LLD------- ----DP---- -------VLC AMANKY-KQT
1C15_gi|741804|         VSSD------ -------SPY LLE------- ----DP---- -------VLM TIAKKH-NQT
1C16_gi|741803|         VDQN------ -------SPV LLN------- ----DP---- -------VLC DVAKKN-KRT
1C17_gi|741805|         VDQN------ -------SPV LLD------- ----DP---- -------VLC DVAKKN-KRS
1C18_gi|1654715|        GGCC-AAGTC CATCGGGGTG TCT------- ----AACTTT AACCGTAGAC AACTGGAGAT
1C20_gi|16905111|       VDKS------ -------FPV LLD------- ----DP---- -------VLG SMAKKY-NRT
1C21_gi|126090770|      VDQN------ -------SPV LLD------- ----EP---- -------VLG SMAKKY-NRT
1C22_gi|38603389|       VDQN------ -------SPV LLN------- ----DP---- -------VLC DVAKKN-KRS
1C23_gi|62856987|       IDQS------ -------SPV LLE------- ----DP---- -------VLC AMAKKY-KRT
1C24_gi|84993586|       VDQN------ -------SPV LLD------- ----DP---- -------ILC DVAKKN-KRS
1C25_gi|15216337|       VDQN------ -------SPA LLE------- ----DP---- -------VLC ALAKKH-KRS
1D1_gi|5174695|         VNVS------ -------SPP LLK------- ----DA---- -------LLN SLGKRY-NKT
1D2_gi|398962|          VNVS------ -------SPP LLK------- ----DE---- -------LLT SLGKKY-NKT
1D3_gi|5689216|         VNTS------ -------LPP LLK------- ----DT---- -------LLN SLGKKY-KKT
1E1_gi|1698718|         ---------- ---------H LMD------- ----DT---- -------VIR KIAKKH-GKS
1E2_gi|269849539|       ---------- ---------D LID------- ----NP---- -------VIK RIAKEH-GKS
1G1_gi|17550248|        AP-------- -------APS DLQ------- ----DQ---- -------NVL ALAEKT-HKT
2A1_gi|134153|          ---------- ----MFGSVS PLD------- ----DP---- -------VLN DVAKKY-GKS
2A2_gi|1835701|         ---------- ----RFGSVS CLD------- ----DP---- -------VLK KLSDKH-NKS
2B1_gi|401428|          RALN------ -------TSP LFE------- ----NE---- -------TIK AIAAKH-GKS
2B2_gi|1351442|         KALN------ -------TPT LFE------- ----HD---- -------TIK SIASKH-KVT
2B3_gi|2492803|         GNET-AKK-- -------TQP LYE------- ----NK---- -------TIT TIAAKH-GKT
2B4_gi|1912051|         RALN------ -------TPT LFE------- ----HE---- -------TIK SIADKH-GKS
2B5_gi|3289019|         RALN------ -------TPT LFA------- ----HD---- -------TIK AIAAKY-NKT
2B6_gi|6321895|         DTPF-SDE-- --TRSTIYSE ILQSKTRFIT KNYIQP--FH ELQELLTKMG DFPKNK-EIE
2B7_gi|1912049|         RALN------ -------TPT LFE------- ----HE---- -------TIK LIADKH-GKS
2B8_gi|4103055|         DHPR-VKD-- -------VKP LFE------- ----HD---- -------VIK SVAGKV-KKT
2C1_gi|1332539|         TESG-KT--- -------YTS LLE------- ----HA---- -------SVK SVADKH-NVS
2D1_gi|7407095|         SVQN-AVD-- -------SPP LFE------- ----HQ---- -------LVK SIAEKH-GRT
2E1_gi|4753912|         MDLP------ --------GP KMD------- ----DP---- -------VLT SLAKKY-EKT
2E2_gi|18479021|        ---------- -----SHNNP VKN------- ----DR---- -------TVA DIARKY-NKT
2E3_gi|5052610|         ---------- ------KTPN YIY------- ----DA---- -------KVQ AIGDKY-KKS
3A1_gi|121087|          ---------- -------DAP LLK------- ----EP---- -------VIL EIAKKN-NVQ
3A2_gi|6320576|         ---------- -------NAP LLK------- ----EQ---- -------AII DMAKKH-GVE
3B1_gi|1142698|         ---------- --------PL LVQ------- ----HP---- -------EIK RIAEKN-GCT
3B2_gi|60458781|        ---------- --------PP LTE------- ----NP---- -------GIV DAAKRL-NHT
3B3_gi|60458785|        ---------- --------PP LTQ------- ----NP---- -------GIE ATAKRL-NHT
3C1_gi|536474|          ---------- -------GAP NLK------- ----IP---- -------LVK KLAEKY-NVT
3C2_gi|74626610|        ---------- -------QGP VAE------- ---------- -------FTK SLESKY-HVS
3C3_gi|38423524|        ---------- -------TNA LAE------- ---------- -------TLK RLAEKY-KKT
3D1_gi|31321885|        ---------- -------GPL LTA------- ----EP---- --------VV KIAEKH-SIS
3E1_gi|22207641|        ---------- -----TTGER VSE------- ----NK---- -------TLN EIAEKG-GNT
3F1_gi|81625481|        ---------- -------RRT LLS------- ----EK---- ----TKRTLE EIAKNH-GAT
3F2_gi|13638516|        ---------- -------YGK ALK------- ----DE---- -------VIA RIAAKH-NAT
3F3_gi|81635765|        ---------- -------RGE VFK------- ----HP---- -------VFA EIGARY-GKT
4A1_gi|112837|          ---------- ---------E VME------- ----ND---- -------VLK EIAEAH-GKS
```

```
4A2_gi|75220959|     ---------- ---------E VME------- ----ND---- -------MLK EIADAH-GKS
4A3_gi|1215788|      ---------- ---------E VME------- ----ND---- -------MLK GIAEAH-GKS
4A4_gi|1514979|      ---------- ---------E VME------- ----ND---- -------MLK GIAEAH-GKS
4B1_gi|2792155|      G--------- -------SGA VME------- ----NQ---- -------ILQ DIATAK-GKT
4B2_gi|6478210|      GSNA------ ---------- VMD------- ----SK---- -------VLH QIAVAR-GKS
4B3_gi|6478204|      GTNA------ ---------- VMH------- ----SK---- -------VLH QIAVAR-GKS
4B4_gi|2792295|      GDDR------ ---------- VLG------- ----SD---- -------IIE EIAQAK-GKS
4B5_gi|112807104|    G-SD------ ---------S VMD------- ----SG---- -------VLH EIAKSK-GKT
4B6_gi|112807098|    G-SD------ ---------S VMA------- ----SA---- -------VLR DIAQSK-GKT
4B7_gi|112807100|    G-SD------ ---------A VMD------- ----AG---- -------VLQ DIAASR-GKS
4B8_gi|112807102|    -GSD------ ---------A VMD------- ----AG---- -------VLQ EIAASR-GKS
4C1_gi|113595|       ---------- -------EKN LAH------- ----DP---- -------VVE KVANKL-NKT
4C2_gi|167113|       ---------- -------EKN LAH------- ----DP---- -------VVE KVANKL-NKT
4C3_gi|75221432|     ---------- ---------- LVH------- ----DP---- -------VVE KVANKL-NKT
4C4_gi|4539944|      ---------- -------ERD LLS------- ----DP---- -------TVL KIANKL-NKS
4C5_gi|13160397|     VKHD------ ---------- VLE------- ----NP---- -------ILV DVAEKL-GKT
4C6_gi|13160399|     VKHD------ ---------- VLE------- ----NP---- -------ILV DVAEKL-GKT
4C8_gi|111182163|    ---------- ------VRLK VLQ------- ----NP---- -------IVT EVAEKL-GKT
4C9_gi|111182165|    LKSD------ ---------- VLK------- ----NP---- -------ILN MVAEKL-GKS
4C10_gi|111182167|   LTSD------ ---------- VLK------- ----NP---- -------ILG GVAEKL-GKT
4C11_gi|111182169|   VKAD------ ---------- VLK------- ----SP---- -------VIE MIAKEI-GKS
5A1_gi|408360251|    ---------- -------QGK LLS------- ----NP---- -------ILS AIGAKY-NKT
5A2_gi|11127591|     ---------- -------QGH LVE------- ----DA---- -------RLK AIGGKY-GKT
5B1_gi|2506173|      DNHG-G---- -------RKH PLT------- ----DP---- -------VIT TIAEAH-GRS
5C1_gi|144969|       G--------- ---------- --A------- ----EP---- --------VT AAAAAH-GKT
5C2_gi|3916039|      ---------- ---------- VFD------- ----QK---- -------VIR DLADKY-GKT
5D1_gi|112735|       ---------- ---------- LLT------- ----EQ---- -------LLQ ELAVVY-GVT
5E1_gi|5354195|      ---------- -------ASK LLQ------- ----DD---- -------TIK KIADRL-GKS
5F1_gi|82504416|     ---------- -------KNG LFQ------- ----HP---- -------VLT AIGQKY-GKS
5G1_gi|16080393|     ---------- ------MQGQ LLD------- ----NE---- -------VLT QIAEKH-NKS
5G2_gi|16079957|     ---------- -------QGQ LLD------- ----HP---- -------VLA DIAQTY-NKS
6A1_gi|18202524|     IPPY-SRASL KGYQWLKDKI LSE------- ----EG---R RQQAKLKELQ AIAERL-GCT
6A2_gi|499328|       IPPY-SRASL KGYQWLKDKI LSE------- ----EG---R RQQAKLKELQ AIAERL-GCT
6A3_gi|2135947|      VPES-SRASL KCYQWLKERI VSE------- ----EG---R KQQNKLKDLS PIAERL-GCT
6A4_gi|975314|       IPPY-SRALL KGYQWLKDKI LSE------- ----EG---R RQQAKLKELQ AIAERL-GCT
6A5_gi|2827466|      SRAS-LKG-- --YQWLKDKI LSE------- ----EG---R RQQAKLKELQ AIAERL-GCT
6A6_gi|7914984|      SRAS-LKG-- --YQWLKDKI LSE------- ----EG---R RQQAKLKELQ AIAERL-GCT
6A7_gi|5019764|      VPES-SRASL KCYQWLKERI VSE------- ----EG---R KQQNKLKDLS PIAERL-GCT
6A8_gi|148747467|    LKER-IVS-- --EEGRKQQN KLK------- ---------- -------DLS PIAERL-GCT
6A9_gi|24648619|     RREF-YRQ-- --FS---NQG MID------- ----GSFPTV TFSKEQKKIV TLTEKEIQTS
6A10a_gi|726465|     VPES-SRASL KCYQWLKERI VSE------- ----EG---R KQQNKLKDLS PIAERL-GCT
6A10b_gi|5019766|    LKER-IVS-- --EEGRKQQN KLK------- ---------- -------DLS PIAERL-GCT
6A11_gi|5922729|     --PEKSRASF KGYHWLKEKA ISQ------- ----EG---K KQHSKVKELH PIADRL-NCT
6A13_gi|52001073|    LKER-IVS-- --EEGRKQQN KLK------- ---------- -------DLS PIAERL-GCT
6A14_gi|226823214|   --PDTCKATV KGYQWLKEKV QSE------- ----EG---K KQQARVMDLL PTARQL-GCT
6B1_gi|902000|       TEDEINRNAA LSPQGSWGKD RID------- ----EG---R RHCDRLRDLA ALAEKL-GCS
6C1_gi|1063415|      KNLA------ -------NRS LVD------- ----DV---- --LRKVSGLK PIAGEL-GVT
6C2_gi|2832783|      KNLA------ -------SRT LVD------- ----DI---- --LRKVNGLK PIAAEL-GVP
7A1_gi|39932720|     GNPF------ -------SQL YMD------- ----RYWKEE HFNGIALVEK ALKTTY-GPT
7A2_gi|41327764|     AETY------ -------RNR FWK------- ----EH---- HFEAIALVEK ALQAAY-GAS
7A3_gi|41152114|     AEMY------ -------RNR YWK------- ----EH---- HFEGIALVEK ALQAAY-GAS
7A4_gi|6815049|      SETY------ -------RNR FWK------- ----EH---- HFEAIALVEK ALKTTY-GTS
8A1_gi|24638123|     LDRF------ -------SPD VFA------- ----KN---- --LPFLQAVE QLAKKF-GMT
8A2_gi|74627022|     TAED------ -------LKD FIKAFPFLRN MDKFNPKVFE KNIPFLKAVE QLAQKF-GMS
9A1_gi|146345520|    EEFQ-QEGTR NMGPQEEKHR LMG------- ---------- ------AKLT EVGERK-GVA
9A2_gi|6093525|      -AEE-FSR-- --EGRKMGPQ DEK------- ----HR---- ---RLGEKLD QMAQQK-NTK
9A3_gi|2492798|      EERR-LKSGE GGRTLLQFDG WLR------- ----NE---- TERKVSKALE KVAEEIGAKS
9B1_gi|6323998|      RKKN-GEG-- -------LRT FVG------- ----GPEQTE LEVKISEALT KIAEEHGTES
9B2_gi|6319951|      KAMEERRKNG EGIRSFVGAS EQT------- ----DA---- -EIKISEALA KIAEEHGTES
9B3_gi|6319958|      KAMEERKKNG EGLRTVSGTS KQT------- ----DK---- -EVKISEALA KVAEEHGTES
9B4_gi|6322615|      KAVE-ERKKK GEG----LRT FFG------- ----TSEQTD MEVKISEALL KVAEEHGTES
9C1_gi|2459734|      LTGESRAA-- -------ESS RFE------- ----ESYLTE ENFDVHDELD AVAGEV-DAT
10A1_gi|4731595|     ADPE-ELRTG QLAKSWHSPA NLE------- ----LR---- -------RAA WLAEHK-GVS
10A2_gi|3256056|     AELR------ -------TSP LA-------- ----ECWYSE TNVERLRRAR TLAERR-GVS
11A1_gi|1176985|     EDTT-FPE-- --GDLRNEQE HFK------- ----GE-RFK ENIRKVNKLA PIAEKH-NVD
11B1_gi|3123233|     EEYT-FEG-- --DDLRNHDP KFQ------- ----KP-RFK EYLSAVNQLD KLAKTRYGKS
11B2_gi|3123121|     FQRENMLK-- -------VID MLE------- ---------- -------QWQ PLCARY-QCT
11B3_gi|85062654|    PDHQ-FAA-- --GDHRSHNR LYA------- ----DPENYQ RVQTALGLLK PIATTK-NCT
11C1_gi|81787577|    PEGE-GY--- -------LNY RYD------- ----EL---- -------KLL RESLPT-DRP
12A1_gi|5305791|     RAQR------ -------TLP ALR------- ----DT---- -----IARYE RFCARV-GRD
12B1_gi|2599278|     -AGT-AVK-- --SAQGRAQV LLPSL----- ----RP---- ----AIEAYE KFCRNL-GED
12C1_gi|5921163|     AQGR------ -------AVE ALE------- ----HH---- --RTTIAAYE DVCADH-GLD
13A1_gi|1351673|     PEGDFRRK-- -------APR YQK------- ----EN--FY KNLELVTKIE KIATAN-NIT
13B1_gi|9106797|     ---------- -------AAG DLA------- ----KP---- -----GGAVD ALAKAK-GAT
13C1_gi|81555851|    ASEDFRSV-- -------SPR FNQ------- ----EN--LA KNYALVELIQ DHAHAK-GVT
14A1_gi|882530|      IPQDSRMHRE GNKVRGLTPK MLT------- ----EA---- -NLNSLRLLN EMAQQR-GQS
15A1_gi|37196700|    ADAP------ -------AEI IAR------- ----TN---- -------RLH DICDEY-HVP
all2316_aldo/keto    ---------- ---------D LQD------- ----IP---- -------AIA QLAKAK-GVS
```

```
                        1141
1A1_gi|5174391|         PAQI---LLR ---------- ---------- ---------- ---------- ----------
1A2_gi|1703236|         PAQI---LLR ---------- ---------- ---------- ---------- ----------
1A3_gi|1703237|         PAQI---LLR ---------- ---------- ---------- ---------- ----------
1A4_gi|10946870|        PAQI---LLR ---------- ---------- ---------- ---------- ----------
1B1_gi|4502049|         TAQV---LIR ---------- ---------- ---------- ---------- ----------
1B2_gi|1703235|         TAQV---LIR ---------- ---------- ---------- ---------- ----------
1B3_gi|1351911|         TAQV---LIR ---------- ---------- ---------- ---------- ----------
1B4_gi|6978491|         TAQV---LIR ---------- ---------- ---------- ---------- ----------
1B5_gi|113594|          TAQV---LIR ---------- ---------- ---------- ---------- ----------
1B6_gi|584742|          TAQV---LIR ---------- ---------- ---------- ---------- ----------
1B7_gi|231525|          VAQV---LIR ---------- ---------- ---------- ---------- ----------
1B8_gi|6679791|         SAQV---LIR ---------- ---------- ---------- ---------- ----------
1B9_gi|2114406|         SAQV---LIR ---------- ---------- ---------- ---------- ----------
1B10_gi|223468663|      AAQV---LIR ---------- ---------- ---------- ---------- ----------
1B12_gi|14330324|       PAQV---LIR ---------- ---------- ---------- ---------- ----------
1B13_gi|15864567|       TAQV---LIR ---------- ---------- ---------- ---------- ----------
1B14_gi|148540194|      IAQV---LIR ---------- ---------- ---------- ---------- ----------
1B15_gi|51094822|       TAQV---LIR ---------- ---------- ---------- ---------- ----------
1C1_gi|5453543|         PALI---ALR ---------- ---------- ---------- ---------- ----------
1C2_gi|4503285|         PALI---ALR ---------- ---------- ---------- ---------- ----------
1C3_gi|24497583|        PALI---ALR ---------- ---------- ---------- ---------- ----------
1C4_gi|308153631|       PALI---ALR ---------- ---------- ---------- ---------- ----------
1C5_gi|1352733|         PALI---ALR ---------- ---------- ---------- ---------- ----------
1C6_gi|13487925|        PALI---ALR ---------- ---------- ---------- ---------- ----------
1C7_gi|129896|          PALV---ALR ---------- ---------- ---------- ---------- ----------
1C8_gi|1709623|         PALI---ALR ---------- ---------- ---------- ---------- ----------
1C9_gi|118634|          PALV---ALR ---------- ---------- ---------- ---------- ----------
1C10a_gi|1345830|       SAEI---AMR ---------- ---------- ---------- ---------- ----------
1C10b_gi|1706132|       SAEV---AMR ---------- ---------- ---------- ---------- ----------
1C11_gi|1669605|        PALV---ALR ---------- ---------- ---------- ---------- ----------
1C12_gi|85719330|       PALI---ALR ---------- ---------- ---------- ---------- ----------
1C13_gi|171846276|      PALI---ALR ---------- ---------- ---------- ---------- ----------
1C14_gi|19527294|       PALI---AIR ---------- ---------- ---------- ---------- ----------
1C15_gi|741804|         PGQV---ALR ---------- ---------- ---------- ---------- ----------
1C16_gi|741803|         PALI---ALR ---------- ---------- ---------- ---------- ----------
1C17_gi|741805|         PALI---ALR ---------- ---------- ---------- ---------- ----------
1C18_gi|1654715|        GATC---CTG ---------- ---------- ---------- ---------- ----------
1C20_gi|16905111|       PALI---ALR ---------- ---------- ---------- ---------- ----------
1C21_gi|126090770|      PALI---ALR ---------- ---------- ---------- ---------- ----------
1C22_gi|38603389|       PALI---ALR ---------- ---------- ---------- ---------- ----------
1C23_gi|62856987|       PALI---ALR ---------- ---------- ---------- ---------- ----------
1C24_gi|84993586|       PALI---ALR ---------- ---------- ---------- ---------- ----------
1C25_gi|15216337|       PALI---ALR ---------- ---------- ---------- ---------- ----------
1D1_gi|5174695|         AAQI---VLR ---------- ---------- ---------- ---------- ----------
1D2_gi|398962|          QAQI---VLR ---------- ---------- ---------- ---------- ----------
1D3_gi|5689216|         AAQI---VLR ---------- ---------- ---------- ---------- ----------
1E1_gi|1698718|         PAQI---LIR ---------- ---------- ---------- ---------- ----------
1E2_gi|269849539|       PAQI---LIR ---------- ---------- ---------- ---------- ----------
1G1_gi|17550248|        PAQV---LLR ---------- ---------- ---------- ---------- ----------
2A1_gi|134153|          VAQI---CLR ---------- ---------- ---------- ---------- ----------
2A2_gi|1835701|         PAQI---VLR ---------- ---------- ---------- ---------- ----------
2B1_gi|401428|          PAQV---LLR ---------- ---------- ---------- ---------- ----------
2B2_gi|1351442|         PQQV---LLR ---------- ---------- ---------- ---------- ----------
2B3_gi|2492803|         PFQV---LLR ---------- ---------- ---------- ---------- ----------
2B4_gi|1912051|         PAQV---LLR ---------- ---------- ---------- ---------- ----------
2B5_gi|3289019|         PAEV---LLR ---------- ---------- ---------- ---------- ----------
2B6_gi|6321895|         ISQLIETSLR RKVSGLHDIC PDLMLLLKIK SISSQGIVTG DELLFHHFLV SESFQNLGLN
2B7_gi|1912049|         PAQV---LLR ---------- ---------- ---------- ---------- ----------
2B8_gi|4103055|         PAQV---LLR ---------- ---------- ---------- ---------- ----------
2C1_gi|1332539|         TGQV---LLR ---------- ---------- ---------- ---------- ----------
2D1_gi|7407095|         PAQV---LLR ---------- ---------- ---------- ---------- ----------
2E1_gi|4753912|         PAQI---VLR ---------- ---------- ---------- ---------- ----------
2E2_gi|18479021|        VNQV---LVR ---------- ---------- ---------- ---------- ----------
2E3_gi|5052610|         TAQV---VLR ---------- ---------- ---------- ---------- ----------
3A1_gi|121087|          PGHV---VIS ---------- ---------- ---------- ---------- ----------
3A2_gi|6320576|         PAQL---IIS ---------- ---------- ---------- ---------- ----------
3B1_gi|1142698|         PAQV---LIA ---------- ---------- ---------- ---------- ----------
3B2_gi|60458781|        PAAV---LIA ---------- ---------- ---------- ---------- ----------
3B3_gi|60458785|        PAAV---LLA ---------- ---------- ---------- ---------- ----------
3C1_gi|536474|          GNDL---LIS ---------- ---------- ---------- ---------- ----------
3C2_gi|74626610|        DTQI---LLK ---------- ---------- ---------- ---------- ----------
3C3_gi|38423524|        EAQV---LLR ---------- ---------- ---------- ---------- ----------
3D1_gi|31321885|        PAAV---LLG ---------- ---------- ---------- ---------- ----------
3E1_gi|22207641|        LAQV---LIA ---------- ---------- ---------- ---------- ----------
3F1_gi|81625481|        IYQI---MLA ---------- ---------- ---------- ---------- ----------
3F2_gi|13638516|        PAQV---ILA ---------- ---------- ---------- ---------- ----------
3F3_gi|81635765|        AAQT---VLR ---------- ---------- ---------- ---------- ----------
4A1_gi|112837|          IAQV---SLR ---------- ---------- ---------- ---------- ----------
```

```
4A2_gi|75220959|      VAQI---SLR ---------- ---------- ---------- ---------- ----------
4A3_gi|1215788|       IAQV---SLR ---------- ---------- ---------- ---------- ----------
4A4_gi|1514979|       IAQV---SLR ---------- ---------- ---------- ---------- ----------
4B1_gi|2792155|       IAQV---ALR ---------- ---------- ---------- ---------- ----------
4B2_gi|6478210|       VAQV---SMR ---------- ---------- ---------- ---------- ----------
4B3_gi|6478204|       VAQV---SMR ---------- ---------- ---------- ---------- ----------
4B4_gi|2792295|       TAQI---SLR ---------- ---------- ---------- ---------- ----------
4B5_gi|112807104|     VAQV---CLR ---------- ---------- ---------- ---------- ----------
4B6_gi|112807098|     VAQV---CLR ---------- ---------- ---------- ---------- ----------
4B7_gi|112807100|     VAQV---CLR ---------- ---------- ---------- ---------- ----------
4B8_gi|112807102|     VAQV---CLR ---------- ---------- ---------- ---------- ----------
4C1_gi|113595|        PGQV---LIK ---------- ---------- ---------- ---------- ----------
4C2_gi|167113|        PGQV---LIK ---------- ---------- ---------- ---------- ----------
4C3_gi|75221432|      PGQV---LIK ---------- ---------- ---------- ---------- ----------
4C4_gi|4539944|       PGQL---LVR ---------- ---------- ---------- ---------- ----------
4C5_gi|13160397|      PAQV---AIR ---------- ---------- ---------- ---------- ----------
4C6_gi|13160399|      PAQV---ALR ---------- ---------- ---------- ---------- ----------
4C8_gi|111182163|     TAQV---ALR ---------- ---------- ---------- ---------- ----------
4C9_gi|111182165|     PAQV---ALR ---------- ---------- ---------- ---------- ----------
4C10_gi|111182167|    PAQV---ALR ---------- ---------- ---------- ---------- ----------
4C11_gi|111182169|    PAQT---ALR ---------- ---------- ---------- ---------- ----------
5A1_gi|408360251|     AAQV---ILR ---------- ---------- ---------- ---------- ----------
5A2_gi|11127591|      AAQV---MLR ---------- ---------- ---------- ---------- ----------
5B1_gi|2506173|       AAQV---ILR ---------- ---------- ---------- ---------- ----------
5C1_gi|144969|        PAQA---VLR ---------- ---------- ---------- ---------- ----------
5C2_gi|3916039|       PAQI---VIR ---------- ---------- ---------- ---------- ----------
5D1_gi|112735|        PTQV---VLR ---------- ---------- ---------- ---------- ----------
5E1_gi|5354195|       IPQV---ILR ---------- ---------- ---------- ---------- ----------
5F1_gi|82504416|      VGQV---VLR ---------- ---------- ---------- ---------- ----------
5G1_gi|16080393|      VAQV---ILR ---------- ---------- ---------- ---------- ----------
5G2_gi|16079957|      VAQI---ILR ---------- ---------- ---------- ---------- ----------
6A1_gi|18202524|      LPQL---AIA ---------- ---------- ---------- ---------- ----------
6A2_gi|499328|        LPQL---AIA ---------- ---------- ---------- ---------- ----------
6A3_gi|2135947|       LPQL---AVA ---------- ---------- ---------- ---------- ----------
6A4_gi|975314|        LPQL---AIA ---------- ---------- ---------- ---------- ----------
6A5_gi|2827466|       LPQL---AIA ---------- ---------- ---------- ---------- ----------
6A6_gi|7914984|       LPQL---AIA ---------- ---------- ---------- ---------- ----------
6A7_gi|5019764|       LPQL---AVA ---------- ---------- ---------- ---------- ----------
6A8_gi|148747467|     LPQL---AVA ---------- ---------- ---------- ---------- ----------
6A9_gi|24648619|      IAKVFGEVQQ ---------- ---------- ---------- ---------- ----------
6A10a_gi|726465|      LPQL---AVA ---------- ---------- ---------- ---------- ----------
6A10b_gi|5019766|     LPQL---AVA ---------- ---------- ---------- ---------- ----------
6A11_gi|5922729|      VTQL---AIA ---------- ---------- ---------- ---------- ----------
6A13_gi|52001073|     LPQL---AVA ---------- ---------- ---------- ---------- ----------
6A14_gi|226823214|    VAQL---AIA ---------- ---------- ---------- ---------- ----------
6B1_gi|902000|        PTQL---SIA ---------- ---------- ---------- ---------- ----------
6C1_gi|1063415|       LAQL---AIA ---------- ---------- ---------- ---------- ----------
6C2_gi|2832783|       LAQL---AIA ---------- ---------- ---------- ---------- ----------
7A1_gi|39932720|      APSMISAAVR ---------- ---------- ---------- ---------- ----------
7A2_gi|41327764|      APSVTSAALR ---------- ---------- ---------- ---------- ----------
7A3_gi|41152114|      APSMTSATLR ---------- ---------- ---------- ---------- ----------
7A4_gi|6815049|       APSMTSAALR ---------- ---------- ---------- ---------- ----------
8A1_gi|24638123|      MPEF---SLL ---------- ---------- ---------- ---------- ----------
8A2_gi|74627022|      MPEF---ALN ---------- ---------- ---------- ---------- ----------
9A1_gi|146345520|     AAAI---ALA ---------- ---------- ---------- ---------- ----------
9A2_gi|6093525|       ATSI---AQA ---------- ---------- ---------- ---------- ----------
9A3_gi|2492798|       ITSV---AIA ---------- ---------- ---------- ---------- ----------
9B1_gi|6323998|       VTAI---AIA ---------- ---------- ---------- ---------- ----------
9B2_gi|6319951|       VTAI---AIA ---------- ---------- ---------- ---------- ----------
9B3_gi|6319958|       VTAI---AIA ---------- ---------- ---------- ---------- ----------
9B4_gi|6322615|       VTAI---AIA ---------- ---------- ---------- ---------- ----------
9C1_gi|2459734|       PAQT---ALA ---------- ---------- ---------- ---------- ----------
10A1_gi|4731595|      STGL---ALA ---------- ---------- ---------- ---------- ----------
10A2_gi|3256056|      STGI---ALA ---------- ---------- ---------- ---------- ----------
11A1_gi|1176985|      IPHI---VLA ---------- ---------- ---------- ---------- ----------
11B1_gi|3123233|      VIHL---AVR ---------- ---------- ---------- ---------- ----------
11B2_gi|3123121|      IPTL---ALA ---------- ---------- ---------- ---------- ----------
11B3_gi|85062654|     LAQL---AIA ---------- ---------- ---------- ---------- ----------
11C1_gi|81787577|     LHEL---ALQ ---------- ---------- ---------- ---------- ----------
12A1_gi|5305791|      PAEV---GLA ---------- ---------- ---------- ---------- ----------
12B1_gi|2599278|      PAEV---GLA ---------- ---------- ---------- ---------- ----------
12C1_gi|5921163|      PAHV---GMA ---------- ---------- ---------- ---------- ----------
13A1_gi|1351673|      PGQL---SLA ---------- ---------- ---------- ---------- ----------
13B1_gi|9106797|      AGQI---ALA ---------- ---------- ---------- ---------- ----------
13C1_gi|81555851|     PAQL---ALS ---------- ---------- ---------- ---------- ----------
14A1_gi|882530|       MAQM---ALS ---------- ---------- ---------- ---------- ----------
15A1_gi|37196700|     LAAA---AMQ ---------- ---------- ---------- ---------- ----------
all2316_aldo/keto     VYSI---VLA ---------- ---------- ---------- ---------- ----------
```

```
                     1201
1A1_gi|5174391|      ---------- ---------- ---------- ---------- ---------- ----------
1A2_gi|1703236|      ---------- ---------- ---------- ---------- ---------- ----------
1A3_gi|1703237|      ---------- ---------- ---------- ---------- ---------- ----------
1A4_gi|10946870|     ---------- ---------- ---------- ---------- ---------- ----------
1B1_gi|4502049|      ---------- ---------- ---------- ---------- ---------- ----------
1B2_gi|1703235|      ---------- ---------- ---------- ---------- ---------- ----------
1B3_gi|1351911|      ---------- ---------- ---------- ---------- ---------- ----------
1B4_gi|6978491|      ---------- ---------- ---------- ---------- ---------- ----------
1B5_gi|113594|       ---------- ---------- ---------- ---------- ---------- ----------
1B6_gi|584742|       ---------- ---------- ---------- ---------- ---------- ----------
1B7_gi|231525|       ---------- ---------- ---------- ---------- ---------- ----------
1B8_gi|6679791|      ---------- ---------- ---------- ---------- ---------- ----------
1B9_gi|2114406|      ---------- ---------- ---------- ---------- ---------- ----------
1B10_gi|223468663|   ---------- ---------- ---------- ---------- ---------- ----------
1B12_gi|14330324|    ---------- ---------- ---------- ---------- ---------- ----------
1B13_gi|15864567|    ---------- ---------- ---------- ---------- ---------- ----------
1B14_gi|148540194|   ---------- ---------- ---------- ---------- ---------- ----------
1B15_gi|51094822|    ---------- ---------- ---------- ---------- ---------- ----------
1C1_gi|5453543|      ---------- ---------- ---------- ---------- ---------- ----------
1C2_gi|4503285|      ---------- ---------- ---------- ---------- ---------- ----------
1C3_gi|24497583|     ---------- ---------- ---------- ---------- ---------- ----------
1C4_gi|308153631|    ---------- ---------- ---------- ---------- ---------- ----------
1C5_gi|1352733|      ---------- ---------- ---------- ---------- ---------- ----------
1C6_gi|13487925|     ---------- ---------- ---------- ---------- ---------- ----------
1C7_gi|129896|       ---------- ---------- ---------- ---------- ---------- ----------
1C8_gi|1709623|      ---------- ---------- ---------- ---------- ---------- ----------
1C9_gi|118634|       ---------- ---------- ---------- ---------- ---------- ----------
1C10a_gi|1345830|    ---------- ---------- ---------- ---------- ---------- ----------
1C10b_gi|1706132|    ---------- ---------- ---------- ---------- ---------- ----------
1C11_gi|1669605|     ---------- ---------- ---------- ---------- ---------- ----------
1C12_gi|85719330|    ---------- ---------- ---------- ---------- ---------- ----------
1C13_gi|171846276|   ---------- ---------- ---------- ---------- ---------- ----------
1C14_gi|19527294|    ---------- ---------- ---------- ---------- ---------- ----------
1C15_gi|741804|      ---------- ---------- ---------- ---------- ---------- ----------
1C16_gi|741803|      ---------- ---------- ---------- ---------- ---------- ----------
1C17_gi|741805|      ---------- ---------- ---------- ---------- ---------- ----------
1C18_gi|1654715|     ---------- ---------- ---------- ---------- ---------- ----------
1C20_gi|16905111|    ---------- ---------- ---------- ---------- ---------- ----------
1C21_gi|126090770|   ---------- ---------- ---------- ---------- ---------- ----------
1C22_gi|38603389|    ---------- ---------- ---------- ---------- ---------- ----------
1C23_gi|62856987|    ---------- ---------- ---------- ---------- ---------- ----------
1C24_gi|84993586|    ---------- ---------- ---------- ---------- ---------- ----------
1C25_gi|15216337|    ---------- ---------- ---------- ---------- ---------- ----------
1D1_gi|5174695|      ---------- ---------- ---------- ---------- ---------- ----------
1D2_gi|398962|       ---------- ---------- ---------- ---------- ---------- ----------
1D3_gi|5689216|      ---------- ---------- ---------- ---------- ---------- ----------
1E1_gi|1698718|      ---------- ---------- ---------- ---------- ---------- ----------
1E2_gi|269849539|    ---------- ---------- ---------- ---------- ---------- ----------
1G1_gi|17550248|     ---------- ---------- ---------- ---------- ---------- ----------
2A1_gi|134153|       ---------- ---------- ---------- ---------- ---------- ----------
2A2_gi|1835701|      ---------- ---------- ---------- ---------- ---------- ----------
2B1_gi|401428|       ---------- ---------- ---------- ---------- ---------- ----------
2B2_gi|1351442|      ---------- ---------- ---------- ---------- ---------- ----------
2B3_gi|2492803|      ---------- ---------- ---------- ---------- ---------- ----------
2B4_gi|1912051|      ---------- ---------- ---------- ---------- ---------- ----------
2B5_gi|3289019|      ---------- ---------- ---------- ---------- ---------- ----------
2B6_gi|6321895|      EIWNIVNLVQ MTCFNDLCKE KFDAKVLERK GVVAGYLSQN EEFKDEFNTE CINSTTWWNI
2B7_gi|1912049|      ---------- ---------- ---------- ---------- ---------- ----------
2B8_gi|4103055|      ---------- ---------- ---------- ---------- ---------- ----------
2C1_gi|1332539|      ---------- ---------- ---------- ---------- ---------- ----------
2D1_gi|7407095|      ---------- ---------- ---------- ---------- ---------- ----------
2E1_gi|4753912|      ---------- ---------- ---------- ---------- ---------- ----------
2E2_gi|18479021|     ---------- ---------- ---------- ---------- ---------- ----------
2E3_gi|5052610|      ---------- ---------- ---------- ---------- ---------- ----------
3A1_gi|121087|       ---------- ---------- ---------- ---------- ---------- ----------
3A2_gi|6320576|      ---------- ---------- ---------- ---------- ---------- ----------
3B1_gi|1142698|      ---------- ---------- ---------- ---------- ---------- ----------
3B2_gi|60458781|     ---------- ---------- ---------- ---------- ---------- ----------
3B3_gi|60458785|     ---------- ---------- ---------- ---------- ---------- ----------
3C1_gi|536474|       ---------- ---------- ---------- ---------- ---------- ----------
3C2_gi|74626610|     ---------- ---------- ---------- ---------- ---------- ----------
3C3_gi|38423524|     ---------- ---------- ---------- ---------- ---------- ----------
3D1_gi|31321885|     ---------- ---------- ---------- ---------- ---------- ----------
3E1_gi|22207641|     ---------- ---------- ---------- ---------- ---------- ----------
3F1_gi|81625481|     ---------- ---------- ---------- ---------- ---------- ----------
3F2_gi|13638516|     ---------- ---------- ---------- ---------- ---------- ----------
3F3_gi|81635765|     ---------- ---------- ---------- ---------- ---------- ----------
4A1_gi|112837|       ---------- ---------- ---------- ---------- ---------- ----------
```

```
4A2_gi|75220959|     ---------- ---------- ---------- ---------- ---------- ----------
4A3_gi|1215788|      ---------- ---------- ---------- ---------- ---------- ----------
4A4_gi|1514979|      ---------- ---------- ---------- ---------- ---------- ----------
4B1_gi|2792155|      ---------- ---------- ---------- ---------- ---------- ----------
4B2_gi|6478210|      ---------- ---------- ---------- ---------- ---------- ----------
4B3_gi|6478204|      ---------- ---------- ---------- ---------- ---------- ----------
4B4_gi|2792295|      ---------- ---------- ---------- ---------- ---------- ----------
4B5_gi|112807104|    ---------- ---------- ---------- ---------- ---------- ----------
4B6_gi|112807098|    ---------- ---------- ---------- ---------- ---------- ----------
4B7_gi|112807100|    ---------- ---------- ---------- ---------- ---------- ----------
4B8_gi|112807102|    ---------- ---------- ---------- ---------- ---------- ----------
4C1_gi|113595|       ---------- ---------- ---------- ---------- ---------- ----------
4C2_gi|167113|       ---------- ---------- ---------- ---------- ---------- ----------
4C3_gi|75221432|     ---------- ---------- ---------- ---------- ---------- ----------
4C4_gi|4539944|      ---------- ---------- ---------- ---------- ---------- ----------
4C5_gi|13160397|     ---------- ---------- ---------- ---------- ---------- ----------
4C6_gi|13160399|     ---------- ---------- ---------- ---------- ---------- ----------
4C8_gi|111182163|    ---------- ---------- ---------- ---------- ---------- ----------
4C9_gi|111182165|    ---------- ---------- ---------- ---------- ---------- ----------
4C10_gi|111182167|   ---------- ---------- ---------- ---------- ---------- ----------
4C11_gi|111182169|   ---------- ---------- ---------- ---------- ---------- ----------
5A1_gi|408360251|    ---------- ---------- ---------- ---------- ---------- ----------
5A2_gi|11127591|     ---------- ---------- ---------- ---------- ---------- ----------
5B1_gi|2506173|      ---------- ---------- ---------- ---------- ---------- ----------
5C1_gi|144969|       ---------- ---------- ---------- ---------- ---------- ----------
5C2_gi|3916039|      ---------- ---------- ---------- ---------- ---------- ----------
5D1_gi|112735|       ---------- ---------- ---------- ---------- ---------- ----------
5E1_gi|5354195|      ---------- ---------- ---------- ---------- ---------- ----------
5F1_gi|82504416|     ---------- ---------- ---------- ---------- ---------- ----------
5G1_gi|16080393|     ---------- ---------- ---------- ---------- ---------- ----------
5G2_gi|16079957|     ---------- ---------- ---------- ---------- ---------- ----------
6A1_gi|18202524|     ---------- ---------- ---------- ---------- ---------- ----------
6A2_gi|499328|       ---------- ---------- ---------- ---------- ---------- ----------
6A3_gi|2135947|      ---------- ---------- ---------- ---------- ---------- ----------
6A4_gi|975314|       ---------- ---------- ---------- ---------- ---------- ----------
6A5_gi|2827466|      ---------- ---------- ---------- ---------- ---------- ----------
6A6_gi|7914984|      ---------- ---------- ---------- ---------- ---------- ----------
6A7_gi|5019764|      ---------- ---------- ---------- ---------- ---------- ----------
6A8_gi|148747467|    ---------- ---------- ---------- ---------- ---------- ----------
6A9_gi|24648619|     ---------- ---------- ---------- ---------- ---------- ----------
6A10a_gi|726465|     ---------- ---------- ---------- ---------- ---------- ----------
6A10b_gi|5019766|    ---------- ---------- ---------- ---------- ---------- ----------
6A11_gi|5922729|     ---------- ---------- ---------- ---------- ---------- ----------
6A13_gi|52001073|    ---------- ---------- ---------- ---------- ---------- ----------
6A14_gi|226823214|   ---------- ---------- ---------- ---------- ---------- ----------
6B1_gi|902000|       ---------- ---------- ---------- ---------- ---------- ----------
6C1_gi|1063415|      ---------- ---------- ---------- ---------- ---------- ----------
6C2_gi|2832783|      ---------- ---------- ---------- ---------- ---------- ----------
7A1_gi|39932720|     ---------- ---------- ---------- ---------- ---------- ----------
7A2_gi|41327764|     ---------- ---------- ---------- ---------- ---------- ----------
7A3_gi|41152114|     ---------- ---------- ---------- ---------- ---------- ----------
7A4_gi|6815049|      ---------- ---------- ---------- ---------- ---------- ----------
8A1_gi|24638123|     ---------- ---------- ---------- ---------- ---------- ----------
8A2_gi|74627022|     ---------- ---------- ---------- ---------- ---------- ----------
9A1_gi|146345520|    ---------- ---------- ---------- ---------- ---------- ----------
9A2_gi|6093525|      ---------- ---------- ---------- ---------- ---------- ----------
9A3_gi|2492798|      ---------- ---------- ---------- ---------- ---------- ----------
9B1_gi|6323998|      ---------- ---------- ---------- ---------- ---------- ----------
9B2_gi|6319951|      ---------- ---------- ---------- ---------- ---------- ----------
9B3_gi|6319958|      ---------- ---------- ---------- ---------- ---------- ----------
9B4_gi|6322615|      ---------- ---------- ---------- ---------- ---------- ----------
9C1_gi|2459734|      ---------- ---------- ---------- ---------- ---------- ----------
10A1_gi|4731595|     ---------- ---------- ---------- ---------- ---------- ----------
10A2_gi|3256056|     ---------- ---------- ---------- ---------- ---------- ----------
11A1_gi|1176985|     ---------- ---------- ---------- ---------- ---------- ----------
11B1_gi|3123233|     ---------- ---------- ---------- ---------- ---------- ----------
11B2_gi|3123121|     ---------- ---------- ---------- ---------- ---------- ----------
11B3_gi|85062654|    ---------- ---------- ---------- ---------- ---------- ----------
11C1_gi|81787577|    ---------- ---------- ---------- ---------- ---------- ----------
12A1_gi|5305791|     ---------- ---------- ---------- ---------- ---------- ----------
12B1_gi|2599278|     ---------- ---------- ---------- ---------- ---------- ----------
12C1_gi|5921163|     ---------- ---------- ---------- ---------- ---------- ----------
13A1_gi|1351673|     ---------- ---------- ---------- ---------- ---------- ----------
13B1_gi|9106797|     ---------- ---------- ---------- ---------- ---------- ----------
13C1_gi|81555851|    ---------- ---------- ---------- ---------- ---------- ----------
14A1_gi|882530|      ---------- ---------- ---------- ---------- ---------- ----------
15A1_gi|37196700|    ---------- ---------- ---------- ---------- ---------- ----------
all2316_aldo/keto    ---------- ---------- ---------- ---------- ---------- ----------
```

```
                    1261
1A1_gi|5174391|      ---------- WQV------- ---------- ---------- ---------- ----------
1A2_gi|1703236|      ---------- WQV------- ---------- ---------- ---------- ----------
1A3_gi|1703237|      ---------- WQV------- ---------- ---------- ---------- ----------
1A4_gi|10946870|     ---------- WQV------- ---------- ---------- ---------- ----------
1B1_gi|4502049|      ---------- FPM------- ---------- ---------- ---------- ----------
1B2_gi|1703235|      ---------- FPM------- ---------- ---------- ---------- ----------
1B3_gi|1351911|      ---------- FPI------- ---------- ---------- ---------- ----------
1B4_gi|6978491|      ---------- FPI------- ---------- ---------- ---------- ----------
1B5_gi|113594|       ---------- FPI------- ---------- ---------- ---------- ----------
1B6_gi|584742|       ---------- FPM------- ---------- ---------- ---------- ----------
1B7_gi|231525|       ---------- FHV------- ---------- ---------- ---------- ----------
1B8_gi|6679791|      ---------- FHI------- ---------- ---------- ---------- ----------
1B9_gi|2114406|      ---------- FHI------- ---------- ---------- ---------- ----------
1B10_gi|223468663|   ---------- FHI------- ---------- ---------- ---------- ----------
1B12_gi|14330324|    ---------- FII------- ---------- ---------- ---------- ----------
1B13_gi|15864567|    ---------- FHI------- ---------- ---------- ---------- ----------
1B14_gi|148540194|   ---------- FHV------- ---------- ---------- ---------- ----------
1B15_gi|51094822|    ---------- FHI------- ---------- ---------- ---------- ----------
1C1_gi|5453543|      ---------- YQL------- ---------- ---------- ---------- ----------
1C2_gi|4503285|      ---------- YQL------- ---------- ---------- ---------- ----------
1C3_gi|24497583|     ---------- YQL------- ---------- ---------- ---------- ----------
1C4_gi|308153631|    ---------- YQL------- ---------- ---------- ---------- ----------
1C5_gi|1352733|      ---------- YQL------- ---------- ---------- ---------- ----------
1C6_gi|13487925|     ---------- YQL------- ---------- ---------- ---------- ----------
1C7_gi|129896|       ---------- YQV------- ---------- ---------- ---------- ----------
1C8_gi|1709623|      ---------- YQL------- ---------- ---------- ---------- ----------
1C9_gi|118634|       ---------- YQL------- ---------- ---------- ---------- ----------
1C10a_gi|1345830|    ---------- FIL------- ---------- ---------- ---------- ----------
1C10b_gi|1706132|    ---------- FIL------- ---------- ---------- ---------- ----------
1C11_gi|1669605|     ---------- YQI------- ---------- ---------- ---------- ----------
1C12_gi|85719330|    ---------- YLF------- ---------- ---------- ---------- ----------
1C13_gi|171846276|   ---------- YLI------- ---------- ---------- ---------- ----------
1C14_gi|19527294|    ---------- YQL------- ---------- ---------- ---------- ----------
1C15_gi|741804|      ---------- YQL------- ---------- ---------- ---------- ----------
1C16_gi|741803|      ---------- YLV------- ---------- ---------- ---------- ----------
1C17_gi|741805|      ---------- YLV------- ---------- ---------- ---------- ----------
1C18_gi|1654715|     ---------- AAC------- ---------- ---------- ---------- ----------
1C20_gi|16905111|    ---------- YQV------- ---------- ---------- ---------- ----------
1C21_gi|126090770|   ---------- YQL------- ---------- ---------- ---------- ----------
1C22_gi|38603389|    ---------- YLI------- ---------- ---------- ---------- ----------
1C23_gi|62856987|    ---------- YQL------- ---------- ---------- ---------- ----------
1C24_gi|84993586|    ---------- YLF------- ---------- ---------- ---------- ----------
1C25_gi|15216337|    ---------- YQL------- ---------- ---------- ---------- ----------
1D1_gi|5174695|      ---------- FNI------- ---------- ---------- ---------- ----------
1D2_gi|398962|       ---------- FDI------- ---------- ---------- ---------- ----------
1D3_gi|5689216|      ---------- FNV------- ---------- ---------- ---------- ----------
1E1_gi|1698718|      ---------- FQI------- ---------- ---------- ---------- ----------
1E2_gi|269849539|    ---------- FQI------- ---------- ---------- ---------- ----------
1G1_gi|17550248|     ---------- YAL------- ---------- ---------- ---------- ----------
2A1_gi|134153|       ---------- WGI------- ---------- ---------- ---------- ----------
2A2_gi|1835701|      ---------- WGV------- ---------- ---------- ---------- ----------
2B1_gi|401428|       ---------- WSS------- ---------- ---------- ---------- ----------
2B2_gi|1351442|      ---------- WAT------- ---------- ---------- ---------- ----------
2B3_gi|2492803|      ---------- WVN------- ---------- ---------- ---------- ----------
2B4_gi|1912051|      ---------- WAT------- ---------- ---------- ---------- ----------
2B5_gi|3289019|      ---------- WAA------- ---------- ---------- ---------- ----------
2B6_gi|6321895|      LERIDHKLFM WIM------- ---------- ---------- ---------- ----------
2B7_gi|1912049|      ---------- WAT------- ---------- ---------- ---------- ----------
2B8_gi|4103055|      ---------- WAT------- ---------- ---------- ---------- ----------
2C1_gi|1332539|      ---------- WAL------- ---------- ---------- ---------- ----------
2D1_gi|7407095|      ---------- WAT------- ---------- ---------- ---------- ----------
2E1_gi|4753912|      ---------- WLV------- ---------- ---------- ---------- ----------
2E2_gi|18479021|     ---------- YLL------- ---------- ---------- ---------- ----------
2E3_gi|5052610|      ---------- YLI------- ---------- ---------- ---------- ----------
3A1_gi|121087|       ---------- WHV------- ---------- ---------- ---------- ----------
3A2_gi|6320576|      ---------- WSI------- ---------- ---------- ---------- ----------
3B1_gi|1142698|      ---------- WAI------- ---------- ---------- ---------- ----------
3B2_gi|60458781|     ---------- WGI------- ---------- ---------- ---------- ----------
3B3_gi|60458785|     ---------- WGI------- ---------- ---------- ---------- ----------
3C1_gi|536474|       ---------- YHI------- ---------- ---------- ---------- ----------
3C2_gi|74626610|     ---------- WAY------- ---------- ---------- ---------- ----------
3C3_gi|38423524|     ---------- YTL------- ---------- ---------- ---------- ----------
3D1_gi|31321885|     ---------- YQI------- ---------- ---------- ---------- ----------
3E1_gi|22207641|     ---------- WGL------- ---------- ---------- ---------- ----------
3F1_gi|81625481|     ---------- WLL------- ---------- ---------- ---------- ----------
3F2_gi|13638516|     ---------- WAM------- ---------- ---------- ---------- ----------
3F3_gi|81635765|     ---------- WIL------- ---------- ---------- ---------- ----------
4A1_gi|112837|       ---------- WLY------- ---------- ---------- ---------- ----------
```

```
4A2_gi|75220959|      ---------- WLY------- ---------- ---------- ---------- ----------
4A3_gi|1215788|       ---------- WLY------- ---------- ---------- ---------- ----------
4A4_gi|1514979|       ---------- WLY------- ---------- ---------- ---------- ----------
4B1_gi|2792155|       ---------- WVY------- ---------- ---------- ---------- ----------
4B2_gi|6478210|       ---------- WVY------- ---------- ---------- ---------- ----------
4B3_gi|6478204|       ---------- WVY------- ---------- ---------- ---------- ----------
4B4_gi|2792295|       ---------- WVY------- ---------- ---------- ---------- ----------
4B5_gi|112807104|     ---------- WVY------- ---------- ---------- ---------- ----------
4B6_gi|112807098|     ---------- WVY------- ---------- ---------- ---------- ----------
4B7_gi|112807100|     ---------- WVY------- ---------- ---------- ---------- ----------
4B8_gi|112807102|     ---------- WVY------- ---------- ---------- ---------- ----------
4C1_gi|113595|        ---------- WAL------- ---------- ---------- ---------- ----------
4C2_gi|167113|        ---------- WAL------- ---------- ---------- ---------- ----------
4C3_gi|75221432|      ---------- WAL------- ---------- ---------- ---------- ----------
4C4_gi|4539944|       ---------- WAV------- ---------- ---------- ---------- ----------
4C5_gi|13160397|      ---------- WGL------- ---------- ---------- ---------- ----------
4C6_gi|13160399|      ---------- WGL------- ---------- ---------- ---------- ----------
4C8_gi|111182163|     ---------- WGL------- ---------- ---------- ---------- ----------
4C9_gi|111182165|     ---------- WGL------- ---------- ---------- ---------- ----------
4C10_gi|111182167|    ---------- WGL------- ---------- ---------- ---------- ----------
4C11_gi|111182169|    ---------- WGL------- ---------- ---------- ---------- ----------
5A1_gi|408360251|     ---------- WNI------- ---------- ---------- ---------- ----------
5A2_gi|11127591|      ---------- WEI------- ---------- ---------- ---------- ----------
5B1_gi|2506173|       ---------- WHF------- ---------- ---------- ---------- ----------
5C1_gi|144969|        ---------- WHL------- ---------- ---------- ---------- ----------
5C2_gi|3916039|       ---------- WHL------- ---------- ---------- ---------- ----------
5D1_gi|112735|        ---------- WHV------- ---------- ---------- ---------- ----------
5E1_gi|5354195|       ---------- WHV------- ---------- ---------- ---------- ----------
5F1_gi|82504416|      ---------- WIF------- ---------- ---------- ---------- ----------
5G1_gi|16080393|      ---------- WDL------- ---------- ---------- ---------- ----------
5G2_gi|16079957|      ---------- WDL------- ---------- ---------- ---------- ----------
6A1_gi|18202524|      ---------- WCL------- ---------- ---------- ---------- ----------
6A2_gi|499328|        ---------- WCL------- ---------- ---------- ---------- ----------
6A3_gi|2135947|       ---------- WCL------- ---------- ---------- ---------- ----------
6A4_gi|975314|        ---------- WCL------- ---------- ---------- ---------- ----------
6A5_gi|2827466|       ---------- WCL------- ---------- ---------- ---------- ----------
6A6_gi|7914984|       ---------- WCL------- ---------- ---------- ---------- ----------
6A7_gi|5019764|       ---------- WCL------- ---------- ---------- ---------- ----------
6A8_gi|148747467|     ---------- WCL------- ---------- ---------- ---------- ----------
6A9_gi|24648619|      ---------- YGYFGILLKC LREETSMEVL EFLIKGVKTM TEKFERYKGI MEEIEMRSPL
6A10a_gi|726465|      ---------- WCL------- ---------- ---------- ---------- ----------
6A10b_gi|5019766|     ---------- WCL------- ---------- ---------- ---------- ----------
6A11_gi|5922729|      ---------- WCL------- ---------- ---------- ---------- ----------
6A13_gi|52001073|     ---------- WCL------- ---------- ---------- ---------- ----------
6A14_gi|226823214|    ---------- WCL------- ---------- ---------- ---------- ----------
6B1_gi|902000|        ---------- WSL------- ---------- ---------- ---------- ----------
6C1_gi|1063415|       ---------- WCA------- ---------- ---------- ---------- ----------
6C2_gi|2832783|       ---------- WCA------- ---------- ---------- ---------- ----------
7A1_gi|39932720|      ---------- WMY------- ---------- ---------- ---------- ----------
7A2_gi|41327764|      ---------- WMY------- ---------- ---------- ---------- ----------
7A3_gi|41152114|      ---------- WMY------- ---------- ---------- ---------- ----------
7A4_gi|6815049|       ---------- WMY------- ---------- ---------- ---------- ----------
8A1_gi|24638123|      ---------- FIM------- ---------- ---------- ---------- ----------
8A2_gi|74627022|      ---------- FII------- ---------- ---------- ---------- ----------
9A1_gi|146345520|     ---------- YLL------- ---------- ---------- ---------- ----------
9A2_gi|6093525|       ---------- YVM------- ---------- ---------- ---------- ----------
9A3_gi|2492798|       ---------- YLM------- ---------- ---------- ---------- ----------
9B1_gi|6323998|       ---------- YVR------- ---------- ---------- ---------- ----------
9B2_gi|6319951|       ---------- YVR------- ---------- ---------- ---------- ----------
9B3_gi|6319958|       ---------- YVR------- ---------- ---------- ---------- ----------
9B4_gi|6322615|       ---------- YVR------- ---------- ---------- ---------- ----------
9C1_gi|2459734|       ---------- WLM------- ---------- ---------- ---------- ----------
10A1_gi|4731595|      ---------- WTL------- ---------- ---------- ---------- ----------
10A2_gi|3256056|      ---------- WTL------- ---------- ---------- ---------- ----------
11A1_gi|1176985|      ---------- WYL------- ---------- ---------- ---------- ----------
11B1_gi|3123233|      ---------- WIL------- ---------- ---------- ---------- ----------
11B2_gi|3123121|      ---------- WIL------- ---------- ---------- ---------- ----------
11B3_gi|85062654|     ---------- WLI------- ---------- ---------- ---------- ----------
11C1_gi|81787577|     ---------- YCL------- ---------- ---------- ---------- ----------
12A1_gi|5305791|      ---------- WLL------- ---------- ---------- ---------- ----------
12B1_gi|2599278|      ---------- WVL------- ---------- ---------- ---------- ----------
12C1_gi|5921163|      ---------- WVL------- ---------- ---------- ---------- ----------
13A1_gi|1351673|      ---------- WLL------- ---------- ---------- ---------- ----------
13B1_gi|9106797|      ---------- WLL------- ---------- ---------- ---------- ----------
13C1_gi|81555851|     ---------- WIL------- ---------- ---------- ---------- ----------
14A1_gi|882530|       ---------- WLL------- ---------- ---------- ---------- ----------
15A1_gi|37196700|     ---------- FPL------- ---------- ---------- ---------- ----------
all2316_aldo/keto     ---------- WLR------- ---------- ---------- ---------- ----------
```

```
                         1321
1A1_gi|5174391|          -------QR- ----KVICIP KSITPSRILQ N--------- ---------- -------IKV
1A2_gi|1703236|          -------QR- ----KVICIP KSVTPSRIPQ N--------- ---------- -------IQV
1A3_gi|1703237|          -------QR- ----KVICIP KSITPSRILQ N--------- ---------- -------IQV
1A4_gi|10946870|         -------QR- ----KVICIP KSINPSRILQ N--------- ---------- -------IQV
1B1_gi|4502049|          -------QR- ----NLVVIP KSVTPERIAE N--------- ---------- -------FKV
1B2_gi|1703235|          -------QR- ----NLVVIP KSVTPARIAE N--------- ---------- -------FQV
1B3_gi|1351911|          -------QR- ----NLVVIP KSVTPVRIAE N--------- ---------- -------LKV
1B4_gi|6978491|          -------QR- ----NLVVIP KSVTPARIAE N--------- ---------- -------FKV
1B5_gi|113594|           -------QR- ----NLIVIP KSVTPERIAE N--------- ---------- -------FQV
1B6_gi|584742|           -------QR- ----NLIVIP KSVTPERIAE N--------- ---------- -------FQV
1B7_gi|231525|           -------QR- ----NVVVIP KSVTPSRIQE N--------- ---------- -------LQV
1B8_gi|6679791|          -------QR- ----NVVVIP KSVTPSRIQE N--------- ---------- -------IQV
1B9_gi|2114406|          -------QR- ----NVVVIP KSVTPARIHE N--------- ---------- -------FQV
1B10_gi|223468663|       -------QR- ----NVIVIP KSVTPARIVE N--------- ---------- -------IQV
1B12_gi|14330324|        -------QR- ----NLAVIP KSDKQQRIKE N--------- ---------- -------MQV
1B13_gi|15864567|        -------QR- ----NVVVIP KSVTPARIQE N--------- ---------- -------IQV
1B14_gi|148540194|       -------QR- ----NVAVIP KSVTLSHIKE N--------- ---------- -------IQV
1B15_gi|51094822|        -------QR- ----NVTVIP KSMTPAHIVE N--------- ---------- -------IQV
1C1_gi|5453543|          -------QR- ----GVVVLA KSYNEQRIRQ N--------- ---------- -------VQV
1C2_gi|4503285|          -------QR- ----GVVVLA KSYNEQRIRQ N--------- ---------- -------VQV
1C3_gi|24497583|         -------QR- ----GVVVLA KSYNEQRIRQ N--------- ---------- -------VQV
1C4_gi|308153631|        -------QR- ----GVVVLA KSYNEQRIRE N--------- ---------- -------IQV
1C5_gi|1352733|          -------QR- ----GIVVLA KSFTEKRIKE N--------- ---------- -------IQV
1C6_gi|13487925|         -------QR- ----GVVVLA KSFSEKRIKE N--------- ---------- -------MQV
1C7_gi|129896|           -------QR- ----GVVVLA KSFNKKRIKE N--------- ---------- -------MQV
1C8_gi|1709623|          -------ER- ----GIVTLV KSFNEERIRE N--------- ---------- -------LQV
1C9_gi|118634|           -------QR- ----GVVPLI RSFNAKRIKE L--------- ---------- -------TQV
1C10a_gi|1345830|        -------QK- ----GIVVLA KSFTPARIKQ N--------- ---------- -------LGV
1C10b_gi|1706132|        -------QK- ----GIVVLA KSFTPARIKQ N--------- ---------- -------LGV
1C11_gi|1669605|         -------QR- ----GVVVLA KSYNKKRIKE N--------- ---------- -------IQV
1C12_gi|85719330|        -------QR- ----GIVPLA QSFKENEMRE N--------- ---------- -------LQV
1C13_gi|171846276|       -------QR- ----GIVPLA QSFKENEMRE N--------- ---------- -------LQV
1C14_gi|19527294|        -------QR- ----GIVVLT RSFKEKRIKE F--------- ---------- -------MKV
1C15_gi|741804|          -------QR- ----GVVVLA KSFNEKRIIE N--------- ---------- -------FQV
1C16_gi|741803|          -------QR- ----GVVPLA QSFKENEMRE N--------- ---------- -------LQV
1C17_gi|741805|          -------QR- ----EVVPLA QSFKENEMRE N--------- ---------- -------LQV
1C18_gi|1654715|         -----AAGCC AGG-GCTCAA GTACAAGCCT G--------- ---------- -------TGT
1C20_gi|16905111|        -------QR- ----GVVVLA KSFIEKRIKE N--------- ---------- -------MQV
1C21_gi|126090770|       -------QR- ----GIVVLN TSLKEERIKE N--------- ---------- -------MQV
1C22_gi|38603389|        -------QR- ----GIVPLA QSFKENEMRE N--------- ---------- -------LQV
1C23_gi|62856987|        -------QR- ----GVVVLA KSYNEKRIKE N--------- ---------- -------VQA
1C24_gi|84993586|        -------QR- ----GVVPLA QSFKENEMRE N--------- ---------- -------LQV
1C25_gi|15216337|        -------QR- ----GVVVLA KSYNEQRIRE N--------- ---------- -------VQV
1D1_gi|5174695|          -------QR- ----GVVVIP KSFNLERIKE N--------- ---------- -------FQI
1D2_gi|398962|           -------QR- ----GLVVIP KSTTPERIKE N--------- ---------- -------FQI
1D3_gi|5689216|          -------QR- ----GVVVIP KSFNPERIKE N--------- ---------- -------FQI
1E1_gi|1698718|          -------QR- ----NLIVIP KSVTPSRIRE N--------- ---------- -------IQV
1E2_gi|269849539|        -------QR- ----NVIVIP GSITPSHIKE N--------- ---------- -------IQV
1G1_gi|17550248|         -------DR- ----GCAILP KSIQENRIKE N--------- ---------- -------FEV
2A1_gi|134153|           -------QR- ----KTAVIP KSSKIQRLKE N--------- ---------- -------LEV
2A2_gi|1835701|          -------QR- ----NTIVIP KSSKTKRLEE N--------- ---------- -------INI
2B1_gi|401428|           -------QR- ----GIAIIP KSNTVPRLLE N--------- ---------- -------KDV
2B2_gi|1351442|          -------QN- ----GIAIIP KSSKKERLLD N--------- ---------- -------LRI
2B3_gi|2492803|          -------QR- ----GIAIIP KSTFPNTLAV N--------- ---------- -------LHV
2B4_gi|1912051|          -------QR- ----NIAVIP KSNNPERLAQ N--------- ---------- -------LSV
2B5_gi|3289019|          -------QR- ----GIAVIP KSNLPERLVQ N--------- ---------- --------RS
2B6_gi|6321895|          -----DI--- ----IVVNNS QSYKNSPINE DEFVNKDWEY YRSKKVVINY KILISFALNV
2B7_gi|1912049|          -------QR- ----NIAVIP KSNNPERLAQ N--------- ---------- -------LSV
2B8_gi|4103055|          -------QR- ----GLAVIP KSNNPDRLLS N--------- ---------- -------LKV
2C1_gi|1332539|          -------DR- ----EFAVIP KSVNAGRMKA N--------- ---------- -------LEI
2D1_gi|7407095|          -------QR- ----GIAVIP KSNNPQRLKQ N--------- ---------- -------LDV
2E1_gi|4753912|          -------DR- ----KVVPIP KTVSPKRLLE N--------- ---------- -------INI
2E2_gi|18479021|         -------ER- ----SLIPIP KSTNQQRIRE N--------- ---------- -------IDV
2E3_gi|5052610|          -------EI- ----GTIPLP KSSNPKRIEE N--------- ---------- -------FQI
3A1_gi|121087|           -------QR- ----GYVVLP KSVNPDRIKT N--------- ---------- -------RKI
3A2_gi|6320576|          -------QR- ----GYVVLA KSVNPERIVS N--------- ---------- -------FKI
3B1_gi|1142698|          -------VG- ----GHSVIP KSVTPSRIGE N--------- ---------- -------FKQ
3B2_gi|60458781|         -------QR- ----GYSVLV KSVTPSRIKS N--------- ---------- ----------
3B3_gi|60458785|         -------QR- ----GYSVLV KSVTPSRIES N--------- ---------- ----------
3C1_gi|536474|           -------RQ- ----GTIVIP RSLNPVRISS S--------- ---------- -------IEF
3C2_gi|74626610|         -------SK- ----GVIPIT TTSKIERMKE C--------- ---------- -------LN-
3C3_gi|38423524|         -------QR- ----GILPVT TSSKESRLKE S--------- ---------- -------LNL
3D1_gi|31321885|         -------AR- ----GITVIP KSVNPDRIKA N--------- ---------- -------AQL
3E1_gi|22207641|         -------RR- ----GYVVLP KSSNPKRIES N--------- ---------- ----------
3F1_gi|81625481|         -------AKP ----NVVAIP KAGRVEHLRE N--------- ---------- -------LKA
3F2_gi|13638516|         -------GE- ----GYSVIP SSTKRKNLES N--------- ---------- -------LKA
3F3_gi|81635765|         -------QK- ----GVSMNT MSTKPENIRA N--------- ---------- -------FEI
4A1_gi|112837|           -------EQ- ----GVTFVP KSYDKERMNQ N--------- ---------- -------LHI
```

```
4A2_gi|75220959|      -------EQ- ----GVTFVP KSYDKERMNQ N--------- ---------- -------LRI
4A3_gi|1215788|       -------EQ- ----GVTFVA KSYDKERMNQ N--------- ---------- -------LQI
4A4_gi|1514979|       -------EQ- ----GVTFVA KSYDKERMNQ N--------- ---------- -------LQI
4B1_gi|2792155|       -------QQ- ----GSSAMA KSFNKERMKQ N--------- ---------- -------LEI
4B2_gi|6478210|       -------QQ- ----GASLVV KSFNEGRMKE N--------- ---------- -------LKI
4B3_gi|6478204|       -------QQ- ----GASLVV KSFNEARMKE N--------- ---------- -------LKI
4B4_gi|2792295|       -------EQ- ----GVSIVT KSYNKERMRQ N--------- ---------- -------LDI
4B5_gi|112807104|     -------EQ- ----GDCLIV KSFDEGRMKE N--------- ---------- -------LDI
4B6_gi|112807098|     -------EQ- ----GDCLIV KSFDEARMRE N--------- ---------- -------LDI
4B7_gi|112807100|     -------EQ- ----GDCLIV KSFDEARMRE N--------- ---------- -------LDV
4B8_gi|112807102|     -------EQ- ----GDCLIV KSFDEARMRE N--------- ---------- -------LDV
4C1_gi|113595|        -------QR- ----GTSVIP KSSKDERIKE N--------- ---------- -------IQV
4C2_gi|167113|        -------QR- ----GTIVIP KSSKDERIKE N--------- ---------- -------IQV
4C3_gi|75221432|      -------QR- ----GTSVIP KSSKDERIKE N--------- ---------- -------IQA
4C4_gi|4539944|       -------QR- ----GTSVIP KSTNPERIKE N--------- ---------- -------IQV
4C5_gi|13160397|      -------QM- ----GHSVLP KSVHESRIKE N--------- ---------- -------IDV
4C6_gi|13160399|      -------QM- ----GHSVLP KSVHESRIKE N--------- ---------- -------IDV
4C8_gi|111182163|     -------QT- ----GHSVLP KSSSGARLKE N--------- ---------- -------LDV
4C9_gi|111182165|     -------QM- ----GHSVLP KSTNEGRIKE N--------- ---------- -------FNV
4C10_gi|111182167|    -------QM- ----GQSVLP KSTHEDRIKQ N--------- ---------- -------FDV
4C11_gi|111182169|    -------QM- ----GHSILP KSTNEGRIRE N--------- ---------- -------FDV
5A1_gi|408360251|     -------QK- ----NLITIP KSVHRERIEE N--------- ---------- -------ADI
5A2_gi|11127591|      -------QA- ----GVITIP KSGNEARIKE N--------- ---------- -------GNI
5B1_gi|2506173|       -------QN- ----DVVAIP KSVNPERIAK N--------- ---------- -------IDV
5C1_gi|144969|        -------QK- ----GFVVFP KSVRRERLEE N--------- ---------- -------LDV
5C2_gi|3916039|       -------DS- ----GLVVIP KSVTPSRIAE N--------- ---------- -------FDV
5D1_gi|112735|        -------QL- ----GSTPIP KSADPDRQRE N--------- ---------- -------ADV
5E1_gi|5354195|       -------QL- ----GAIPIP KASSKERQIE N--------- ---------- -------LSL
5F1_gi|82504416|      -------QR- ----GIVSLA KSVRKERMEE N--------- ---------- -------INI
5G1_gi|16080393|      -------QH- ----GVVTIP KSIKEHRIIE N--------- ---------- -------ADI
5G2_gi|16079957|      -------QH- ----GIITIP KSTKEHRIKE N--------- ---------- -------ASV
6A1_gi|18202524|      -------RNE G---VSSVLL GASSADQLME N--------- ---------- ----IGAIQV
6A2_gi|499328|        -------RN- -EG-VSSVLL GASNAEQLME N--------- ---------- ----IGAIQV
6A3_gi|2135947|       -------RN- -EG-VSSVLL GSSTPEQLIE N--------- ---------- ----LGAIQV
6A4_gi|975314|        -------RNE G---VSSVLL GASNAEQLME N--------- ---------- ----IGAIQV
6A5_gi|2827466|       -------RNE -G--VSSVLL GASNADQLME N--------- ---------- ----IGAIQV
6A6_gi|7914984|       -------RN- -E-GVSSVLL GASNAEQLME N--------- ---------- ----IGAIQV
6A7_gi|5019764|       -------RNE G---VSSVLL GSSTPEQLIE N--------- ---------- ----LGAIQV
6A8_gi|148747467|     -------RNE -G--VSSVLL GSSTPEQLIE N--------- ---------- ----LGAIQV
6A9_gi|24648619|      SDRERPRFDF PSQPQPTPIP QQPPVNPTEA D--------- ---------- --------EV
6A10a_gi|726465|      -------RNE G---VSSVLL GSSTPEQLIE N--------- ---------- ----LGAIQV
6A10b_gi|5019766|     -------RNE -G--VSSVLL GSSTPEQLIE N--------- ---------- ----LGAIQV
6A11_gi|5922729|      -----RSEG- ----VSSVLL GVSNIEQLLE N--------- ---------- -------LGA
6A13_gi|52001073|     -------RN- -E-GVSSVLL GSSTPEQLIE N--------- ---------- ----LGAIQV
6A14_gi|226823214|    -------RSE G---VSSVLL GVSSAEQLME H--------- ---------- ----LGSLQV
6B1_gi|902000|        -------KHE P---VQCLLL GATSAEQLHQ S--------- ---------- ----LQSLQL
6C1_gi|1063415|       -----SNPN- ----VSSVIT GATRGSQIQE N--------- ---------- -------MKA
6C2_gi|2832783|       -----SNPH- ----VSSVIT GATKESQIIE N--------- ---------- -------MKA
7A1_gi|39932720|      -----HHSQL KGTQGDAVIL GMSSLEQLEQ N--------- ---------- -------LAL
7A2_gi|41327764|      -----HHSQL QGAHGDAVIL GMSSLEQLEQ N--------- ---------- -------LAA
7A3_gi|41152114|      -----HHSQL QGAHGDAVIL GMSSLEQLEQ N--------- ---------- -------LAA
7A4_gi|6815049|       -----HHSQL QGTRGDAVIL GMSSLEQLEQ N--------- ---------- -------LAA
8A1_gi|24638123|      ----ASGNG- ----LVIPIP GSTSVSRTKS N--------- ---------- -------LNA
8A2_gi|74627022|      -----ANGKG ----MIIPIP GSTTVQRAES N--------- ---------- -------LSA
9A1_gi|146345520|     -----HKSP- ----YVFPVI GCRTVEQLEA N--------- ---------- -------ITS
9A2_gi|6093525|       -----HK--- -APYVF-PVI GGRKVEHLKE N--------- ---------- -------IEA
9A3_gi|2492798|       -----QKFP- ----YVFPIV GGRKVEHLYA N--------- ---------- -------LEA
9B1_gi|6323998|       -----SKAK- ----NVFPLI GGRKIEHLKQ N--------- ---------- -------IEA
9B2_gi|6319951|       -----SKAK- ----NFFPSV EGGKIEDLKE N--------- ---------- -------IKA
9B3_gi|6319958|       -----SKAK- ----NVFPLV GGRKIEHLKQ N--------- ---------- -------IEA
9B4_gi|6322615|       -----SKAK- ----HVFPLV GGRKIEHLKQ N--------- ---------- -------IEA
9C1_gi|2459734|       -----HRDG- ----VTAPIV GARTVEQLTE N--------- ---------- -------LEA
10A1_gi|4731595|      -----SQPF- ----PVVPLI GPRQPQEVRD S--------- ---------- -------LAS
10A2_gi|3256056|      -----SQPF- ----RVVALI GPRQPEEVRD S--------- ---------- -------LAA
11A1_gi|1176985|      -------ARP ---EIDILIP GAKRADQLID N--------- ---------- -------IKT
11B1_gi|3123233|      -------DQP ---GADIALW GARKPGQLEA L--------- ---------- -------SEI
11B2_gi|3123121|      -------KQS D---LISILS GATAPEQVRE N--------- ---------- -------VAA
11B3_gi|85062654|     -------RQ- ---PQTNAIV GARNAQQAIA N--------- ---------- -------AQA
11C1_gi|81787577|     -------AHD ---VVATVAA GASSIDQVKA N--------- ---------- -------VQA
12A1_gi|5305791|      -------SRP G---VSGAVI GPRTTGHLVS A--------- ---------- -------LRA
12B1_gi|2599278|      -------SRP ---GIAGAVI GPRTPEQLDS A--------- ---------- -------LKA
12C1_gi|5921163|      -------SRP G---VTGLVI GPRTEQHVDG A--------- ---------- -------LHA
13A1_gi|1351673|      -----AQGD- ----DILPIP GTKRVKYLEE N--------- ---------- -------FGA
13B1_gi|9106797|      -----KRSP- ----VILPIP GTSKVAHLEE N--------- ---------- -------VAA
13C1_gi|81555851|     -----HTQK- ----IIVPLF GTTKESRLIE N--------- ---------- -------IGA
14A1_gi|882530|       -----KDDR- ----VTSVLI GASRAEQLEE N--------- ---------- -------VQA
15A1_gi|37196700|     -----RHEA- ----VSSILI GVRSPEQIRQ N--------- ---------- -------VVW
all2316_aldo/keto     -----AKSP- ----AILPIP GASKISSIED S--------- ---------- -------VSA
```

```
                   1381
1A1_gi|5174391|      FD-FT-FSPE EMKQLNA--- -LNK------ -NW---RYIV PMLTVDGKRV PRDAGHPLYP
1A2_gi|1703236|      FD-FT-FSPE EMKQLDA--- -LNK------ -NL---RFIV PMLTVDGKRV PRDAGHPLYP
1A3_gi|1703237|      FD-FT-FSPE EMKQLDA--- -LNK------ -NW---RYIV PMITVDGKRV PRDAGHPLYP
1A4_gi|10946870|     FD-FT-FSPE EMKQLDA--- -LNK------ -NW---RYIV PMITVDGKRV PRDAGHPLYP
1B1_gi|4502049|      FD-FE-LSSQ DMTTLLS--- -YNR------ -NW---RVCA LLSC------ ---TSHKDYP
1B2_gi|1703235|      FD-FE-LSSE DMTTLLS--- -YNR------ -NW---RVCA LVSC------ ---ASHKDYP
1B3_gi|1351911|      FD-FE-VSSE DMATLLS--- -YNR------ -NW---RVCA LMSC------ ---AKHKDYP
1B4_gi|6978491|      FD-FE-LSNE DMATLLS--- -YNR------ -NW---RVCA LMSC------ ---AKHKDYP
1B5_gi|113594|       FD-FE-LDKE DMNTLLS--- -YNR------ -DW---RACA LVSC------ ---ASHRDYP
1B6_gi|584742|       FD-FE-LSPE DMNTLLS--- -YNR------ -NW---RVCA LMSC------ ---ASHKDYP
1B7_gi|231525|       FD-FQ-LSEE DMAAILS--- -FNR------ -NW---RACD LLDA------ ---RTEEDYP
1B8_gi|6679791|      FD-FQ-LSDE EMATILS--- -FNR------ -NW---RACL LPET------ ---VNMEEYP
1B9_gi|2114406|      FD-FQ-LSDQ EMATILG--- -FNR------ -NW---RACL LPET------ ---VNMEEYP
1B10_gi|223468663|   FD-FK-LSDE EMATILS--- -FNR------ -NW---RACN VLQS------ ---SHLEDYP
1B12_gi|14330324|    FD-FE-LSKK EMDVILS--- -FNR------ -NW---RAIP VPQS------ ---ANHKDYP
1B13_gi|15864567|    FD-FQ-LSDQ EMATILS--- -FNR------ -NW---RACL LPET------ ---VNMEEFP
1B14_gi|148540194|   FD-FQ-LSEE DMAAILS--- -LNR------ -NW---RACG LFVT------ ---SDEEDFP
1B15_gi|51094822|    FD-FK-LSDE EMATILS--- -FNR------ -NW---RAFD FKEF------ ---SHLEDFP
1C1_gi|5453543|      FE-FQ-LTSE EMKAIDG--- -LNR------ -NV---RYLT LDIF------ ---AGPPNYP
1C2_gi|4503285|      FE-FQ-LTSE EMKAIDG--- -LNR------ -NV---RYLT LDIF------ ---AGPPNYP
1C3_gi|24497583|     FE-FQ-LTAE DMKAIDG--- -LDR------ -NL---HYFN SDSF------ ---ASHPNYP
1C4_gi|308153631|    FE-FQ-LTSE DMKVLDG--- -LNR------ -NY---RYVV MDFL------ ---MDHPDYP
1C5_gi|1352733|      FE-FQ-LPSE DMKVIDS--- -LNR------ -NF---RYVT ADFA------ ---IGHPNYP
1C6_gi|13487925|     FE-FQ-LTSE DMKVLDD--- -LNK------ -NI---RYIS GSSF------ ---KDHPDFP
1C7_gi|129896|       FD-FE-LTPE DMKAIDG--- -LNR------ -NI---RYYD FQKG------ ---IGHPEYP
1C8_gi|1709623|      FD-FQ-LASD DMEILDN--- -LDR------ -NL---RYFP ANMF------ ---KAHPNFP
1C9_gi|118634|       FE-FQ-LASE DMKALDG--- -LNR------ -NF---RYNN AKYF------ ---DDHPNHP
1C10a_gi|1345830|    FE-FE-LKPE DMKSLES--- -LDR------ -NL---HYGP FREV------ ---KQHPEYP
1C10b_gi|1706132|    FE-FE-LKPE DMKTLES--- -LDR------ -NL---HYGP FREV------ ---KQHPEYP
1C11_gi|1669605|     FD-FE-LTPE DMKAIDG--- -LNS------ -NM---RYNE LLLG------ ---VGHPEYP
1C12_gi|85719330|    FE-FQ-LSPE DMKTLDG--- -LNK------ -NF---RYLP AEFL------ ---ADHPEYP
1C13_gi|171846276|   FG-FQ-LSPE DMKTLDG--- -LNK------ -NF---RYLP AEFL------ ---VDHPEYP
1C14_gi|19527294|    FE-FQ-LASE DMKVLDG--- -LHR------ -NL---RYNT ASYF------ ---DDHPNHP
1C15_gi|741804|      FD-FE-LTPE DMKTIDS--- -LNR------ -NF---RYSQ MAFA------ ---LDHPDYP
1C16_gi|741803|      FD-FQ-LSPE DMKTLDG--- -LNK------ -NF---RYLS AEFL------ ---AGHPEYP
1C17_gi|741805|      FD-FQ-LHPE DMKTLDG--- -LNK------ -NF---RYLS AEFL------ ---AGHPGCP
1C18_gi|1654715|     GCAAC-CAGG TAGAATGCCA TCTTTATCTC ACCAGAGCAA GCTGCTGGCC TACTGCAAGA
1C20_gi|16905111|    M--------- ---------- ---------- ---------- -SCF------ ----------
1C21_gi|126090770|   FE-FQ-LSSE DMKVLDG--- -LNR------ -NM---RYIP AAIF------ ---KGHPNWP
1C22_gi|38603389|    FG-FQ-LSPE DMKTLDG--- -LNK------ -NF---RYLP AEFL------ ---VDHPEYP
1C23_gi|62856987|    FG-FQ-LTSE DMKVLDG--- -LNR------ -NL---RYVT LEMF------ ---AGHPEYP
1C24_gi|84993586|    FE-FQ-LSPE DMKTLDG--- -LNK------ -NF---RYLS AEFL------ ---ADHPEYP
1C25_gi|15216337|    FE-FQ-LTSE DMKVLDD--- -LNR------ -NF---RYVV MDFL------ ---VDHPDYP
1D1_gi|5174695|      FD-FS-LTEE EMKDIEA--- -LNK------ -NV---RFVE LLMW------ ---RDHPEYP
1D2_gi|398962|       FD-FS-LTKE EMKDIEA--- -LNK------ -NV---RFVE MLMW------ ---SDHPEYP
1D3_gi|5689216|      FD-FS-LTEE EMKDIEA--- -LNK------ -NV---RYVE LLMW------ ---RDHPEYP
1E1_gi|1698718|      FD-FE-LTEK DMEELLS--- -LDK------ -NL---RFAT FPTT------ ---ENHQDYP
1E2_gi|269849539|    FD-FE-LTQH DMDNILS--- -LNR------ -NL---RLAM FPIT------ ---KNHKDYP
1G1_gi|17550248|     FD-FS-LTEE DIAKLEE--- ---------- -SKNSQRLFL QDFM------ ---TGHPEDA
2A1_gi|134153|       LE-FQ-LSDE DMQLIYS--- -IDR------ -KY---RTSL PSKT------ ---WGLDVYA
2A2_gi|1835701|      FD-FE-LSKE DMELIKT--- -MER------ -NQ---RSNT PAKA------ ---WGIDVYA
2B1_gi|401428|       NS-FD-LDEQ DFADIAK--- -LDI------ -NL---RFND PWDW------ ------DKIP
2B2_gi|1351442|      NDALT-LTDD ELKQISG--- -LNQ------ -NI---RFND PWEW------ -----LDNEFP
2B3_gi|2492803|      DE-FD-LTKE DFEEIAK--- -LDR------ -HL---RFND PWTW------ ------DKIP
2B4_gi|1912051|      VD-FD-LTKD DLDNIAK--- -LDI------ -GL---RFND PWDW------ ------DNIP
2B5_gi|3289019|      FNTFD-LTKE DFEEIAK--- -LDI------ -GL---RFND PWDW------ ------DNIP
2B6_gi|6321895|      LLNYH-FGFT DLRSLCN--- -VNDQ----- ------RFCI PVFINDEFVD ADTVNAVFIK
2B7_gi|1912049|      VD-FD-LTKD DLDNIAK--- -LDI------ -GL---RFND PWDW------ ------DNIP
2B8_gi|4103055|      ND-FD-LSQE DFQEISK--- -LDI------ -EL---RFNN PWDW------ ------DKIP
2C1_gi|1332539|      LD-IK-LDAE DNKTLDS--- -LKT------ -NQ---RFND PMTY------ -----GFGLP
2D1_gi|7407095|      TG-WN-LEEE EIKAISG--- -LDR------ -GL---RFND PLGY------ -----GLYAP
2E1_gi|4753912|      FD-FK-LKEE EIEKINQ--- -FNS------ -NT---RYTL PSFW------ ---QKHPFYP
2E2_gi|18479021|     FD-FQ-LSPE DINAIGK--- -LDK------ ---------- ---------- -------DLS
2E3_gi|5052610|      FD-FQ-LDAE DHAILDS--- -YNT------ -GE---RLIP MTHA------ ---IKSKNYP
3A1_gi|121087|       FT----LSTE DFEAINN--- -ISK------ -EKGEKRVVH PNWS------ ---------P
3A2_gi|6320576|      FT----LPED DFKTISN--- -LSK------ -VHGTKRVVD MKWG------ -------SFP
3B1_gi|1142698|      VS----LSQE DVDAVSK--- -LGE------ -GSGRRRYNI PCTYSPKWDI NVFGEEDEKS
3B2_gi|60458781|     FEQIT-LSDE EFQRVTN--- -LIKEYGESR NNV---PFNY KPSWSIDVFG TQDEAKATHK
3B3_gi|60458785|     YDQIT-LSPE EFQKVTD--- -LIKEYGESR NNI---PLNY KPSWPISVFG TSDEAKATHK
3C1_gi|536474|       AS----LTKD ELQELND--- -FGEKY---- -PV---RFID EPFA------ ---AILPEFT
3C2_gi|74626610|     FDSFT-LDKA DIDELGT--- -LGV------ -QHHKRTFMK HMDE------ ----------
3C3_gi|38423524|     FD-FE-LTDE EVNEINK--- -IGD------ ---------- ---------- ---ANPYRAF
3D1_gi|31321885|     KD----LDAE DMKLLNDYSE QLAK------ -DGKLNRYVF PPFG------ ------TDFG
3E1_gi|22207641|     FKSIE-LSDA DFEAINA--- -VAK-----G RHF---RFVN MKDT------ ---FGYDVWP
3F1_gi|81625481|     TE-IK-LSEE EMKLLDS--- -LG------- ---------- ---------- ----------
3F2_gi|13638516|     QN-LQ-LDAE DKKAIAA--- -LDC------ -ND---RLVS PEGL------ --------AP
3F3_gi|81635765|     LD-FA-LSPH DMKRIDA--- -MNA------ TNY---RILK AGML------ ------PWVP
4A1_gi|112837|       FD-WA-LTEQ DHHKISQ--- -ISQ------ -----SRLIS GPTK------ -----PQLAD
```

```
4A2_gi|75220959|     FD-WS-LTKE DHEKIDQ--- -IKQ------ -----NRLIP GPTK------ -----PGLND
4A3_gi|1215788|      FD-WE-LTTE DHQKIDQ--- -IKQ------ -----NRLIP GPTK------ -----PQLND
4A4_gi|1514979|      FD-WE-LTTE DHQKIDQ--- -IKQ------ -N----RLIP GPTK------ -----PQLND
4B1_gi|2792155|      FD-FE-LSEE ELEKIKQ--- -IPQ------ -----RRQYT GDMW----LS ENGSCKTLEE
4B2_gi|6478210|      FD-WE-LTAE DMEKISE--- -IPQ------ SRTSSAAFLL SPTG------ ---PFKTEEE
4B3_gi|6478204|      FD-WE-LTAE DMEKISE--- -IPQ------ SRTSSAAFLL SPTG------ ---PFKTEEE
4B4_gi|2792295|      FD-FC-LTEE ELEKMSH--- -LPQ------ ------RKGV TFAS------ ILGPHDIVLE
4B5_gi|112807104|    VD-WE-LSEE ERQRISK--- -IPQ------ RKINQGRRYV SEHG------ ---PYKSFEE
4B6_gi|112807098|    VG-WE-LTEE ERQRIAG--- -IPQ------ ------RKIN RALR---FVS DHGPYKSLDD
4B7_gi|112807100|    DG-WE-LTEE ERRRIAE--- -IPQ------ RKINLGKRYV SDHG------ ---PYKSLEE
4B8_gi|112807102|    DG-WE-LTEE ERRRIAE--- -IPQ------ RKINLGKRYV SEHG------ ---PYKSLEE
4C1_gi|113595|       FG-WE-IPEE DFKVLCS--- -IKDEKRVLT GEE---LFVN KTHG------ ---PYRSAAD
4C2_gi|167113|       FG-WE-IPEE DFQVLCS--- -IKDEKRVLT GEE---LFVN KTHG------ ---PYKSASE
4C3_gi|75221432|     FG-WE-IPED DFQVLCS--- -IKDEKRVLT GEE---LFVN KTHG------ ---PYKSASE
4C4_gi|4539944|      FG-WE-IPAE DFQILSS--- -LSEQKRVLD GED---LFVN KTHG------ ---PFRSAAE
4C5_gi|13160397|     FS-WC-IPDD LFAKFSE--- -IEQ------ ---------- ---------- -VSPGKPEFP
4C6_gi|13160399|     FS-WC-IPDV LFAKFSE--- -IEQ------ ---------- ---------- -VSPGKPEFP
4C8_gi|111182163|    FD-WS-IPED LFTKFSN--- -IPQ------ -----EKFCR ATEF---AHE THGFYKTIEE
4C9_gi|111182165|    FD-WS-IPDY MFAKFAE--- -IEQ------ -----ARLVT GSFL---VHE TLSPYKSIEE
4C10_gi|111182167|   FN-WS-IPED MLSKFSE--- -IGQ------ -----GRLVR GMSF----VH ETSPYKSLEE
4C11_gi|111182169|   LG-WS-IPKE MFDKFSK--- -IEQ------ -----ARLVQ GTSF---VHE TLSPYKTLEE
5A1_gi|408360251|    FD-FE-LGAE DVMSIDA--- -LNT------ -NS---RYGP DPDE------ ----------
5A2_gi|11127591|     FD-FE-LTAE DIQVIDG--- -MNA------ -GH---RYGP DPEV------ ----------
5B1_gi|2506173|      FD-FA-LSDA EMAQLDE--- -LDT------ ------GVRI GPDP------ ---RDVDTSS
5C1_gi|144969|       FD-FD-LTDT EIAAIDA--- -MDP------ -GDGSGRVSA HPDEVD---- ----------
5C2_gi|3916039|      WD-FR-LDKD ELGEIAK--- -LDQ------ ---------- ---------- ----GKRLGP
5D1_gi|112735|       FG-FA-LTAD QVDAISG--- -LER------ ---------- GRLW------ -----DGDPD
5E1_gi|5354195|      FD-FE-LSPQ DVEIIAT--- -LAR------ --------PD GRLA------ -----DQDPA
5F1_gi|82504416|     LD-FE-LSGE DMLQIAA--- -LDT----AT SAFFSHRDPA MVEW------ ---LTGRKLD
5G1_gi|16080393|     FD-FE-LSQE DMDKIDA--- -LNK------ ---------D ERVG------ ---PNPDELL
5G2_gi|16079957|     FD-FE-LTQD DMNRIDA--- -LNE------ -NL---RVGP DPDN------ --------FD
6A1_gi|18202524|     LP--K-LSSS IIHEIDS--- ---------- ------ILGN KPYS------ -----KKDYR
6A2_gi|499328|       LP--K-LSSS IVHEIDS--- ---------- ------ILGN KPYS------ -----KKDYR
6A3_gi|2135947|      LP--K-MTSH VVNEIDN--- ---------- ------ILRN KPYS------ -----KKDYR
6A4_gi|975314|       LP--K-LSSS IVHEIDS--- ---------- ------ILGN KPYS------ -----KKDYR
6A5_gi|2827466|      LP--K-LSSS IIHEIDS--- ---------- ------ILGN KPYS------ -----KKDYR
6A6_gi|7914984|      LP--K-LSSS IIHEIDS--- ---------- ------ILGN KPYS------ -----KKDYR
6A7_gi|5019764|      LP--K-MTSH VVNEIDN--- ---------- ------ILRN KPYS------ -----KKDYR
6A8_gi|148747467|    LP--K-MTSH VVNEIDN--- ---------- ------ILRN KPYS------ -----KKDYR
6A9_gi|24648619|     IE-SI-LNSQ DAQLLEK--A FETQMQINAQ GKTAEKRHID EFYYKQFAVP LRQFFEELKT
6A10a_gi|726465|     LP--K-MTSH VVNEIDN--- ---------- ------ILRN KPYS------ -----KKDYR
6A10b_gi|5019766|    LP--K-MTSH VVNEIDN--- ---------- ------ILRN KPYS------ -----KKDYR
6A11_gi|5922729|     IQVLPHLTPQ LVTEIDQ--- ---------- ------ILGN KPNL------ -----KKDSR
6A13_gi|52001073|    LP--K-MTSH VVNEIDN--- ---------- ------ILRN KPYS------ -----KKDYR
6A14_gi|226823214|   LS--Q-LTPQ TVVEIDA--- ---------- ------LLGN KSHSKK---- ----------
6B1_gi|902000|       LP--R-LSSS VMLELER--- ---------- ------ILEN KPVR------ -----PPMIS
6C1_gi|1063415|      VDVIPLLTPI VLDKIEQ--- ---------- ---------- ---------- ---VIQSKPK
6C2_gi|2832783|      IDVIPLLTPE VLEKIEA--- ---------- ---------- ---------- ---VVQSKPK
7A1_gi|39932720|     VEEGP-LEPA VVDAFDQ--- ---------- -AWNLVAHEC PNYFR----- ----------
7A2_gi|41327764|     TEEGP-LEPA VVDAFNQ--- ---------- -AWHLVAHEC PNYFR----- ----------
7A3_gi|41152114|     AEEGP-LEPA VVDAFNQ--- ---------- -AWHLVTHEC PNYFR----- ----------
7A4_gi|6815049|      TEEGP-LEPA VVEAFNQ--- ---------- -AWNVVAHEC PNYFR----- ----------
8A1_gi|24638123|     LN-KS-LSPE QFKEAKE--- ---------- ------VLSK YPIY------ -------GLR
8A2_gi|74627022|     LK-KS-LSSE QLEEAKK--- VLDK------ ------HQIF GLRY------ ---NKQLEST
9A1_gi|146345520|    LG-VE-LSDE EIYEIEDTIP FDVGFPMAFL FESPQQKYRS DMTTRHIWQV TCNARIESVP
9A2_gi|6093525|      LG-LV-LSEE EIREIDDAEP FDVGFPMNFS FETPTQSYRT NMTSKDIWQL SCNTRLETVP
9A3_gi|2492798|      LD-IS-LSPE QMQFLND-TV PFNK------ -GFPYLLFGD GSDY--NIVH KAAGHYDKWP
9B1_gi|6323998|      LS-IK-LTPE QIEYLES--- -IVP------ FDVGFPKSLI GDDPAVTKKL SPLTSMSARI
9B2_gi|6319951|      LS-ID-LTPD NIKYLES--- ---------- -------IVP FDIGFPNNFI VLNSLTQKYG
9B3_gi|6319958|      LS-IK-LTPE QIEYLES--- -IIPFDVGFP TNF---IGDD PAVTKKASLL TAMSAQISFD
9B4_gi|6322615|      LS-IK-LTPE QIKYLES-IV PFDV------ -GFPTNFIGD DPAV------ ---TKKPSFL
9C1_gi|2459734|      AT-ID-LTDE QVDRLTG--- ---------- ------AKPD PYVGL----- ----------
10A1_gi|4731595|     AA-LR-LTEA ERDWLET--- ---------- ------GKGT MPPV------ ----------
10A2_gi|3256056|     AE-LR-LTEA ERDWLEN--- ---------- ------GTGE PPRE------ ----------
11A1_gi|1176985|     AD-VT-LSQE DISFIDK--- -LFA------ ---------- ---------- ----------
11B1_gi|3123233|     TG-WT-LNSE DQKDINT--- -ILE------ ------NTIS DPVG------ ---PEFMAPP
11B2_gi|3123121|     LN-IN-LSDA DATLMREMAE ALER------ ---------- ---------- ----------
11B3_gi|85062654|    ID-VE-LTAK DLEAIDH--- -IGR------ ------TVTD PLDE------ --------NP
11C1_gi|81787577|    VEATP-LTAE ERQHIQK--- -LAK------ ---------- ---------- -----AAVYE
12A1_gi|5305791|     VE-LE-LSEE EHRELEA--- ---------- --------LF PPVG------ ---SGGEVPE
12B1_gi|2599278|     SA-MT-LDEQ ALSELDE--- ---------- --------IF PAVA------ ---SGGAAPE
12C1_gi|5921163|     LR-TP-LPEP VLARLEE--- ---------- --------LF PPVG------ ---RGGSAPD
13A1_gi|1351673|     LK-VK-LSDA TVKEIRE--- ---------- -ACDNAEVIG ARYP------ ---PGAGSKI
13B1_gi|9106797|     AA-IT-LSDE EFAELDA--- ---------- ---------A APRG------ ----------
13C1_gi|81555851|    LQ-VS-WSQK ELEIFQK--- ---------- -ELTAIKIEG ARYP------ -----ERINE
14A1_gi|882530|      LNNLT-FSTK ELAQIDQ--- ---------- ------HIAD GELN------ -------LWQ
15A1_gi|37196700|    FE-QS-IPDE FWTTLRS--- ---------- ---------E GLIS------ ----------
all2316_aldo/keto    VN-VQ-LSDE EVQKI----- ---------- ---------- ---------- ----------
```

```
                     1441
1A1_gi|5174391|      FNDPY----- ---------- ---------- ---------- ---
1A2_gi|1703236|      FNDPY----- ---------- ---------- ---------- ---
1A3_gi|1703237|      FNDPY----- ---------- ---------- ---------- ---
1A4_gi|10946870|     FNDPY----- ---------- ---------- ---------- ---
1B1_gi|4502049|      FHEEF----- ---------- ---------- ---------- ---
1B2_gi|1703235|      FHAEF----- ---------- ---------- ---------- ---
1B3_gi|1351911|      FHAEV----- ---------- ---------- ---------- ---
1B4_gi|6978491|      FHAEV----- ---------- ---------- ---------- ---
1B5_gi|113594|       FHEEF----- ---------- ---------- ---------- ---
1B6_gi|584742|       FHEEY----- ---------- ---------- ---------- ---
1B7_gi|231525|       FHEEY----- ---------- ---------- ---------- ---
1B8_gi|6679791|      YDAEY----- ---------- ---------- ---------- ---
1B9_gi|2114406|      YDAEY----- ---------- ---------- ---------- ---
1B10_gi|223468663|   FNAEY----- ---------- ---------- ---------- ---
1B12_gi|14330324|    FNAEY----- ---------- ---------- ---------- ---
1B13_gi|15864567|    YDAEY----- ---------- ---------- ---------- ---
1B14_gi|148540194|   FHEEY----- ---------- ---------- ---------- ---
1B15_gi|51094822|    FDAEY----- ---------- ---------- ---------- ---
1C1_gi|5453543|      FSDEY----- ---------- ---------- ---------- ---
1C2_gi|4503285|      FSDEY----- ---------- ---------- ---------- ---
1C3_gi|24497583|     YSDEY----- ---------- ---------- ---------- ---
1C4_gi|308153631|    FSDEY----- ---------- ---------- ---------- ---
1C5_gi|1352733|      FSDEY----- ---------- ---------- ---------- ---
1C6_gi|13487925|     FWDEY----- ---------- ---------- ---------- ---
1C7_gi|129896|       FSEEY----- ---------- ---------- ---------- ---
1C8_gi|1709623|      FSDEY----- ---------- ---------- ---------- ---
1C9_gi|118634|       FTDE------ ---------- ---------- ---------- ---
1C10a_gi|1345830|    FHDEY----- ---------- ---------- ---------- ---
1C10b_gi|1706132|    FHDEY----- ---------- ---------- ---------- ---
1C11_gi|1669605|     FVEEY----- ---------- ---------- ---------- ---
1C12_gi|85719330|    FSEEY----- ---------- ---------- ---------- ---
1C13_gi|171846276|   FVEEY----- ---------- ---------- ---------- ---
1C14_gi|19527294|    FTDEY----- ---------- ---------- ---------- ---
1C15_gi|741804|      FLEEY----- ---------- ---------- ---------- ---
1C16_gi|741803|      FSEEY----- ---------- ---------- ---------- ---
1C17_gi|741805|      FSEEY----- ---------- ---------- ---------- ---
1C18_gi|1654715|     TGAATGACAT TGTTCTGGTT GCTATGGTGC CCTGGGAACC CAA
1C20_gi|16905111|    ---GY----- ---------- ---------- ---------- ---
1C21_gi|126090770|   FLDEY----- ---------- ---------- ---------- ---
1C22_gi|38603389|    FVEEY----- ---------- ---------- ---------- ---
1C23_gi|62856987|    FSDDY----- ---------- ---------- ---------- ---
1C24_gi|84993586|    FSEEY----- ---------- ---------- ---------- ---
1C25_gi|15216337|    FSDEY----- ---------- ---------- ---------- ---
1D1_gi|5174695|      FHDEY----- ---------- ---------- ---------- ---
1D2_gi|398962|       FHDEY----- ---------- ---------- ---------- ---
1D3_gi|5689216|      FNDEY----- ---------- ---------- ---------- ---
1E1_gi|1698718|      FHIEY----- ---------- ---------- ---------- ---
1E2_gi|269849539|    FHIEY----- ---------- ---------- ---------- ---
1G1_gi|17550248|     FAAERK---- ---------- ---------- ---------- ---
2A1_gi|134153|       ---------- ---------- ---------- ---------- ---
2A2_gi|1835701|      ---------- ---------- ---------- ---------- ---
2B1_gi|401428|       IFV------- ---------- ---------- ---------- ---
2B2_gi|1351442|      TFI------- ---------- ---------- ---------- ---
2B3_gi|2492803|      TFV------- ---------- ---------- ---------- ---
2B4_gi|1912051|      IFV------- ---------- ---------- ---------- ---
2B5_gi|3289019|      IFV------- ---------- ---------- ---------- ---
2B6_gi|6321895|      KWAHYYKKF- ---------- ---------- ---------- ---
2B7_gi|1912049|      IFV------- ---------- ---------- ---------- ---
2B8_gi|4103055|      TFI------- ---------- ---------- ---------- ---
2C1_gi|1332539|      LFD------- ---------- ---------- ---------- ---
2D1_gi|7407095|      IF-------- ---------- ---------- ---------- ---
2E1_gi|4753912|      FDMVPNPIPD PFRSEMKS-- ---------- ---------- ---
2E2_gi|18479021|     IFD------- ---------- ---------- ---------- ---
2E3_gi|5052610|      FNIEF----- ---------- ---------- ---------- ---
3A1_gi|121087|       FEVFK----- ---------- ---------- ---------- ---
3A2_gi|6320576|      IFQ------- ---------- ---------- ---------- ---
3B1_gi|1142698|      CKNAVKIK-- ---------- ---------- ---------- ---
3B2_gi|60458781|     INA------- ---------- ---------- ---------- ---
3B3_gi|60458785|     INTNL----- ---------- ---------- ---------- ---
3C1_gi|536474|       GNGPNLDNLK Y--------- ---------- ---------- ---
3C2_gi|74626610|     ---------- ---------- ---------- ---------- ---
3C3_gi|38423524|     FHEQFKDL-- ---------- ---------- ---------- ---
3D1_gi|31321885|     FPDKVGQ--- ---------- ---------- ---------- ---
3E1_gi|22207641|     EETAKNLSA- ---------- ---------- ---------- ---
3F1_gi|81625481|     ---------- ---------- ---------- ---------- ---
3F2_gi|13638516|     EWD------- ---------- ---------- ---------- ---
3F3_gi|81635765|     DWDR------ ---------- ---------- ---------- ---
4A1_gi|112837|       LWDDQI---- ---------- ---------- ---------- ---
```

```
4A2_gi|75220959|        LYDD------ ---------- ---------- ---------- ---
4A3_gi|1215788|         LWDDEL---- ---------- ---------- ---------- ---
4A4_gi|1514979|         LWDDEI---- ---------- ---------- ---------- ---
4B1_gi|2792155|         LWDGDV---- ---------- ---------- ---------- ---
4B2_gi|6478210|         FWDEKD---- ---------- ---------- ---------- ---
4B3_gi|6478204|         FWDEKD---- ---------- ---------- ---------- ---
4B4_gi|2792295|         VDEEL----- ---------- ---------- ---------- ---
4B5_gi|112807104|       LWAGEI---- ---------- ---------- ---------- ---
4B6_gi|112807098|       LWDGEI---- ---------- ---------- ---------- ---
4B7_gi|112807100|       LWDGEI---- ---------- ---------- ---------- ---
4B8_gi|112807102|       LWDGEI---- ---------- ---------- ---------- ---
4C1_gi|113595|          VWDHEN---- ---------- ---------- ---------- ---
4C2_gi|167113|          VWDNEN---- ---------- ---------- ---------- ---
4C3_gi|75221432|        VWDHEN---- ---------- ---------- ---------- ---
4C4_gi|4539944|         LWDGEV---- ---------- ---------- ---------- ---
4C5_gi|13160397|        VHPEISQYKT VEEMWDGGI- ---------- ---------- ---
4C6_gi|13160399|        VHPEISQYKT VEEMWDGGI- ---------- ---------- ---
4C8_gi|111182163|       LWDGEI---- ---------- ---------- ---------- ---
4C9_gi|111182165|       LWDGEI---- ---------- ---------- ---------- ---
4C10_gi|111182167|      LWDGEI---- ---------- ---------- ---------- ---
4C11_gi|111182169|      LWDGEI---- ---------- ---------- ---------- ---
5A1_gi|408360251|       --AQF----- ---------- ---------- ---------- ---
5A2_gi|11127591|        FMNDF----- ---------- ---------- ---------- ---
5B1_gi|2506173|         FAEFV----- ---------- ---------- ---------- ---
5C1_gi|144969|          ---------- ---------- ---------- ---------- ---
5C2_gi|3916039|         DPDQFGG--- ---------- ---------- ---------- ---
5D1_gi|112735|          THEEM----- ---------- ---------- ---------- ---
5E1_gi|5354195|         RYEEF----- ---------- ---------- ---------- ---
5F1_gi|82504416|        V--------- ---------- ---------- ---------- ---
5G1_gi|16080393|        F--------- ---------- ---------- ---------- ---
5G2_gi|16079957|        F--------- ---------- ---------- ---------- ---
6A1_gi|18202524|        S--------- ---------- ---------- ---------- ---
6A2_gi|499328|          S--------- ---------- ---------- ---------- ---
6A3_gi|2135947|         S--------- ---------- ---------- ---------- ---
6A4_gi|975314|          S--------- ---------- ---------- ---------- ---
6A5_gi|2827466|         S--------- ---------- ---------- ---------- ---
6A6_gi|7914984|         S--------- ---------- ---------- ---------- ---
6A7_gi|5019764|         S--------- ---------- ---------- ---------- ---
6A8_gi|148747467|       S--------- ---------- ---------- ---------- ---
6A9_gi|24648619|        IDLDQLVKQH QEWFECKENF TSLLDDILGA LKRDDENMIS DCY
6A10a_gi|726465|        S--------- ---------- ---------- ---------- ---
6A10b_gi|5019766|       S--------- ---------- ---------- ---------- ---
6A11_gi|5922729|        A--------- ---------- ---------- ---------- ---
6A13_gi|52001073|       S--------- ---------- ---------- ---------- ---
6A14_gi|226823214|      ---------- ---------- ---------- ---------- ---
6B1_gi|902000|          TLALR----- ---------- ---------- ---------- ---
6C1_gi|1063415|         RPESYR---- ---------- ---------- ---------- ---
6C2_gi|2832783|         RPESYR---- ---------- ---------- ---------- ---
7A1_gi|39932720|        ---------- ---------- ---------- ---------- ---
7A2_gi|41327764|        ---------- ---------- ---------- ---------- ---
7A3_gi|41152114|        ---------- ---------- ---------- ---------- ---
7A4_gi|6815049|         ---------- ---------- ---------- ---------- ---
8A1_gi|24638123|        YNEQLAGTLS V--------- ---------- ---------- ---
8A2_gi|74627022|        LSI------- ---------- ---------- ---------- ---
9A1_gi|146345520|       KPRPIEPKQG YKQMDRK--- ---------- ---------- ---
9A2_gi|6093525|         KQQPIEPFQG AKYFGSASK- ---------- ---------- ---
9A3_gi|2492798|         AQQAIRPQK- ---------- ---------- ---------- ---
9B1_gi|6323998|         AFDN------ ---------- ---------- ---------- ---
9B2_gi|6319951|         TNNV------ ---------- ---------- ---------- ---
9B3_gi|6319958|         ---------- ---------- ---------- ---------- ---
9B4_gi|6322615|         TEMSAKISFE D--------- ---------- ---------- ---
9C1_gi|2459734|         ---------- ---------- ---------- ---------- ---
10A1_gi|4731595|        ---------- ---------- ---------- ---------- ---
10A2_gi|3256056|        ---------- ---------- ---------- ---------- ---
11A1_gi|1176985|        ---------- ---------- ---------- ---------- ---
11B1_gi|3123233|        TREEI----- ---------- ---------- ---------- ---
11B2_gi|3123121|        ---------- ---------- ---------- ---------- ---
11B3_gi|85062654|       LLWNW----- ---------- ---------- ---------- ---
11C1_gi|81787577|       QHRE------ ---------- ---------- ---------- ---
12A1_gi|5305791|        AWQN------ ---------- ---------- ---------- ---
12B1_gi|2599278|        AWLQ------ ---------- ---------- ---------- ---
12C1_gi|5921163|        AWLS------ ---------- ---------- ---------- ---
13A1_gi|1351673|        FMDTPPMPK- ---------- ---------- ---------- ---
13B1_gi|9106797|        ---------- ---------- ---------- ---------- ---
13C1_gi|81555851|       MVNQ------ ---------- ---------- ---------- ---
14A1_gi|882530|         ASSDK----- ---------- ---------- ---------- ---
15A1_gi|37196700|       ---------- ---------- ---------- ---------- ---
all2316_aldo/keto       ---------- ---------- ---------- ---------- ---
```
