# Supplementary material for: A Novel Aldo-Keto Reductase (AKR17A1) of Anabaena sp. PCC 7120 Degrades the Rice Field Herbicide Butachlor and Confers Tolerance to Abiotic Stresses in E. coli
Source: PLoS One. 2015 Sep 15;10(9):e0137744. doi: 10.1371/journal.pone.0137744 (PMC4570671; doi:10.1371/journal.pone.0137744)
Supplement: S3 File — Real time quantitative RT PCR analysis results (Table A). SDS-PAGE (12%) analysis of recombinant protein in E. coli BL21 (DE3). (Fig A). Immunoblot detection of AKR17A1 protein in E. coli transformed with recombinant (R) and empty vectors (E) (Fig B). Aldo/keto reductase activity at various concentrations of (a) benzaldehyde, (b) ethyl pyruvate, (c) methyl glyoxal, (d) isatin, (e) o-nitro benzaldehyde and (e) butachlor (Fig C). GC-MS spectra of butachlor and metabolites produced after degradation (Fig D). Absorption spectra of NADPH in reaction mix without butachlor (Fig E). Comparison of AKR171A1 activity for isatin and o-nitrobenzaldehyde with other characterized AKRs (Table B). (DOCX) [file pone.0137744.s003.docx]

**Table A.** Real time quantitative RT PCR analysis results.

| **Sample name** | **Relative Normalized expression** | **Fold increase** |
| --- | --- | --- |
| Control | 0.27010 ± 0.04931 | - |
| Butachlor | 1.02329 ± 0.16036 | 3.788557 |
| Cd | 1.10780 ± 0.04512 | 4.101449 |
| UV-B | 1.13309 ± 0.10230 | 4.195083 |
| Heat | 1.13258 ± 0.11115 | 4.19318 |
| Salt | 0.59586 ± 0.12118 | 2.206062 |
| Drought | 0.71257 ± 0.03515 | 2.638166 |
| As | 0.83880± 0.05463 | 3.105533 |

**Figure A.** SDS-PAGE (12 %) analysis of recombinant protein in *E. coli* BL21 (DE3). Lane (M) protein marker, (L1) whole cell lysate of *E. coli* cells containing recombinant plasmid pET-21a-AKR without IPTG induction (L2) cell lysate obtained after 16 h induction with 0.05 mM IPTG at 16˚C, and (L3-L5) purified recombinant protein AKR17A1. The number on the right side is the apparent molecular weight of the recombinant AKR17A1 protein (31 kDa).


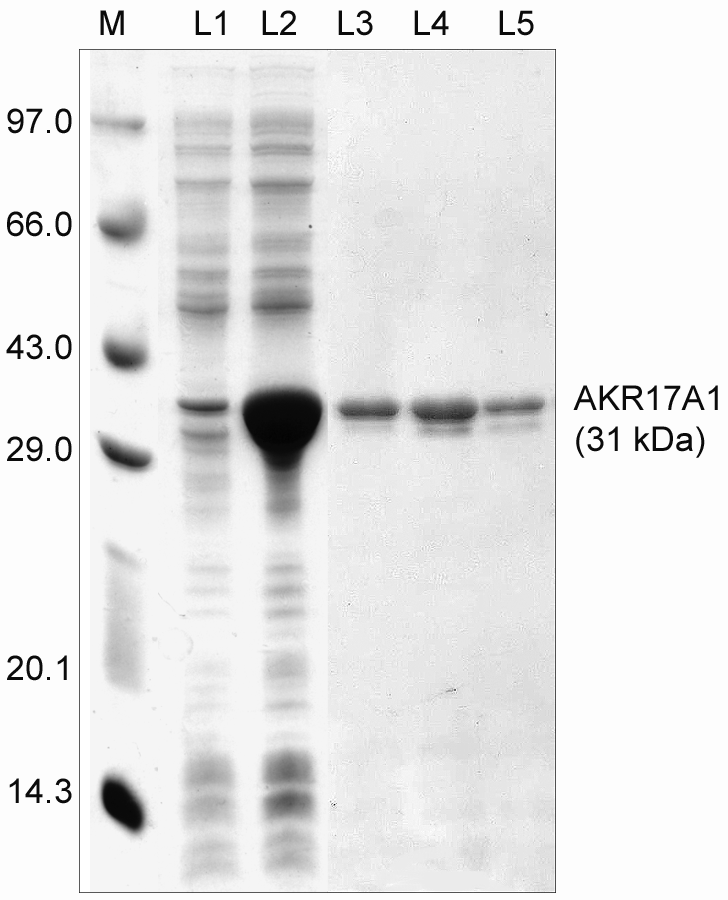


**Figure B.** Immunoblot detection of AKR17A1 protein in *E. coli* transformed with recombinant (R) and empty vectors (E).

**R E**


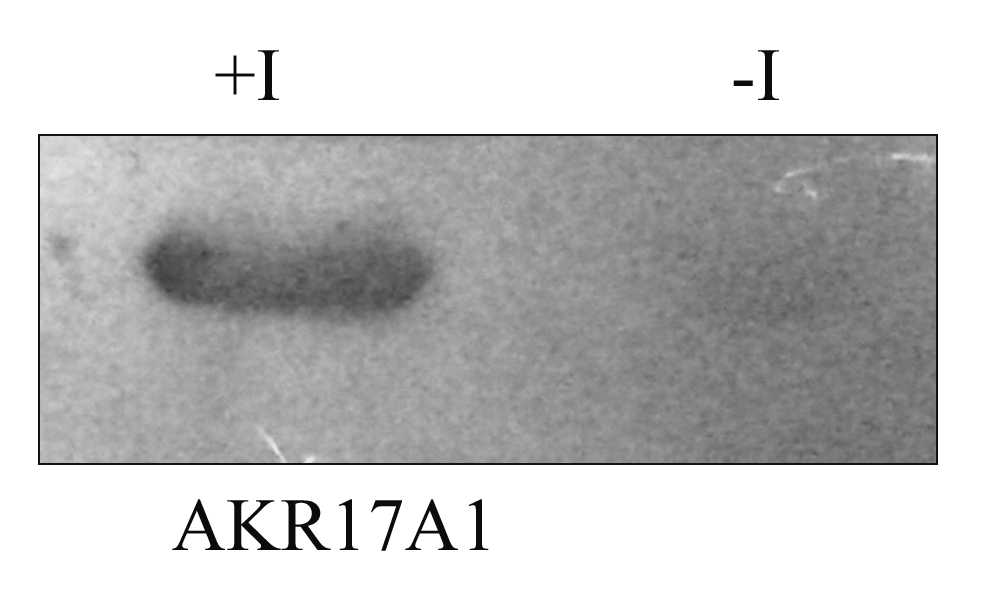


**Figure C.** Aldo/keto reductase activity at various concentrations of (a) benzaldehyde, (b) ethyl pyruvate, (c) methyl glyoxal, (d) isatin, (e) o-nitro benzaldehyde and (f) butachlor. Enzyme activities were assayed in triplicate. Bars indicate ±SD.







(a) (b)







(c) (d)







(e) (f)

**Figure D.** GC-MS spectra of butachlor and metabolites produced after degradation.

1. Butachlor

1. 1,2-benzenedicarboxylic acid

(C) 2,6-bis(1,1-dimethylethyl)-4-methyl Phenol

**Figure E.** Absorption spectra of NADPH in reaction mix without butachlor

**

**

**Table B. Comparison of AKR171A1 activity for isatin and o-nitrobenzaldehyde with other characterized AKRs**

| **AKR** | **Km (mM)** | **Kcat/Km**  **(M^-1^ min^-1^)** | **Reference** |
| --- | --- | --- | --- |
| **Isatin** | | | |
| **AKR17A1** | 0.011 ±0.005 | 81.79×10^5^ |  |
| **AKR14A1** | 2.05 ±1.36 | 5.35×10^4^ | Grant et al 2003 |
| **AKR8A1** | NMA | - | Nakano et al., 1999 |
| **AKR2E4** | 0.063 ±0.0025 | 7.2×10^2^ | Yamamoto et al., 2013 |
| **AKR4C8** | 0.035±0.009 | 51.58×10^5^ | Simpson et al., 2009 |
| **AKR4C9** | vh | 9.1×10^4^ | Simpson et al., 2009 |
| **o-nitrobenzaldehyde** | | | |
| **AKR17A1** | 0.014 ±0.002 | 68.12×10^5^ |  |
| **AKR11B3** | 0.31±0.03 | 2.66×10^5^ | Xu et al., 2006 |
| **AKR8A1** | 0.096±0.009 | 3.5×10^5^ | Nakano et al., 1999 |

NMA, not measurable activity

vh, very high; could not be fitted with any accuracy to the Michaelis–Menten equation.
